# Exome variants associated with asthma and allergy

**DOI:** 10.1038/s41598-022-24960-6

**Published:** 2022-12-05

**Authors:** Matthias Wjst

**Affiliations:** 1 Institute of Lung Health and Immunity (LHI), Helmholtz Zentrum München - German Research Center for Environmental Health, Ingolstädter Landstr. 1, 85764 Neuherberg, München, Germany; 2 Institut für KI und Informatik in der Medizin, Lehrstuhl für Medizinische Informatik, Klinikum Rechts der Isar, Grillparzerstr. 18, 81675 München, Germany

**Keywords:** Target identification, Asthma, Diagnostic markers, Predictive markers, Genome-wide association studies

## Abstract

The mutational spectrum of asthma and allergy associated genes is not known although recent biobank based exome sequencing studies included these traits. We therefore conducted a secondary analysis of exome data from 281,104 UK Biobank samples for association of mostly rare variants with asthma, allergic rhinitis and atopic dermatitis. Variants of interest (VOI) were tabulated, shared genes annotated and compared to earlier genome-wide SNP association studies (GWAS), whole genome sequencing, exome and bisulfit sequencing studies. 354 VOI were significantly associated with asthma, allergic rhinitis and atopic dermatitis. They cluster mainly in two large regions on chromosome 6 and 17. After exclusion of the variants associated with atopic dermatitis and redundant variants, 321 unique VOI remain in 122 unique genes. 30 genes are shared among the 87 genes with increased and the 65 genes with decreased risk for allergic disease. 85% of genes identified earlier by common GWAS SNPs are not replicated here. Most identified genes are located in interferon ɣ and IL33 signaling pathway. These genes include already known but also new pharmacological targets, including the IL33 receptor ST2/IL1RL1, as well as TLR1, ALOX15, GSDMA, BTNL2, IL13 and IKZF3. Future pharmacological studies will need to included these VOI for stratification of the study population paving the way to individualized treatment.

It has been a long way from the first molecular study of David Marsh in the early seventies^1^ and the Transatlantic Airway Conference 1997 in Key Biscayne where the first positional candidate gene from Tristan da Cunha was announced^2^. A genome-wide linkage scan^3^ confirmed the chromosome 6 linkage, while only the following two studies tagged chromosome 17^4^ or chromosome 2 and 9^5^ where later the IKZF3-ORMDL3-GSDMA cluster or IL33 and its receptor^6^ could be identified^7^. The genomic resolution was however poor during that time. Only until thousands of SNPs had been discovered, the technology opened the door to hundreds of new association studies. It was reaching a new height by a meta-analysis of 180,000 cases^8^ in 2017 but unfortunately left the genetics of asthma and allergy field basically stuck with long lists of SNPs^9^. The situation changed only by the last year when two ultra-large exome scans released the variants of 281,000 respective 455,000 participants of the UK biobank^10^.

Rare exon variants^11^ are not uniformly distributed across the genome but depend on many factors from sequence context to selective pressure in the population. Highly deleterious mutations will not be transmitted to offspring so mainly missense and synonymous variants are found with milder but nevertheless important effects. As most of these variants are in the coding region of genes, they are expected to identify directly functional active genes just like knock-out or knock-in experiments in animals. As humans are outbred, a significantly increased and decreased risk by a mutation may not be associated with disease due to the variable genetic background or possible rescue mutations^12^. Nevertheless a significant association of a mutation with reasonable effects size will highlight the chain of events protecting from or leading to complex disease.

Although the recent biobank based exome studies included thousands of different traits, no particular attention has been given so far to allergy and asthma. A secondary analysis of this large dataset is therefore timely.

## Methods

The 450 K datasets is being used here, which is a set of around 450,000 exome sequenced participants that is of high quality, predominantly unrelated, and mainly of European ancestry (n = 394,695). Only allelic results are given here which may lead to some bias as associations had been pre-filtered for p ≤ 0.01 and only variants that had been identified in at least 30 samples. The 450 K dataset is based on UKB Phenotypic Release v41065 plus Hospital Episode Statistics HES and an extended set of the so called 300 K dataset^10^. This smaller set includes 269,171 individuals of European ancestry that is enriched for loss of function mediated traits and approved drug targets with additional data from 11,933 UKB participants of African, East or South Asian ancestry.

For allergic rhinitis the variable "#20002#1387#hayfever|allergic_rhinitis" was selected which is defined as "non cancer illness code, self-reported hayfever/allergic rhinitis" and includes 22,310 cases and 307,542 controls resulting in a disease prevalence of 7%. For atopic dermatitis the variable "Union#L20#L20 Atopic dermatitis" was selected which is defined as ICD Code L20 atopic dermatitis "category contains summary fields relating to diagnoses made during hospital inpatient admissions" and includes 10,953 cases and 361,503 controls (3% prevalence). For asthma the variable "#6152#8#Asthma (UK Biobank)" was selected which is defined by data field 6152 "asthma diagnosed by doctor." As several known gene associations including IL33 were not found using this definition instead "Union#J45#J45 Asthma" was selected which is defined as ICD Code J45 asthma "category contains summary fields relating to diagnoses made during hospital inpatient admissions") including 57,594 cases and 314,471 controls (15% prevalence).

Sequencing data for UK Biobank participants were generated at the Regeneron Genetics Center as described before^13^ in a collaboration between AbbVie, Alnylam Pharmaceuticals, AstraZeneca, Biogen, Bristol-Myers Squibb, Pfizer, Regeneron and Takeda with the UK Biobank. Genomic DNA underwent paired-end 75 bp whole exome sequencing using the IDT xGen v1 capture kit on the NovaSeq 6000 platform. The kit consists of 5′ biotin–modified oligonucleotide probes that are individually synthesized and analyzed by electrospray ionization-mass spectrometry (ESI-MS) covering (v2) 415,115 probes that spans a 34 Mb target region (19,433 genes) of the human genome.

For the present analysis reference sequence BSgenome.Hsapiens.UCSC.hg38 was selected along with genomic coordinates hg38. Earlier data were converted to this format using the Golden Path Liftover tools from https://hgdownload.cse.ucsc.edu/goldenpath/hg19/liftOver. For gene names HUGO nomenclature^13^ (https://www.genenames.org) was used while in a few cases SNPs or genes were matched using surrounding base sequence. Exome data were downloaded on Jan, 18, 2022 from https://azphewas.com.

Using R software, the tidyverse framework and the biomaRt library, gene transcript data were obtained on Jan, 25, 2022 from https://www.ensembl.org/biomart/martview. GWAS data were already received by Nov, 3, 2017 from https://genepi.qimr.edu.au/staff/manuelf/gwas_results/intro.html This dataset is not complete as 23andme did not respond to repeated requests for full result sharing although promised in^8^. Linkage disequilibrium data were downloaded on Nov, 11, 2017 from https://data.broadinstitute.org/alkesgroup/LDSCORE. Gene set enrichment and pathway analysis were obtained of the Enrichr API https://maayanlab.cloud/Enrichr^14^ including Reactome and Human Gene Atlas pathways. Data were plotted using ggplot and gviz libraries. The data analysis pipeline is available under https://github.com/under-score/allergy_exome.

## Results

For the trait allergic rhinitis allelic results of 6917 variants exome variants were downloaded, leading to 50 significantly associated variants of interest (VOI). For asthma 9695 VOI were identified, where 302 allelic VOI remain after applying a stringent significance threshold of P ≤ 2 × 10^–9^. For atopic dermatitis 5965 VOI were found but only 2 significant VOI remain after filtering. Both VOI, 1_152312600 and 1_152313385 are located in filaggrin. There are a few more missense variants with odds ratios > 3 for allergic rhinitis and > 5 for asthma that are not significant by the applied genome-wide threshold.

**Figure 1:**
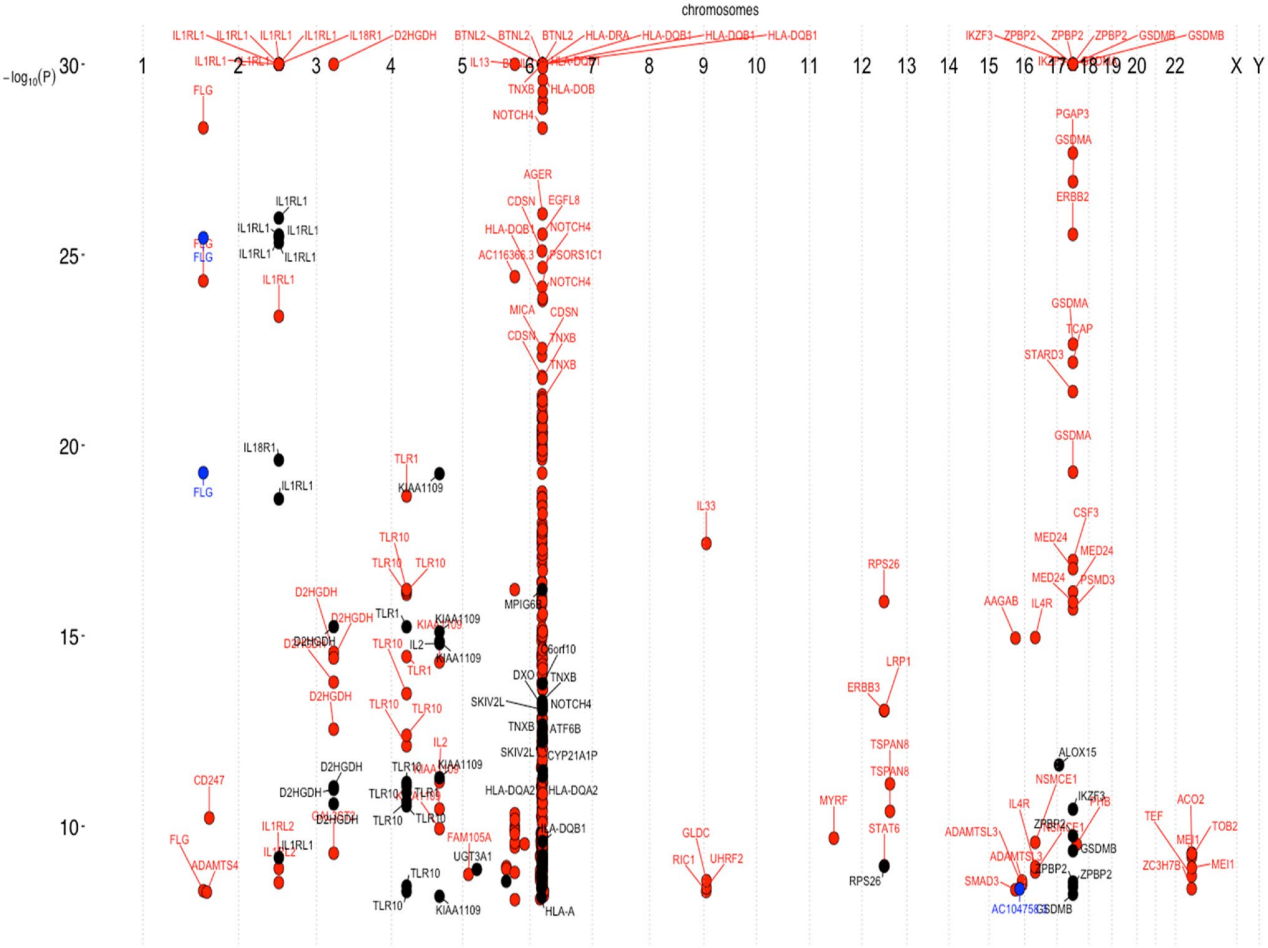
Exon variants of interest (N = 354) significantly associated with asthma, allergic rhinitis or atopic dermatitis in the 450 K UK Biobank samples. The plot shows significance using a threshold of P ≤ 2 × 10^–9^ versus chromosomal position as Manhattan plot. Atopic dermatitis is indicated by blue dots, asthma by red dots and allergic rhinitis by black dots. Variant clustering is observed at chromosome 6 and 17.

**Figure 2:**
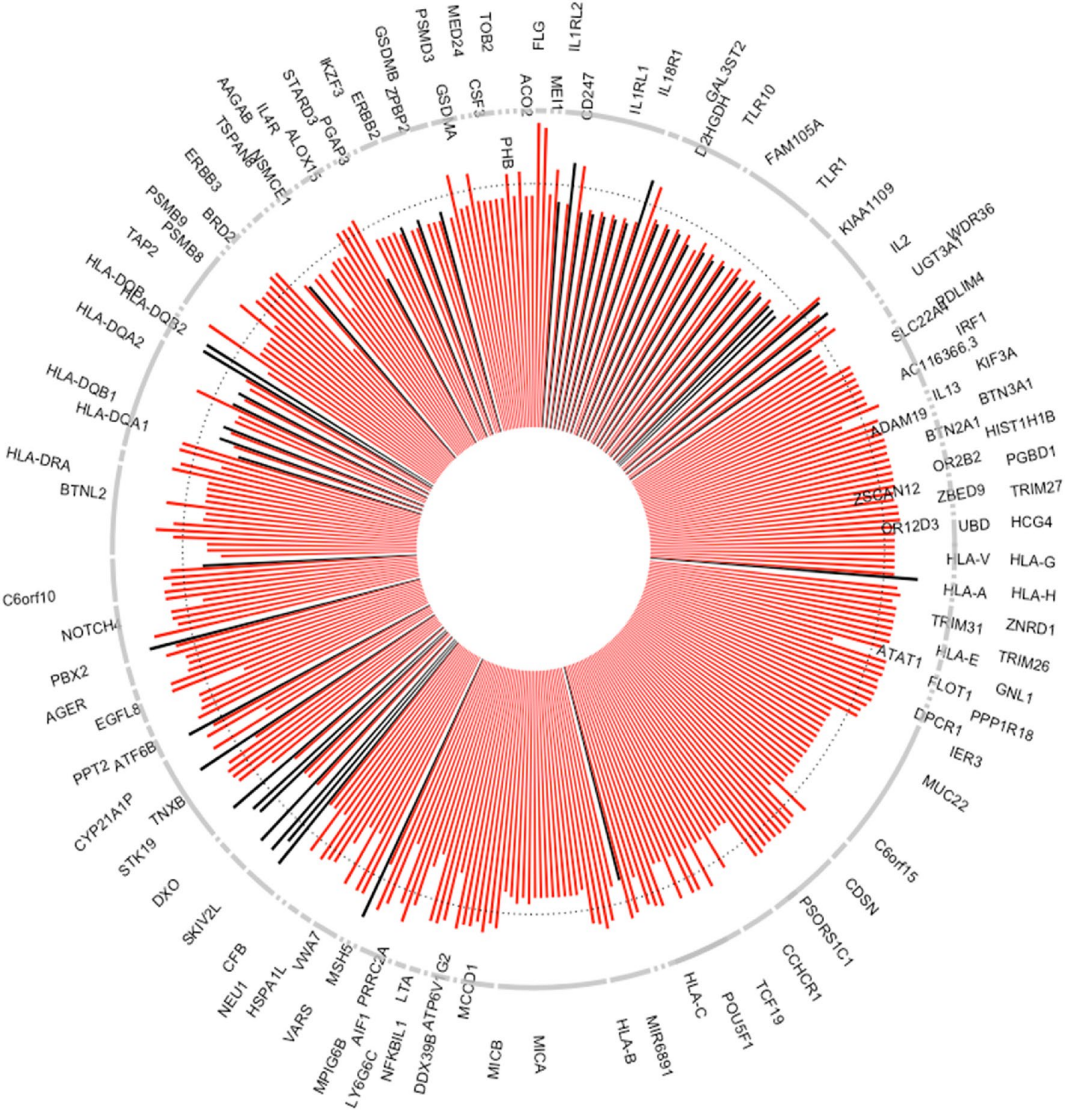
Exon variants of interest (N = 352) significantly associated with asthma or allergic rhinitis in the 450 K UK Biobank samples. The plot shows odds ratios of variants filter by a threshold of P ≤ 2 × 10^–9^ as circular barplot where OR of 1 is indicated by a dotted line. Results for asthma are shown as red bars, allergic rhinitis as black bars. There were more variants associated with asthma than with allergic rhinitis while also number of variants per gene is highly variable with most VOI seen in MUC22 (N = 18), MICA (N = 15) and IL1RL1 (N = 14).

**Figure 3:**
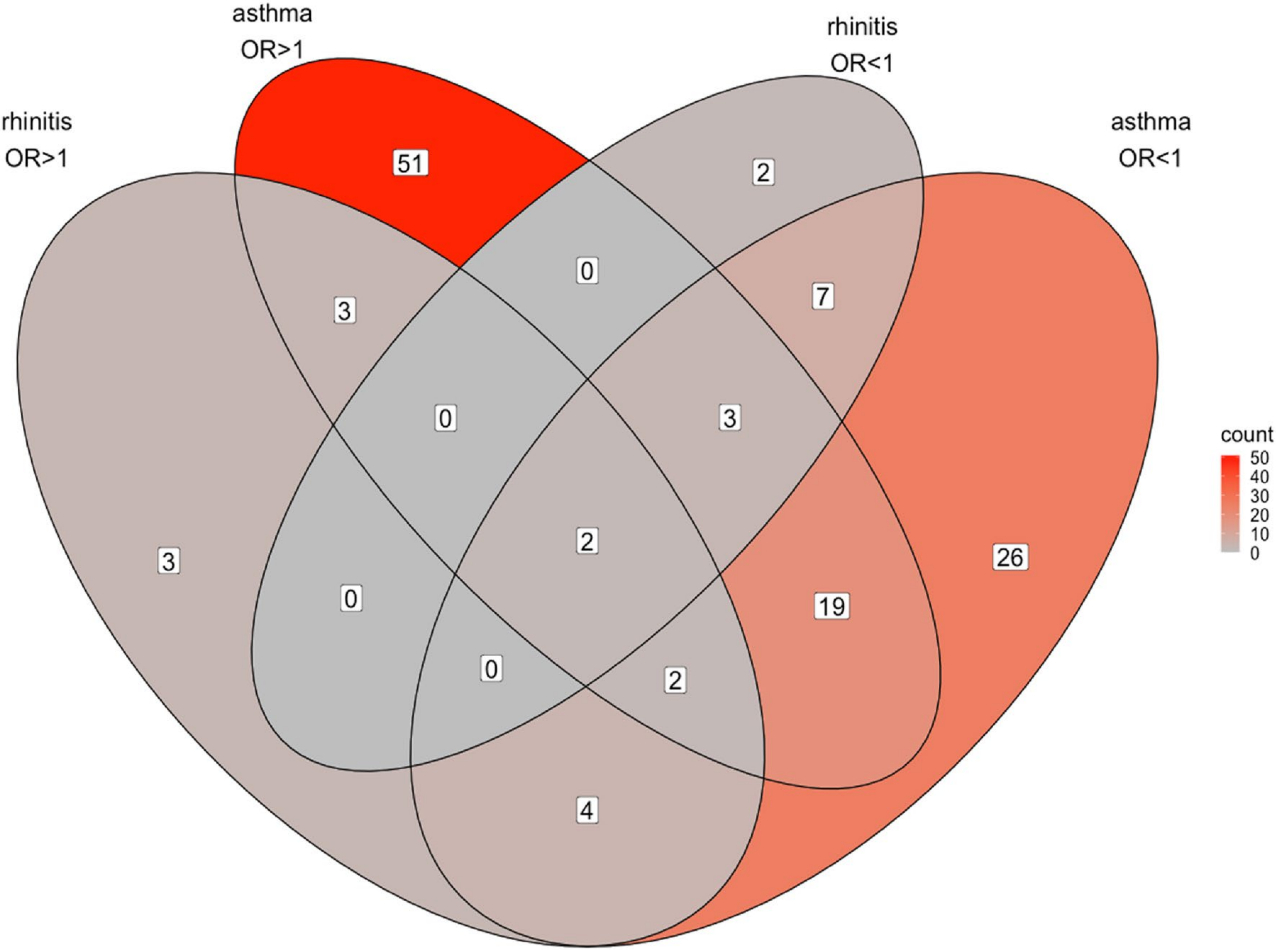
Shared genes (N = 122) significantly associated with asthma or allergic rhinitis in the 450 K UK Biobank sample by either increased or decreased odds ratios. Most genes are observed in the group with increased asthma risk, followed by the group with decreased asthma risk.

Genome positions for all VOI are given in Fig. 1 as well as in the supplemental table. Clearly, there are two major gene cluster on chromosome 6 and 17 that are known from earlier association studies. After exclusion of the two atopic dermatitis variants, 352 VOI remain of which 321 are unique. They are situated in 122 unique genes while there is an overlap of 30 genes in the 87 genes with increased and the 65 genes associated with decreased odds ratios (Figs. 2 and 3). Most VOI are leading to missense base exchanges (41%), followed by synonymous (30%), non coding (12%) and splice (5%) variants among others (Fig S1). Associated VOI have rather low allele frequencies (Fig S2) and modest odds ratios (Fig S3). Most interesting are missense variants of high allele frequency but decreased risk for asthma and allergic rhinitis (Fig S4 until Fig S10) where primarily VOI in IL1RL1 (IL33 receptor), PGAP3 (a phospholipase), IKZF3 (B cell transcription factor), ZPBP2 (zona pellucida binding protein) and GSDMB (gasdermin) are interesting as they have been identified already in earlier association studies. Fig S13 to S127 contain genomic plots of the identified genes.

An annotation of the VOI showed similar pathways for protective and risk VOI. The interferon ɣ pathway carries both VOI with increased but also decreased risk which is also found with general cytokine signaling (Figs. S11 and S12). Dendritic cells und B seem to be key immune tissues along with genes active in the reproductive tract.

As a last question we ask if there are any shared genes with earlier GWAS studies. When comparing the current set of 122 genes with the 193 genes of a recent GWAS meta-analysis^8^) there is only a small overlap of 21 shared genes, including IL1RL1, IL18R1, D2HGDH, TLR1, KIAA1109, RPS26, ALOX15, ZPBP2, GSDMB, FLG, CD247, FAM105A, WDR36, SLC22A4, IL13, HLA-C, PRRC2A, ERBB3, STAT6, AAGAB and MEI1.

## Discussion

Exome sequencing has been around now for a decade^15^ and is increasingly used also in clinical diagnostics. While the wet technology is already advanced, data analysis remains a bottleneck even by using preprocessed data.

At present false positive or negative findings cannot be excluded as there is not a second sample of that size. Although we can assume from the large UK Biobank that there are no genotyping errors, that phenotypes have been correctly ascribed and data correctly handled there is still some risk of the known “Weisburd’s paradox”, the difficulty in maintaining quality control as studies get larger^16^. Non-exon and some ultra-rare exonic variants are not included in the current analysis, neither are copy number variants^17^. There are also two more paper from the UK Biobank published during revision of the current paper including the association of eosinophil counts with ALOX15, CSF2RB, IL17RA, IL33, JAK2, S1PR4, SH2B3, NPAT and RMI1^18^ as well as an abandoned preprint^19^ from the Scandinavian Asthma Genetic Study that includes also UK Biobank participants highlighting protein-truncating variants in FLG and IL33.

Atopic dermatitis. It seems from this analysis, that the genetics of atopic dermatitis is clearly separated from allergic rhinitis and asthma. Atopic dermatitis is already sufficiently described by filaggrin mutations^20^ which is in line with our earlier observation that atopic dermatitis is genetically a largely different disease^21^. Filaggrin mutations may act on top of an allergic predisposition while there are no further shared variants in this analysis.

Asthma. The asthma risk is increased by a large number of variants. Most of them are being private and basically limited to two genomic regions in known inflammatory gene cluster that are thought to be jointly regulated^22^. The asthma risk can be diminished by a number of missense variants in the same gene cluster, requiring now more detailed molecular studies. It should be noted that also VOI in pseudogenes may have functional effects by partial recombination with functional genes or by providing inactive transcription factor bindings sites^23^. Also synonymous base exchanges^24^ may affect the translational efficiency by indirect effects on mRNA stability.

Allergic rhinitis risk is increased by a much lower number of variants that are also much more scattering in the human genome than the asthma VOI. A major peak is found at chromosome 2 that is mainly caused by IL1RL1/ST2, the IL33 receptor^25^.

Earlier GWAS. While there are numerous explanations for the "missing heritability"^26,27^ there are less explanations of the "vague associations" namely SNP associations without any known structural or functional explanation. The focus on the common disease/common variants may have lead to inadequate conclusions as inflated statistical methods have led to synthetic associations^28^. There might be local linkage disequilibrium effects along with undetectable selective sweeps while many recent GWAS hits are functional irrelevant until proven otherwise. To resolve the apparent discrepancy of the numbers of significant GWAS SNPs and significant exon variants, a combined analysis of common and rare variants in the same individuals will be a future research question.

WES (whole genome exome sequencing). De Wan^29^ sequenced a nuclear family with 4 children where two children have been suffering of asthma. None of the 10 variants described in this paper could be found here. Backman^30^ reported two associations in the UK Biobank sample, the missense variant G>C 1_153775113 in SLC27A3 (OR_asthma_ = 0.65, *p* = 8.2 × 10^–8^) as well as another G>C variant 9_6255967 in IL33 (OR_allergy_ = 0.60, *P* = 9.52 × 10^–27^). The first variant is missing from the current dataset probably as there were not enough carriers. The second VOI is also found in the current dataset with similar effect size (see page 28 of the supplement) and identical to rs146597587 described earlier^31^ which has a strong effect by disrupting a canonical splice acceptor site of the last coding exon of IL33.

WGS (whole genome sequencing). The first large whole-genome sequencing study on asthma was published only during the revision of the current paper^32^. It includes 3181 asthma patients and 3590 controls as a follow-up of various clinical studies and shows 8 major associations at 2:102,265,885 (IL1RL2, gene but not exact match with current results), 5:111,069,301 (TSLP, no match), 6:32,634,706 (HLA-DQA1, gene match), 6:90,240,909 (BACH2, no match), 11:76,559,639 (C11orf30, no match), 14:68,287,700 (RAD51B, no match), 15:71,314,041 (THSD4, no match) and 17:39,913,818 (GSDMB, gene match)^32^. Reasons why there are so little perfect matches could be manifold. There could be technical errors by different chemistry or danalysis strategy and there is a large population heterogeneity as the result in this study is obtained of numerous clinical trials. There could be issues with selection of controls and phenotypes may have been classified in a different way. Possibly also the authors may have filtered for criteria like HWE or did not select the most important sentinel variants in the associated regions.

WBS (whole genome bisulfite sequencing). Comparing the current 122 genes with the 65 genes of a genomewide methylation scan of IgE in peripheral lymphocytes^33^ we found only one shared gene (IL1RL1). Of the 24 genes found in whole blood of children with asthma^34^ no shared gene could be identified.

Other genes. Although ORMDL3 has been repeatedly favored by the Oxford group^7^ along with their repeated claim on FcεR1ß/MS4A2^35^, a structural contribution of both genes to asthma and allergy development is unlikely from the current analysis. There is no further support that blocking MS4A2 or ORMDL3 expression^36^ would be beneficial in asthma or allergy therapy. CYSLTR2, the Tristan da Cunha asthma gene^37^ was also not tagged here neither was ADAM33^38^. FCER1A is also missing here although a formal analysis of total IgE in UK Biobank data is still pending. Genetic associations for serum IL4 and IL13 were also surprisingly weak as well as for serum IFNγ and IL10 (unpublished own observation).

Clinical relevance. As the British Biobank recruited in the beginning only participants aged 40–69 years who lived within ∼25 miles of one of the 22 assessment centers located throughout England, Wales and Scotland, results may not be generalized to the European mainland in particular when variants are ultra rare in the population. Further exome sequencing studies will therefore have to be repeated also in different populations.

As asthma and allergic diseases have their peak prevalence in childhood, some diagnoses may have been missed by examining a mid-aged population only. Even with the balanced sampling scheme and the large sample size of the UK Biobank, recent results show that there may be further biases^39^ including less health related risk factors and diseases^40^. There may be also an overlap of asthma and COPD^41^ cases that should be resolved at a later stage. In future studies, replication of this results with disease related QTL will be necessary as well as interaction testing of VOI and the construction of improved risk scores. It is an open question if the previously described interaction of variants in IL1RL1 and IL5 or IL13^42^ will be also be found with the VOI described here.

Nevertheless, the location of some variants can now been used to elucidate biological pathways. For example all IL1RL1 VOI show a negative association with allergy except variant 2_102341256 in an exon that is exclusively used by the ST2V transcript^43^ leading to an shortened version of the soluble decoy receptor sST2. If we assume a weaker IL33 blocking capacity, it seems reasonable to develop recombinant fusion proteins as alternative IL33 traps^44^.

The VOI results here provide also a basis for planning and re-analyzing clinical trials. While therapies to restore or substitute broken gene functions (eg VOI with increased disease risk) are are difficult to target at the moment, dampening or turning off genes (eg VOI that show decreased disease risk) are promising. IL33 is already one of the most relevant therapeutical targets where monoclonal antibodies including etokimab, itepekimab, tozorakimab have been tested in clinical trials. From the current analysis they are expected to have even stronger clinical effects than anti- IL4R (dupilumab), IL5 (mepolizumab, reslizumab) , IL5R (benralizumab) or IL-13 (tralokinumab) antibodies while anti ST2 antibodies like astegolimab may be even more effective. A major condition for future clinical trial will be an adequate stratification of patients as for example individuals with a deleterious ALOX5 mutation will probably not respond to zileuton, neither will patients with an already defunct IL33 receptor respond to astegolimab. There are also refined or repurposed treatment options as for example lenalidomide is targeting IKZF3^44^ or disulfiram which was used earlier to treat atopic dermatitis^45^ could be used to block gasdermins^46^. There are also new treatment possibilities of small-molecule antagonists like MMG-11 on TLR1^47^ or ZINC59514725 on IL1RL1^48^.

Taken together, UK Biobank released here the largest dataset of asthma and allergy genetics right at the 25th anniversary of the Transatlantic Airway Conference where this research took off^2^. Genetics finally unfolds the underlying biology but unfortunately there are still many efforts necessary to bring these results now from bench to bedside.

## Supplementary Information

Supplementary Information.

## Data Availability

The data analysed in the current study are available at https://azphewas.com where phenotypes can be entered in the search field resulting in URLs like https://azphewas.com/phenotypeView/7e2a7fab-97f0-45f7-9297-f976f7e667c8/eb827281-c7bc-4852-aaca-07fa69cab580/glr.
