# Supplementary material for: Exome variants associated with asthma and allergy
Source: Sci Rep. 2022 Dec 5;12:21028. doi: 10.1038/s41598-022-24960-6 (PMC9722654; doi:10.1038/s41598-022-24960-6)

Supplement

Contents

Figures general **7**

Fig S1 distribution of exome variants by functional consequence . . . 7

Fig S2 distribution of exome variants by minor allel frequency in cases . . . 8

Fig S3 distribution of odds ratios . . . 9

Fig S4 exome variants by functional consequence: minor allele frequency in cases versus odds ratio . . . 10

Fig S5 exome variants by functional consequence: minor allele frequency in cases versus odds ratio . . . 11

Fig S6 exome variants by functional consequence: minor allele frequency in cases versus odds ratio . . . 12

Fig S7 exome variants by functional consequence: minor allele frequency in cases versus odds ratio . . . 13

Fig S8 exome variants by functional consequence: minor allele frequency in cases versus odds ratio . . . 14

Fig S9 exome variants by functional consequence: minor allele frequency in cases versus odds ratio . . . 15

Fig S10 exome variants by functional consequence: minor allele frequency in cases versus odds ratio . . . 16

Fig S11 Reactome annotation of exome variants . . . 17

Fig S12 Human Gene Atlas annotation of exome variants . . . 18

Table exome variants **19**

Figures genome context **30**

Fig S13 AAGAB . . . 31

Fig S14 ACO2 . . . 32

Fig S15 ADAM19 . . . 33

Fig S16 AGER . . . 34
Fig S17 AIF1 . . . 35
Fig S18 ALOX15 . . . 36
Fig S19 ATAT1 . . . 37
Fig S20 ATF6B . . . 38
Fig S21 ATP6V1G2 . . . 39
Fig S22 BRD2 . . . 40
Fig S23 BTN2A1 . . . 41
Fig S24 BTN3A1 . . . 42
Fig S25 BTNL2 . . . 43
Fig S26 C6orf15 . . . 44
Fig S27 CCHCR1 . . . 45
Fig S28 CD247 . . . 46
Fig S29 CDSN . . . 47
Fig S30 CFB . . . 48
Fig S31 CSF3 . . . 49
Fig S32 CYP21A1P . . . 50
Fig S33 D2HGDH . . . 51
Fig S34 DDX39B . . . 52
Fig S35 DXO . . . 53
Fig S36 EGFL8 . . . 54
Fig S37 ERBB2 . . . 55
Fig S38 ERBB3 . . . 56
Fig S39 FLG . . . 57
Fig S40 FLOT1 . . . 58
Fig S41 GAL3ST2 . . . 59

Fig S42 GNL1 60
Fig S43 GSDMA 61
Fig S44 GSDMB 62
Fig S45 HCG4 63
Fig S46 HLA-A 64
Fig S47 HLA-B 65
Fig S48 HLA-C 66
Fig S49 HLA-DOB 67
Fig S50 HLA-DQA1 68
Fig S51 HLA-DQA2 69
Fig S52 HLA-DQB1 70
Fig S53 HLA-DQB2 71
Fig S54 HLA-DRA 72
Fig S55 HLA-E 73
Fig S56 HLA-G 74
Fig S57 HLA-H 75
Fig S58 HLA-V 76
Fig S59 HSPA1L 77
Fig S60 IER3 78
Fig S61 IKZF3 79
Fig S62 IL13 80
Fig S63 IL18R1 81
Fig S64 IL1RL1 82
Fig S65 IL1RL2 83
Fig S66 IL2 84
Fig S67 IL33 85

Fig S68 IL4R . . . 86
Fig S69 IRF1 . . . 87
Fig S70 KIAA1109 . . . 88
Fig S71 KIF3A . . . 89
Fig S72 LRP1 . . . 90
Fig S73 LTA . . . 91
Fig S74 LY6G6C . . . 92
Fig S75 MCCD1 . . . 93
Fig S76 MED24 . . . 94
Fig S77 MEI1 . . . 95
Fig S78 MICA . . . 96
Fig S79 MICB . . . 97
Fig S80 MIR6891 . . . 98
Fig S81 MPIG6B . . . 99
Fig S82 MSH5 . . . 100
Fig S83 MUC22 . . . 101
Fig S84 MYRF . . . 102
Fig S85 NEU1 . . . 103
Fig S86 NFKBIL1 . . . 104
Fig S87 NOTCH4 . . . 105
Fig S88 NSMCE1 . . . 106
Fig S89 OR12D3 . . . 107
Fig S90 OR2B2 . . . 108
Fig S91 PBX2 . . . 109
Fig S92 PDLIM4 . . . 110
Fig S93 PGAP3 . . . 111

Fig S94 PGBD1 . . . 112
Fig S95 PHB . . . 113
Fig S96 POU5F1 . . . 114
Fig S97 PPP1R18 . . . 115
Fig S98 PPT2 . . . 116
Fig S99 PRRC2A . . . 117
Fig S100 PSMB8 . . . 118
Fig S101 PSMB9 . . . 119
Fig S102 PSMD3 . . . 120
Fig S103 PSORS1C1 . . . 121
Fig S104 RPS26 . . . 122
Fig S105 SKIV2L . . . 123
Fig S106 SLC22A4 . . . 124
Fig S107 STARD3 . . . 125
Fig S108 STAT6 . . . 126
Fig S109 STK19 . . . 127
Fig S110 TAP2 . . . 128
Fig S111 TCAP . . . 129
Fig S112 TCF19 . . . 130
Fig S113 TLR1 . . . 131
Fig S114 TLR10 . . . 132
Fig S115 TNXB . . . 133
Fig S116 TOB2 . . . 134
Fig S117 TRIM26 . . . 135
Fig S118 TRIM27 . . . 136
Fig S119 TRIM31 . . . 137

Fig S120 TSPAN8 138
Fig S121 UBD 139
Fig S122 UGT3A1 140
Fig S123 VWA7 141
Fig S124 WDR36 142
Fig S125 ZBED9 143
Fig S126 ZPBP2 144
Fig S127 ZSCAN12 145

Figures general

Fig S1 distribution of exome variants by functional consequence

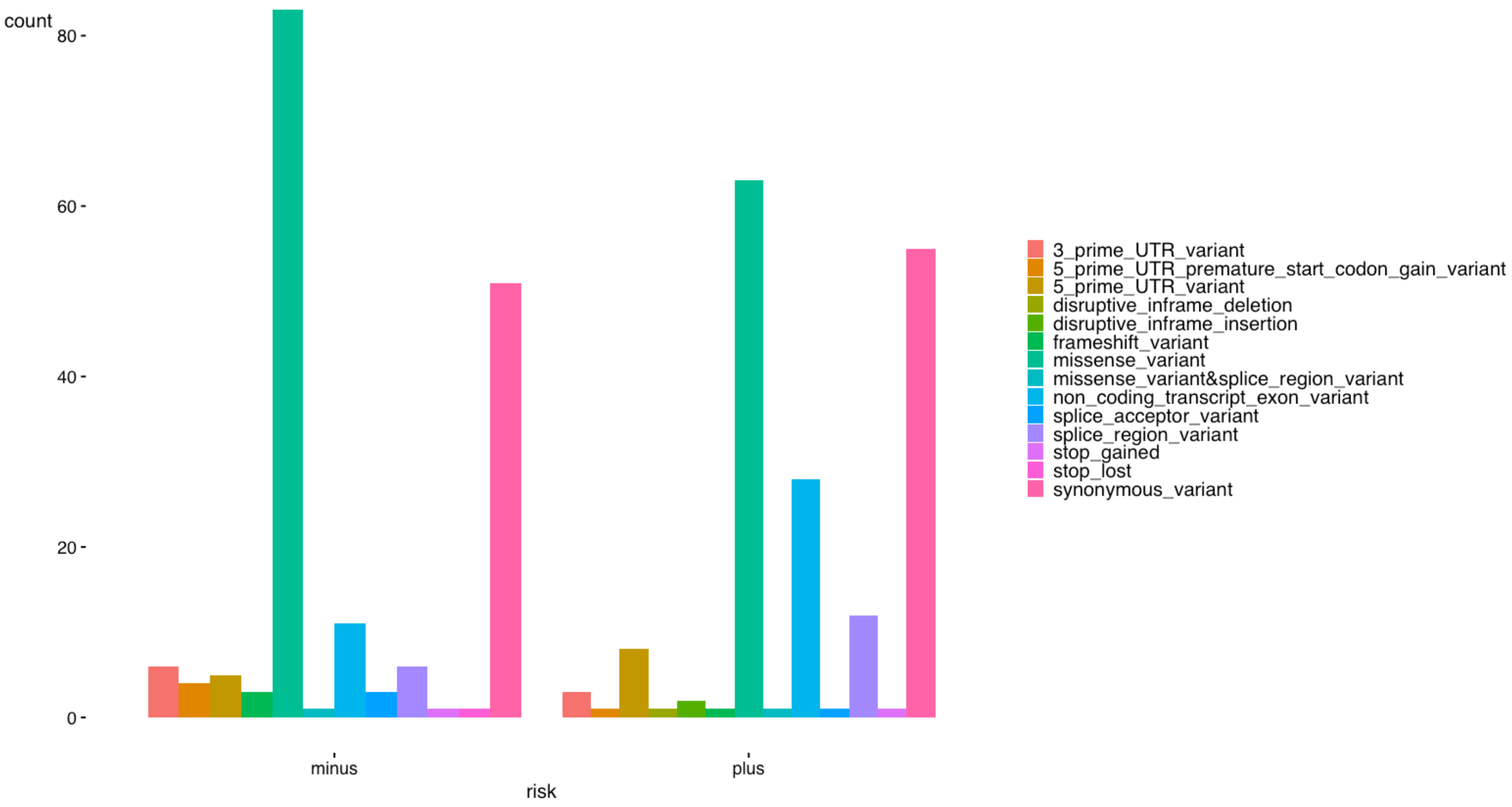

Fig S2 distribution of exome variants by minor allele frequency in cases

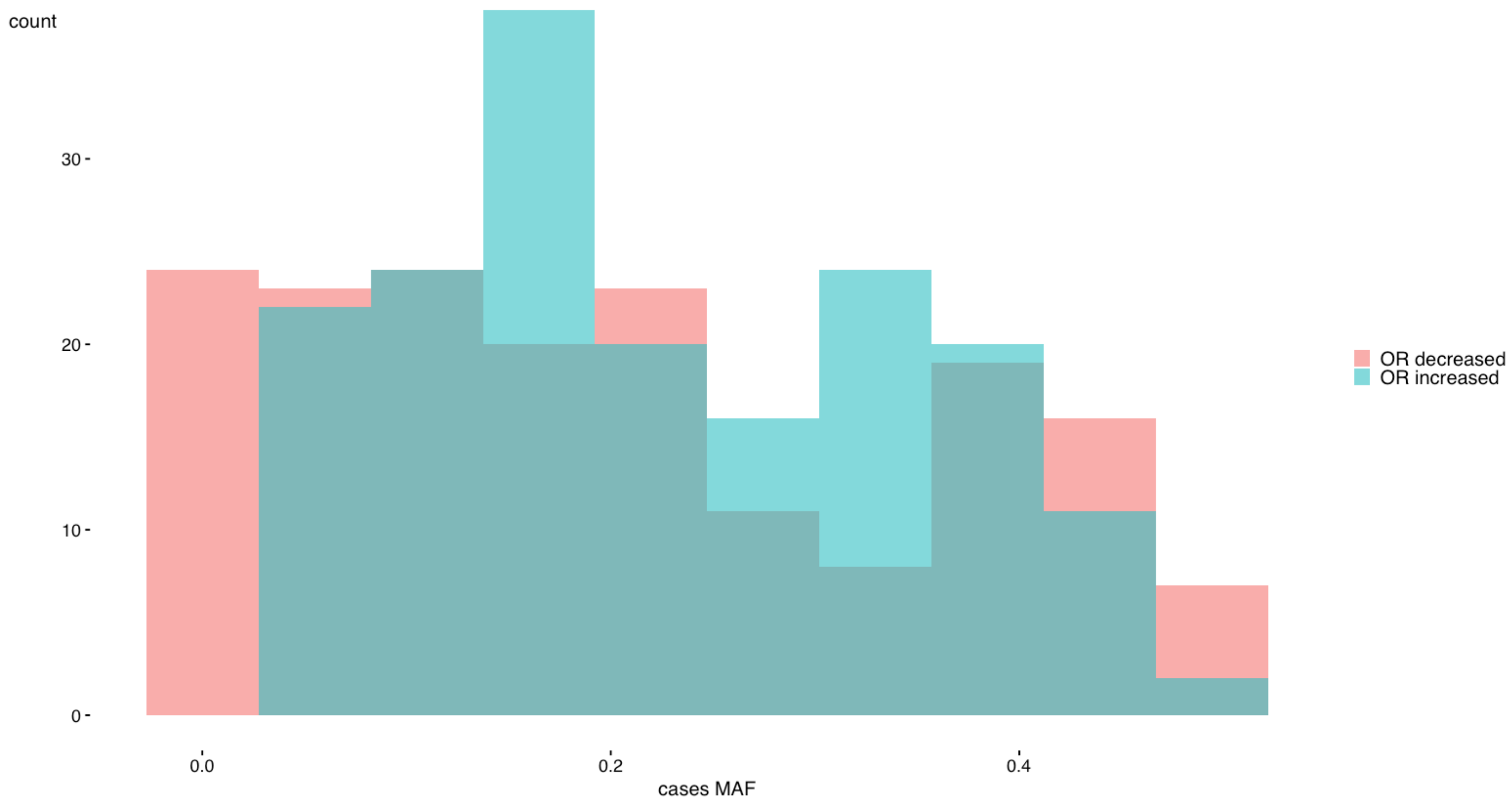

Fig S3 distribution of odds ratios

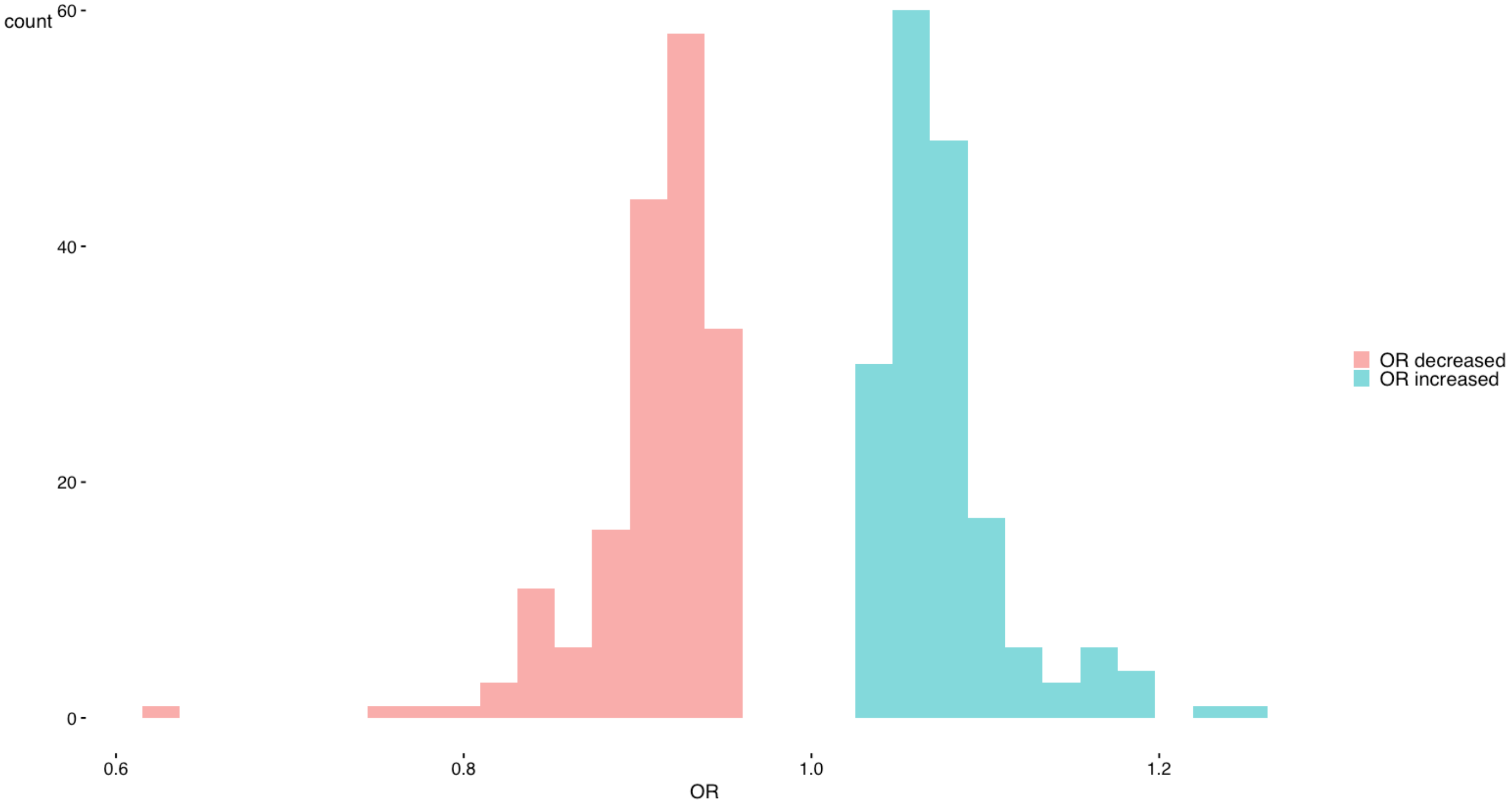

Fig S4 exome variants by functional consequence: minor allele frequency in cases versus odds ratio

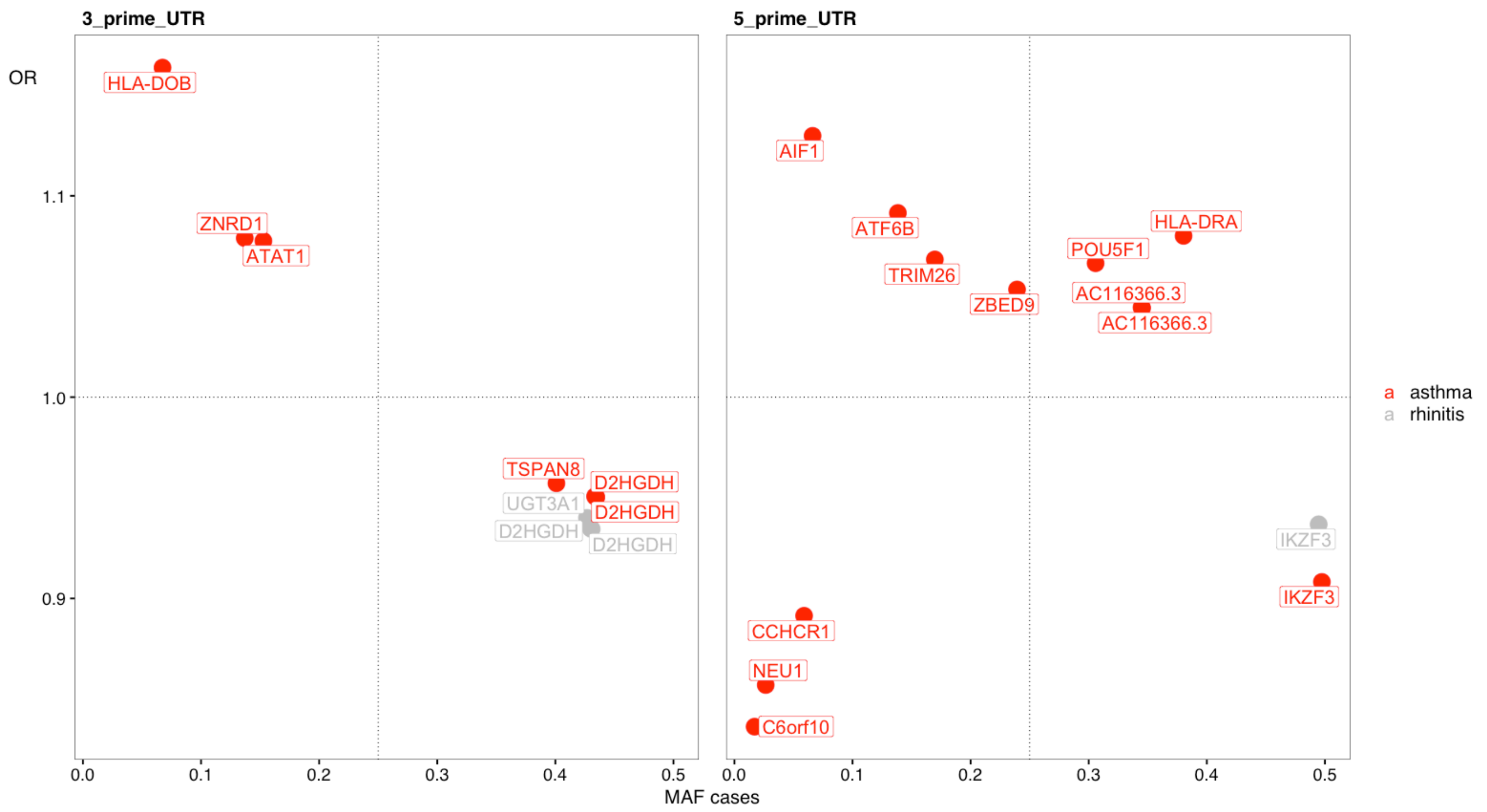

Fig S5 exome variants by functional consequence: minor allele frequency in cases versus odds ratio

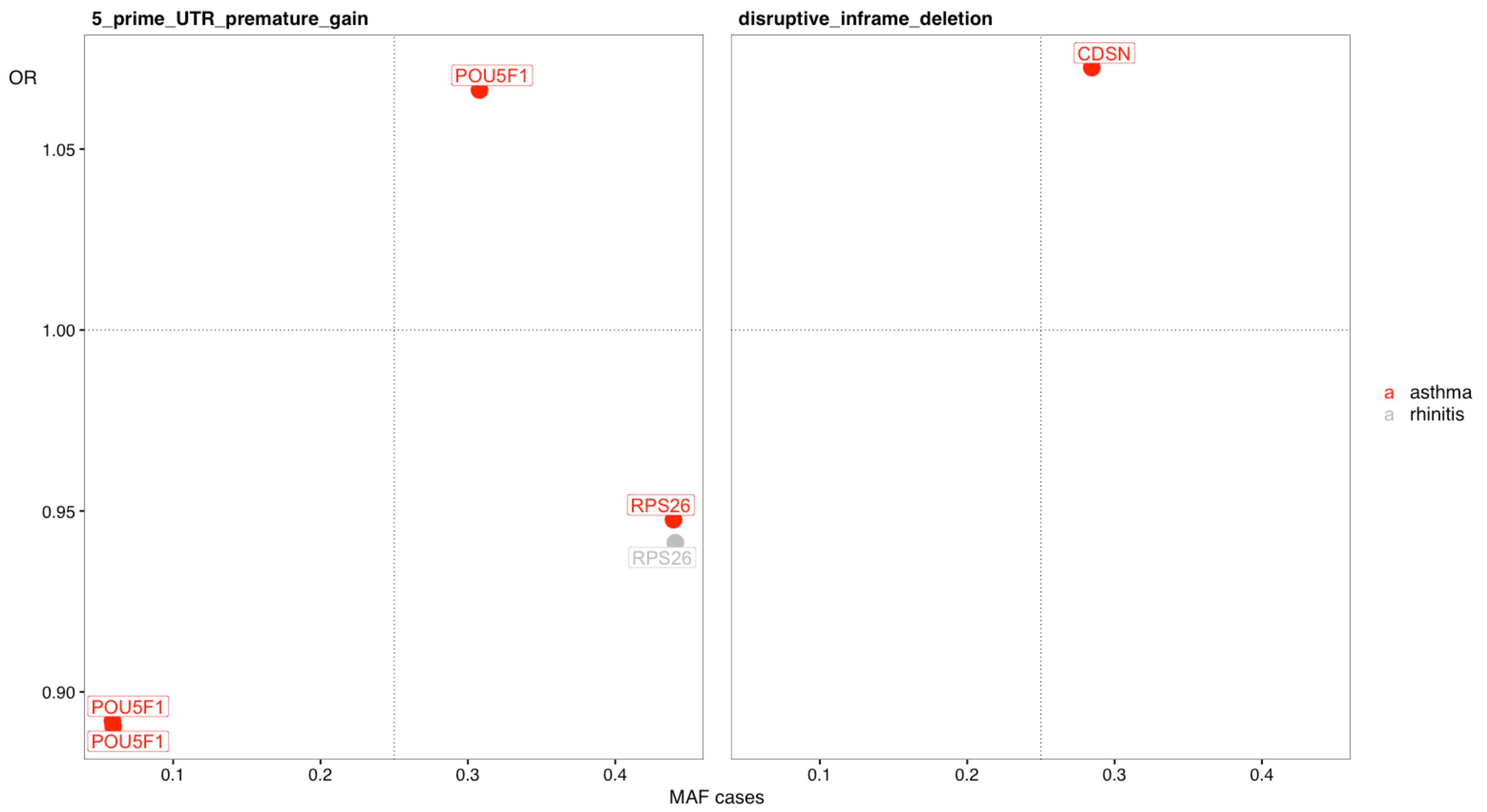

Fig S6 exome variants by functional consequence: minor allele frequency in cases versus odds ratio

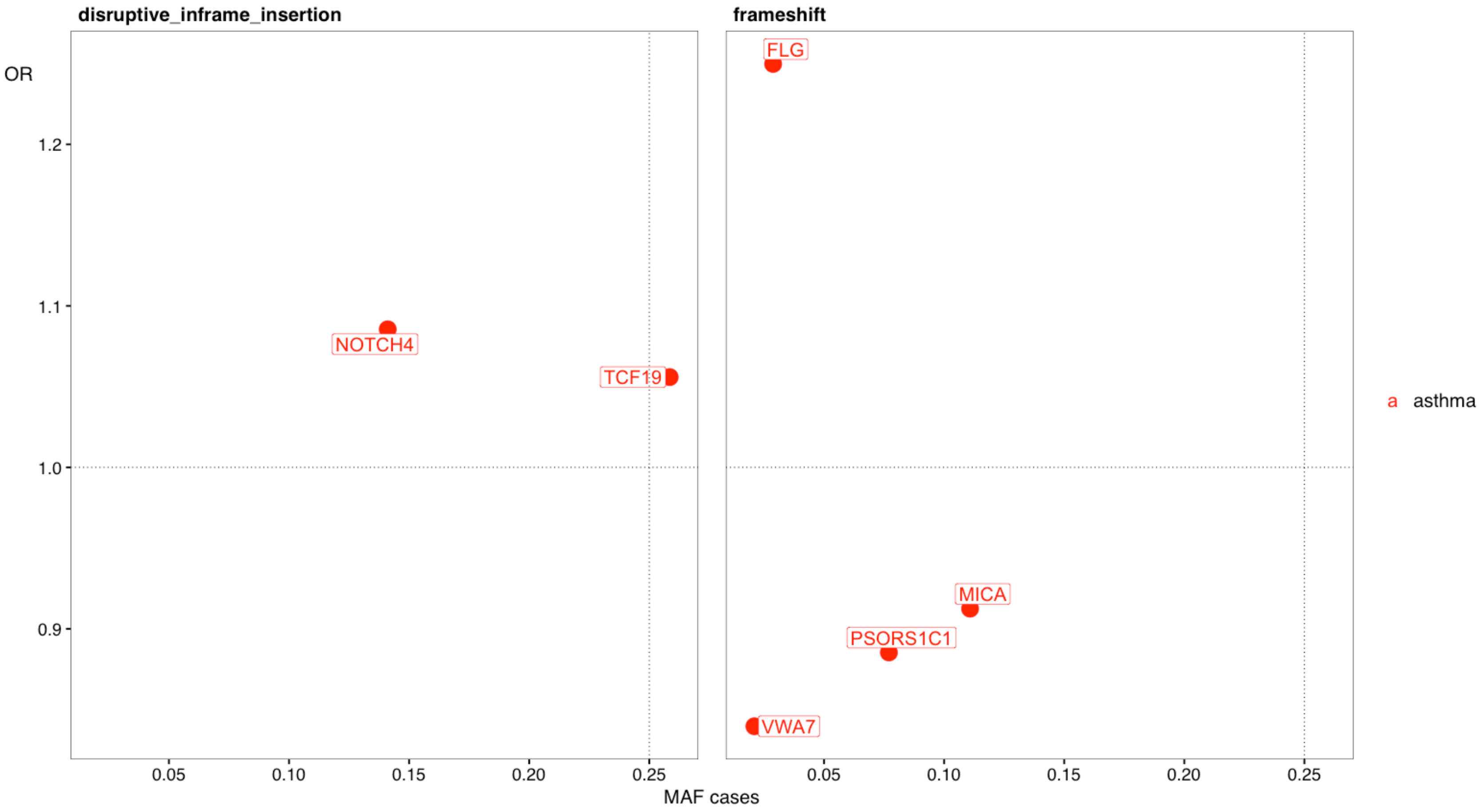

Fig S7 exome variants by functional consequence: minor allele frequency in cases versus odds ratio

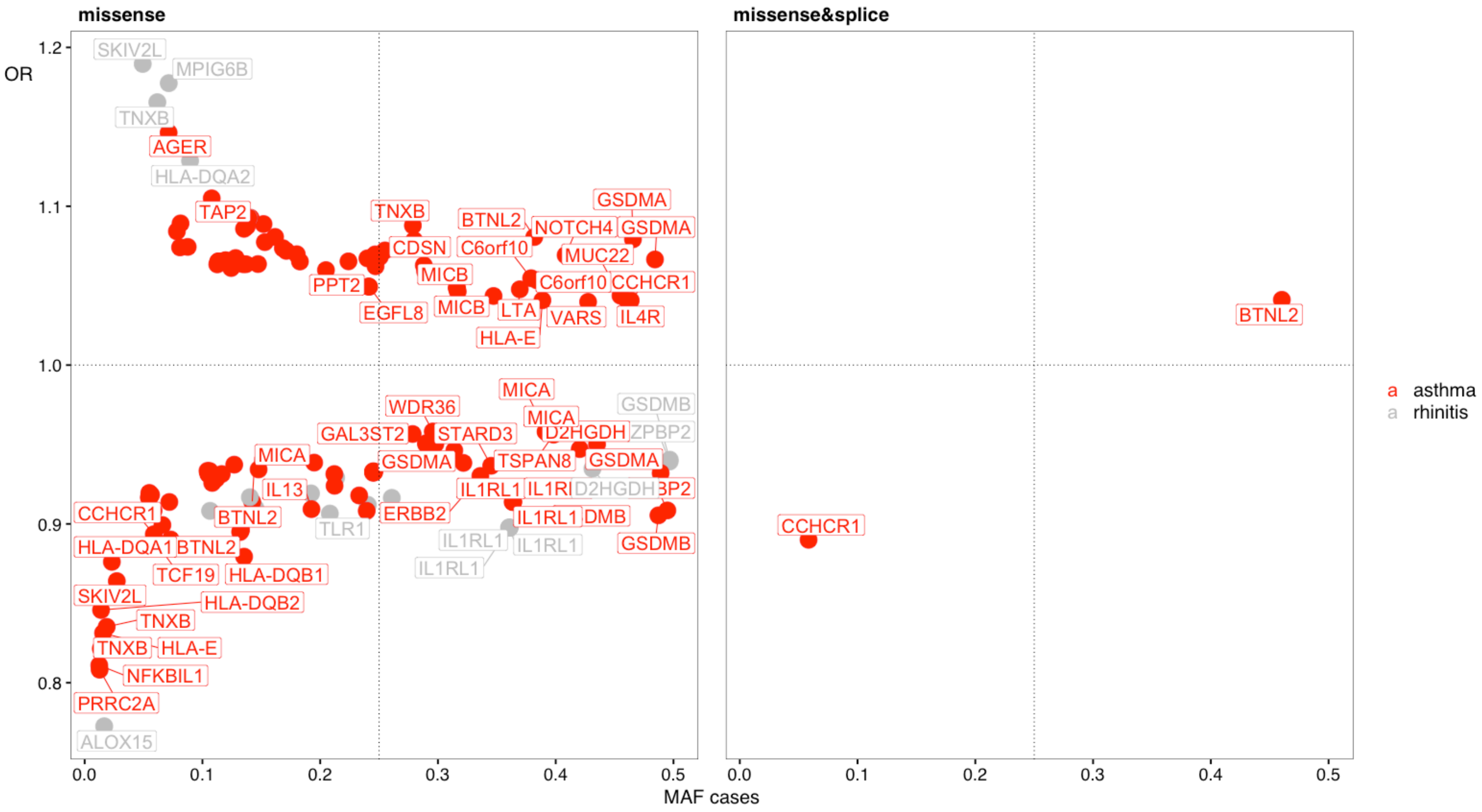

Fig S8 exome variants by functional consequence: minor allele frequency in cases versus odds ratio

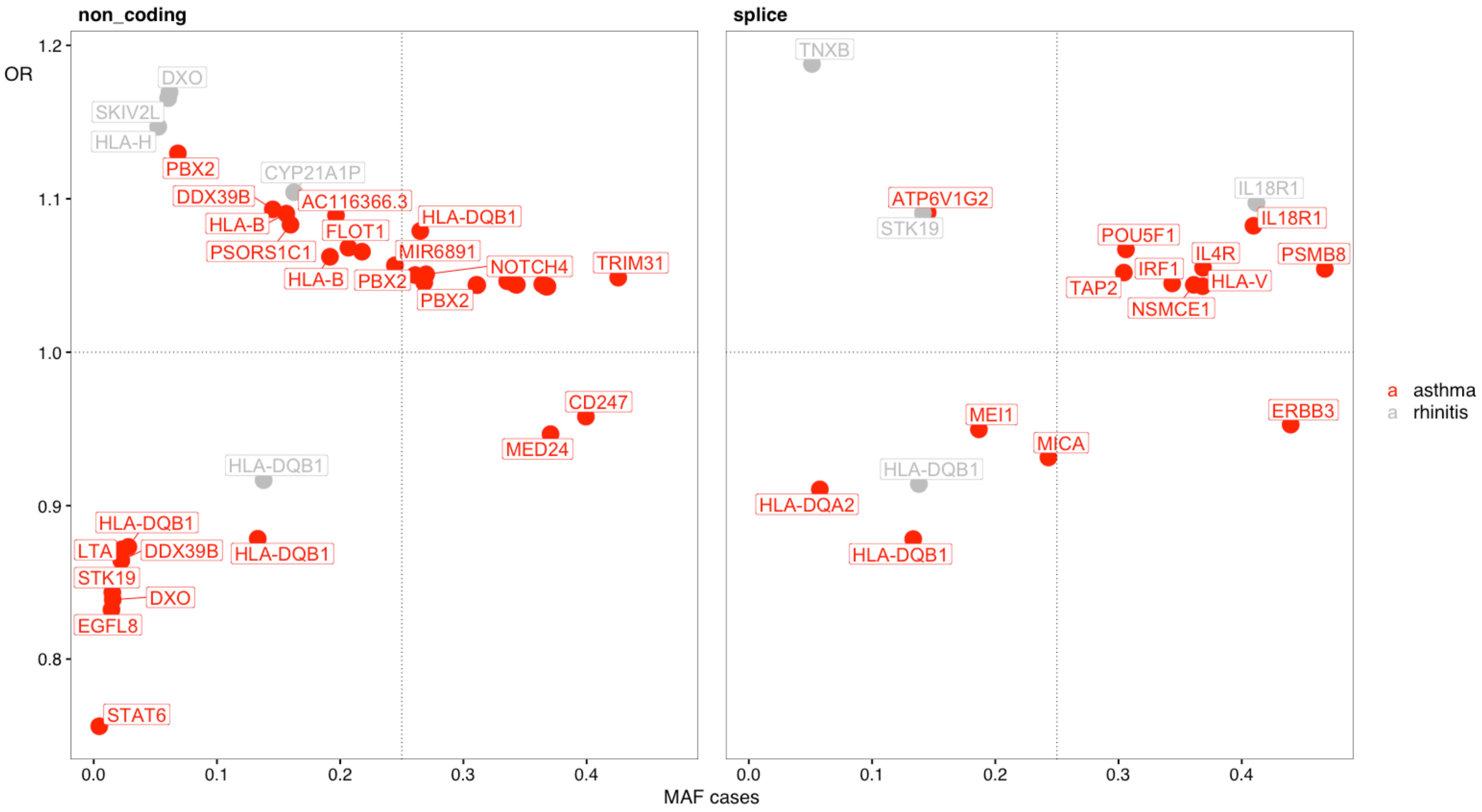

Fig S9 exome variants by functional consequence: minor allele frequency in cases versus odds ratio

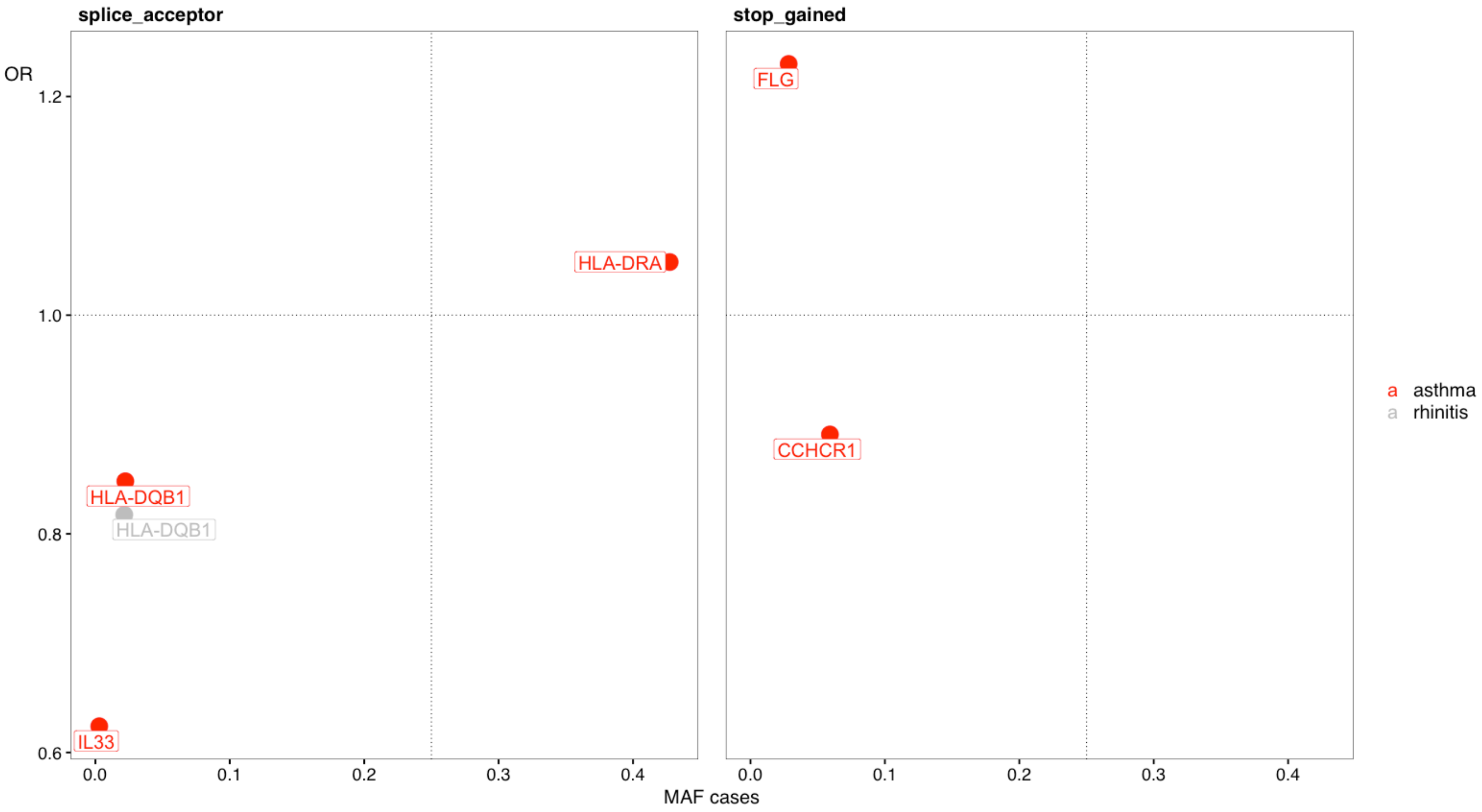

Fig S10 exome variants by functional consequence: minor allele frequency in cases versus odds ratio

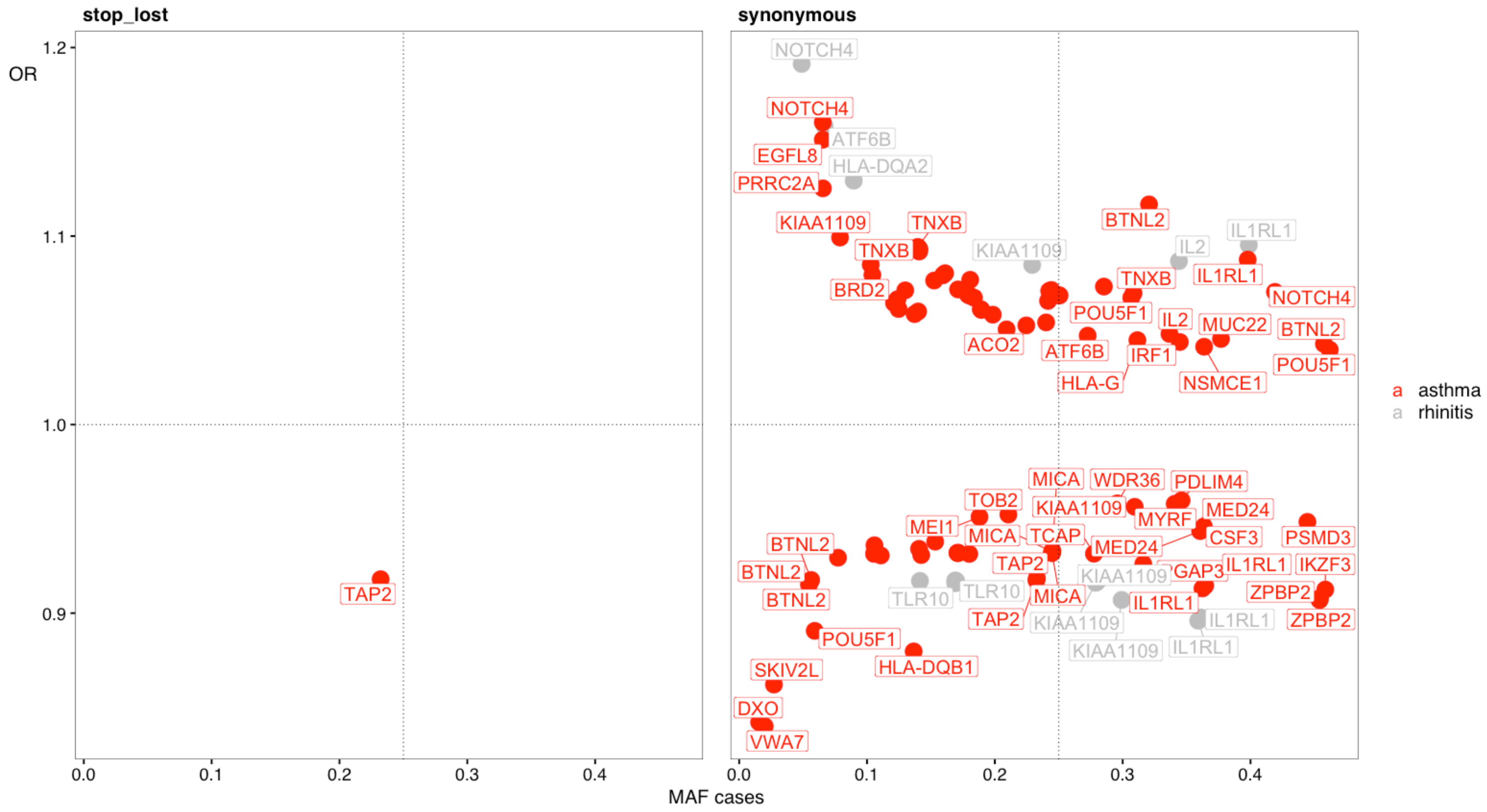

Fig S11 Reactome annotation of exome variants

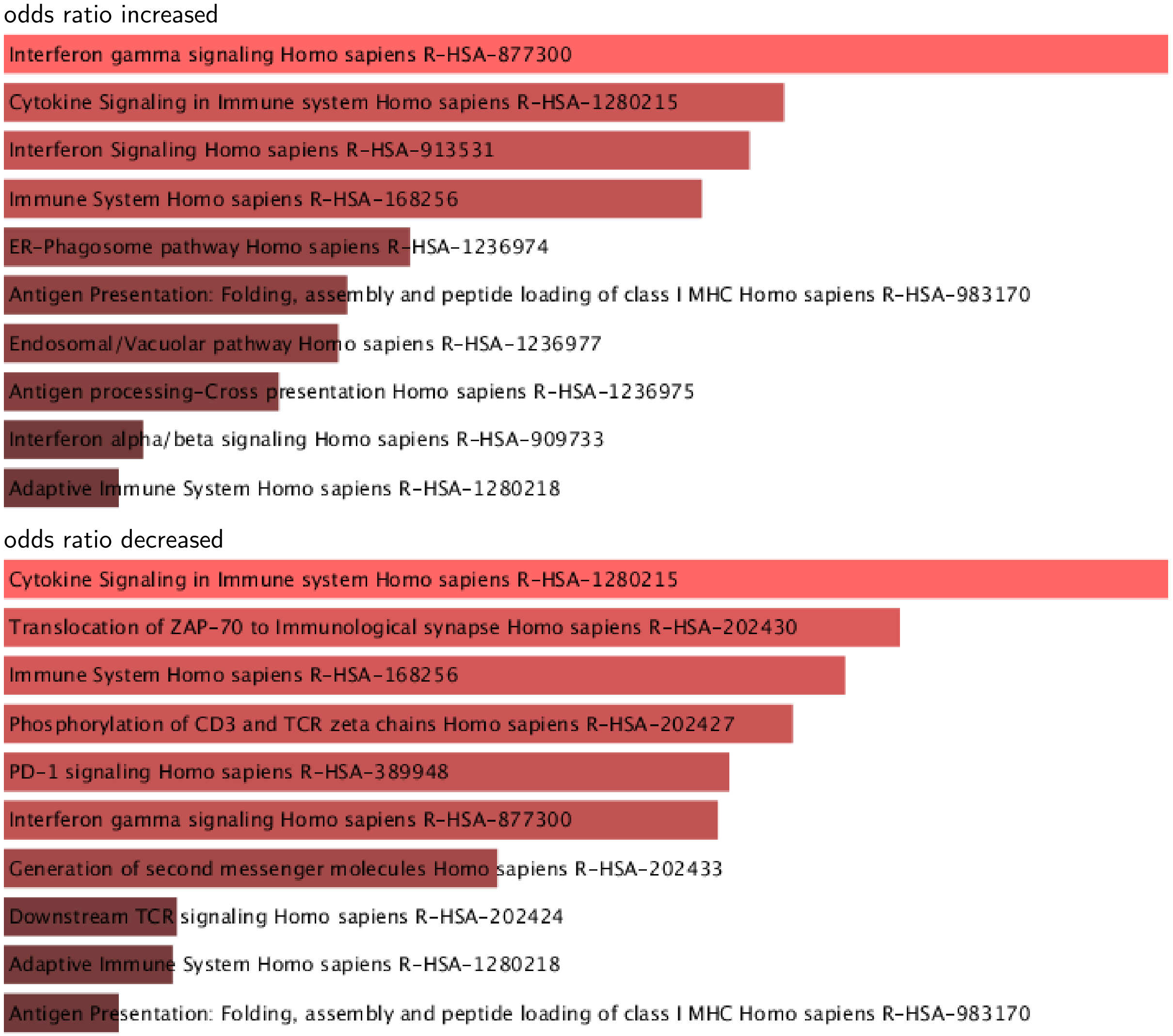

Fig S12 Human Gene Atlas annotation of exome variants

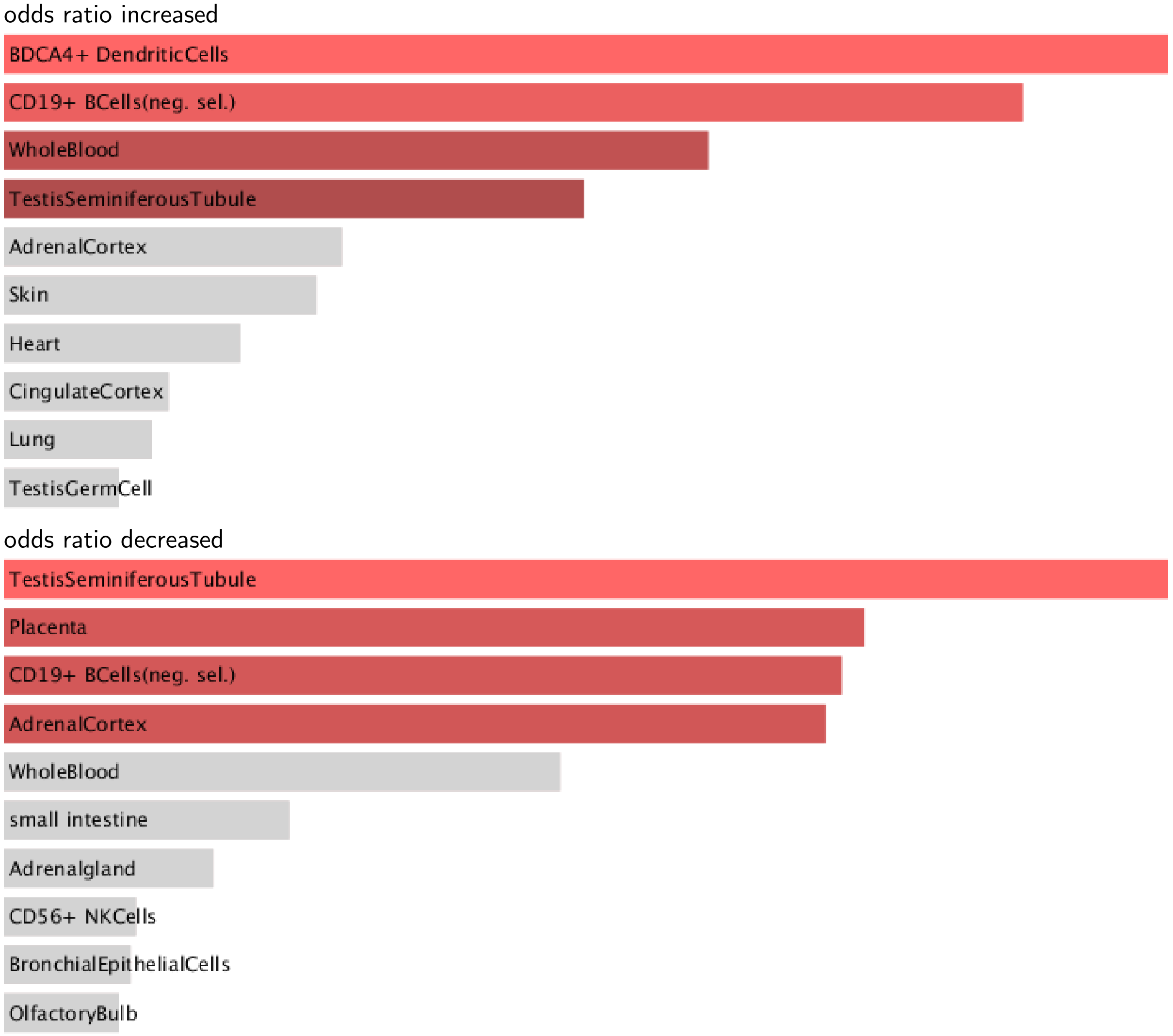

Table exome variants

- Sequence coordinates are using hg38 position
- Only results with P > -log10(8^10-7) are given for the exome analysis while all P > -log10(1^10-5´30) are truncated at this value
- Variants may appear twice with different traits

	chr	pos	Gene	cDNA	Consequence	Case.MAF	Control.MAF	Odds.ratio	p	trait
1	1	152312600	FLG	c.2282_22...	frameshift	0.0288	0.0232	1.2498	28.329940	asthma
2	1	152313385	FLG	c.1501C>T	stop_gained	0.0284	0.0232	1.2298	24.313006	asthma
3	1	167439433	CD247	n.208G>A	non_coding	0.3994	0.4097	0.9580	10.217958	asthma
4	2	102226007	IL1RL2	c.1101T>C	synonymous	0.1247	0.1183	1.0613	8.894149	asthma
5	2	102339008	IL1RL1	c.233C>A	missense	0.2135	0.2261	0.9291	9.175289	rhinitis
6	2	102339008	IL1RL1	c.233C>A	missense	0.2122	0.2257	0.9240	23.383419	asthma
7	2	102341256	IL1RL1	c.679C>T	synonymous	0.3987	0.3771	1.0953	18.591251	rhinitis
8	2	102341256	IL1RL1	c.679C>T	synonymous	0.3978	0.3779	1.0875	36.071450	asthma
9	2	102351547	IL1RL1	c.1297G>A	missense	0.3604	0.3856	0.8977	25.315963	rhinitis
10	2	102351547	IL1RL1	c.1297G>A	missense	0.3634	0.3845	0.9138	40.882397	asthma
11	2	102351615	IL1RL1	c.1365T>C	synonymous	0.3613	0.3866	0.8977	25.518844	rhinitis
12	2	102351615	IL1RL1	c.1365T>C	synonymous	0.3646	0.3855	0.9146	40.414652	asthma
13	2	102351751	IL1RL1	c.1501C>A	missense	0.3608	0.3861	0.8977	25.472112	rhinitis
14	2	102351751	IL1RL1	c.1501C>A	missense	0.3639	0.3850	0.9139	40.922996	asthma
15	2	102351752	IL1RL1	c.1502A>G	missense	0.3608	0.3860	0.8976	25.460422	rhinitis
16	2	102351752	IL1RL1	c.1502A>G	missense	0.3639	0.3849	0.9139	40.884057	asthma
17	2	102351825	IL1RL1	c.1575T>C	synonymous	0.3591	0.3848	0.8960	25.962574	rhinitis
18	2	102351825	IL1RL1	c.1575T>C	synonymous	0.3624	0.3836	0.9131	41.242680	asthma
19	2	102367819	IL18R1	c.59-6T>C	splice	0.4119	0.3896	1.0973	19.610834	rhinitis
20	2	102367819	IL18R1	c.59-6T>C	splice	0.4095	0.3905	1.0824	32.717831	asthma
21	2	241735388	D2HGDH	c.164G>A	missense	0.2978	0.3086	0.9503	12.546529	asthma
22	2	241748877	D2HGDH	c.427C>T	missense	0.4312	0.4478	0.9348	10.967784	rhinitis
23	2	241748877	D2HGDH	c.427C>T	missense	0.4351	0.4477	0.9501	14.562408	asthma
24	2	241748951	D2HGDH	c.*191C>T	3_prime_UTR	0.4307	0.4473	0.9345	11.034046	rhinitis
25	2	241748951	D2HGDH	c.*191C>T	3_prime_UTR	0.4347	0.4473	0.9503	14.415217	asthma
26	2	241749257	D2HGDH	c.*497C>T	3_prime_UTR	0.4296	0.4461	0.9350	10.585863	rhinitis
27	2	241749257	D2HGDH	c.*497C>T	3_prime_UTR	0.4336	0.4460	0.9507	13.782253	asthma
28	2	241751260	D2HGDH	c.1012G>A	missense	0.2404	0.2576	0.9120	15.237922	rhinitis

(continued)

	chr	pos	Gene	cDNA	Consequence	Case.MAF	Control.MAF	Odds.ratio	p	trait
29	2	241751260	D2HGDH	c.1012G>A	missense	0.2395	0.2575	0.9082	37.580209	asthma
30	2	241776965	GAL3ST2	c.10A>T	missense	0.2785	0.2875	0.9566	9.291070	asthma
31	4	38773164	TLR10	c.2427T>C	synonymous	0.1690	0.1815	0.9173	10.531800	rhinitis
32	4	38773164	TLR10	c.2427T>C	synonymous	0.1707	0.1809	0.9320	16.088310	asthma
33	4	38773268	TLR10	c.2323A>G	missense	0.1450	0.1571	0.9099	11.148680	rhinitis
34	4	38773268	TLR10	c.2323A>G	missense	0.1477	0.1565	0.9343	13.480960	asthma
35	4	38773419	TLR10	c.2172T>C	synonymous	0.1697	0.1822	0.9171	10.650334	rhinitis
36	4	38773419	TLR10	c.2172T>C	synonymous	0.1715	0.1817	0.9320	16.158766	asthma
37	4	38774486	TLR10	c.1105A>C	missense	0.2900	0.3005	0.9508	12.111596	asthma
38	4	38774559	TLR10	c.1032G>T	synonymous	0.1691	0.1818	0.9159	10.881405	rhinitis
39	4	38774559	TLR10	c.1032G>T	synonymous	0.1710	0.1812	0.9318	16.213036	asthma
40	4	38774870	TLR10	c.721A>C	missense	0.2899	0.3005	0.9502	12.386475	asthma
41	4	38797027	TLR1	c.1805G>T	missense	0.2082	0.2248	0.9065	15.234182	rhinitis
42	4	38797027	TLR1	c.1805G>T	missense	0.2120	0.2241	0.9314	18.666956	asthma
43	4	38798089	TLR1	c.743A>G	missense	0.1922	0.2057	0.9192	11.046289	rhinitis
44	4	38798089	TLR1	c.743A>G	missense	0.1949	0.2051	0.9386	14.453828	asthma
45	4	122224596	KIAA1109	c.2712T>A	synonymous	0.2789	0.2969	0.9161	15.100344	rhinitis
46	4	122271228	KIAA1109	c.7704T>C	synonymous	0.2803	0.2981	0.9169	14.858550	rhinitis
47	4	122307977	KIAA1109	c.9870C>T	synonymous	0.2992	0.3200	0.9070	19.252122	rhinitis
48	4	122307977	KIAA1109	c.9870C>T	synonymous	0.3094	0.3191	0.9564	9.935168	asthma
49	4	122355806	KIAA1109	c.14316G>A	synonymous	0.0789	0.0723	1.0992	14.310691	asthma
50	4	122359705	KIAA1109	c.14784T>C	synonymous	0.2292	0.2151	1.0847	11.274660	rhinitis
51	4	122359705	KIAA1109	c.14784T>C	synonymous	0.2246	0.2158	1.0526	10.449405	asthma
52	4	122456327	IL2	c.114G>T	synonymous	0.3440	0.3255	1.0867	14.795609	rhinitis
53	4	122456327	IL2	c.114G>T	synonymous	0.3366	0.3262	1.0481	11.165643	asthma
54	5	14610200	FAM105A	c.957C>G	missense	0.0810	0.0758	1.0741	8.735418	asthma
55	5	35955580	UGT3A1	c.*49G>C	3_prime_UTR	0.4268	0.4420	0.9400	8.867420	rhinitis
56	5	111103810	WDR36	c.790A>G	missense	0.2956	0.3045	0.9582	8.937794	asthma
57	5	111121006	WDR36	c.2181A>T	synonymous	0.2957	0.3047	0.9582	8.876475	asthma
58	5	132266473	PDLIM4	c.255T>C	synonymous	0.3461	0.3369	0.9598	8.787280	asthma
59	5	132340627	SLC22A4	c.1507C>T	missense	0.4204	0.4337	0.9471	16.210278	asthma
60	5	132484106	IRF1	n.4132A>G	non_coding	0.3355	0.3255	1.0463	10.325139	asthma
61	5	132484108	IRF1	n.4130C>T	non_coding	0.3402	0.3303	1.0453	10.005903	asthma

(continued)

	chr	pos	Gene	cDNA	Consequence	Case.MAF	Control.MAF	Odds.ratio	p	trait
62	5	132486174	IRF1	n.1180A>C	non_coding	0.3434	0.3337	1.0440	9.453951	asthma
63	5	132486363	IRF1	c.555A>G	synonymous	0.3447	0.3351	1.0438	9.574792	asthma
64	5	132486380	IRF1	c.545-7T>C	splice	0.3435	0.3337	1.0448	9.905529	asthma
65	5	132486441	AC116366.3	c.-388T>C	5_prime_UTR	0.3447	0.3349	1.0446	9.903438	asthma
66	5	132486532	AC116366.3	c.-297T>C	5_prime_UTR	0.3452	0.3354	1.0443	9.803548	asthma
67	5	132579521	AC116366.3	n.730G>A	non_coding	0.1966	0.1835	1.0892	24.424466	asthma
68	5	132660272	IL13	c.431A>G	missense	0.1926	0.1783	0.9093	30.164690	asthma
69	5	132706454	KIF3A	c.1234G>A	missense	0.1369	0.1298	1.0635	10.176070	asthma
70	5	157509356	ADAM19	c.850A>G	missense	0.3472	0.3376	1.0435	9.533429	asthma
71	6	26409662	BTN3A1	c.845G>C	missense	0.1126	0.1066	1.0634	8.729321	asthma
72	6	26463346	BTN2A1	c.533G>T	missense	0.1135	0.1073	1.0653	9.241845	asthma
73	6	26463347	BTN2A1	c.534G>T	missense	0.1135	0.1073	1.0651	9.187154	asthma
74	6	26463432	BTN2A1	c.619G>A	missense	0.1244	0.1181	1.0611	8.851397	asthma
75	6	27867440	HIST1H1B	c.90C>T	synonymous	0.1211	0.1146	1.0645	9.536406	asthma
76	6	27911422	OR2B2	c.898G>T	missense	0.1189	0.1124	1.0655	9.641494	asthma
77	6	27912204	OR2B2	c.116T>C	missense	0.1196	0.1130	1.0660	9.824488	asthma
78	6	28301047	PGBD1	c.1193A>G	missense	0.1206	0.1141	1.0649	9.610302	asthma
79	6	28398374	ZSCAN12	c.32T>C	missense	0.1199	0.1133	1.0657	9.775726	asthma
80	6	28575487	ZBED9	c.1218G>A	synonymous	0.2401	0.2306	1.0542	11.540155	asthma
81	6	28586766	ZBED9	c.-49C>A	5_prime_UTR	0.2393	0.2300	1.0535	11.044842	asthma
82	6	28923399	TRIM27	c.234A>G	synonymous	0.1238	0.1170	1.0665	10.261854	asthma
83	6	29374998	OR12D3	c.290C>T	missense	0.1282	0.1211	1.0674	10.803548	asthma
84	6	29556180	UBD	c.198T>C	synonymous	0.1299	0.1223	1.0712	12.052664	asthma
85	6	29792099	HCG4	n.652C>T	non_coding	0.3667	0.3570	1.0429	9.423428	asthma
86	6	29792108	HCG4	n.643C>T	non_coding	0.3106	0.3015	1.0439	9.119529	asthma
87	6	29792146	HCG4	n.605A>G	non_coding	0.3112	0.3021	1.0437	9.057298	asthma
88	6	29792219	HLA-V	n.102-6T>C	splice	0.3682	0.3585	1.0430	9.575282	asthma
89	6	29792334	HCG4	n.417C>G	non_coding	0.3681	0.3584	1.0427	9.450016	asthma
90	6	29792411	HCG4	n.340C>T	non_coding	0.3679	0.3581	1.0432	9.623423	asthma
91	6	29792575	HCG4	n.155_175...	non_coding	0.3640	0.3540	1.0443	9.799971	asthma
92	6	29829862	HLA-G	c.942C>T	synonymous	0.3115	0.3022	1.0448	9.488518	asthma
93	6	29888649	HLA-H	n.532C>G	non_coding	0.0523	0.0459	1.1469	8.792635	rhinitis
94	6	29942982	HLA-A	c.299T>C	missense	0.2880	0.2756	1.0628	16.091140	asthma
95	6	30064745	ZNRD1	c.*48A>C	3_prime_UTR	0.1370	0.1282	1.0790	14.865504	asthma

(continued)

	chr	pos	Gene	cDNA	Consequence	Case.MAF	Control.MAF	Odds.ratio	p	trait
96	6	30108978	TRIM31	n.361C>T	non_coding	0.4256	0.4140	1.0486	12.227165	asthma
97	6	30110498	TRIM31	c.694G>A	missense	0.1801	0.1704	1.0698	14.977572	asthma
98	6	30110553	TRIM31	c.639G>A	synonymous	0.1789	0.1693	1.0689	14.520713	asthma
99	6	30112497	TRIM31	c.309C>T	synonymous	0.1837	0.1742	1.0673	14.232918	asthma
100	6	30112719	TRIM31	c.87C>T	synonymous	0.1803	0.1707	1.0684	14.441291	asthma
101	6	30198489	TRIM26	c.474G>A	synonymous	0.2416	0.2301	1.0656	16.417482	asthma
102	6	30199109	TRIM26	c.-6G>A	5_prime_UTR	0.1697	0.1606	1.0684	13.735418	asthma
103	6	30490287	HLA-E	c.382G>A	missense	0.3887	0.3983	1.0408	8.931072	asthma
104	6	30491388	HLA-E	c.862C>T	missense	0.0156	0.0187	0.8313	12.765736	asthma
105	6	30547266	GNL1	c.1287A>G	synonymous	0.1525	0.1433	1.0764	15.488651	asthma
106	6	30643573	ATAT1	c.*558C>A	3_prime_UTR	0.1528	0.1434	1.0776	15.999132	asthma
107	6	30685004	PPP1R18	c.1015G>A	missense	0.1530	0.1436	1.0774	15.924453	asthma
108	6	30730764	FLOT1	n.761T>C	non_coding	0.2067	0.1961	1.0681	15.815025	asthma
109	6	30744028	IER3	c.379G>C	missense	0.0875	0.0934	1.0744	9.778847	asthma
110	6	30951614	DPCR1	c.3150G>A	synonymous	0.1712	0.1616	1.0717	15.119587	asthma
111	6	30952347	DPCR1	c.1255G>A	missense	0.1712	0.1616	1.0719	15.201626	asthma
112	6	31025536	MUC22	c.105C>T	synonymous	0.1895	0.1806	1.0608	12.105130	asthma
113	6	31025722	MUC22	c.291C>T	synonymous	0.3769	0.3874	1.0454	10.715344	asthma
114	6	31025756	MUC22	c.325A>G	missense	0.4550	0.4656	1.0437	10.460548	asthma
115	6	31026089	MUC22	c.658A>T	missense	0.1164	0.1239	0.9314	12.037157	asthma
116	6	31026103	MUC22	c.672G>C	missense	0.1270	0.1343	0.9373	10.905529	asthma
117	6	31027401	MUC22	c.1970C>T	missense	0.1051	0.1120	0.9313	11.200935	asthma
118	6	31027410	MUC22	c.1979T>C	missense	0.1052	0.1120	0.9322	10.926282	asthma
119	6	31027520	MUC22	c.2089A>G	missense	0.1052	0.1121	0.9316	11.100234	asthma
120	6	31027755	MUC22	c.2324T>C	missense	0.1061	0.1128	0.9333	10.650917	asthma
121	6	31028355	MUC22	c.2924C>T	missense	0.1061	0.1130	0.9322	11.015653	asthma
122	6	31028713	MUC22	c.3282C>T	synonymous	0.1055	0.1124	0.9314	11.205303	asthma
123	6	31028732	MUC22	c.3301G>A	missense	0.1043	0.1109	0.9334	10.453087	asthma
124	6	31029301	MUC22	c.3870T>C	synonymous	0.1108	0.1181	0.9306	11.970616	asthma
125	6	31029557	MUC22	c.4126A>G	missense	0.1051	0.1119	0.9321	10.946537	asthma
126	6	31029575	MUC22	c.4144A>G	missense	0.1101	0.1175	0.9288	12.462433	asthma
127	6	31030039	MUC22	c.4608G>T	synonymous	0.1533	0.1618	0.9379	12.389021	asthma
128	6	31030047	MUC22	c.4616T>C	missense	0.1116	0.1192	0.9283	12.778325	asthma

(continued)

	chr	pos	Gene	cDNA	Consequence	Case.MAF	Control.MAF	Odds.ratio	p	trait
129	6	31032362	MUC22	c.4836C>T	synonymous	0.1060	0.1128	0.9322	11.004233	asthma
130	6	31111867	C6orf15	c.492C>T	synonymous	0.1611	0.1510	1.0803	17.706858	asthma
131	6	31112217	C6orf15	c.142G>A	missense	0.0660	0.0728	0.8993	16.203842	asthma
132	6	31112239	C6orf15	c.120G>C	missense	0.2049	0.1956	1.0598	12.408601	asthma
133	6	31116036	CDSN	c.1579A>G	missense	0.2467	0.2594	1.0697	18.771600	asthma
134	6	31116271	CDSN	c.1344T>C	synonymous	0.2853	0.2711	1.0732	22.340369	asthma
135	6	31117165	CDSN	c.447_449...	disruptiv...	0.2845	0.2704	1.0725	21.808270	asthma
136	6	31117187	CDSN	c.428A>G	missense	0.2506	0.2631	1.0681	18.456801	asthma
137	6	31117423	CDSN	c.192T>C	synonymous	0.2506	0.2632	1.0685	18.621602	asthma
138	6	31117492	CDSN	c.123T>C	synonymous	0.2501	0.2628	1.0686	18.615109	asthma
139	6	31117579	PSORS1C1	n.2586C>T	non_coding	0.1597	0.1492	1.0831	17.635637	asthma
140	6	31120368	CDSN	c.52A>T	missense	0.2798	0.2648	1.0786	25.097780	asthma
141	6	31138682	PSORS1C1	c.70C>A	missense	0.0730	0.0813	0.8902	21.071963	asthma
142	6	31138723	PSORS1C1	c.118dupC	frameshift	0.0770	0.0862	0.8854	24.149721	asthma
143	6	31144707	CCHCR1	c.1880G>A	missense	0.0587	0.0653	0.8935	16.391902	asthma
144	6	31151121	CCHCR1	c.536T>A	missense&...	0.0584	0.0652	0.8900	17.439735	asthma
145	6	31154538	CCHCR1	c.492C>G	missense	0.4637	0.4538	1.0407	9.235973	asthma
146	6	31157480	CCHCR1	c.121G>T	stop_gained	0.0591	0.0658	0.8909	17.289883	asthma
147	6	31157625	CCHCR1	c.-25G>C	5_prime_UTR	0.0590	0.0657	0.8914	17.075359	asthma
148	6	31161533	TCF19	c.325C>T	missense	0.1617	0.1515	1.0807	17.964971	asthma
149	6	31161930	TCF19	c.722C>T	missense	0.0591	0.0659	0.8899	17.628563	asthma
150	6	31164637	POU5F1	c.1047C>T	synonymous	0.3067	0.3208	1.0673	20.194771	asthma
151	6	31164872	POU5F1	c.817-5T>G	splice	0.3060	0.3199	1.0670	19.939302	asthma
152	6	31165179	POU5F1	c.765C>G	synonymous	0.0591	0.0659	0.8906	17.445753	asthma
153	6	31165732	POU5F1	c.-93C>T	5_prime_UTR	0.3058	0.3196	1.0665	19.635261	asthma
154	6	31165886	POU5F1	c.-247A>G	5_prime_U...	0.0588	0.0655	0.8920	16.867740	asthma
155	6	31166093	POU5F1	c.-229C>T	5_prime_U...	0.0593	0.0661	0.8904	17.539403	asthma
156	6	31166166	POU5F1	c.-302G>T	5_prime_U...	0.3079	0.3217	1.0663	19.779892	asthma
157	6	31166341	TCF19	c.620_622...	disruptiv...	0.2585	0.2691	1.0559	12.846795	asthma
158	6	31170600	POU5F1	c.21G>A	synonymous	0.4620	0.4523	1.0397	8.832092	asthma
159	6	31271830	HLA-C	c.112C>T	missense	0.0719	0.0781	0.9138	11.616723	asthma
160	6	31355235	MIR6891	n.82C>T	non_coding	0.2444	0.2344	1.0566	11.714443	asthma
161	6	31355544	HLA-B	c.668C>T	missense	0.1683	0.1586	1.0736	15.059932	asthma

(continued)

	chr	pos	Gene	cDNA	Consequence	Case.MAF	Control.MAF	Odds.ratio	p	trait
162	6	31355560	HLA-B	c.652A>G	missense	0.1065	0.1160	0.9082	8.977984	rhinitis
163	6	31355560	HLA-B	c.652A>G	missense	0.1083	0.1160	0.9258	13.255551	asthma
164	6	31355632	HLA-B	n.171A>G	non_coding	0.1917	0.2012	1.0622	12.863279	asthma
165	6	31355639	HLA-B	n.164C>G	non_coding	0.1563	0.1452	1.0903	21.318397	asthma
166	6	31356638	HLA-B	n.414T>G	non_coding	0.2177	0.2071	1.0655	13.804100	asthma
167	6	31403653	MICA	c.21T>C	synonymous	0.1984	0.2076	1.0583	11.872571	asthma
168	6	31410581	MICA	c.70T>G	missense	0.1423	0.1537	0.9138	22.540909	asthma
169	6	31410610	MICA	c.99T>C	synonymous	0.2450	0.2581	0.9330	20.027334	asthma
170	6	31410648	MICA	c.137A>G	missense	0.2449	0.2579	0.9331	19.970616	asthma
171	6	31411200	MICA	c.454G>A	missense	0.2458	0.2590	0.9325	20.425621	asthma
172	6	31411332	MICA	c.586G>A	missense	0.2457	0.2588	0.9330	20.119587	asthma
173	6	31411996	MICA	c.336C>T	synonymous	0.2447	0.2580	0.9318	20.713993	asthma
174	6	31412017	MICA	c.357C>T	synonymous	0.2449	0.2581	0.9325	20.322941	asthma
175	6	31412018	MICA	c.358A>G	missense	0.2449	0.2580	0.9326	20.234481	asthma
176	6	31412030	MICA	c.370C>T	missense	0.2450	0.2582	0.9322	20.493766	asthma
177	6	31412040	MICA	c.380T>C	missense	0.3914	0.4017	0.9581	10.138764	asthma
178	6	31412046	MICA	c.386C>G	missense	0.2455	0.2587	0.9326	20.338187	asthma
179	6	31412154	MICA	c.494G>A	missense	0.3917	0.4020	0.9580	10.205721	asthma
180	6	31412314	MICA	c.566-4dupT	splice	0.2431	0.2565	0.9314	20.759201	asthma
181	6	31412384	MICA	c.625_626...	frameshift	0.1108	0.1201	0.9125	17.718058	asthma
182	6	31505769	MICB	c.223A>G	missense	0.2240	0.2352	1.0653	15.904831	asthma
183	6	31505784	MICB	c.238A>G	missense	0.3160	0.3058	1.0486	11.163802	asthma
184	6	31506180	MICB	c.363C>G	missense	0.1519	0.1413	1.0888	20.307153	asthma
185	6	31506223	MICB	c.406G>A	missense	0.3161	0.3060	1.0485	11.122859	asthma
186	6	31509904	MICB	c.1147A>G	missense	0.2550	0.2685	1.0722	20.655215	asthma
187	6	31529929	MCCD1	c.354C>T	synonymous	0.2442	0.2572	1.0714	19.767512	asthma
188	6	31530467	DDX39B	n.393dupG	non_coding	0.1453	0.1346	1.0931	21.230401	asthma
189	6	31531452	DDX39B	n.293G>A	non_coding	0.0224	0.0258	0.8642	10.656001	asthma
190	6	31538871	DDX39B	c.408C>A	synonymous	0.2425	0.2554	1.0709	19.268170	asthma
191	6	31546470	ATP6V1G2	c.82+8T>C	splice	0.1451	0.1347	1.0911	20.346498	asthma
192	6	31558135	NFKBIL1	c.625C>T	missense	0.0814	0.0880	1.0892	12.823330	asthma
193	6	31558303	NFKBIL1	c.793G>A	missense	0.0124	0.0152	0.8110	13.204398	asthma
194	6	31572916	LTA	n.336G>C	non_coding	0.0227	0.0260	0.8715	10.221415	asthma
195	6	31573007	LTA	c.179C>A	missense	0.3695	0.3587	1.0477	11.518414	asthma

(continued)

	chr	pos	Gene	cDNA	Consequence	Case.MAF	Control.MAF	Odds.ratio	p	trait
196	6	31616064	AIF1	c.-48C>G	5_prime_UTR	0.0662	0.0591	1.1298	19.730954	asthma
197	6	31626851	PRRC2A	c.1062T>A	synonymous	0.1400	0.1472	1.0600	9.673255	asthma
198	6	31632329	PRRC2A	c.3656C>A	missense	0.0127	0.0157	0.8082	13.989700	asthma
199	6	31636267	PRRC2A	c.5683T>G	missense	0.0783	0.0844	1.0842	11.143997	asthma
200	6	31636578	PRRC2A	c.5904C>A	synonymous	0.0655	0.0587	1.1253	18.390192	asthma
201	6	31719231	LY6G6C	c.243C>T	synonymous	0.1800	0.1907	0.9314	17.031144	asthma
202	6	31724609	MPIG6B	c.432C>G	missense	0.0714	0.0613	1.1775	16.215240	rhinitis
203	6	31760120	MSH5	c.1716C>T	synonymous	0.1408	0.1305	1.0918	20.318849	asthma
204	6	31765689	VWA7	c.2581A>G	missense	0.1409	0.1305	1.0927	20.718285	asthma
205	6	31765982	VWA7	c.2400C>T	synonymous	0.0200	0.0237	0.8400	14.388170	asthma
206	6	31766568	VWA7	c.2079C>A	synonymous	0.1410	0.1306	1.0931	20.868061	asthma
207	6	31772986	VWA7	c.1041_10...	frameshift	0.0210	0.0249	0.8397	15.107516	asthma
208	6	31781043	VARS	c.2625C>T	synonymous	0.1424	0.1513	0.9308	14.505845	asthma
209	6	31795066	VARS	c.152C>G	missense	0.4275	0.4179	1.0398	8.703774	asthma
210	6	31810169	HSPA1L	c.1804G>A	missense	0.3168	0.3071	1.0463	10.223371	asthma
211	6	31810495	HSPA1L	c.1478C>T	missense	0.1828	0.1924	1.0652	13.761452	asthma
212	6	31862816	NEU1	c.-40T>G	5_prime_UTR	0.0265	0.0307	0.8570	14.671213	asthma
213	6	31947158	CFB	c.450A>G	synonymous	0.1890	0.1983	1.0612	12.534023	asthma
214	6	31962574	SKIV2L	c.1200A>G	synonymous	0.0272	0.0315	0.8621	13.994391	asthma
215	6	31967790	SKIV2L	c.2659G>A	missense	0.0273	0.0314	0.8640	13.586030	asthma
216	6	31967973	SKIV2L	c.2749G>A	missense	0.0493	0.0418	1.1896	13.060281	rhinitis
217	6	31968902	SKIV2L	c.3212C>T	missense	0.1394	0.1291	1.0927	9.190912	rhinitis
218	6	31969260	SKIV2L	n.586C>T	non_coding	0.0604	0.0523	1.1656	12.406050	rhinitis
219	6	31970240	DXO	n.1683delC	non_coding	0.0154	0.0183	0.8387	11.487983	asthma
220	6	31970858	DXO	n.1066C>G	non_coding	0.0615	0.0530	1.1695	13.138227	rhinitis
221	6	31971568	DXO	c.108C>T	synonymous	0.0156	0.0184	0.8421	11.094798	asthma
222	6	31980623	STK19	n.233T>G	non_coding	0.0151	0.0178	0.8434	10.571217	asthma
223	6	31980644	STK19	c.902-6_9...	splice	0.1413	0.1311	1.0907	8.914709	rhinitis
224	6	32007072	CYP21A1P	n.1141C>G	non_coding	0.1626	0.1495	1.1043	12.240106	rhinitis
225	6	32053637	TNXB	c.8542G>A	missense	0.0187	0.0223	0.8352	14.397831	asthma
226	6	32056126	TNXB	c.8192C>G	missense	0.0617	0.0534	1.1655	12.660747	rhinitis
227	6	32056618	TNXB	c.8111G>A	missense	0.0549	0.0594	0.9195	8.818442	asthma
228	6	32058086	TNXB	c.7797G>A	synonymous	0.1807	0.1700	1.0768	17.846185	asthma

(continued)

	chr	pos	Gene	cDNA	Consequence	Case.MAF	Control.MAF	Odds.ratio	p	trait
229	6	32058330	TNXB	c.7553G>A	missense	0.2787	0.2959	1.0879	31.731422	asthma
230	6	32061428	TNXB	c.7461C>T	synonymous	0.1394	0.1290	1.0940	21.052370	asthma
231	6	32061449	TNXB	c.7440T>C	synonymous	0.3085	0.3230	1.0697	21.753994	asthma
232	6	32061654	TNXB	c.7235C>T	missense	0.0545	0.0591	0.9167	9.365825	asthma
233	6	32062152	TNXB	c.7168+5G>A	splice	0.0512	0.0435	1.1879	13.288868	rhinitis
234	6	32067917	TNXB	c.6288G>A	synonymous	0.1398	0.1293	1.0942	21.173472	asthma
235	6	32084667	TNXB	c.3191G>A	missense	0.0137	0.0166	0.8215	12.810790	asthma
236	6	32096949	TNXB	c.904A>G	missense	0.1392	0.1289	1.0926	20.472241	asthma
237	6	32121077	ATF6B	c.603C>G	synonymous	0.2726	0.2635	1.0472	9.734946	asthma
238	6	32126145	ATF6B	c.441C>T	synonymous	0.0668	0.0582	1.1584	12.605198	rhinitis
239	6	32128224	ATF6B	c.-17G>T	5_prime_UTR	0.1384	0.1283	1.0915	19.875496	asthma
240	6	32154609	PPT2	c.15C>G	missense	0.1344	0.1418	1.0635	10.386158	asthma
241	6	32154695	PPT2	c.101C>A	missense	0.2411	0.2324	1.0495	9.823041	asthma
242	6	32166733	EGFL8	c.257G>A	missense	0.2416	0.2329	1.0493	9.787812	asthma
243	6	32167051	EGFL8	n.415C>T	non_coding	0.0144	0.0172	0.8322	11.739452	asthma
244	6	32167360	EGFL8	c.612G>A	synonymous	0.0654	0.0573	1.1511	25.538952	asthma
245	6	32183666	AGER	c.244G>A	missense	0.0713	0.0628	1.1463	26.071963	asthma
246	6	32184217	AGER	c.6T>A	synonymous	0.1372	0.1440	1.0586	9.108351	asthma
247	6	32186508	PBX2	n.668T>C	non_coding	0.2682	0.2596	1.0454	8.851397	asthma
248	6	32187221	PBX2	n.1072G>A	non_coding	0.2608	0.2514	1.0503	10.616005	asthma
249	6	32188712	PBX2	n.105C>G	non_coding	0.0683	0.0609	1.1296	20.176983	asthma
250	6	32200994	NOTCH4	c.4152C>A	synonymous	0.1594	0.1494	1.0792	17.138227	asthma
251	6	32201155	NOTCH4	c.4101G>A	synonymous	0.0489	0.0414	1.1913	13.177832	rhinitis
252	6	32201368	NOTCH4	c.3888C>T	synonymous	0.0654	0.0568	1.1603	28.322119	asthma
253	6	32216928	NOTCH4	n.2007C>T	non_coding	0.2697	0.2796	1.0508	11.177244	asthma
254	6	32222613	NOTCH4	c.349A>C	missense	0.4084	0.3924	1.0692	23.800519	asthma
255	6	32222629	NOTCH4	c.333T>C	synonymous	0.4191	0.4027	1.0704	24.664542	asthma
256	6	32222707	NOTCH4	c.255C>T	synonymous	0.0773	0.0827	0.9294	9.150765	asthma
257	6	32223881	NOTCH4	c.42_47du...	disruptiv...	0.1411	0.1315	1.0855	17.780154	asthma
258	6	32293475	C6orf10	c.1192A>C	missense	0.1377	0.1281	1.0873	18.200797	asthma
259	6	32293994	C6orf10	c.673T>C	missense	0.3791	0.3667	1.0547	15.006740	asthma
260	6	32335915	C6orf10	c.442A>T	missense	0.1353	0.1259	1.0858	17.257589	asthma
261	6	32366178	C6orf10	c.206A>G	missense	0.3842	0.3720	1.0533	14.240861	asthma

(continued)

	chr	pos	Gene	cDNA	Consequence	Case.MAF	Control.MAF	Odds.ratio	p	trait
262	6	32369909	C6orf10	c.88T>C	missense	0.2608	0.2443	0.9164	13.741123	rhinitis
263	6	32371721	C6orf10	c.-15G>C	5_prime_UTR	0.0171	0.0203	0.8363	13.040577	asthma
264	6	32394964	BTNL2	c.1140G>A	missense	0.1320	0.1452	0.8949	31.664943	asthma
265	6	32394968	BTNL2	c.1136C>T	missense	0.1328	0.1459	0.8962	31.070785	asthma
266	6	32396039	BTNL2	c.1078A>G	missense&...	0.4603	0.4503	1.0411	9.167810	asthma
267	6	32396067	BTNL2	c.1050G>A	synonymous	0.3206	0.2971	1.1168	50.000000	asthma
268	6	32396178	BTNL2	c.939A>G	synonymous	0.4574	0.4470	1.0428	10.087406	asthma
269	6	32403017	BTNL2	c.627G>A	synonymous	0.0556	0.0603	0.9170	9.455932	asthma
270	6	32403039	BTNL2	c.605C>T	missense	0.0585	0.0640	0.9083	12.025396	asthma
271	6	32403058	BTNL2	c.586A>G	missense	0.3818	0.3636	1.0806	30.815025	asthma
272	6	32403102	BTNL2	c.542G>A	missense	0.0549	0.0596	0.9165	9.439137	asthma
273	6	32403131	BTNL2	c.513A>T	synonymous	0.0546	0.0594	0.9151	9.702896	asthma
274	6	32405086	BTNL2	c.280T>A	missense	0.0564	0.0611	0.9183	9.300596	asthma
275	6	32405186	BTNL2	c.180C>T	synonymous	0.0563	0.0611	0.9176	9.433327	asthma
276	6	32439932	HLA-DRA	c.-19C>A	5_prime_UTR	0.3803	0.3623	1.0801	30.184157	asthma
277	6	32443258	HLA-DRA	c.329-2A>C	splice_ac...	0.4273	0.4389	1.0486	12.536704	asthma
278	6	32637532	HLA-DQA1	c.74A>G	missense	0.0231	0.0263	0.8761	8.833570	asthma
279	6	32642029	HLA-DQA1	c.389C>T	missense	0.2401	0.2285	1.0672	16.707966	asthma
280	6	32642175	HLA-DQA1	c.535T>C	missense	0.2470	0.2359	1.0622	14.633390	asthma
281	6	32660883	HLA-DQB1	c.773-1A>G	splice_ac...	0.0215	0.0262	0.8174	9.050074	rhinitis
282	6	32660883	HLA-DQB1	c.773-1A>G	splice_ac...	0.0223	0.0262	0.8481	14.133594	asthma
283	6	32661352	HLA-DQB1	c.767A>G	missense	0.1403	0.1511	0.9168	9.208730	rhinitis
284	6	32661352	HLA-DQB1	c.767A>G	missense	0.1354	0.1512	0.8794	43.232177	asthma
285	6	32661378	HLA-DQB1	c.741G>T	synonymous	0.1414	0.1522	0.9171	9.235450	rhinitis
286	6	32661378	HLA-DQB1	c.741G>T	synonymous	0.1365	0.1523	0.8797	43.459420	asthma
287	6	32661480	HLA-DQB1	n.3728A>C	non_coding	0.0281	0.0321	0.8731	11.988853	asthma
288	6	32661939	HLA-DQB1	n.3269A>G	non_coding	0.2650	0.2505	1.0790	23.858864	asthma
289	6	32661941	HLA-DQB1	n.3267C>T	non_coding	0.1379	0.1486	0.9166	8.982549	rhinitis
290	6	32661941	HLA-DQB1	n.3267C>T	non_coding	0.1331	0.1487	0.8784	42.583859	asthma
291	6	32661960	HLA-DQB1	c.661+7G>A	splice	0.1380	0.1490	0.9141	9.600153	rhinitis
292	6	32661960	HLA-DQB1	c.661+7G>A	splice	0.1334	0.1491	0.8782	43.232251	asthma
293	6	32746077	HLA-DQA2	c.613+5G>A	splice	0.0576	0.0629	0.9106	11.313185	asthma
294	6	32746306	HLA-DQA2	c.680T>C	missense	0.0895	0.0801	1.1286	11.319665	rhinitis
295	6	32746391	HLA-DQA2	c.765A>G	synonymous	0.0896	0.0801	1.1294	11.451365	rhinitis

(continued)

	chr	pos	Gene	cDNA	Consequence	Case.MAF	Control.MAF	Odds.ratio	p	trait
296	6	32758976	HLA-DQB2	c.520C>T	missense	0.0139	0.0164	0.8458	9.500725	asthma
297	6	32813180	HLA-DOB	c.*36G>A	3_prime_UTR	0.0674	0.0585	1.1637	29.909037	asthma
298	6	32828876	TAP2	c.2088G>T	synonymous	0.2325	0.2482	0.9174	29.584526	asthma
299	6	32828908	TAP2	c.2059T>C	stop_lost	0.2322	0.2478	0.9180	29.033952	asthma
300	6	32828974	TAP2	c.1993A>G	missense	0.2331	0.2487	0.9179	29.278272	asthma
301	6	32829520	TAP2	c.1812A>G	synonymous	0.2329	0.2484	0.9186	28.844057	asthma
302	6	32830032	TAP2	c.1693G>A	missense	0.1079	0.0986	1.1050	20.737312	asthma
303	6	32830771	TAP2	c.1308C>T	synonymous	0.1058	0.1122	0.9360	9.812197	asthma
304	6	32832635	TAP2	c.1135G>A	missense	0.1472	0.1397	1.0636	10.850165	asthma
305	6	32837530	TAP2	c.608+7G>A	splice	0.3043	0.2937	1.0519	12.312382	asthma
306	6	32842666	PSMB8	c.395+6C>T	splice	0.4674	0.4542	1.0543	15.576263	asthma
307	6	32857313	PSMB9	c.179G>A	missense	0.2891	0.2775	1.0590	15.109915	asthma
308	6	32976317	BRD2	c.678G>C	synonymous	0.1029	0.0956	1.0848	13.588380	asthma
309	6	32980649	BRD2	c.2337C>T	synonymous	0.1042	0.0973	1.0794	12.211973	asthma
310	9	6255967	IL33	c.613-1G>C	splice_ac...	0.0029	0.0047	0.6240	17.423198	asthma
311	11	61783884	MYRF	c.3033T>C	synonymous	0.3407	0.3504	0.9580	9.686344	asthma
312	12	56042145	RPS26	c.-22C>G	5_prime_U...	0.4408	0.4259	0.9412	8.951558	rhinitis
313	12	56042145	RPS26	c.-22C>G	5_prime_U...	0.4395	0.4262	0.9476	15.896196	asthma
314	12	56083910	ERBB3	c.234+8A>T	splice	0.4397	0.4279	0.9528	13.032358	asthma
315	12	57104437	STAT6	n.405A>G	non_coding	0.0045	0.0059	0.7562	8.960983	asthma
316	12	57141483	LRP1	c.300C>T	synonymous	0.1407	0.1491	0.9341	13.041102	asthma
317	12	71125316	TSPAN8	c.*18C>G	3_prime_UTR	0.4009	0.4115	0.9572	10.392223	asthma
318	12	71139754	TSPAN8	c.218G>C	missense	0.3982	0.4089	0.9562	11.111091	asthma
319	15	67236036	AAGAB	c.394A>C	missense	0.3139	0.3259	0.9462	14.936667	asthma
320	16	27226713	NSMCE1	c.600+7G>A	splice	0.3611	0.3512	1.0439	9.576099	asthma
321	16	27226789	NSMCE1	c.531G>A	synonymous	0.3637	0.3544	1.0413	8.798603	asthma
322	16	27344882	IL4R	c.178A>G	missense	0.4612	0.4515	1.0400	8.932557	asthma
323	16	27345038	IL4R	n.700C>T	splice	0.3686	0.3562	1.0550	14.951558	asthma
324	17	4632019	ALOX15	c.1679C>T	missense	0.0166	0.0214	0.7726	11.609065	rhinitis
325	17	39657827	STARD3	c.350G>A	missense	0.3452	0.3305	0.9366	21.407601	asthma
326	17	39666058	TCAP	c.453A>C	synonymous	0.2775	0.2635	0.9315	22.176526	asthma
327	17	39674647	PGAP3	c.465T>C	synonymous	0.3161	0.2997	0.9261	27.670602	asthma
328	17	39727784	ERBB2	c.3508C>G	missense	0.3360	0.3200	0.9302	25.534320	asthma

(continued)

	chr	pos	Gene	cDNA	Consequence	Case.MAF	Control.MAF	Odds.ratio	p	trait
329	17	39766006	IKZF3	c.1080C>T	synonymous	0.4585	0.4813	0.9125	45.298259	asthma
330	17	39864166	IKZF3	c.-40A>G	5_prime_UTR	0.4947	0.4784	0.9368	10.445269	rhinitis
331	17	39864166	IKZF3	c.-40A>G	5_prime_UTR	0.4974	0.4786	0.9082	49.755723	asthma
332	17	39868373	ZPBP2	c.19C>T	synonymous	0.4544	0.4784	0.9080	50.000000	asthma
333	17	39872381	ZPBP2	c.518G>T	missense	0.4967	0.4810	0.9393	9.748362	rhinitis
334	17	39872381	ZPBP2	c.518G>T	missense	0.4948	0.4812	0.9085	49.846490	asthma
335	17	39875421	ZPBP2	c.876C>T	synonymous	0.4540	0.4783	0.9069	50.000000	asthma
336	17	39905943	GSDMB	c.892C>T	missense	0.4469	0.4717	0.9052	50.000000	asthma
337	17	39905964	GSDMB	c.871G>A	missense	0.4968	0.4878	0.9404	9.355561	rhinitis
338	17	39905964	GSDMB	c.871G>A	missense	0.4872	0.4880	0.9054	50.000000	asthma
339	17	39965740	GSDMA	c.53G>A	missense	0.4656	0.4467	1.0792	31.557834	asthma
340	17	39966427	GSDMA	c.382G>T	missense	0.4891	0.4933	0.9320	26.919013	asthma
341	17	39966433	GSDMA	c.388G>A	missense	0.3216	0.3356	0.9384	19.294393	asthma
342	17	39974934	GSDMA	c.941C>A	missense	0.4843	0.4682	1.0665	22.652085	asthma
343	17	39981111	PSMD3	c.141G>A	synonymous	0.4445	0.4576	0.9484	15.701365	asthma
344	17	40016890	CSF3	c.555G>A	synonymous	0.3624	0.3757	0.9446	16.975514	asthma
345	17	40020003	MED24	c.237T>A	synonymous	0.3606	0.3741	0.9435	16.757459	asthma
346	17	40023239	MED24	c.2103T>C	synonymous	0.3635	0.3764	0.9460	16.148375	asthma
347	17	40026837	MED24	n.313C>A	non_coding	0.3707	0.3836	0.9466	15.887060	asthma
348	17	49406866	PHB	n.608C>G	non_coding	0.3424	0.3328	1.0441	9.526075	asthma
349	22	41437112	TOB2	c.234G>A	synonymous	0.2106	0.2026	0.9523	9.182369	asthma
350	22	41507809	ACO2	c.192A>C	synonymous	0.2092	0.2011	1.0505	9.293965	asthma
351	22	41743074	MEI1	c.1332-6A>G	splice	0.1867	0.1790	0.9497	9.274742	asthma
352	22	41763225	MEI1	c.2172G>T	synonymous	0.1880	0.1805	0.9510	8.912574	asthma

Figures genome context

- From top: Cytogenetic bands were obtained from http://genome.ucsc.edu
- Gene transcripts were downloaded from http://www.ensembl.org/biomart/martview
- GWAS ß values obtained from http://genepi.qimr.edu.au/staff/manuelf/gwas_results/SHARE-without23andMe.LDSCORE-GC.SE-META.v0(A. Sample does not include 23andme samples
- Only results with P > -log10(8^10-7) are given for the exome analysis while all P > -log10(1^10-30) are truncated at this value

Fig S13 AAGAB

alpha and gamma adaptin binding protein [Source:HGNC Symbol;Acc:HGNC:25662]

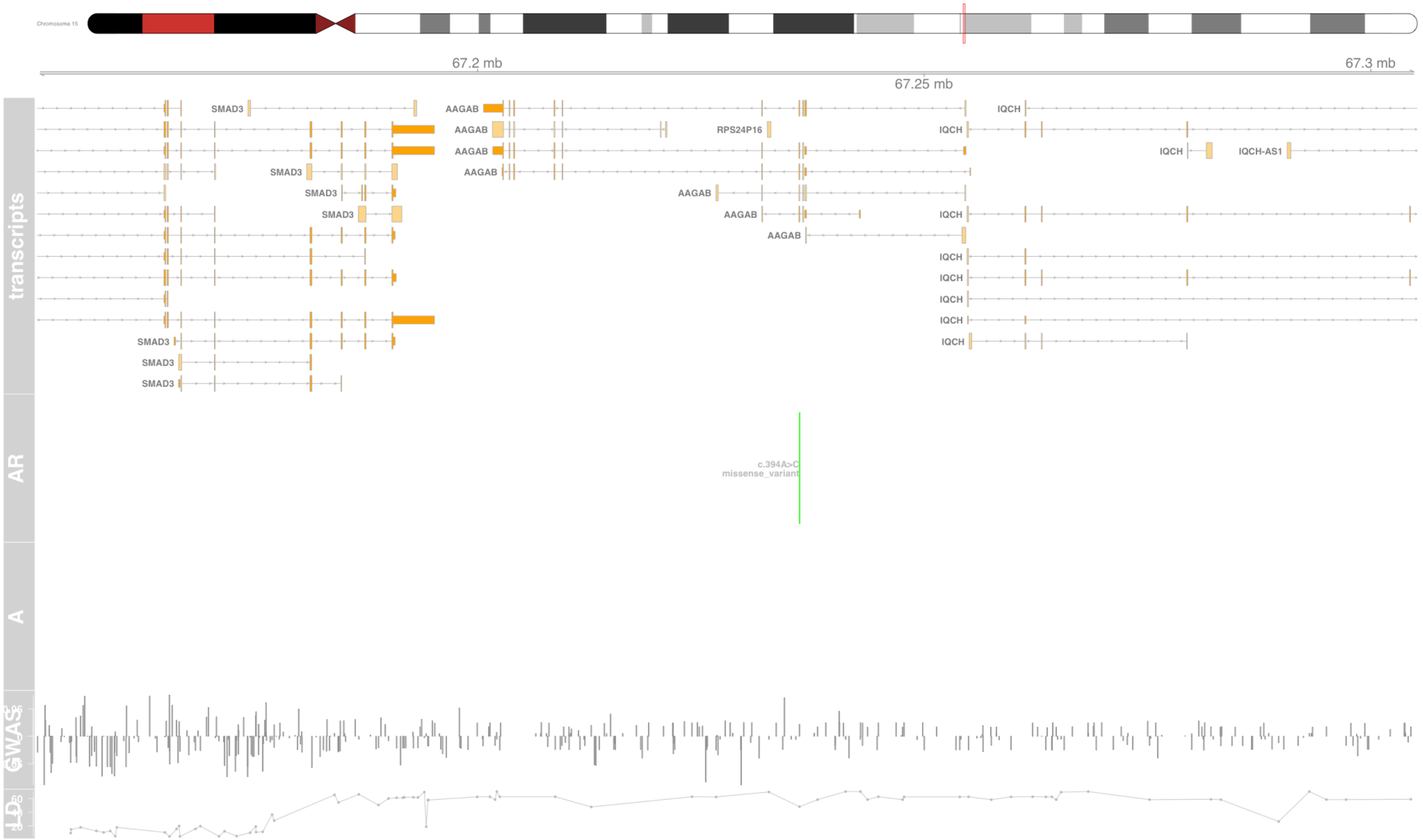

Fig S14 ACO2

aconitase 2 [Source:HGNC Symbol;Acc:HGNC:118]

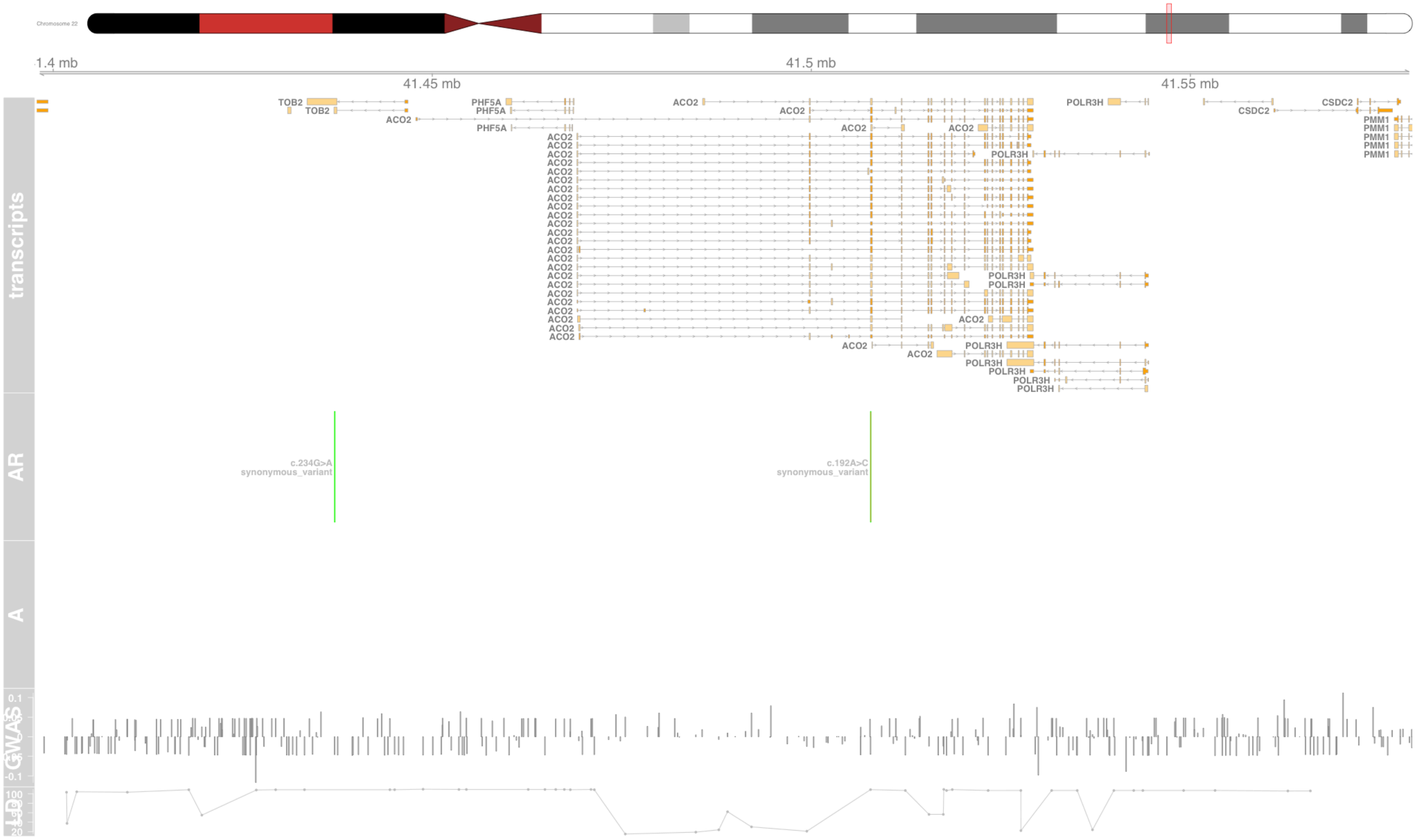

Fig S15 ADAM19

ADAM metallopeptidase domain 19 [Source:HGNC Symbol;Acc:HGNC:197]

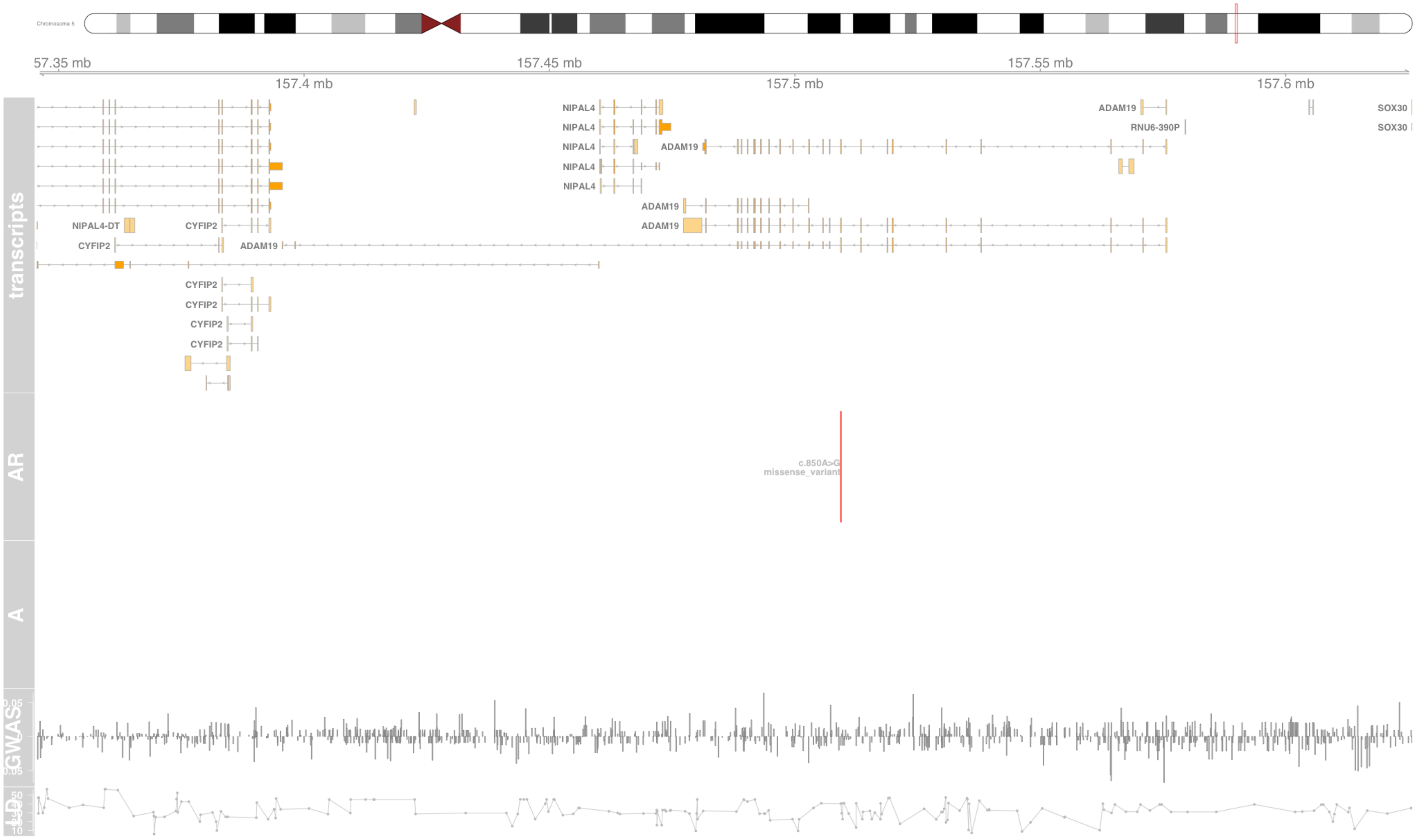

Fig S16 AGER

advanced glycosylation end-product specific receptor [Source:HGNC Symbol;Acc:HGNC:320]

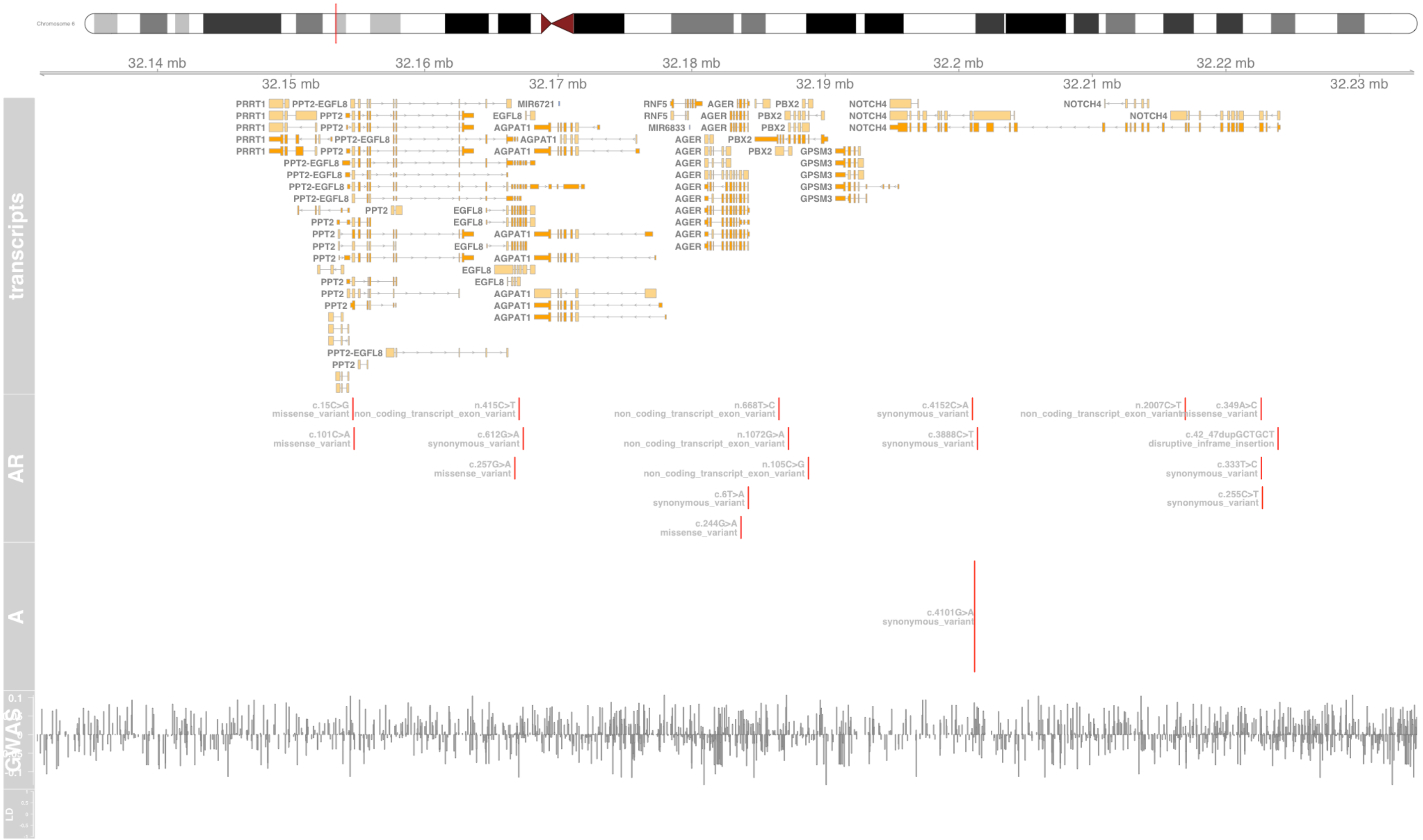

Fig S17 AIF1

allograft inflammatory factor 1 [Source:HGNC Symbol;Acc:HGNC:352]

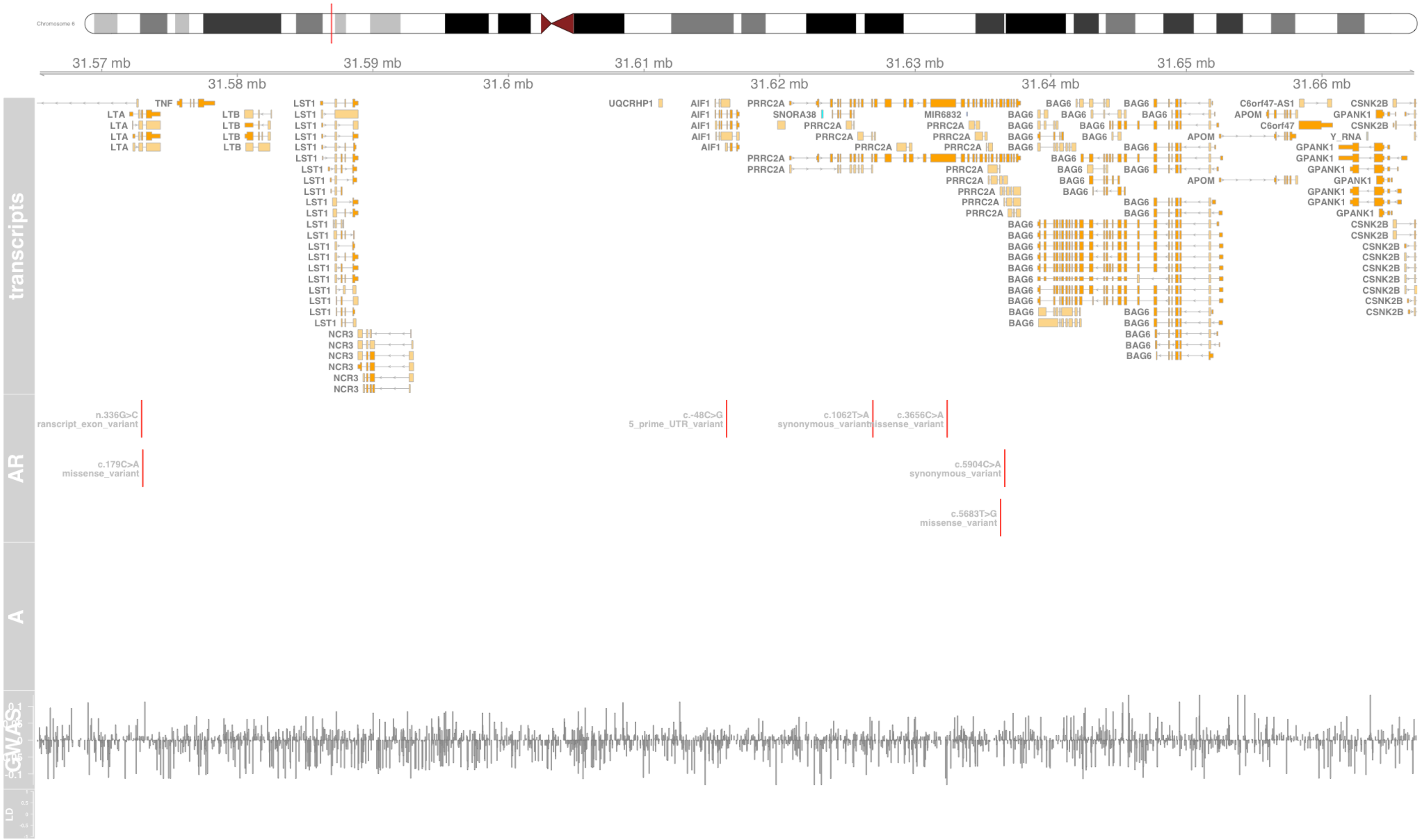

Fig S18 ALOX15

arachidonate 15-lipoxygenase [Source:HGNC Symbol;Acc:HGNC:433]

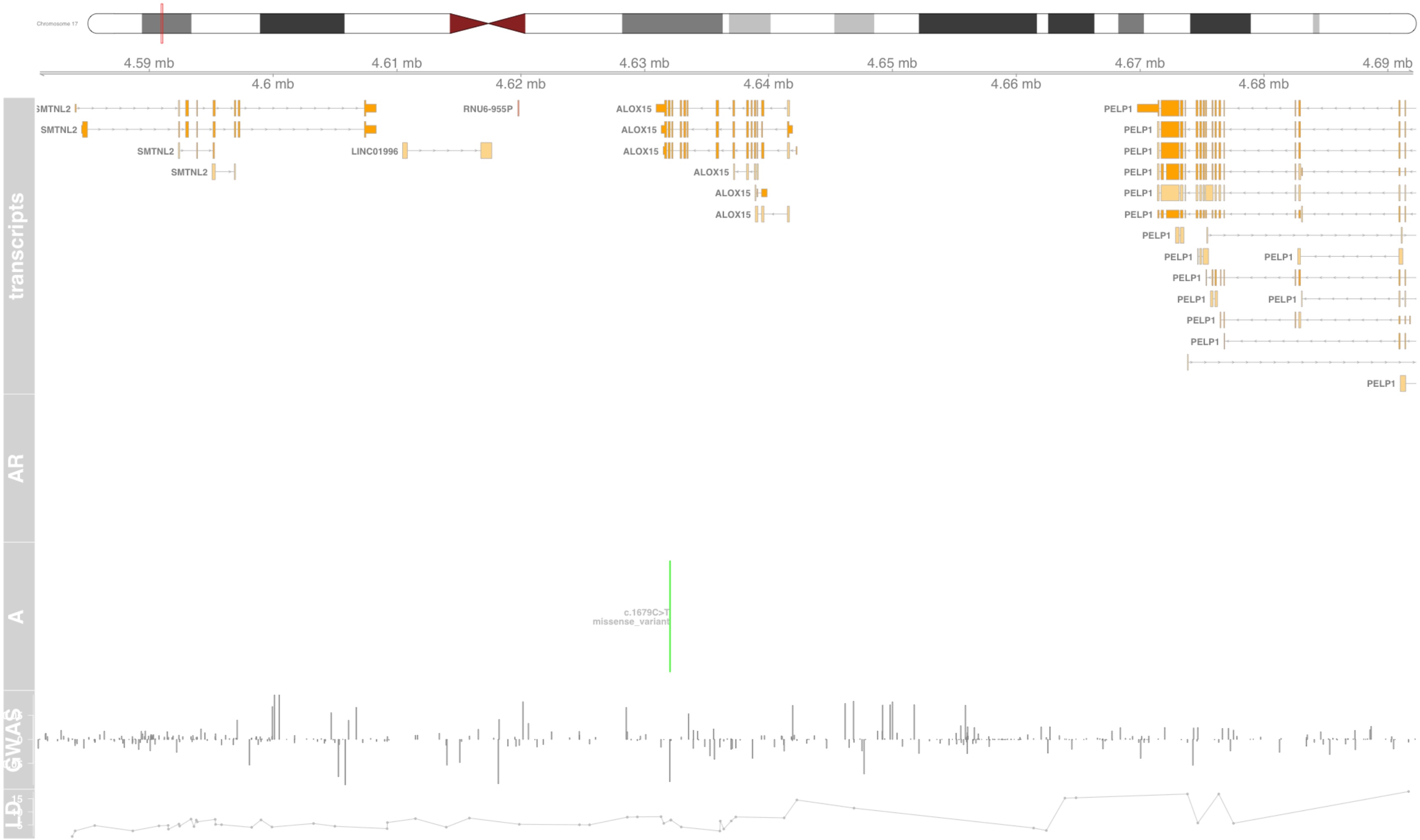

Fig S19 ATAT1

alpha tubulin acetyltransferase 1 [Source:HGNC Symbol;Acc:HGNC:21186]

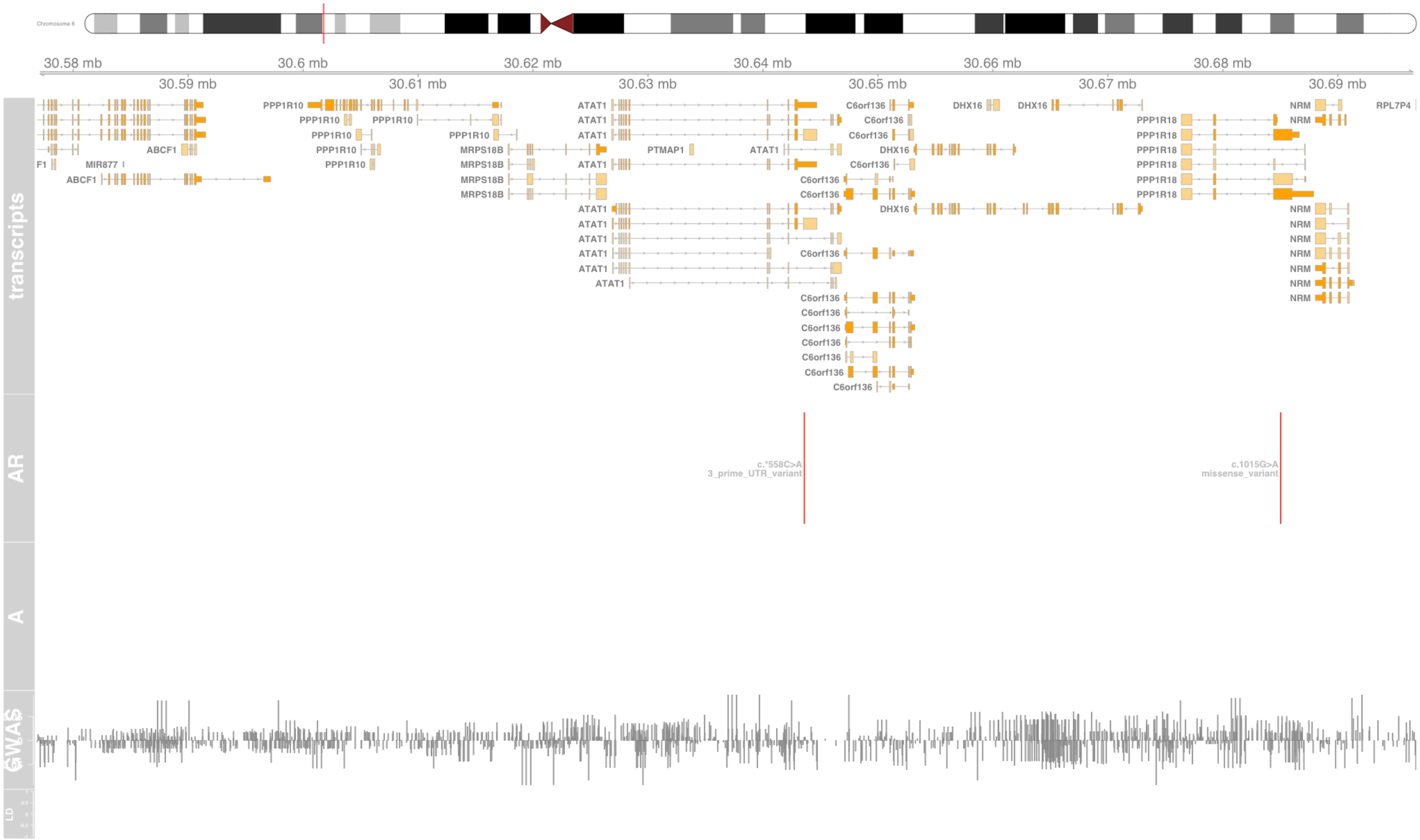

Fig S20 ATF6B

activating transcription factor 6 beta [Source:HGNC Symbol;Acc:HGNC:2349]

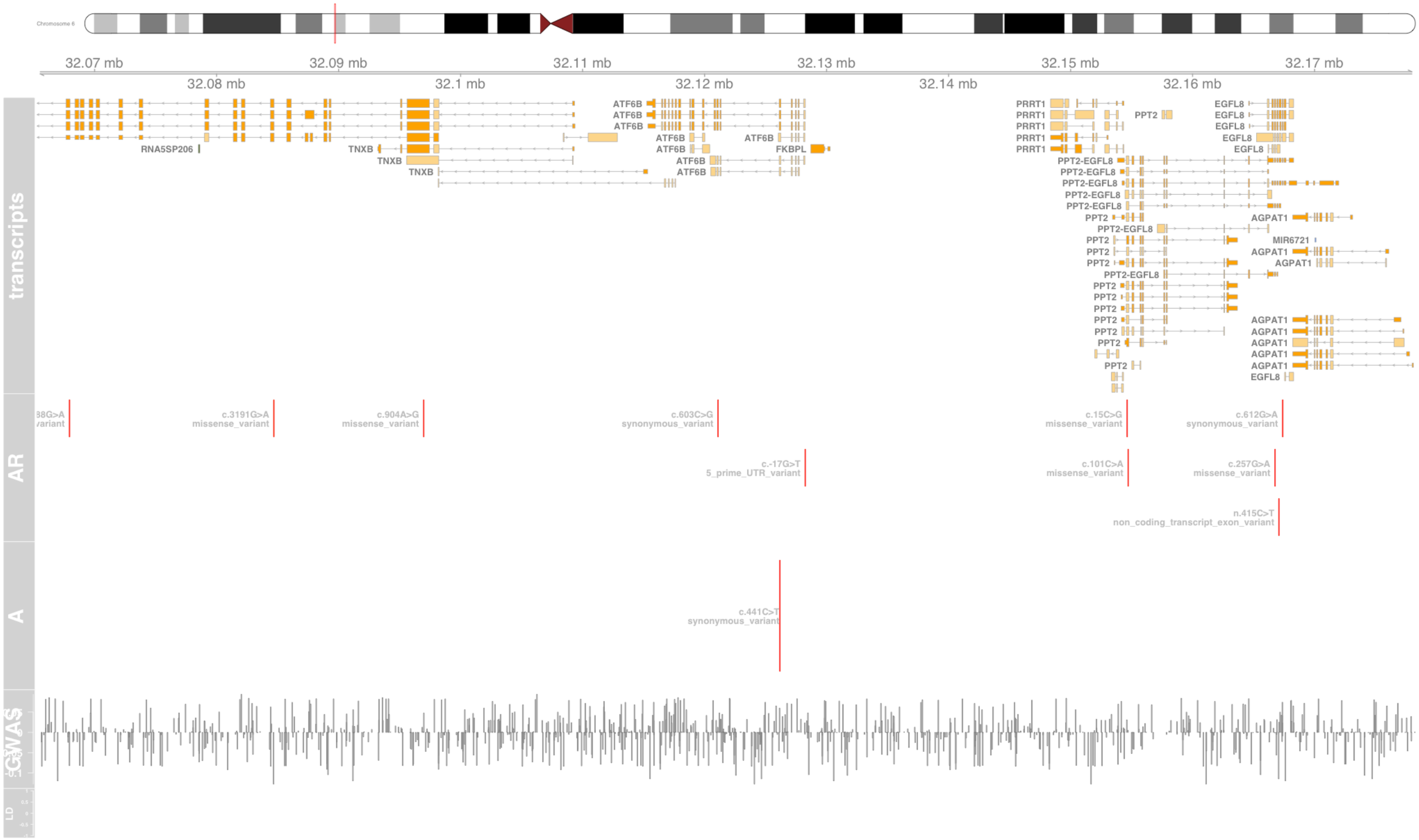

Fig S21 ATP6V1G2

ATPase H+ transporting V1 subunit G2 [Source:HGNC Symbol;Acc:HGNC:862]

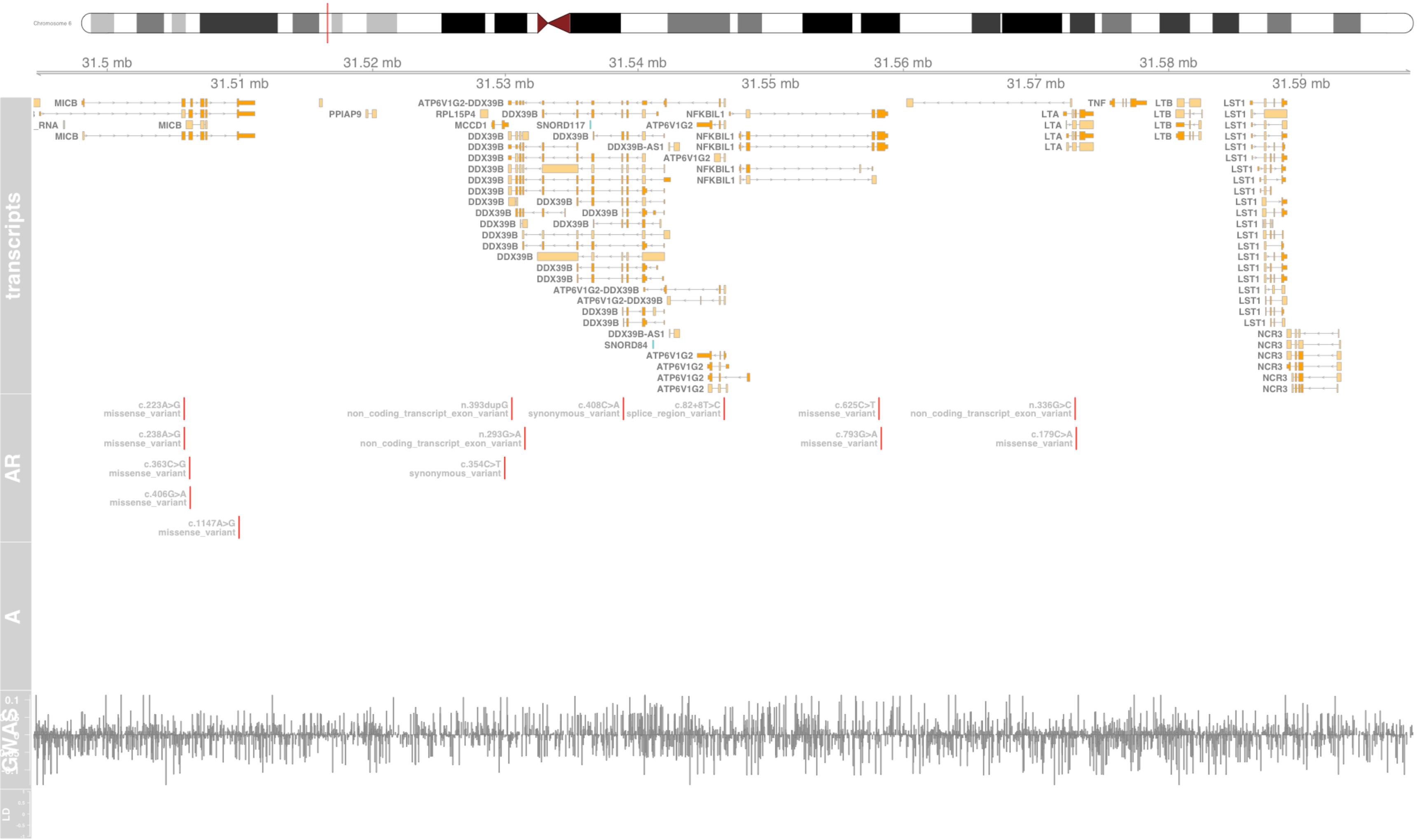

Fig S22 BRD2

bromodomain containing 2 [Source:HGNC Symbol;Acc:HGNC:1103]

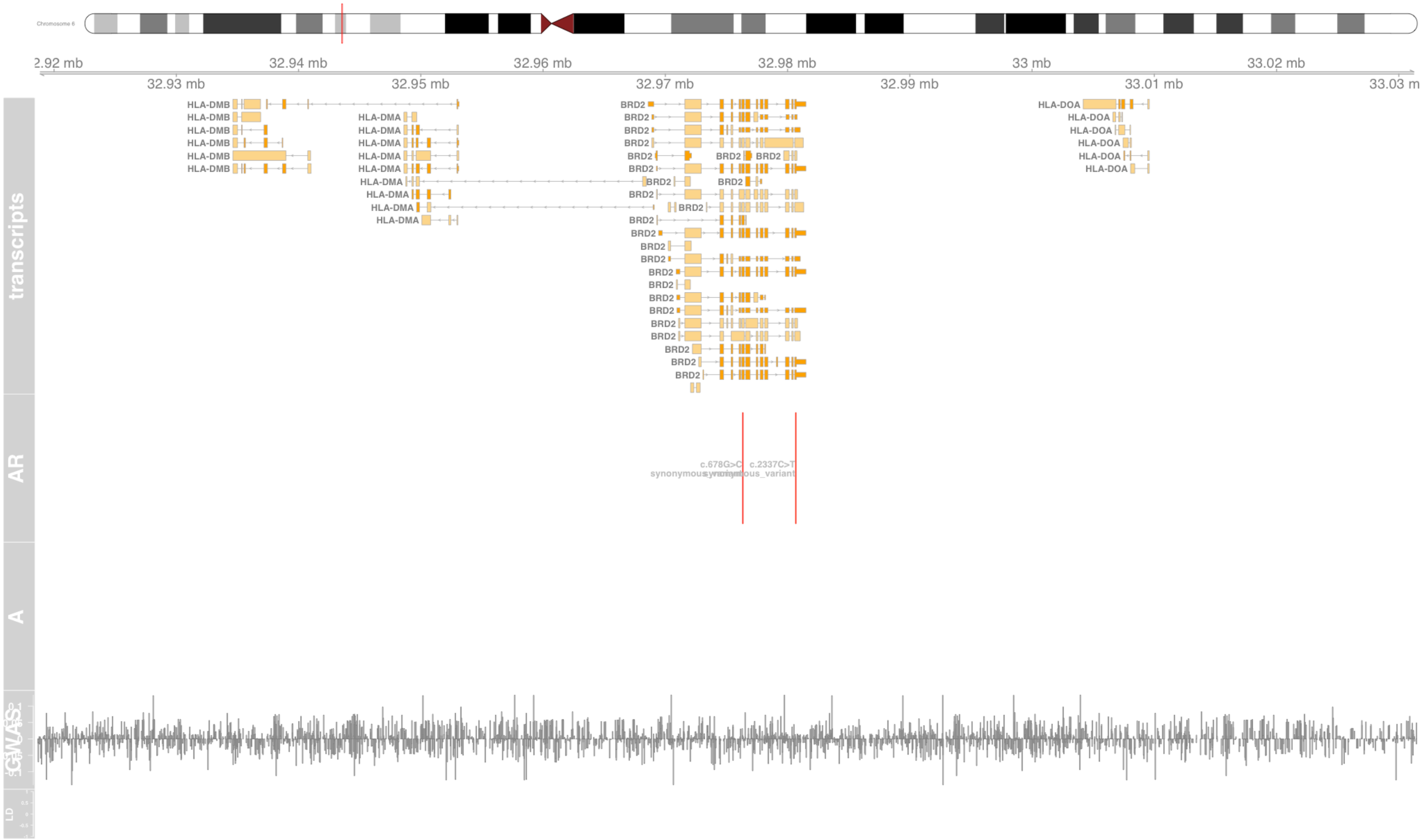

Fig S23 BTN2A1

butyrophilin subfamily 2 member A1 [Source:HGNC Symbol;Acc:HGNC:1136]

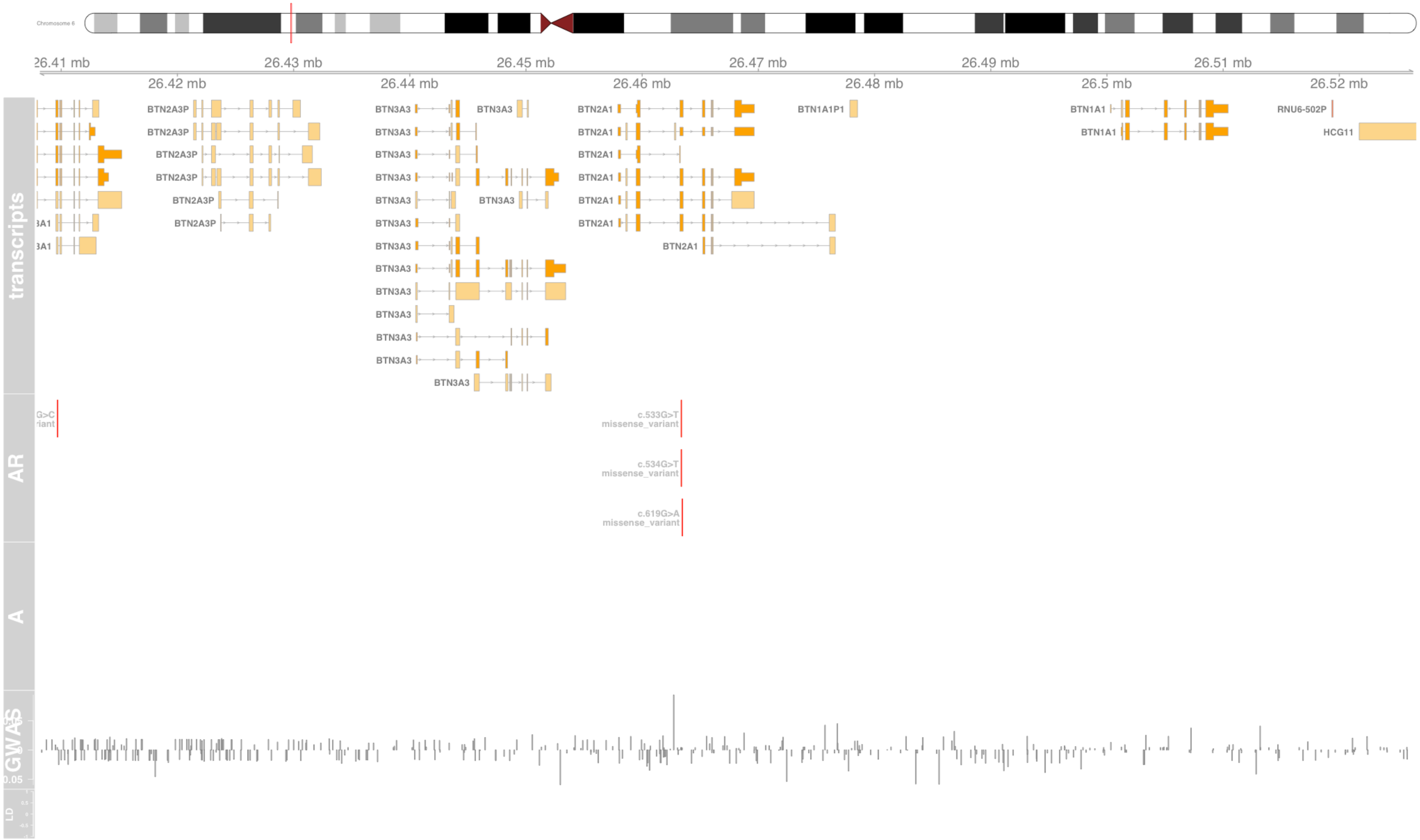

Fig S24 BTN3A1

butyrophilin subfamily 3 member A1 [Source:HGNC Symbol;Acc:HGNC:1138]

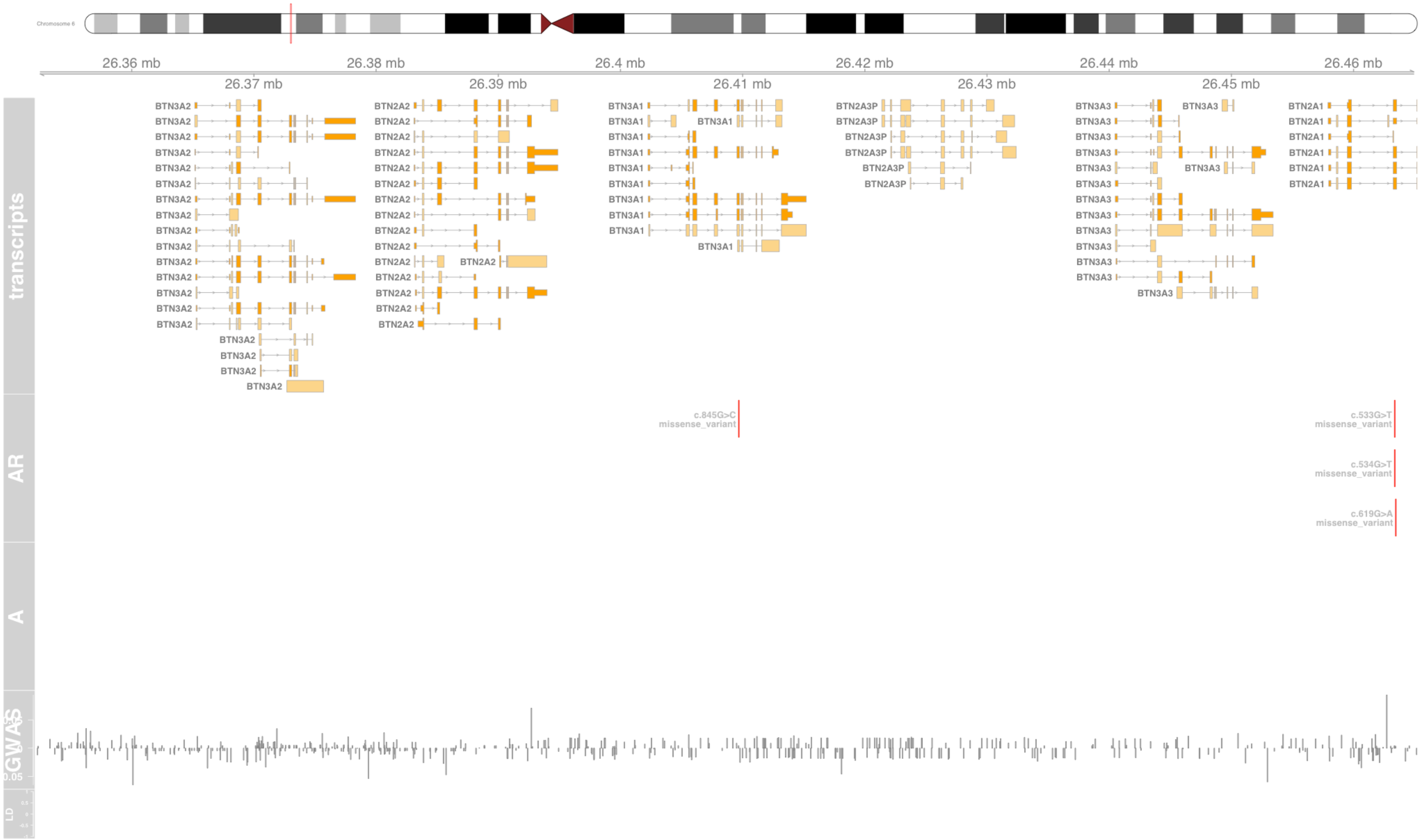

Fig S25 BTNL2

butyrophilin like 2 [Source:HGNC Symbol;Acc:HGNC:1142]

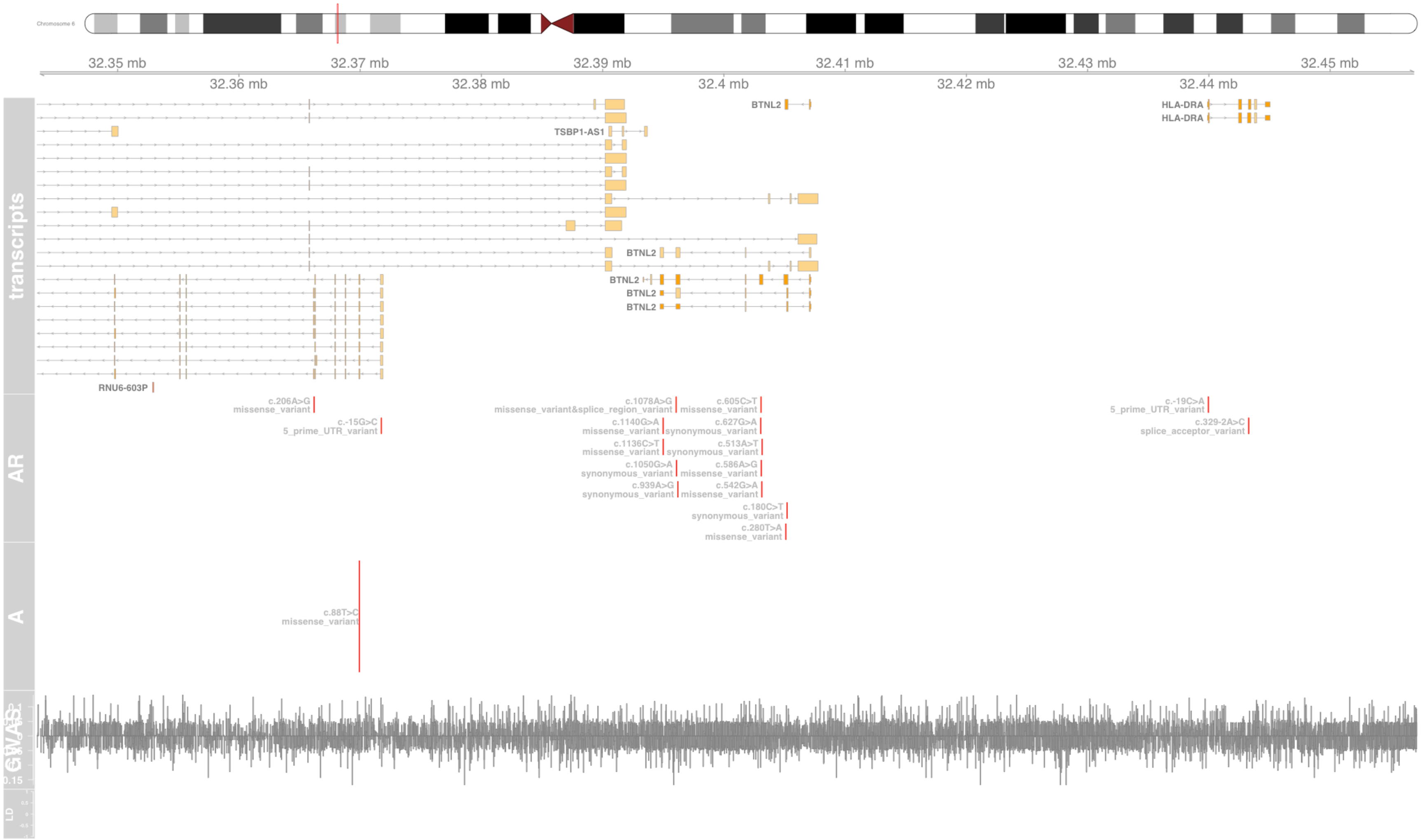

Fig S26 C6orf15

chromosome 6 open reading frame 15 [Source:HGNC Symbol;Acc:HGNC:13927]

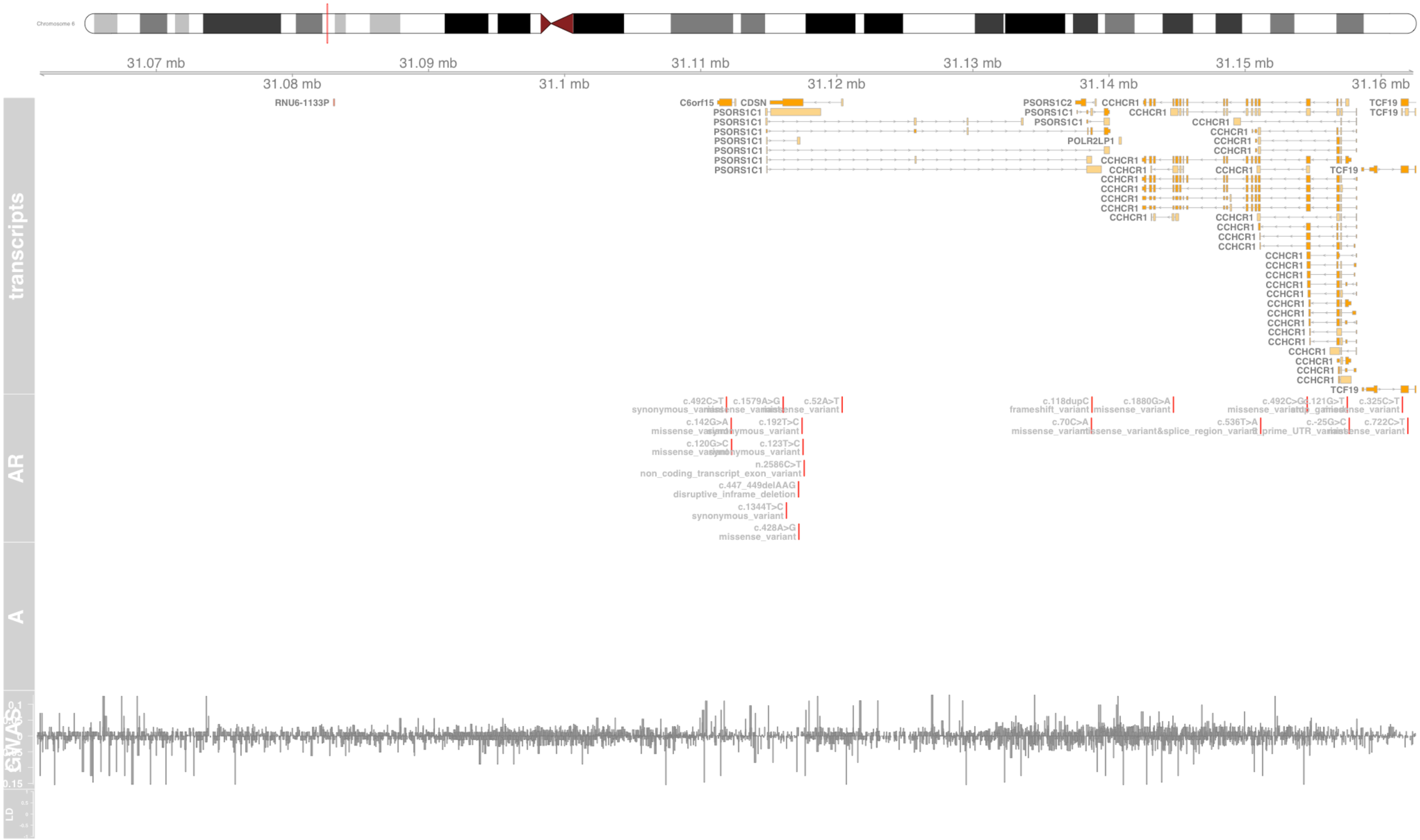

Fig S27 CCHCR1

coiled-coil alpha-helical rod protein 1 [Source:HGNC Symbol;Acc:HGNC:13930]

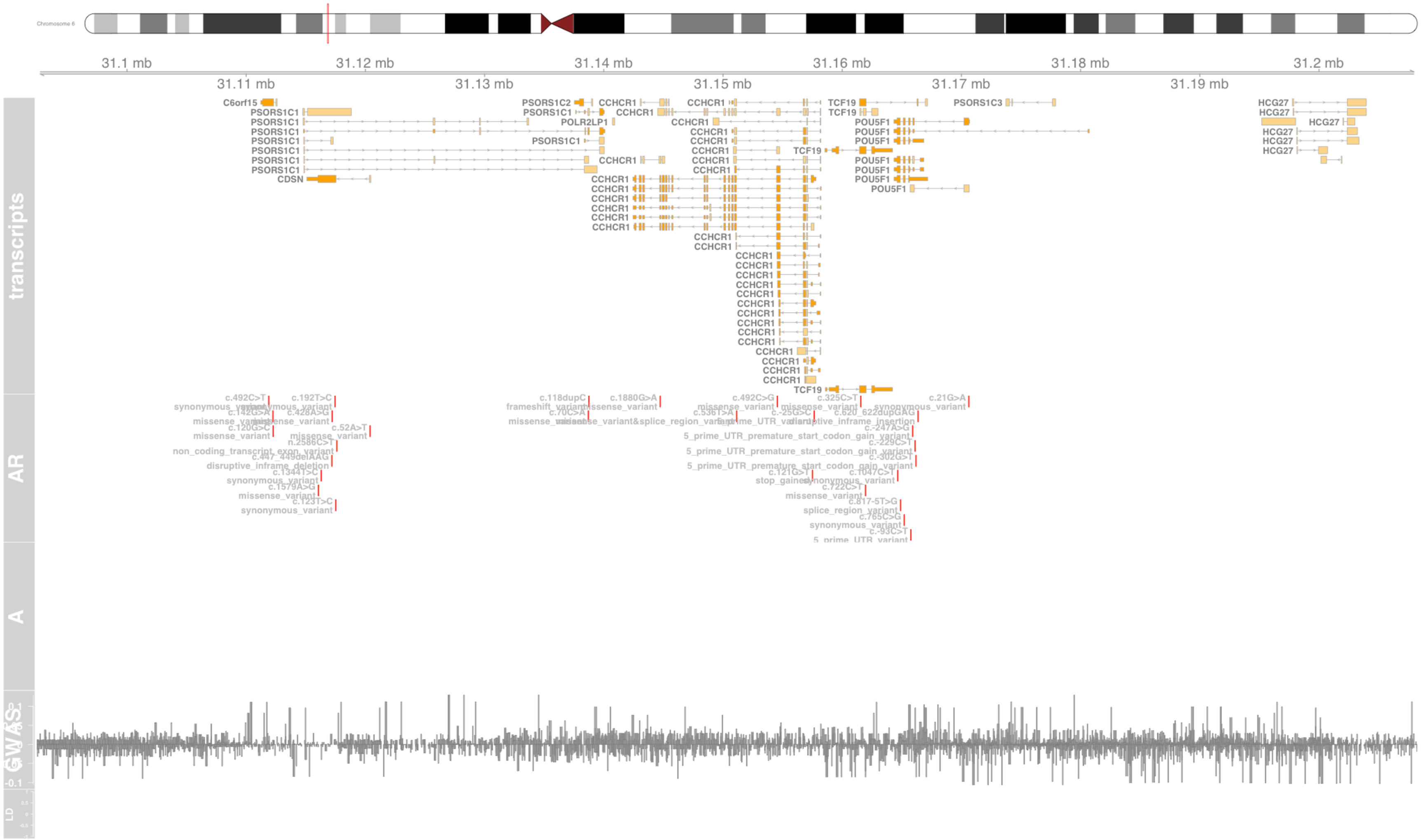

Fig S28 CD247

CD247 molecule [Source:HGNC Symbol;Acc:HGNC:1677]

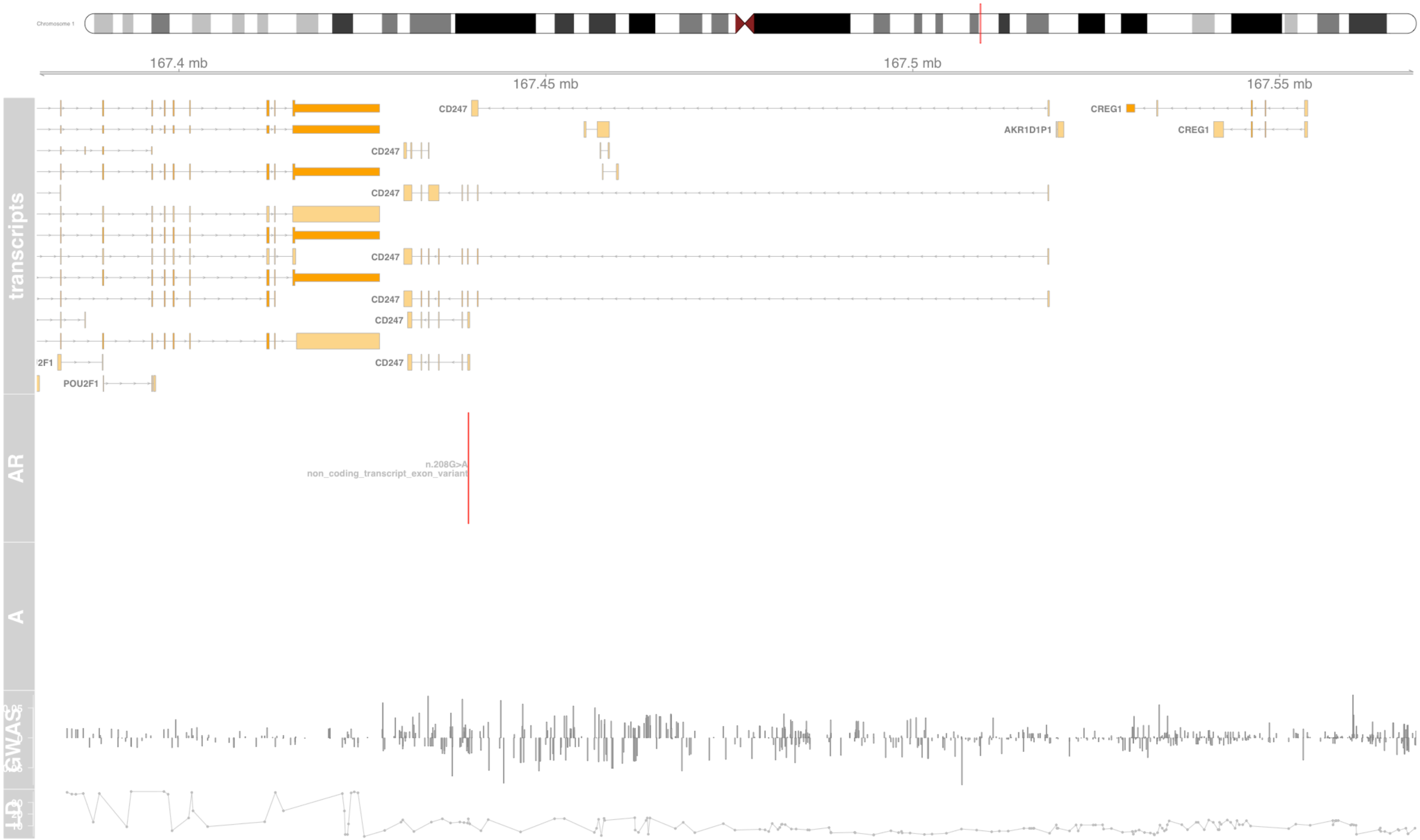

Fig S29 CDSN

corneodesmosin [Source:HGNC Symbol;Acc:HGNC:1802]

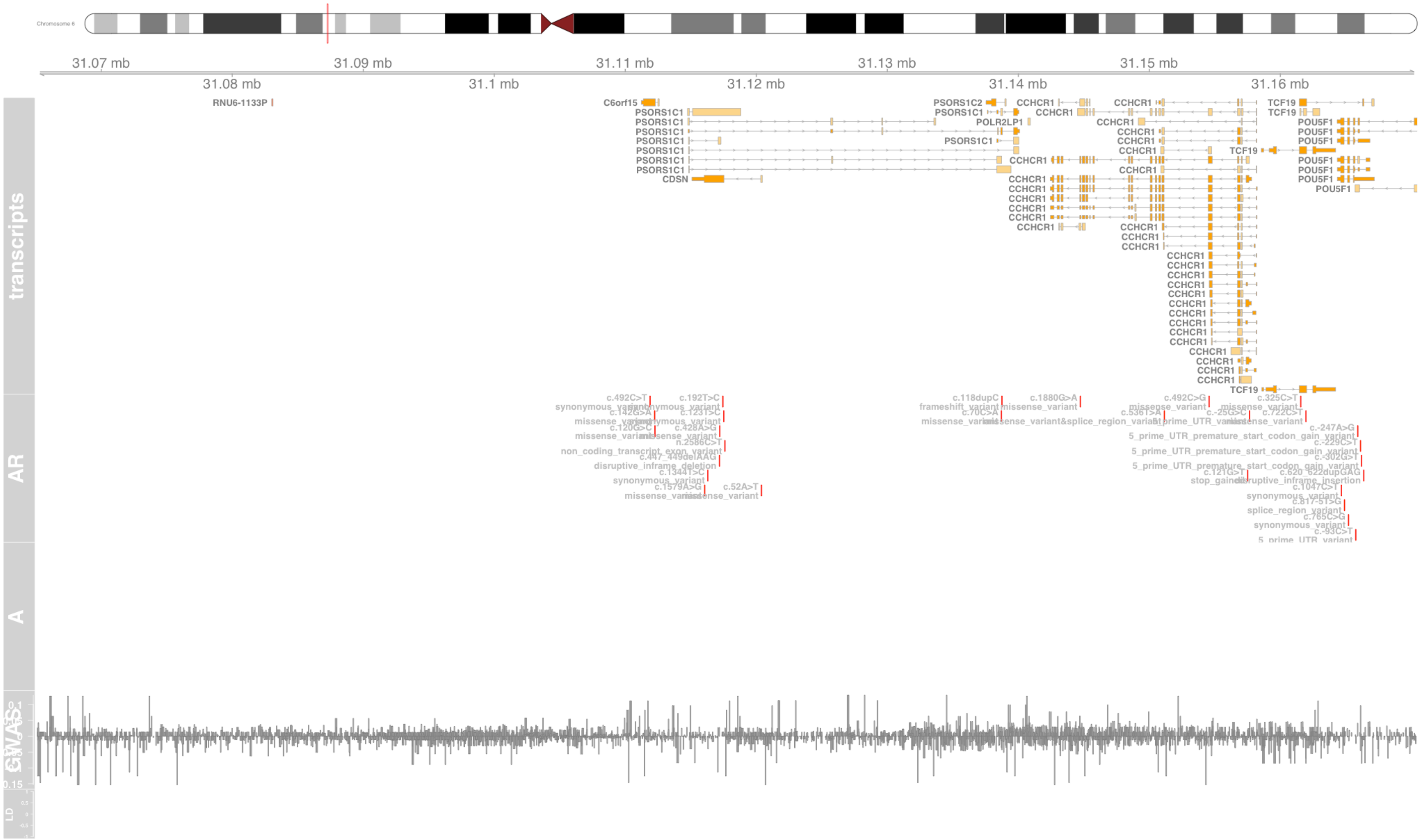

Fig S30 CFB

complement factor B [Source:HGNC Symbol;Acc:HGNC:1037]

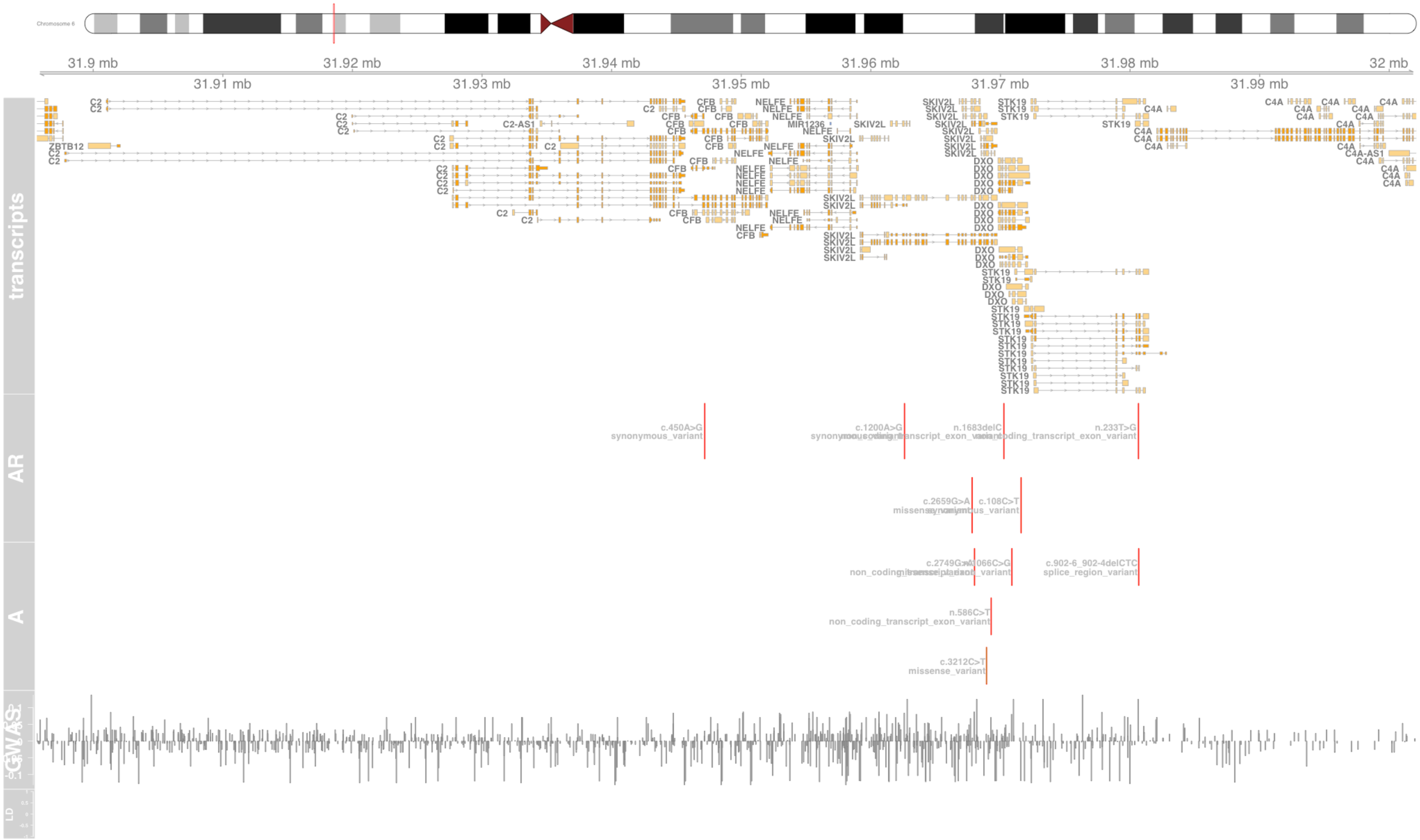

Fig S31 CSF3

colony stimulating factor 3 [Source:HGNC Symbol;Acc:HGNC:2438]

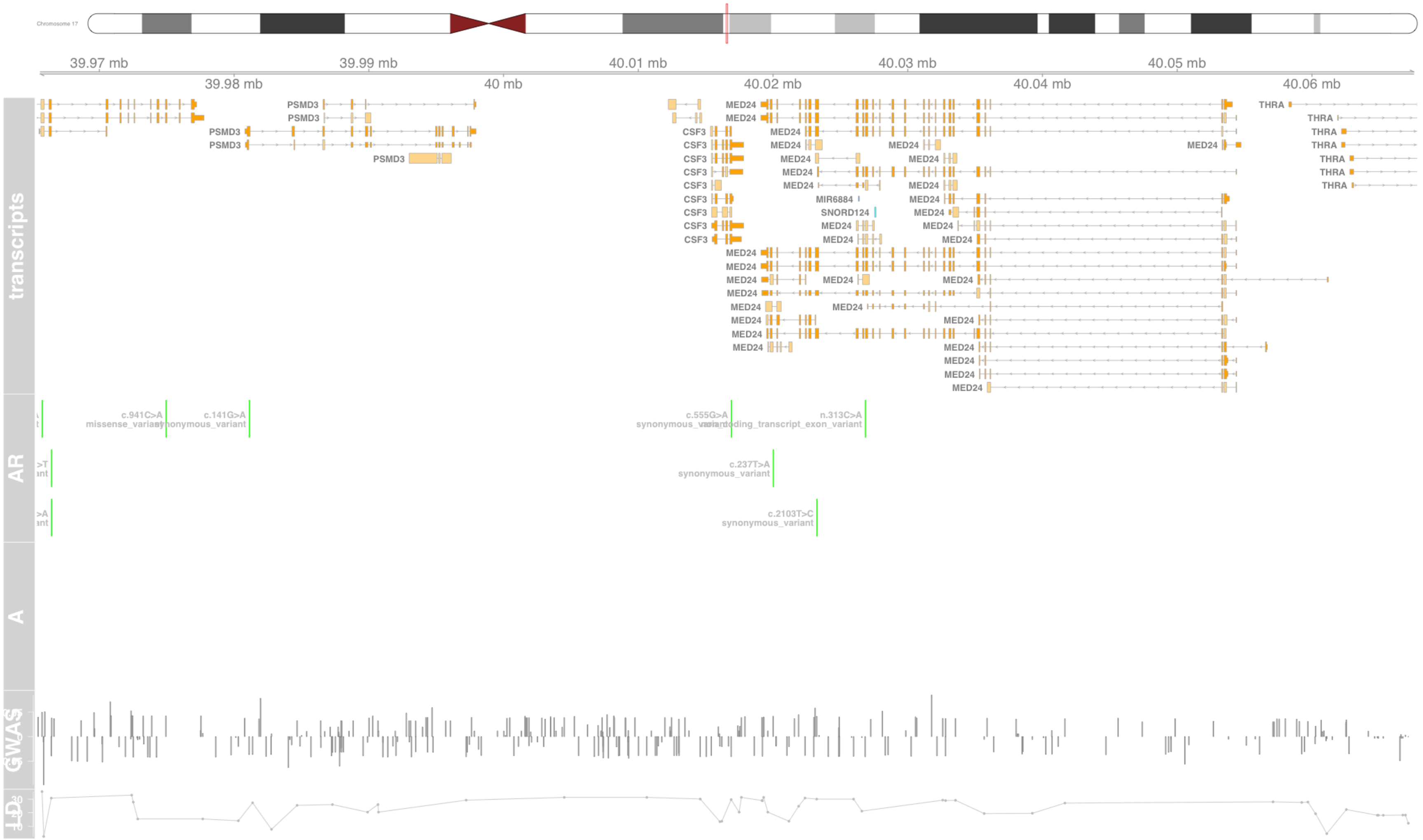

Fig S32 CYP21A1P

cytochrome P450 family 21 subfamily A member 1, pseudogene [Source:HGNC Symbol;Acc:HGNC:2599]

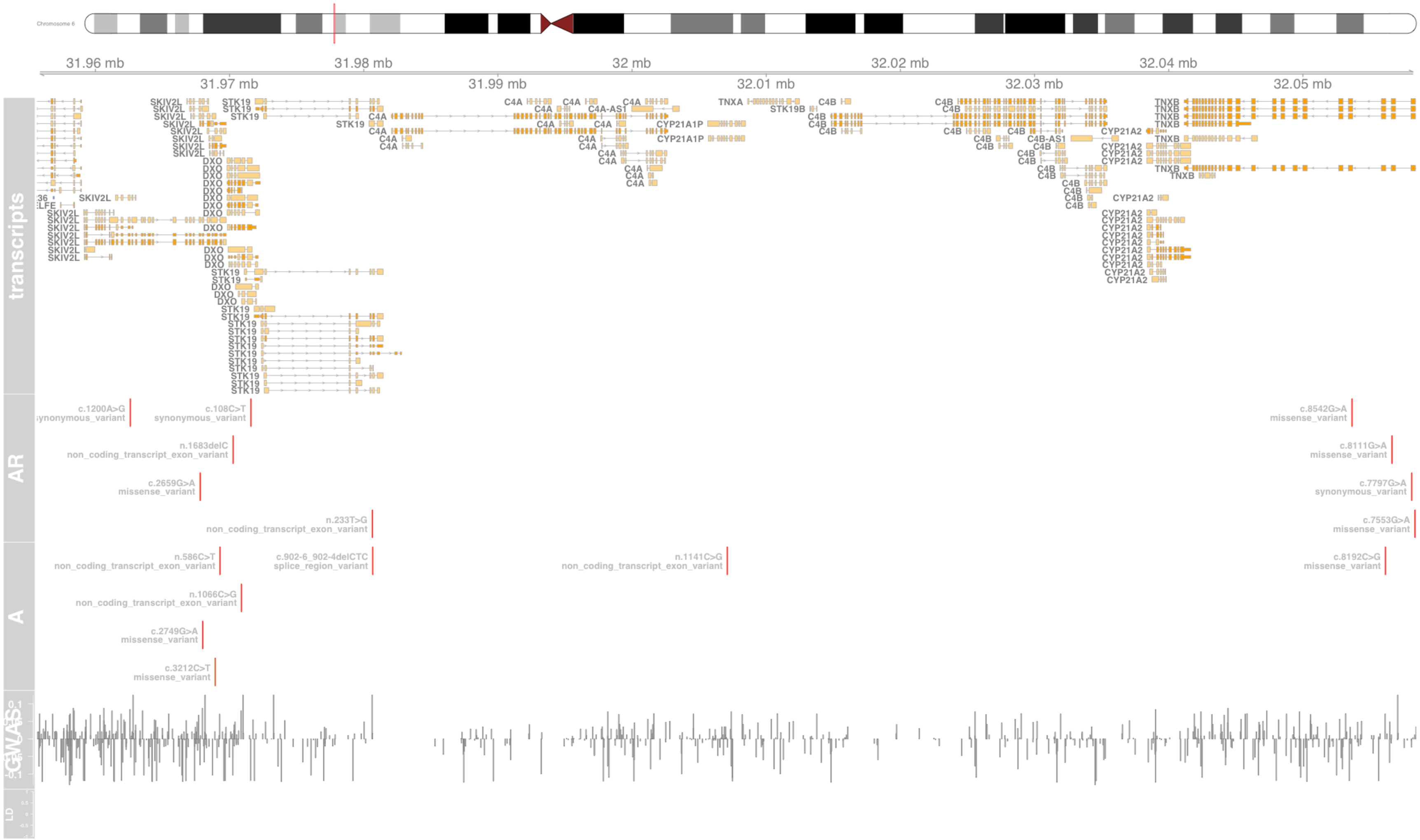

Fig S33 D2HGDH

D-2-hydroxyglutarate dehydrogenase [Source:HGNC Symbol;Acc:HGNC:28358]

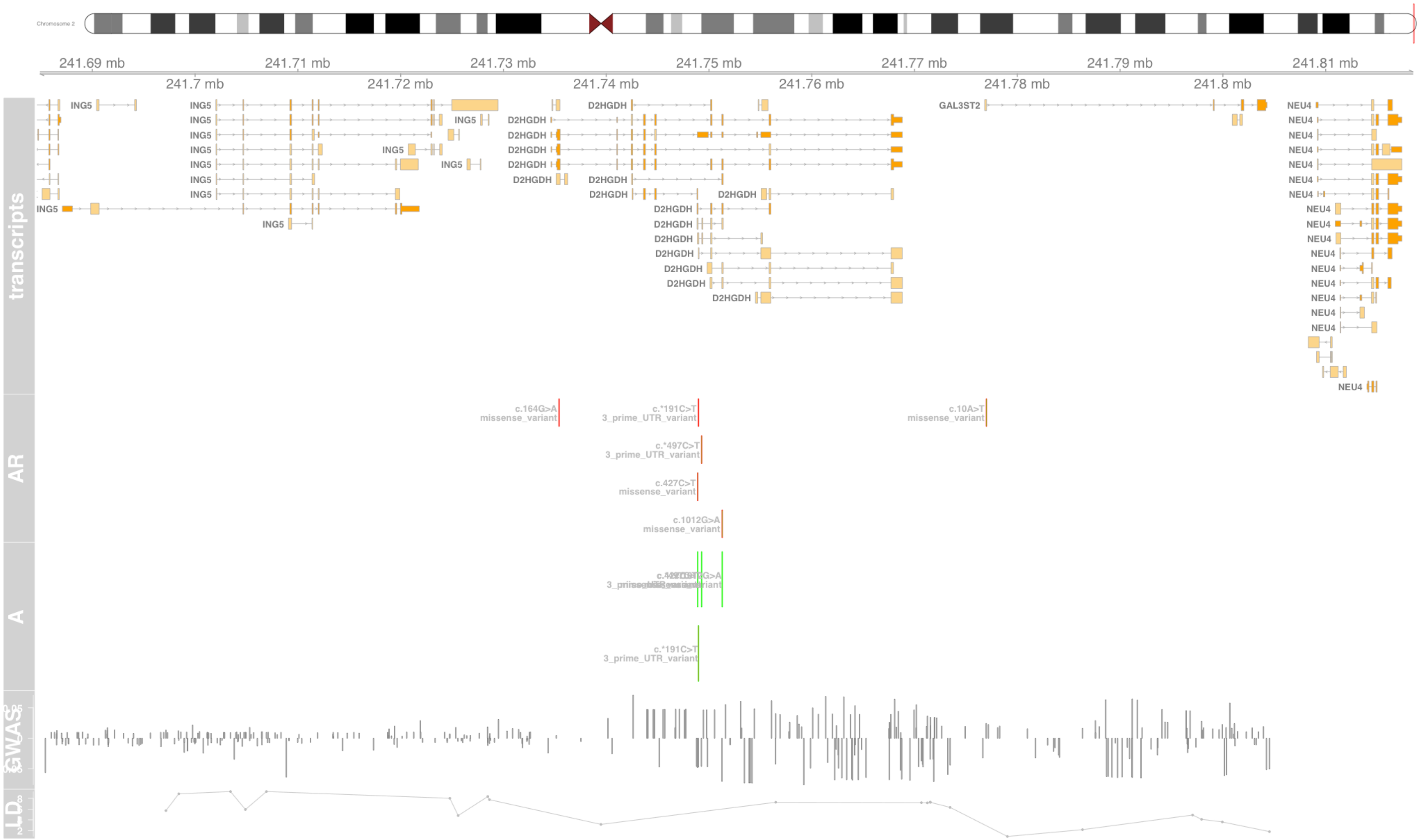

Fig S34 DDX39B

DExD-box helicase 39B [Source:HGNC Symbol;Acc:HGNC:13917]

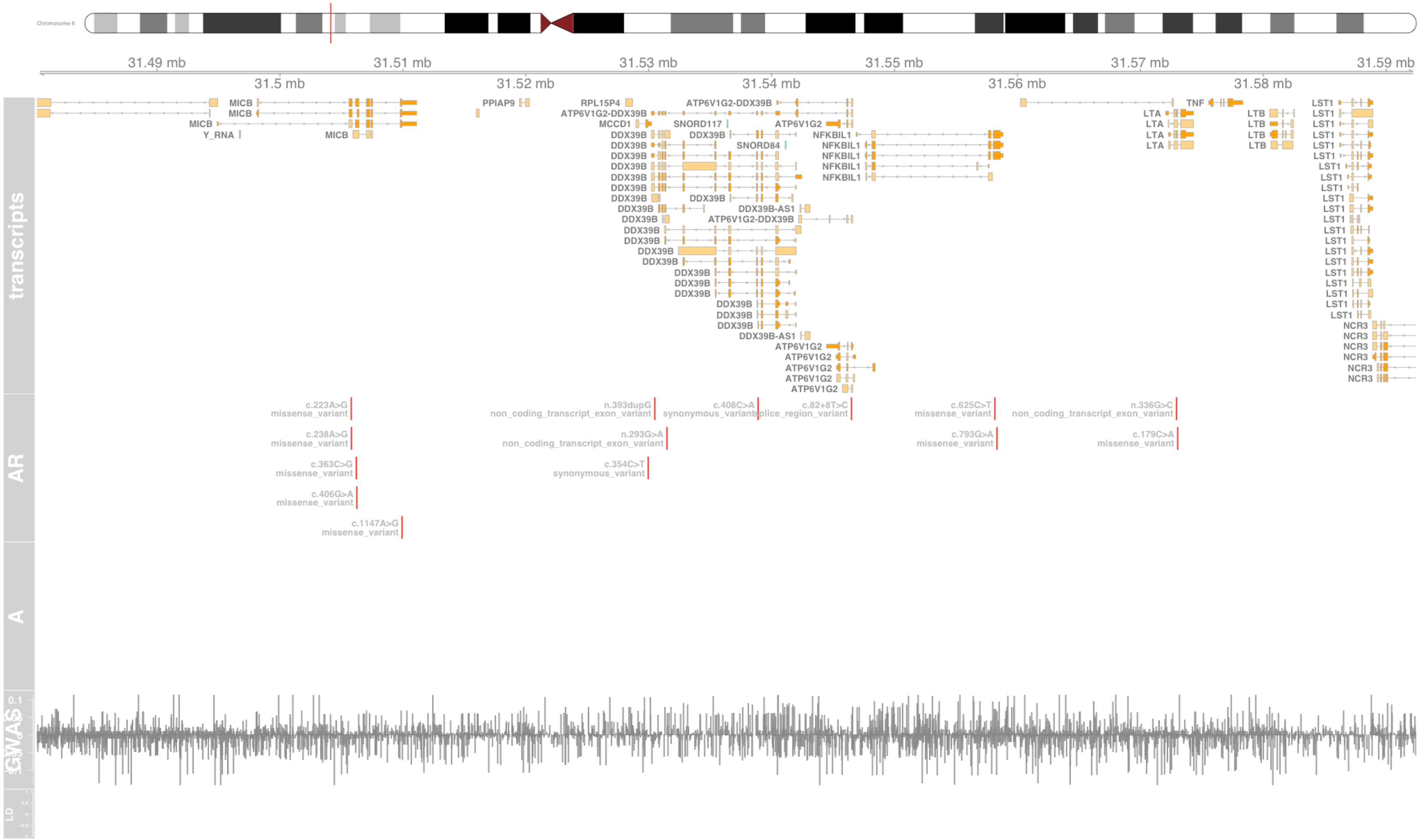

Fig S35 DXO

decapping exoribonuclease [Source:HGNC Symbol;Acc:HGNC:2992]

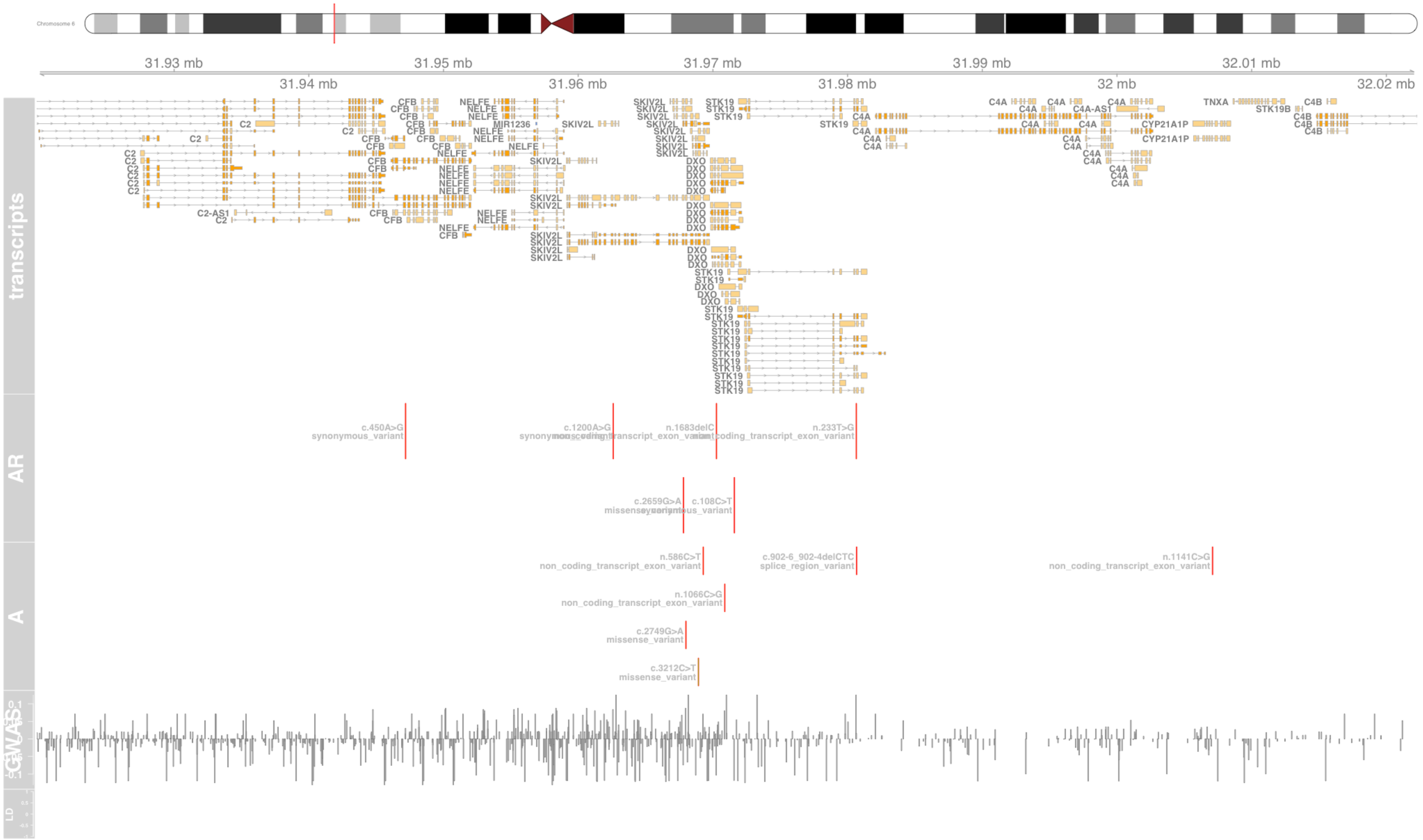

Fig S36 EGFL8

EGF like domain multiple 8 [Source:HGNC Symbol;Acc:HGNC:13944]

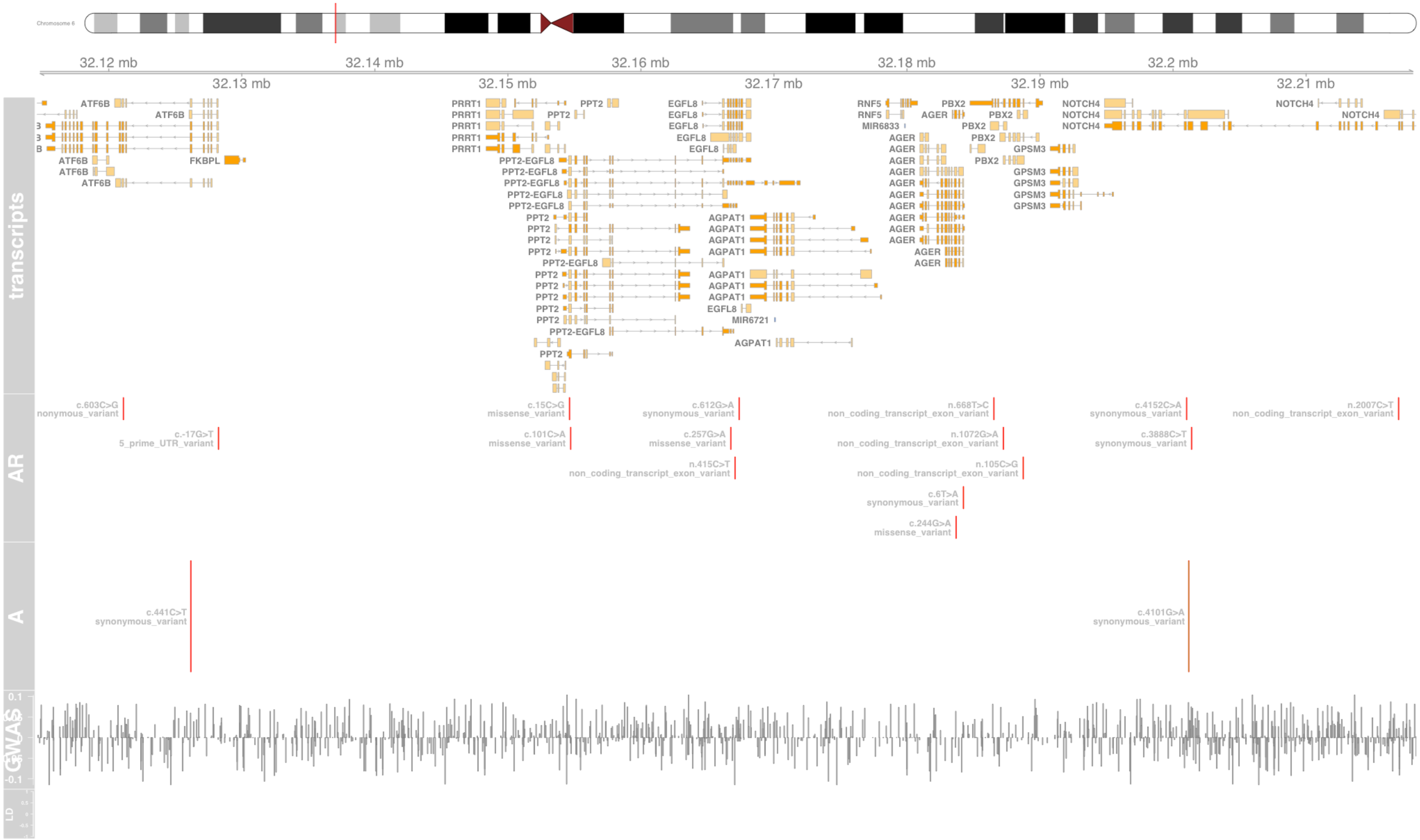

Fig S37 ERBB2

erb-b2 receptor tyrosine kinase 2 [Source:HGNC Symbol;Acc:HGNC:3430]

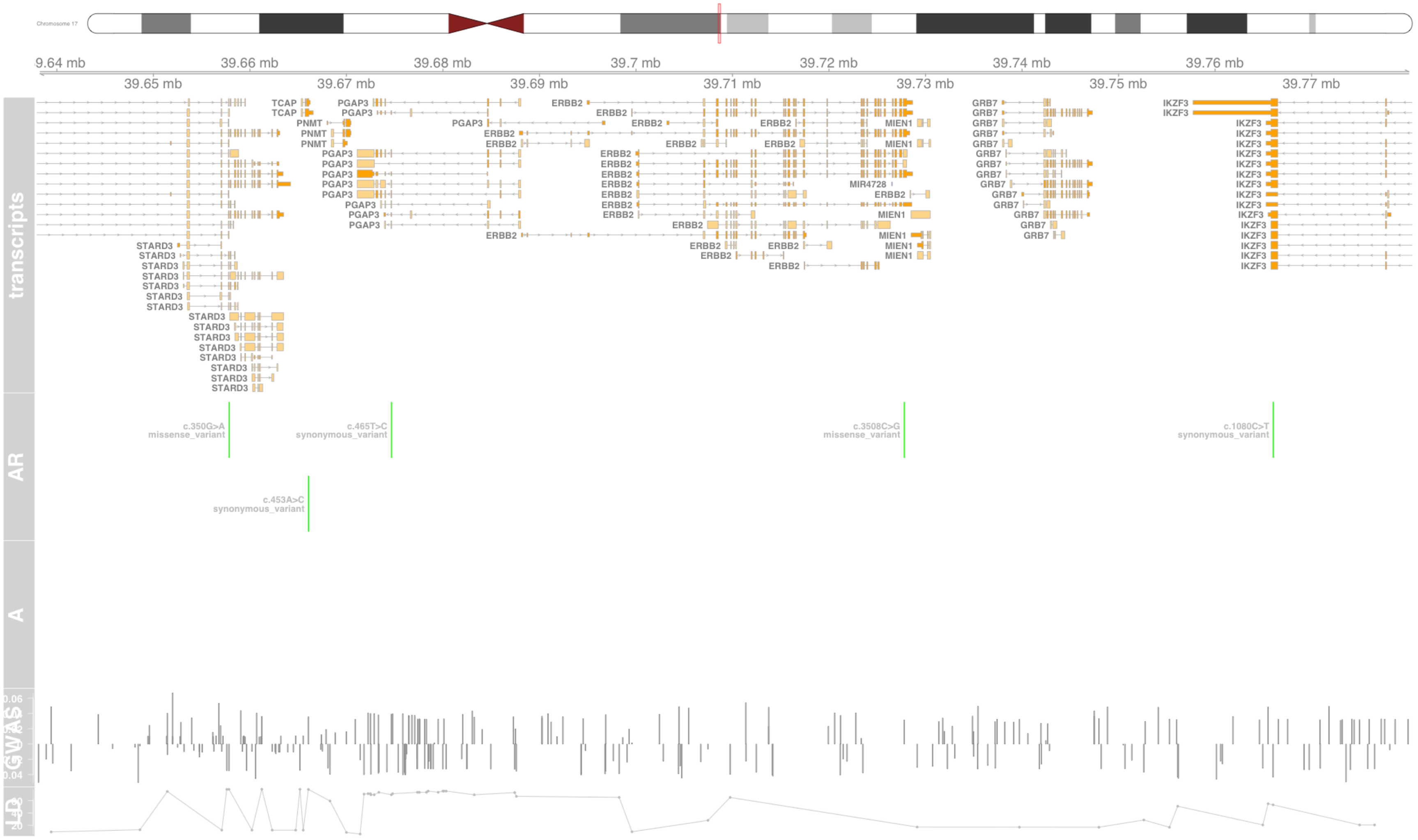

Fig S38 ERBB3

erb-b2 receptor tyrosine kinase 3 [Source:HGNC Symbol;Acc:HGNC:3431]

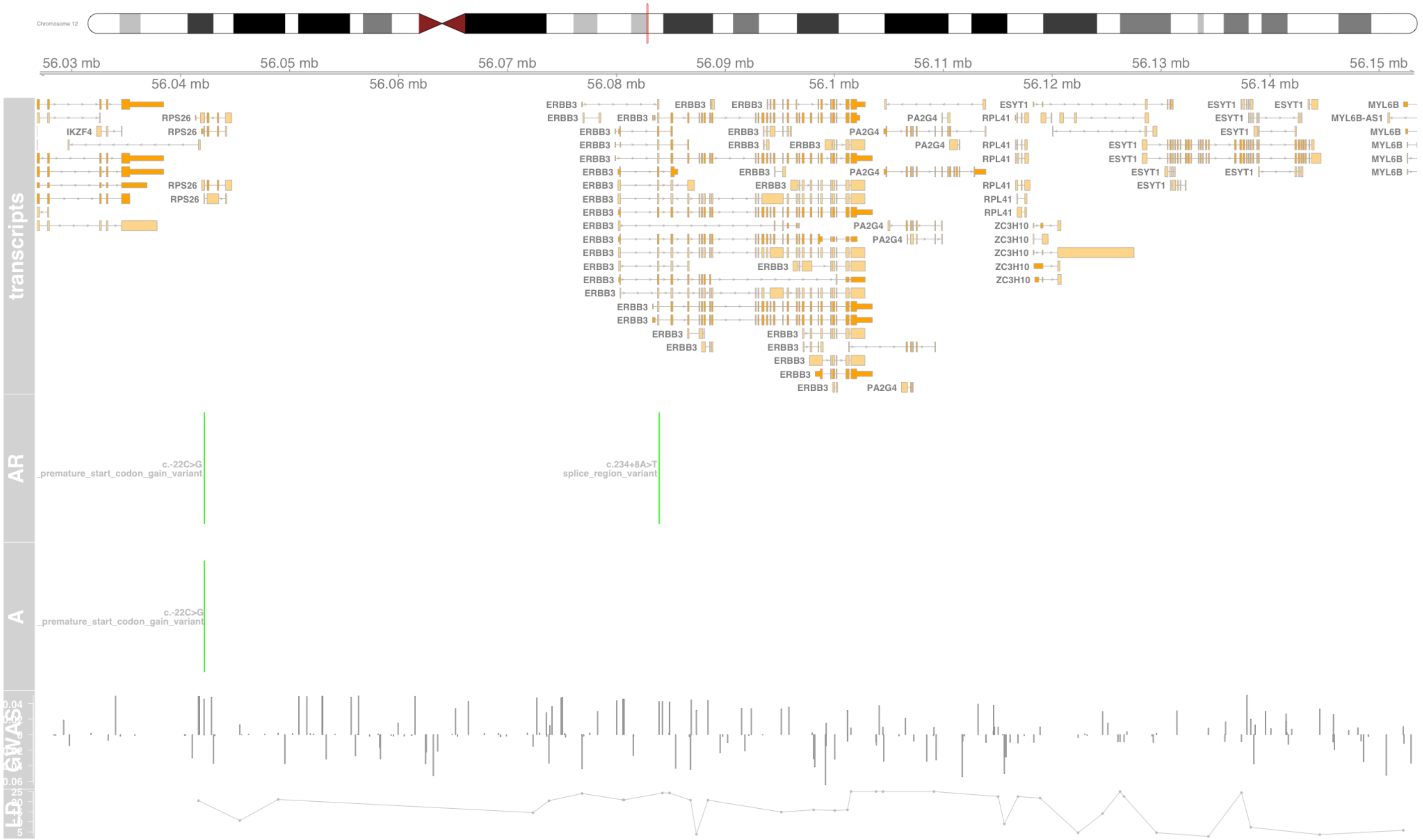

Fig S39 FLG

filaggrin [Source:HGNC Symbol;Acc:HGNC:3748]

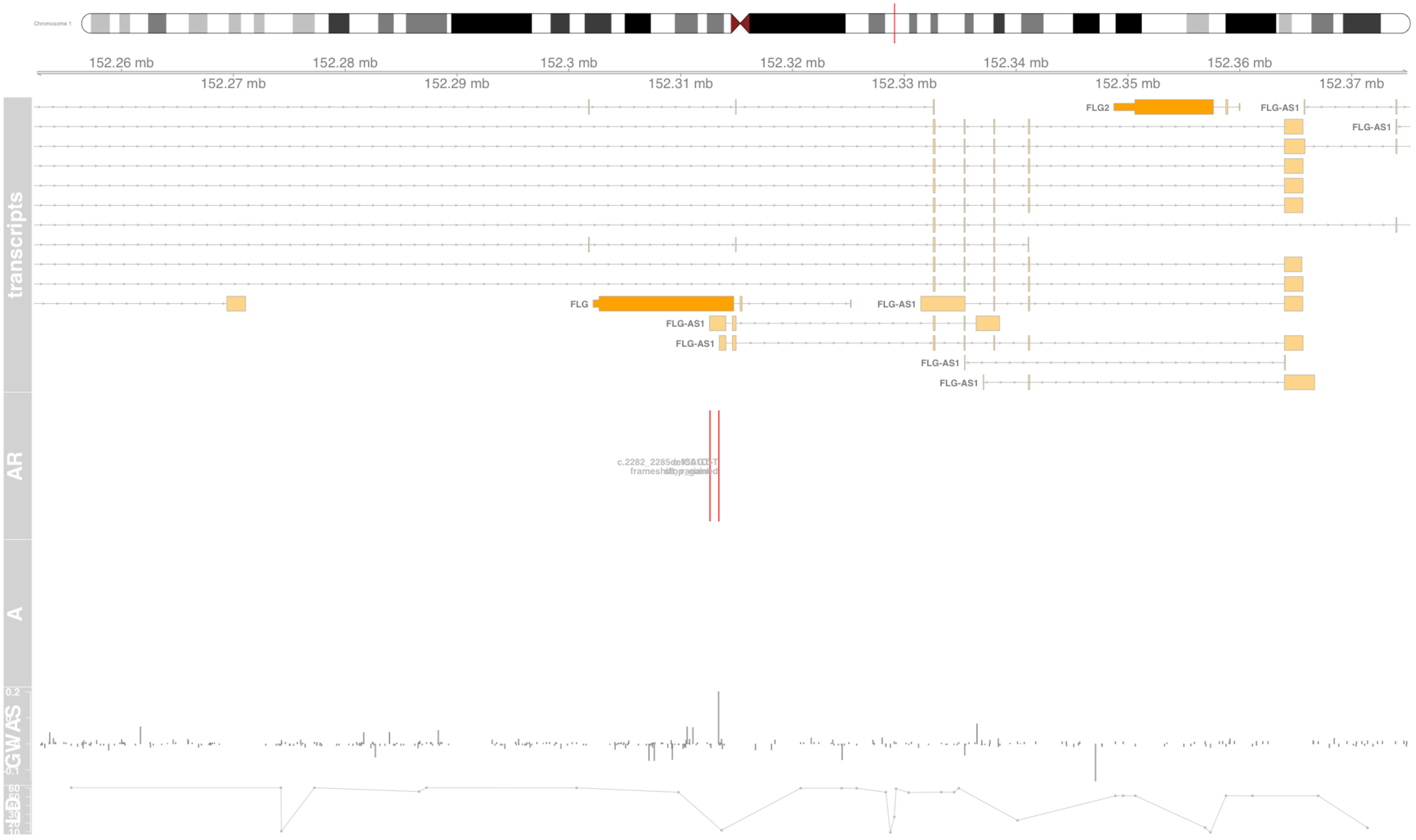

Fig S40 FLOT1

flotillin 1 [Source:HGNC Symbol;Acc:HGNC:3757]

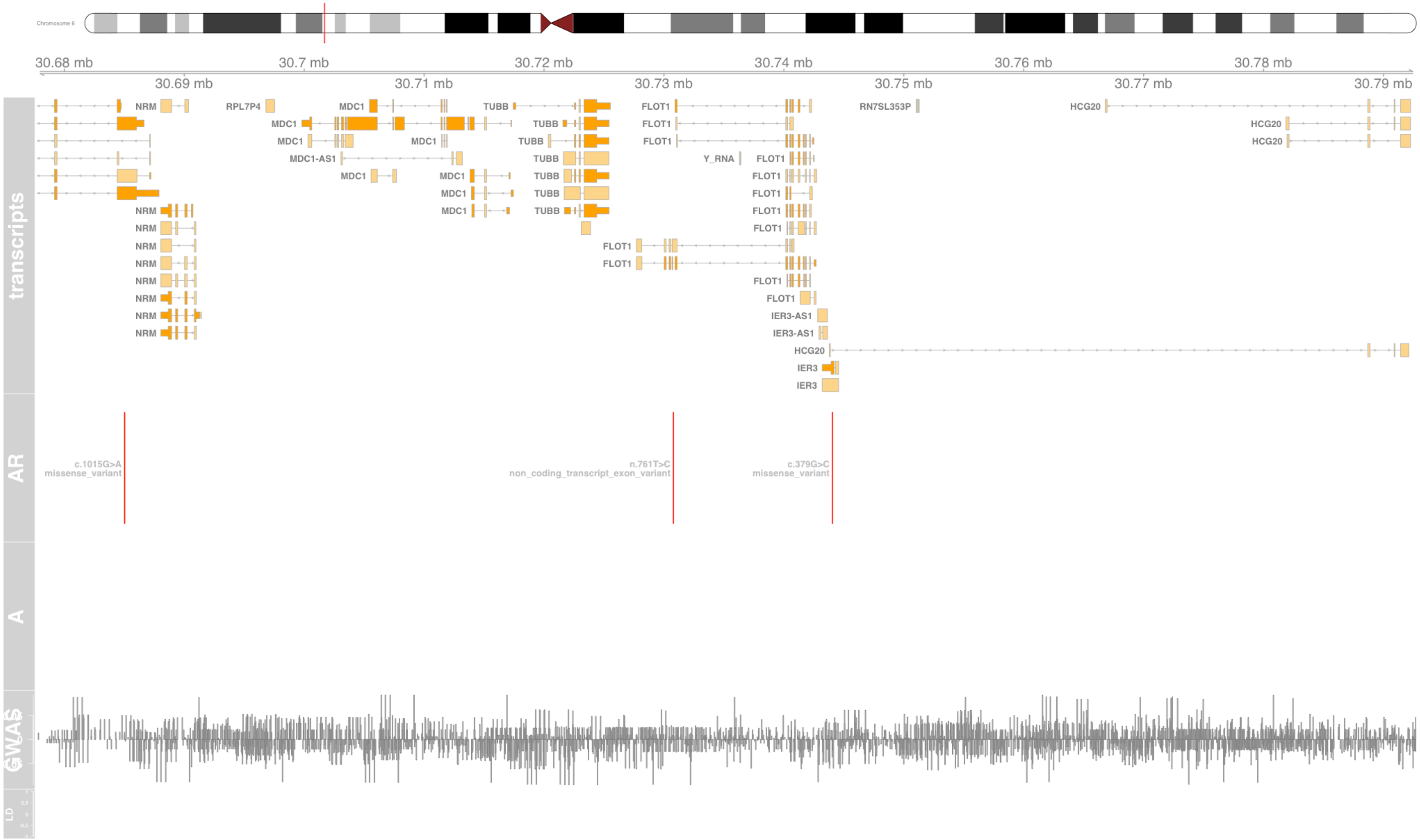

Fig S41 GAL3ST2

galactose-3-O-sulfotransferase 2 [Source:HGNC Symbol;Acc:HGNC:24869]

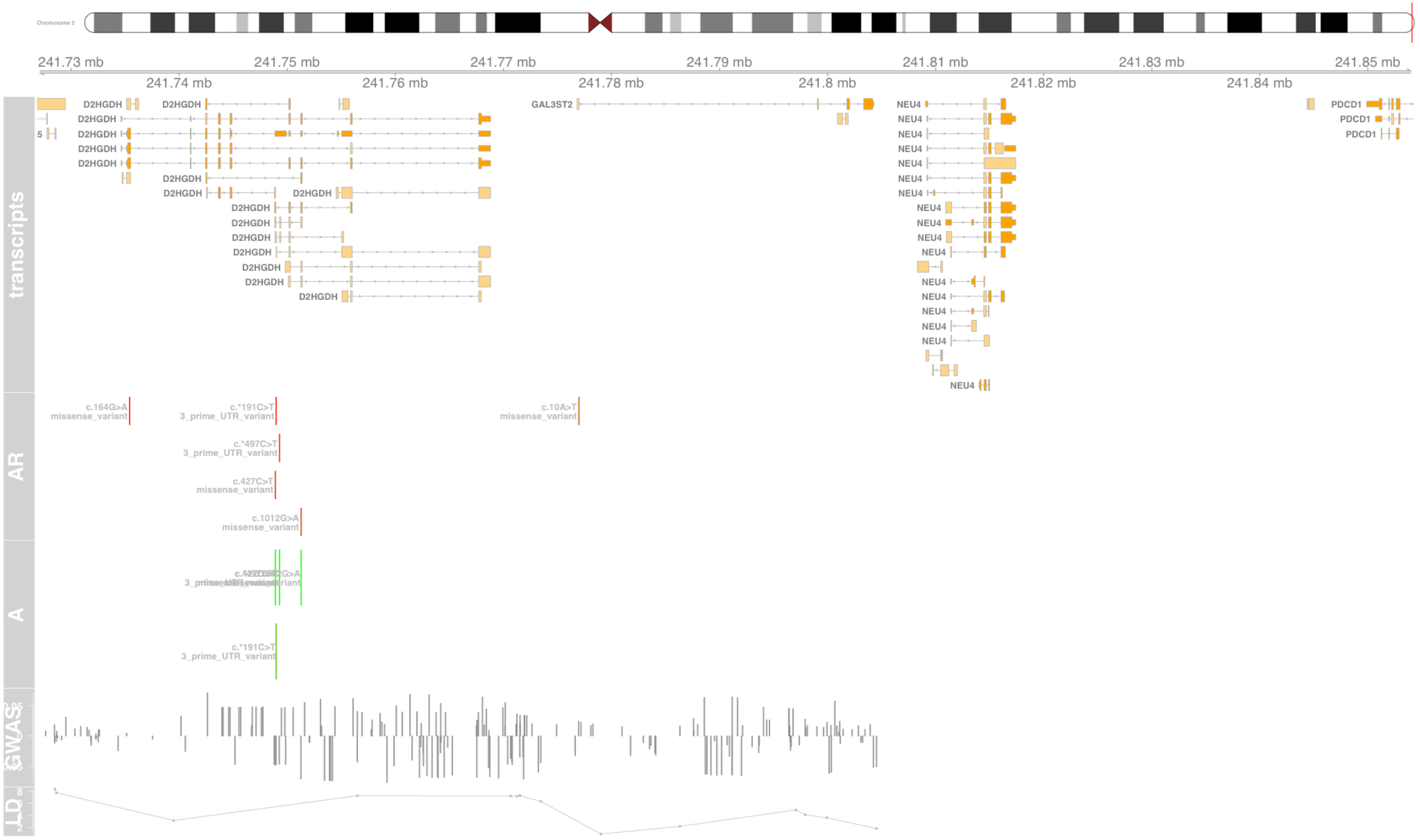

Fig S42 GNL1

G protein nucleolar 1 (putative) [Source:HGNC Symbol;Acc:HGNC:4413]

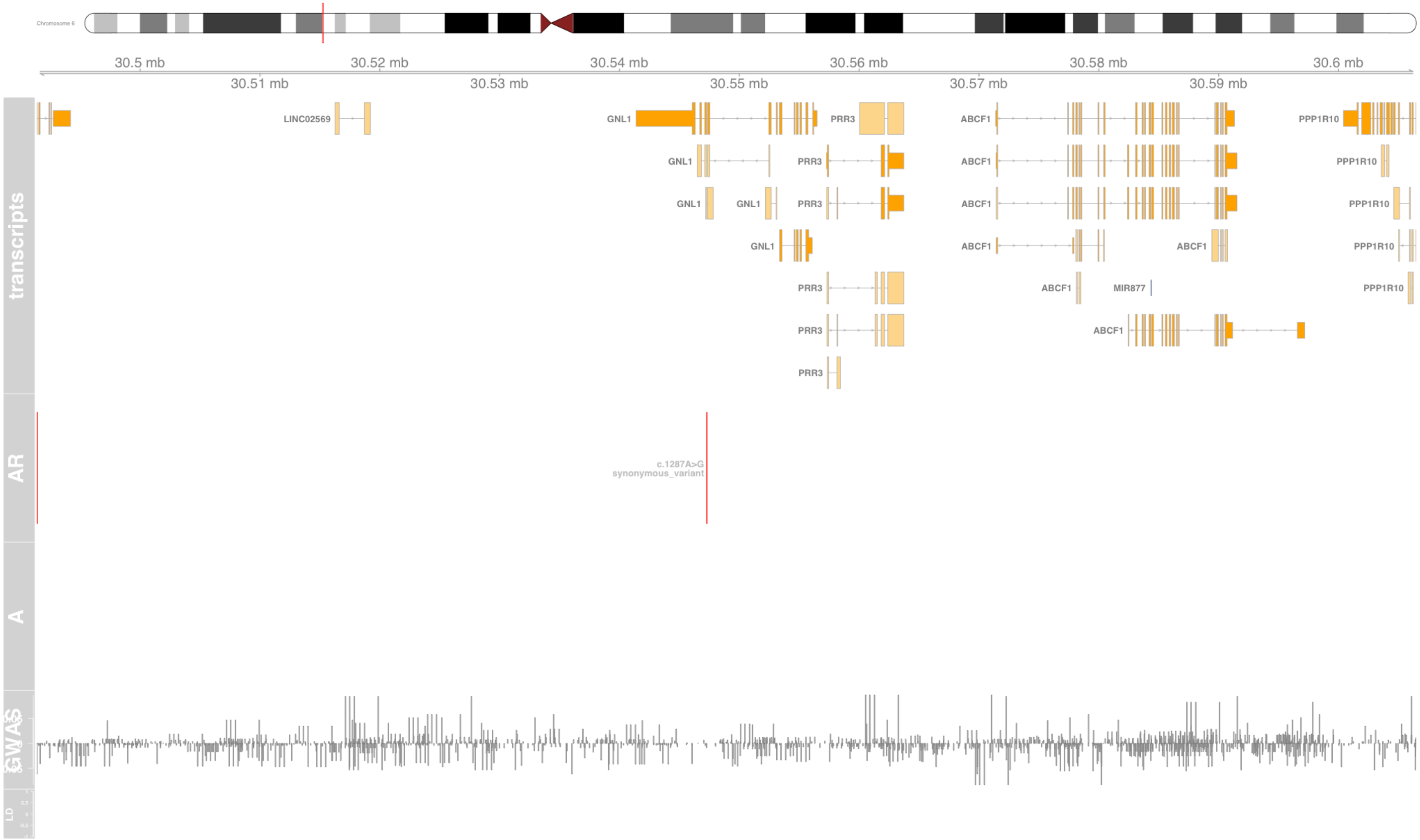

Fig S43 GSDMA

gasdermin A [Source:HGNC Symbol;Acc:HGNC:13311]

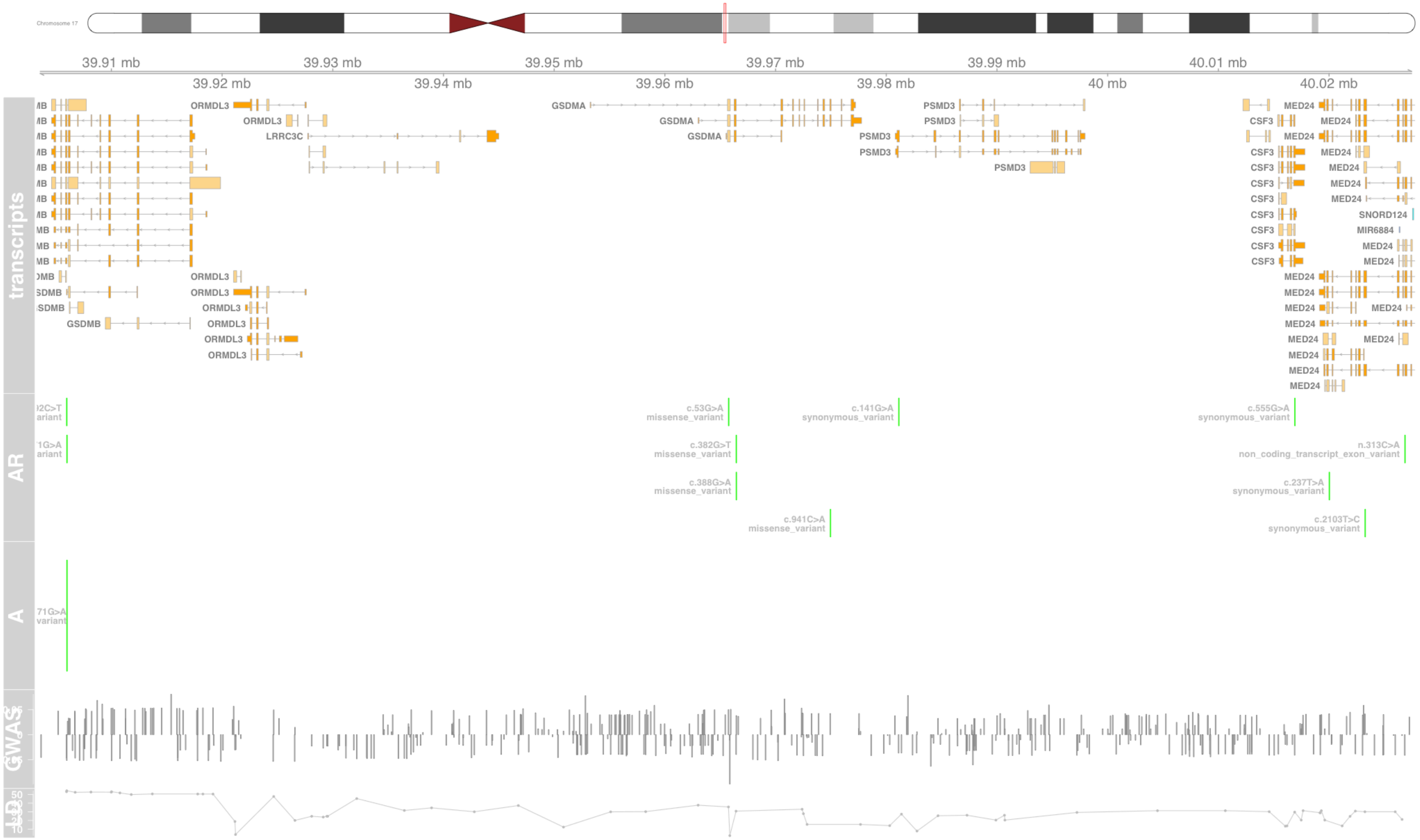

Fig S44 GSDMB

gasdermin B [Source:HGNC Symbol;Acc:HGNC:23690]

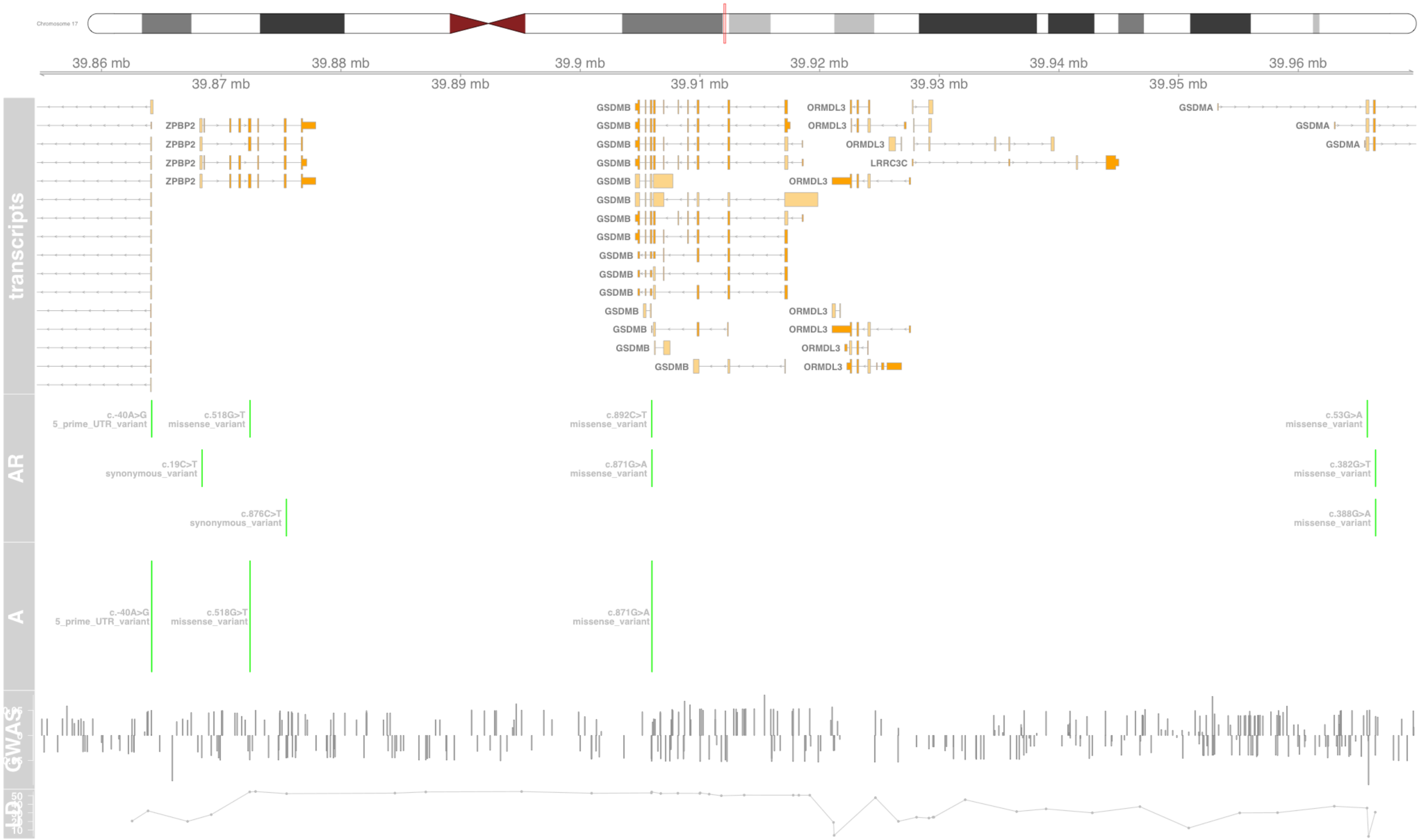

Fig S45 HCG4

HLA complex group 4 [Source:HGNC Symbol;Acc:HGNC:21241]

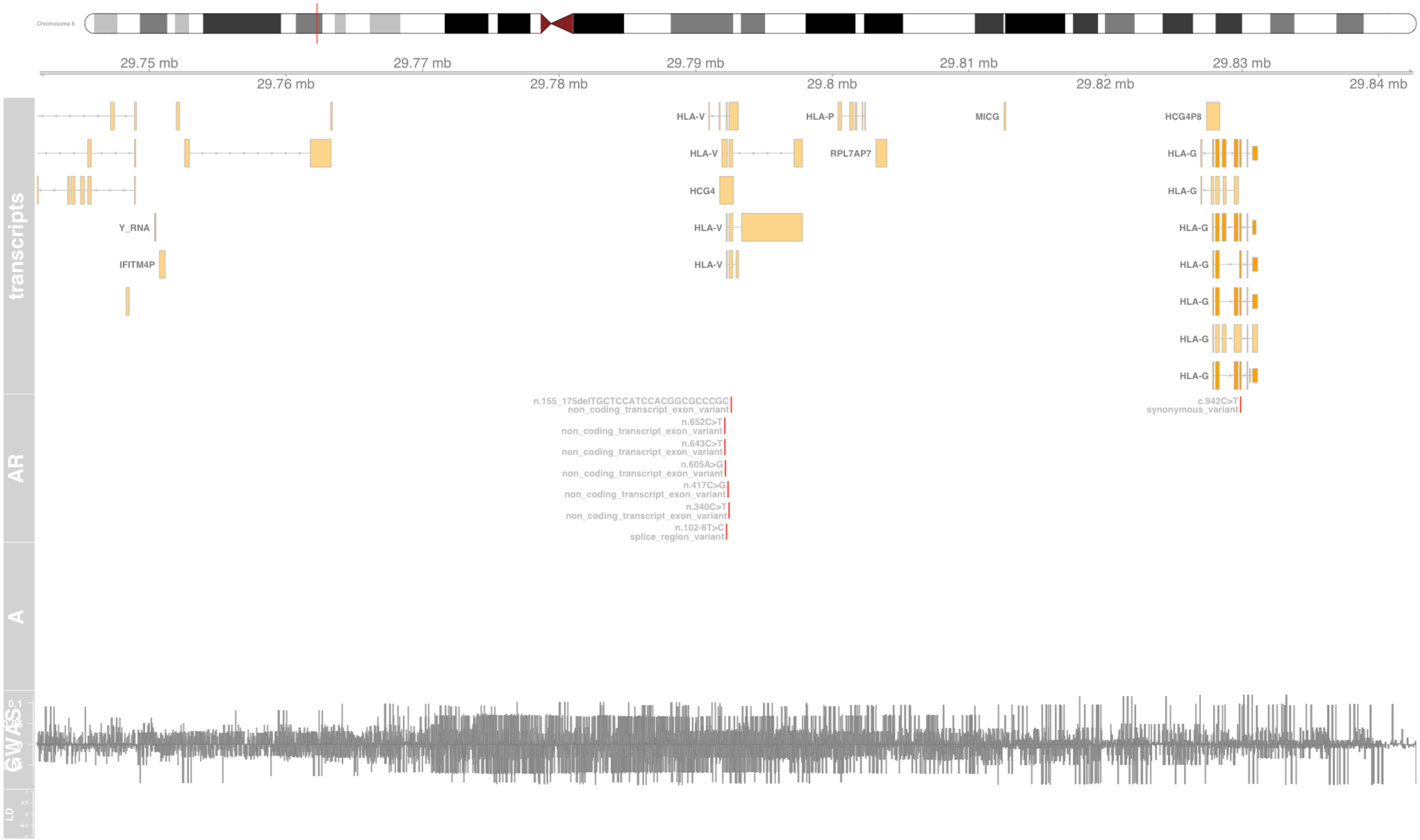

Fig S46 HLA-A

major histocompatibility complex, class I, A [Source:HGNC Symbol;Acc:HGNC:4931]

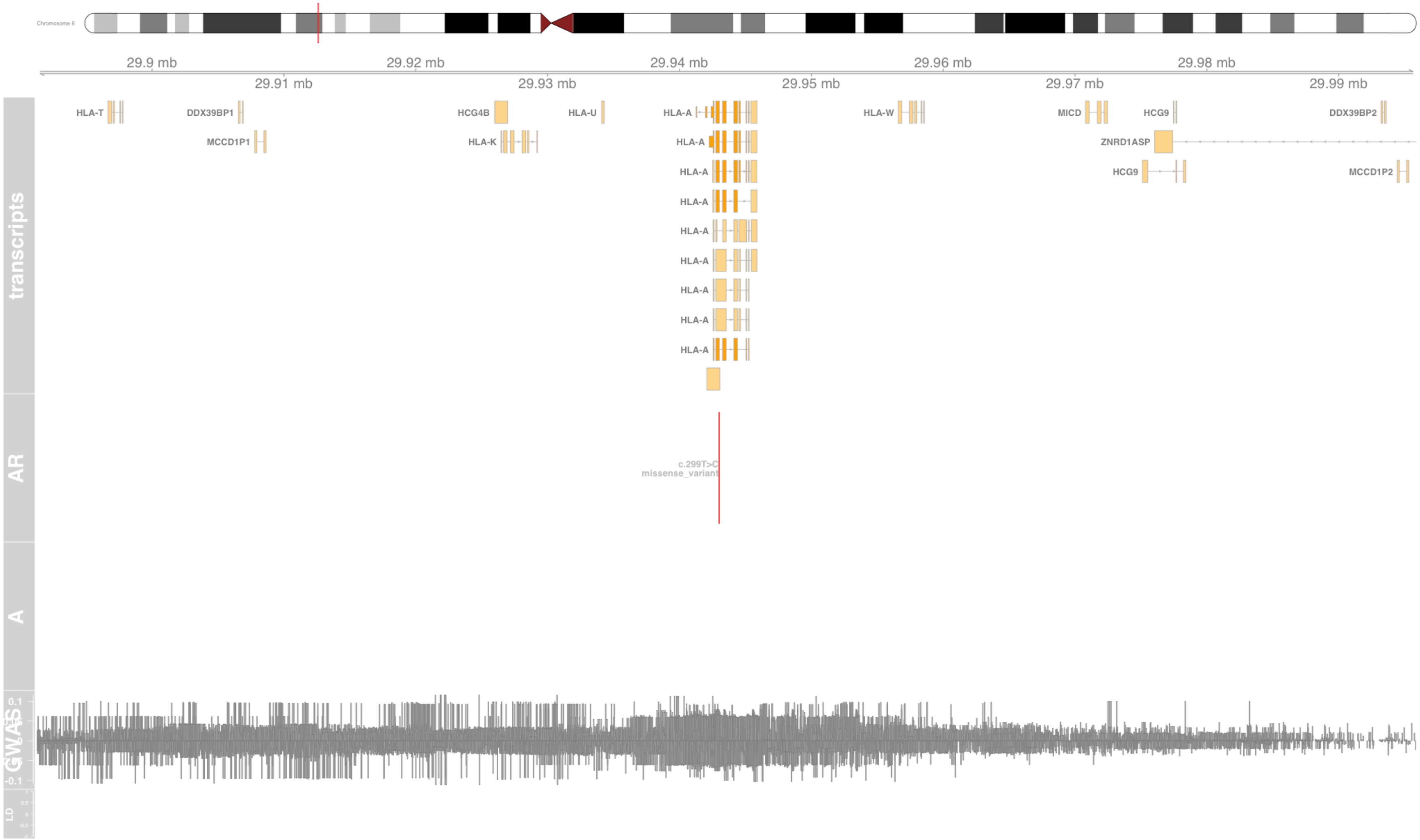

Fig S47 HLA-B

major histocompatibility complex, class I, B [Source:HGNC Symbol;Acc:HGNC:4932]

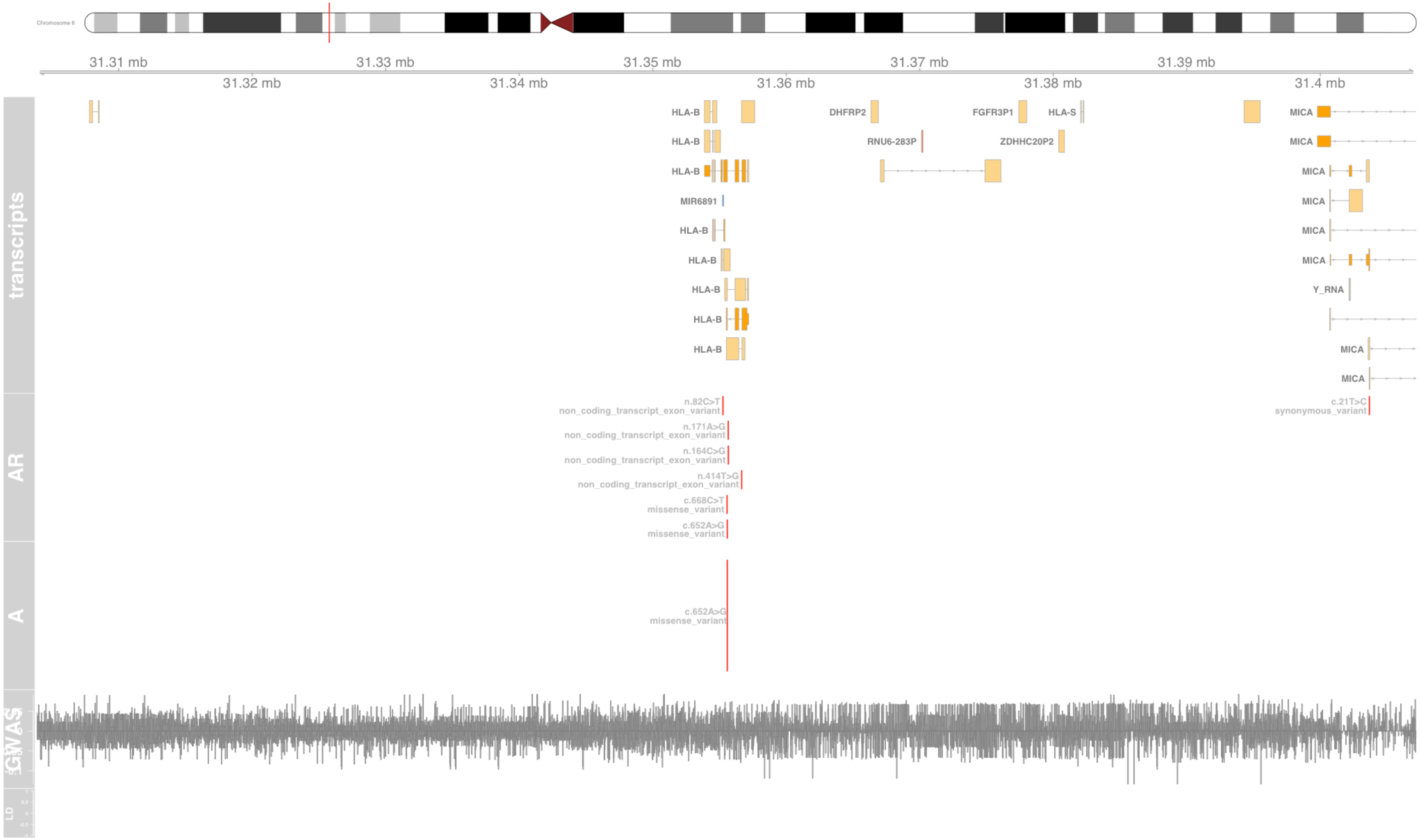

Fig S48 HLA-C

major histocompatibility complex, class I, C [Source:HGNC Symbol;Acc:HGNC:4933]

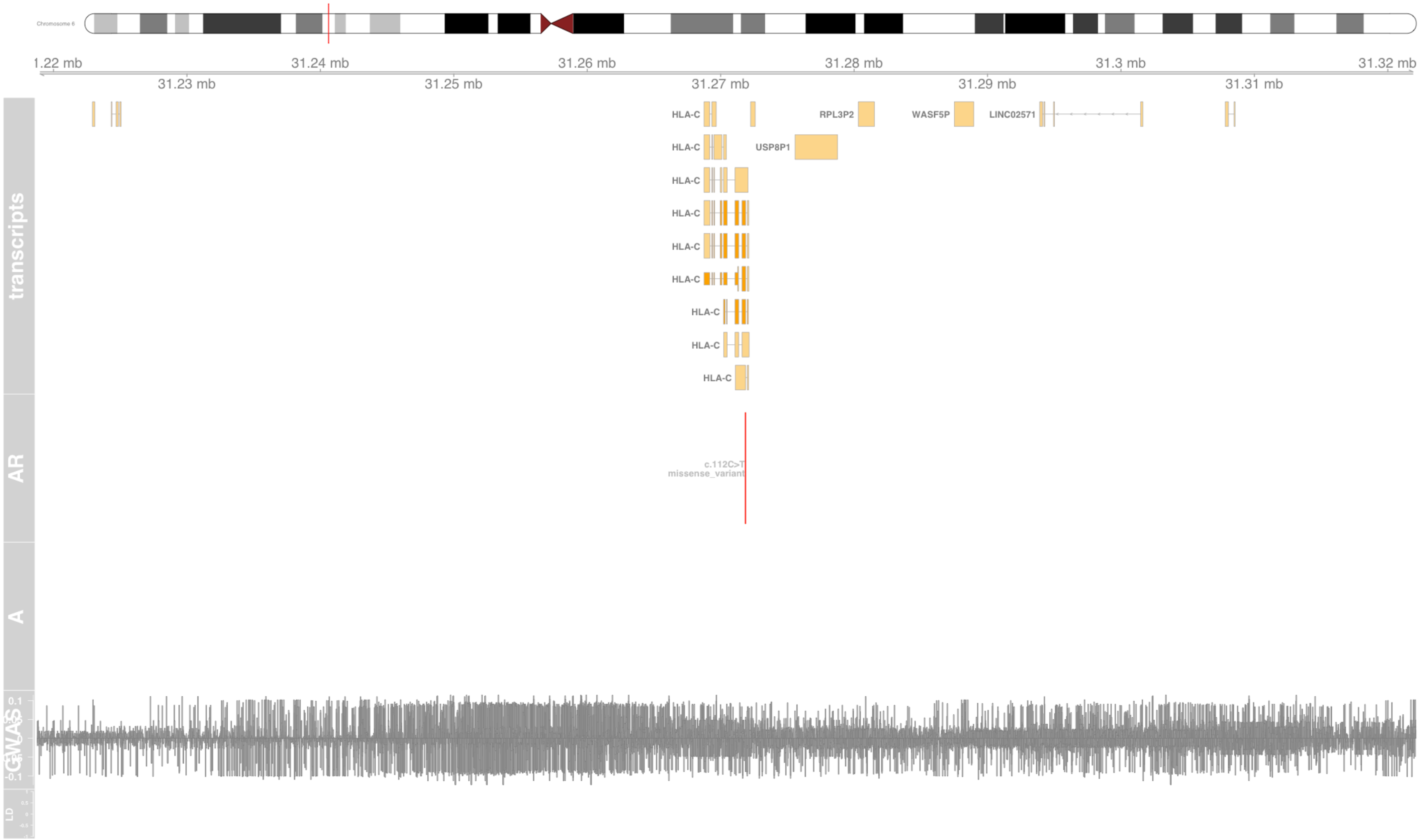

Fig S49 HLA-DOB

major histocompatibility complex, class II, DO beta [Source:HGNC Symbol;Acc:HGNC:4937]

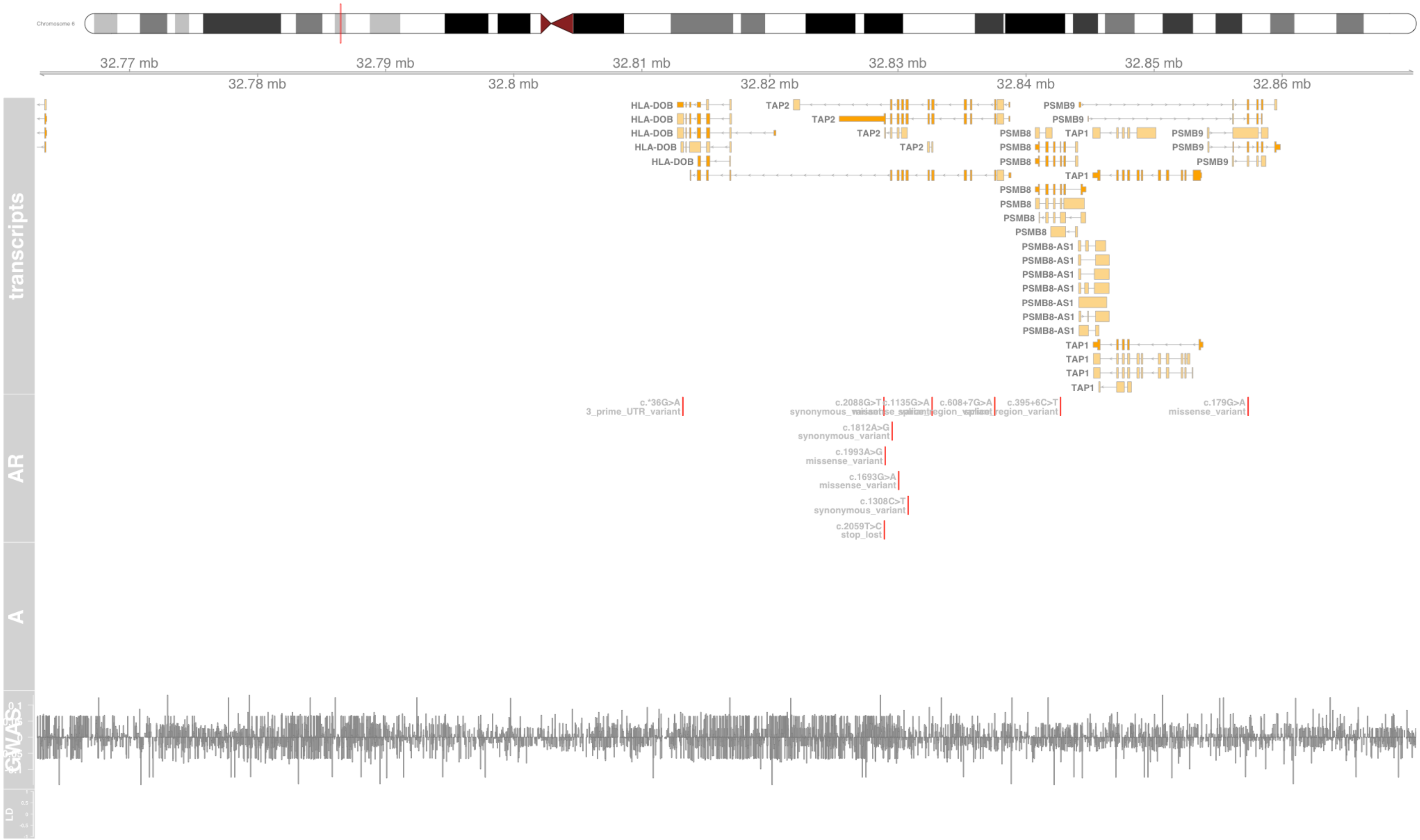

Fig S50 HLA-DQA1

major histocompatibility complex, class II, DQ alpha 1 [Source:HGNC Symbol;Acc:HGNC:4942]

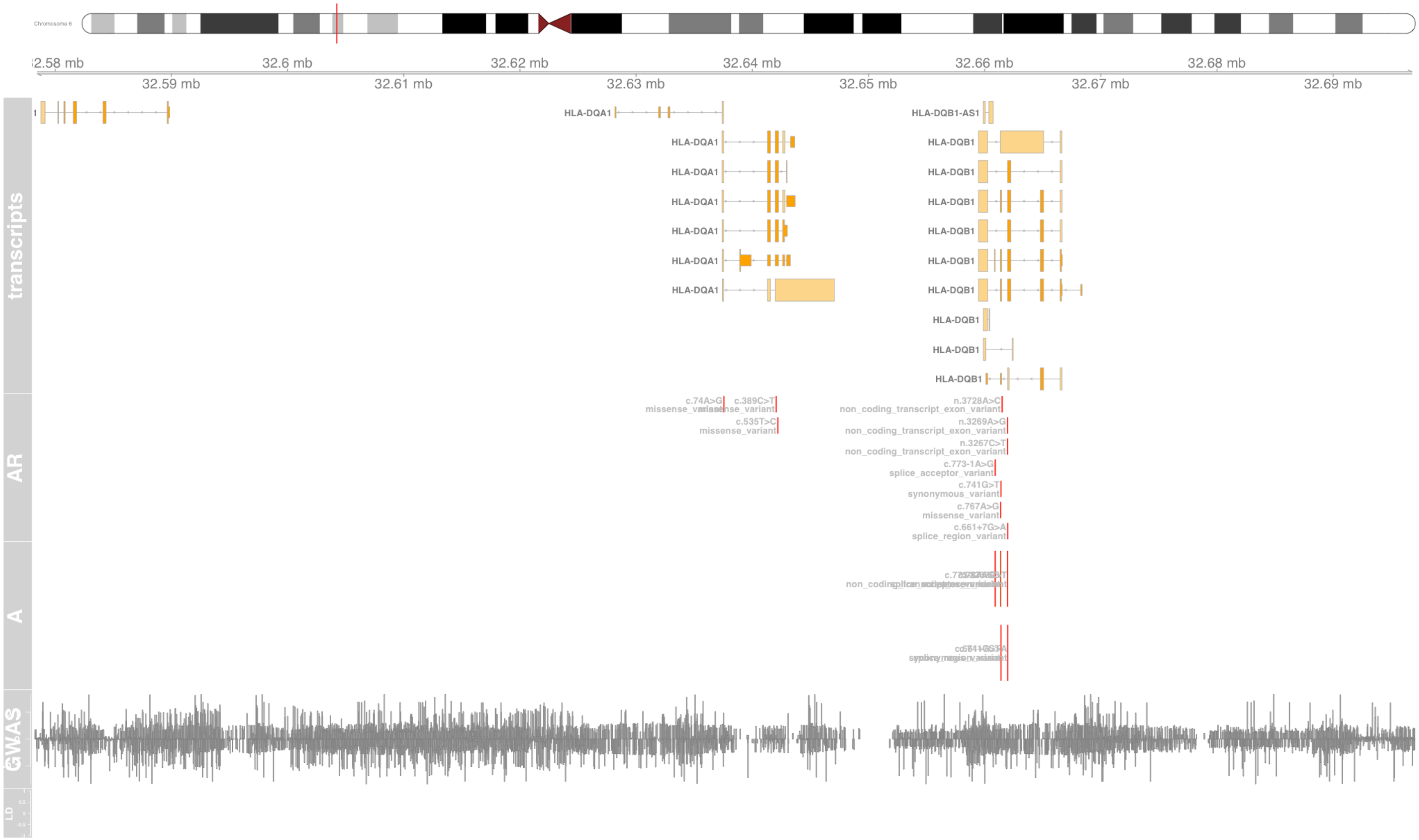

Fig S51 HLA-DQA2

major histocompatibility complex, class II, DQ alpha 2 [Source:HGNC Symbol;Acc:HGNC:4943]

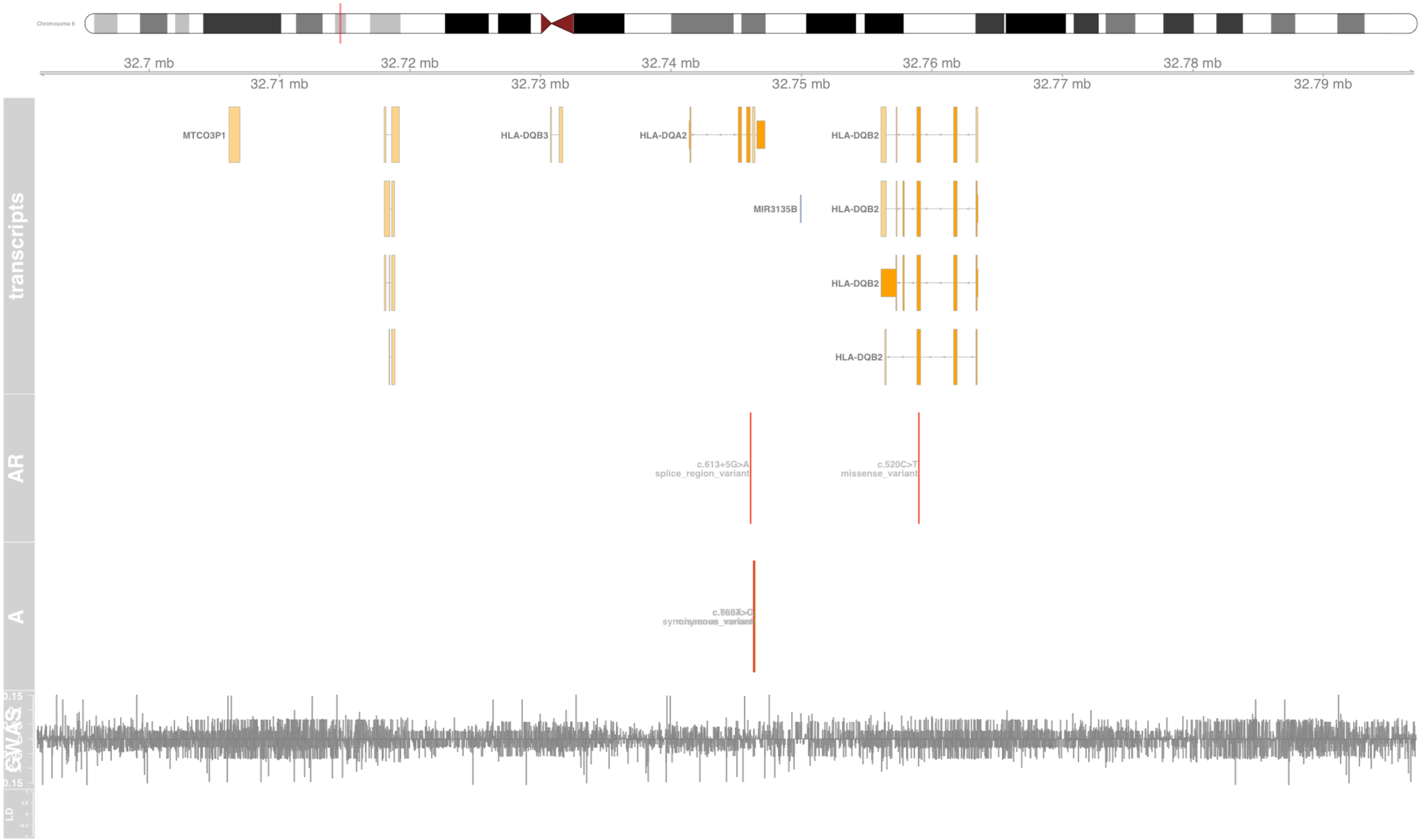

Fig S52 HLA-DQB1

major histocompatibility complex, class II, DQ beta 1 [Source:HGNC Symbol;Acc:HGNC:4944]

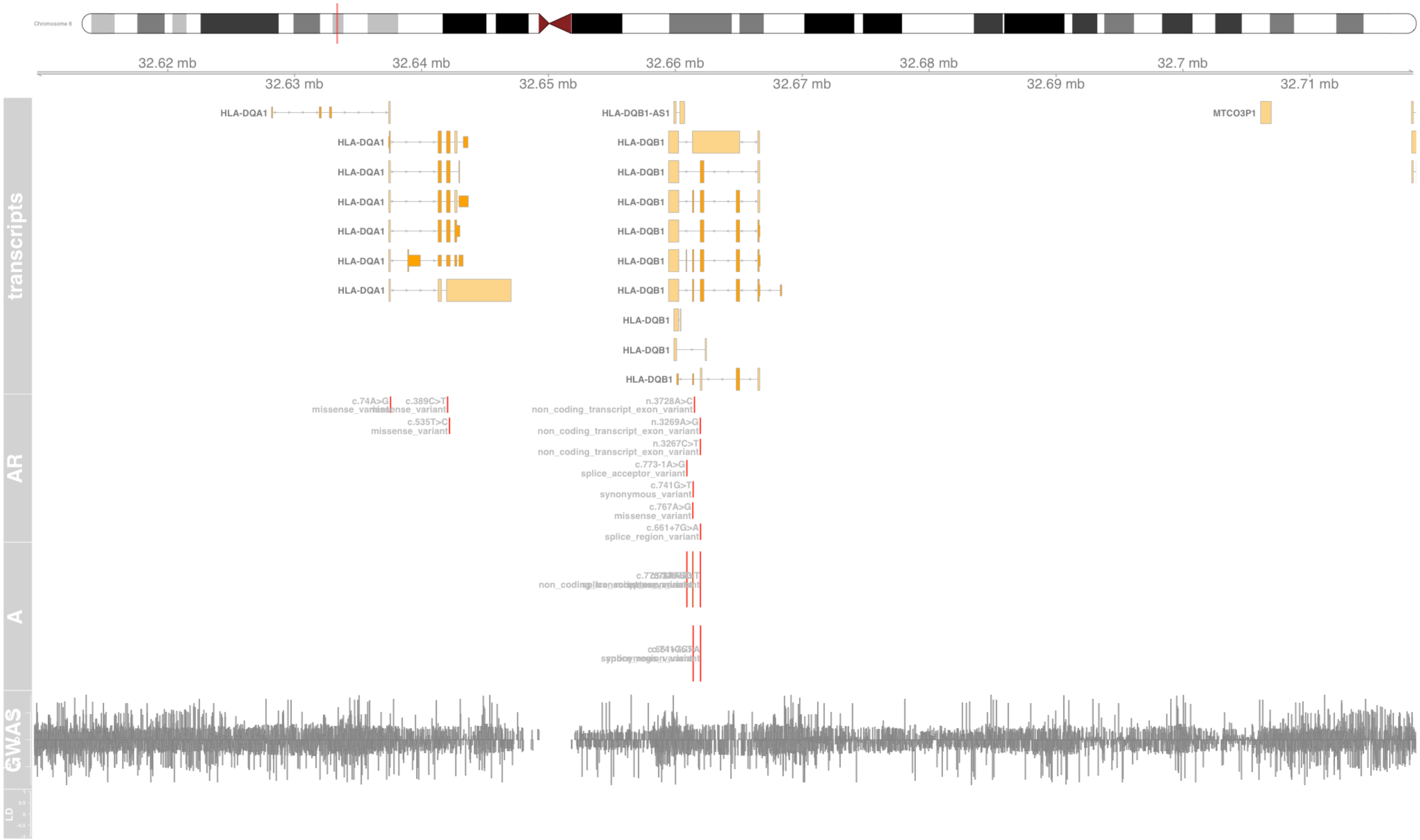

Fig S53 HLA-DQB2

major histocompatibility complex, class II, DQ beta 2 [Source:HGNC Symbol;Acc:HGNC:4945]

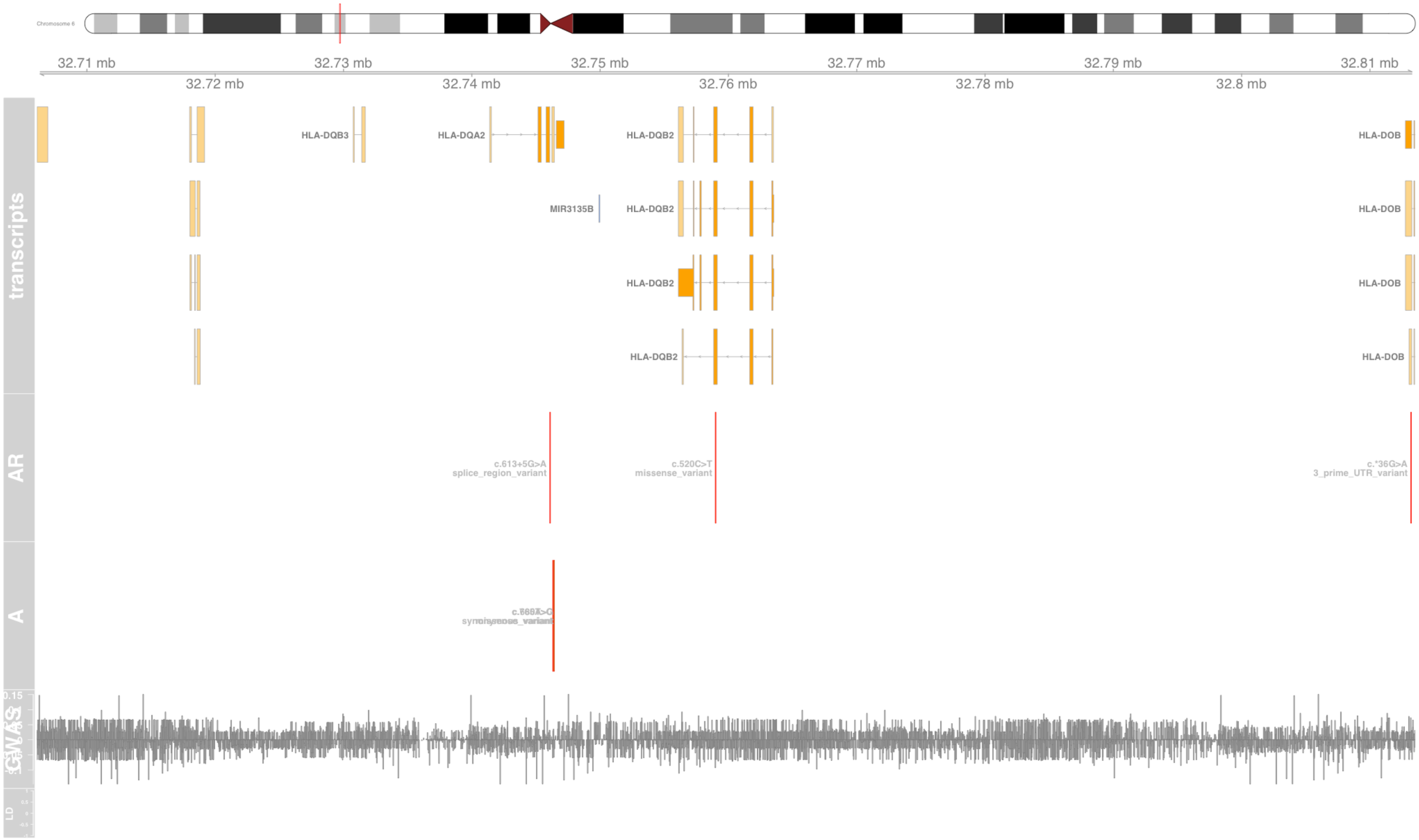

Fig S54 HLA-DRA

major histocompatibility complex, class II, DR alpha [Source:HGNC Symbol;Acc:HGNC:4947]

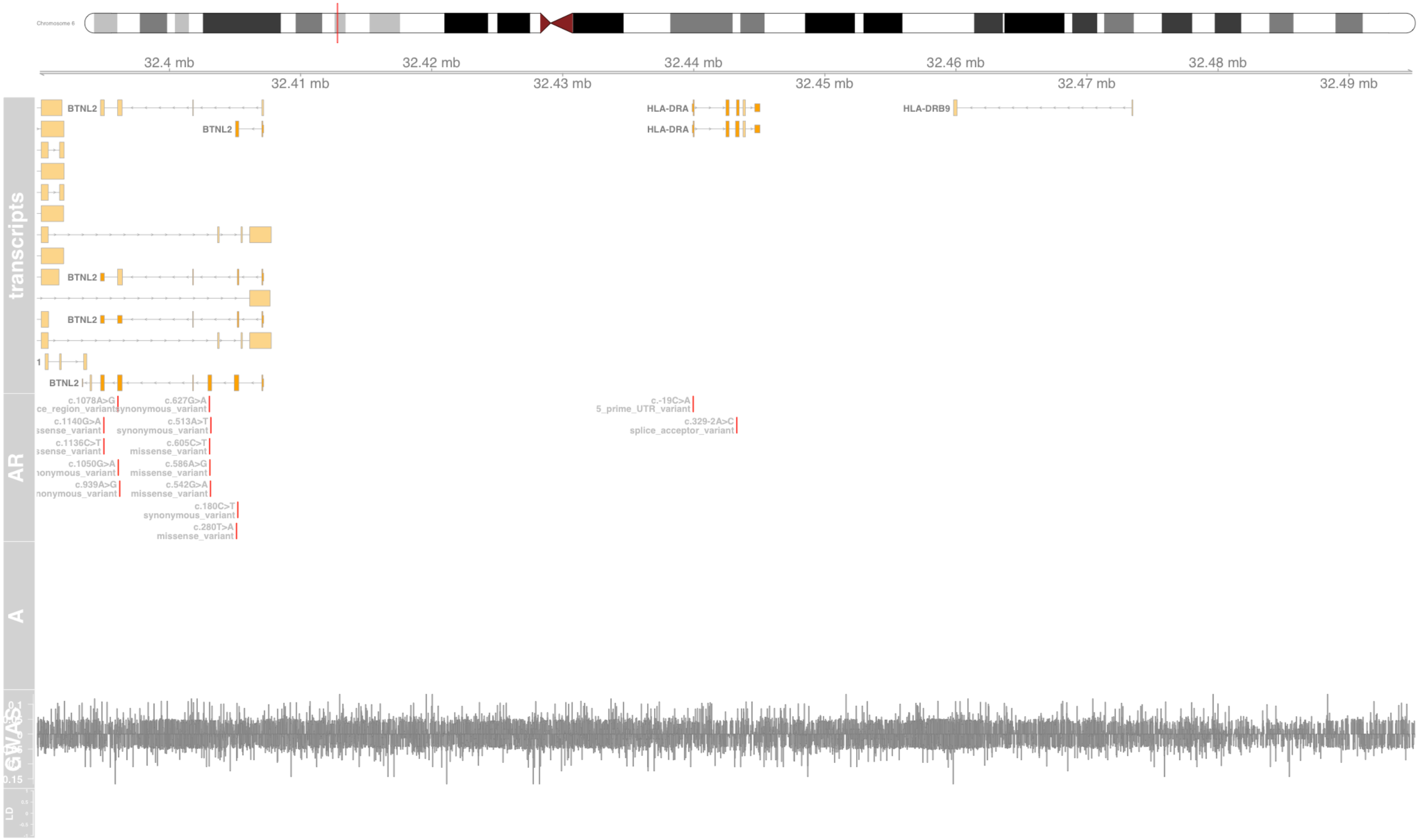

Fig S55 HLA-E

major histocompatibility complex, class I, E [Source:HGNC Symbol;Acc:HGNC:4962]

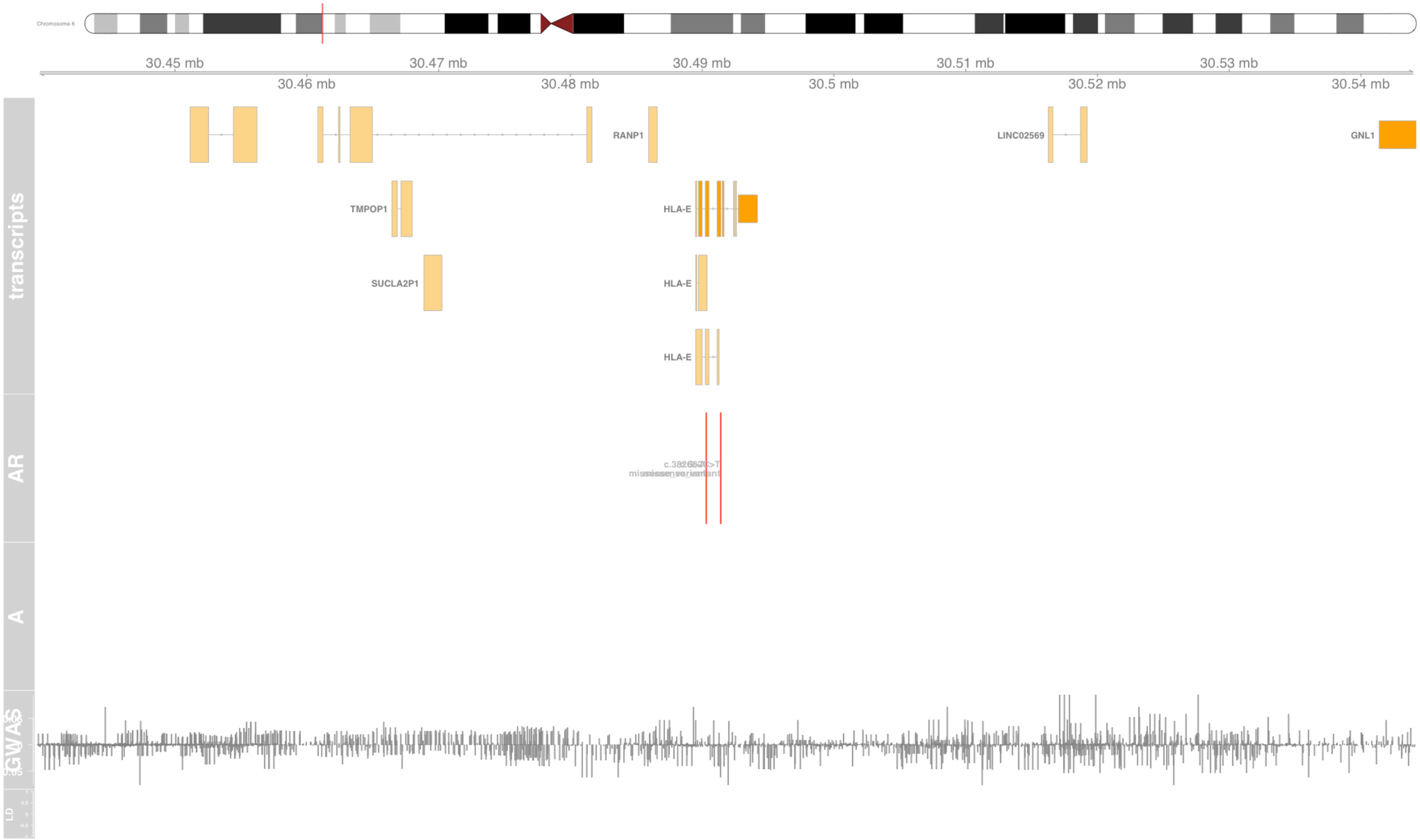

Fig S56 HLA-G

major histocompatibility complex, class I, G [Source:HGNC Symbol;Acc:HGNC:4964]

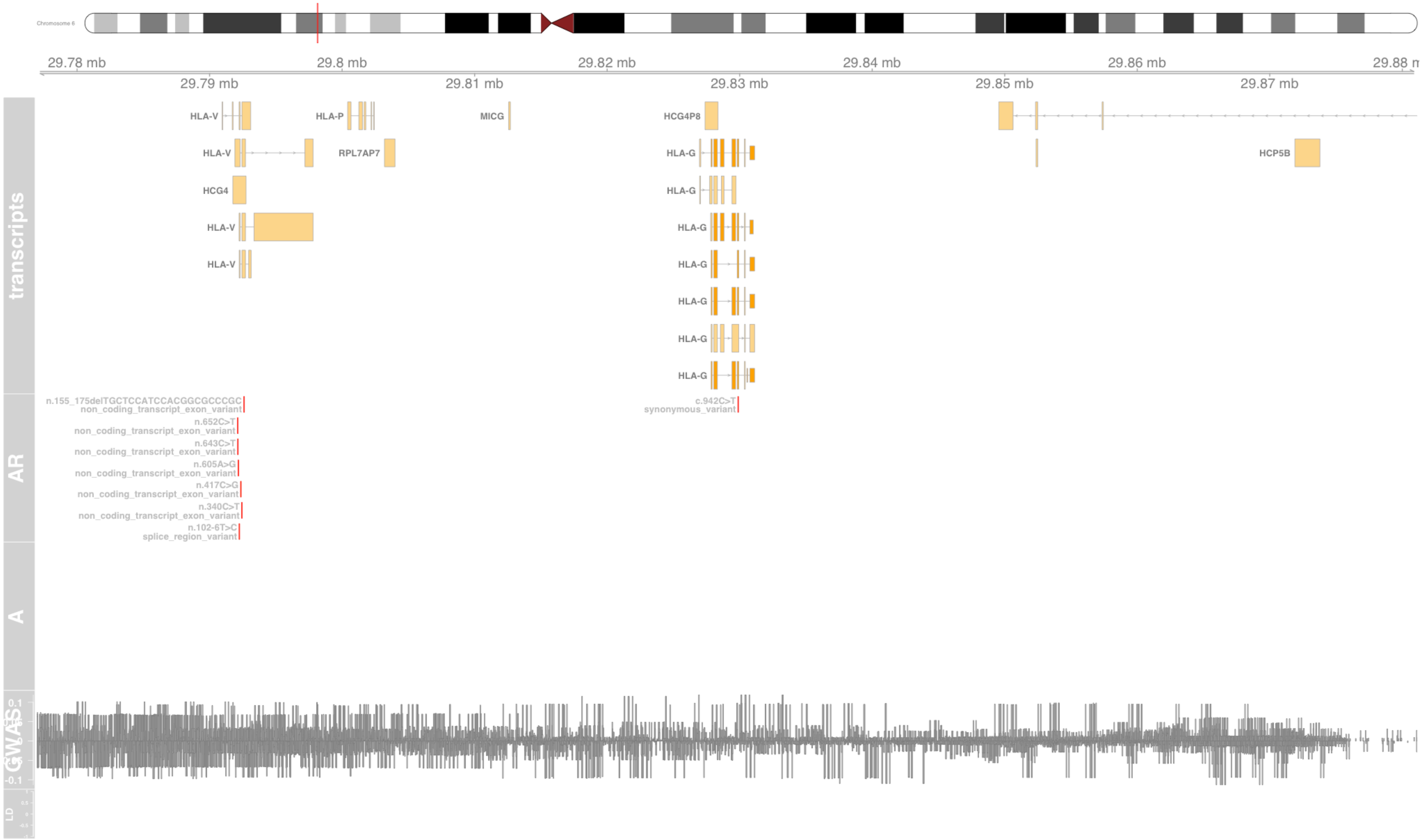

Fig S57 HLA-H

major histocompatibility complex, class I, H (pseudogene) [Source:HGNC Symbol;Acc:HGNC:4965]

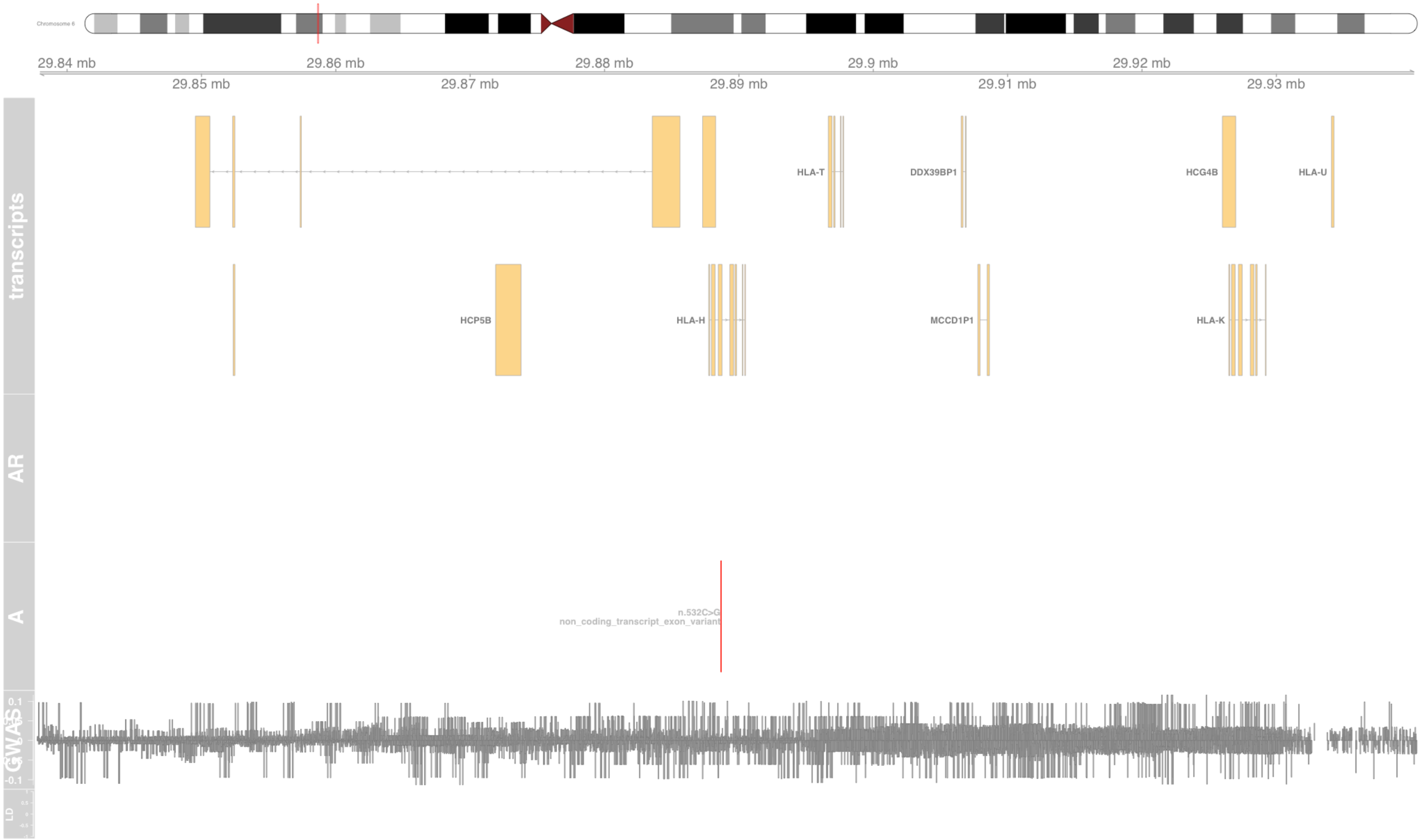

Fig S58 HLA-V

major histocompatibility complex, class I, V (pseudogene) [Source:HGNC Symbol;Acc:HGNC:23482]

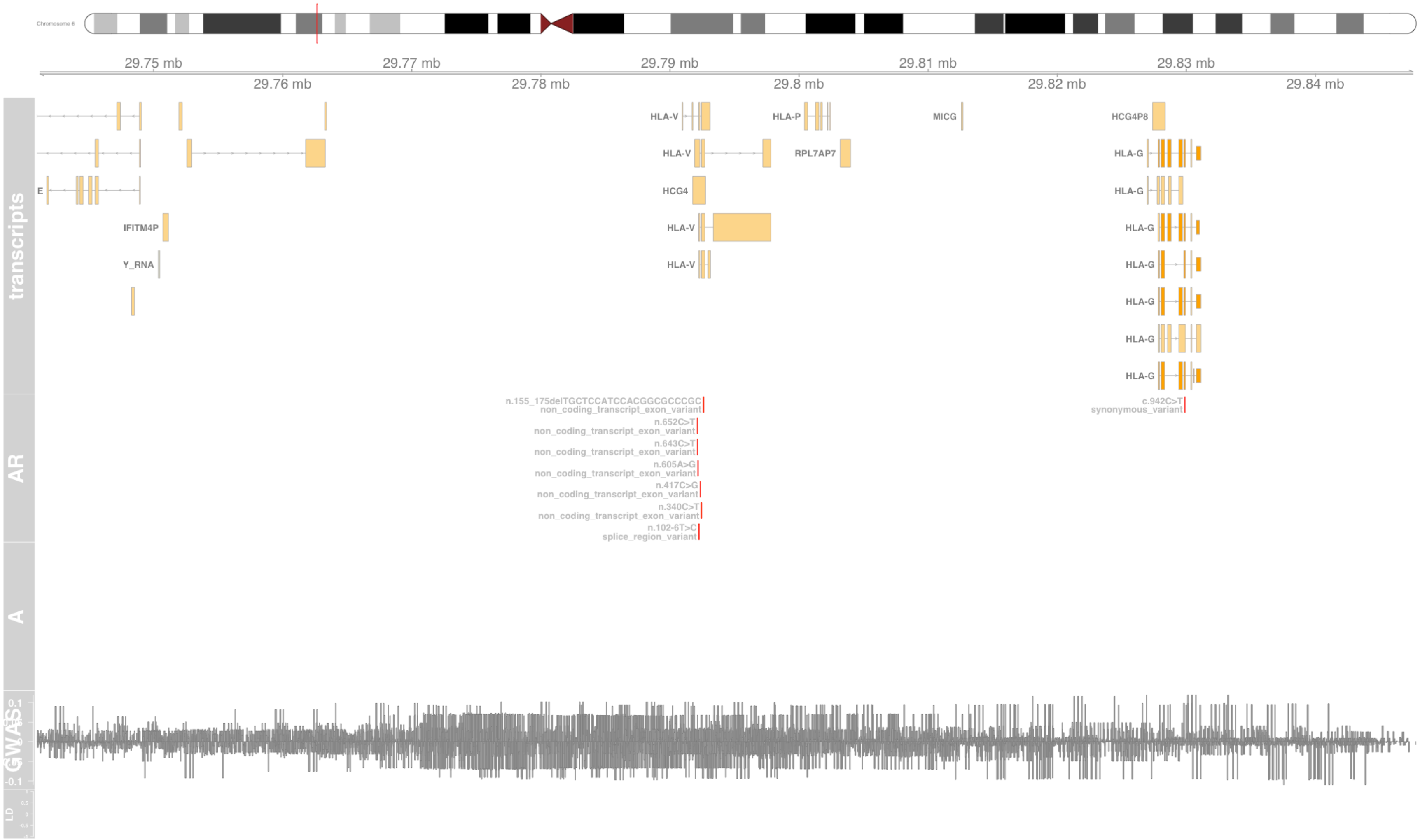

Fig S59 HSPA1L

heat shock protein family A (Hsp70) member 1 like [Source:HGNC Symbol;Acc:HGNC:5234]

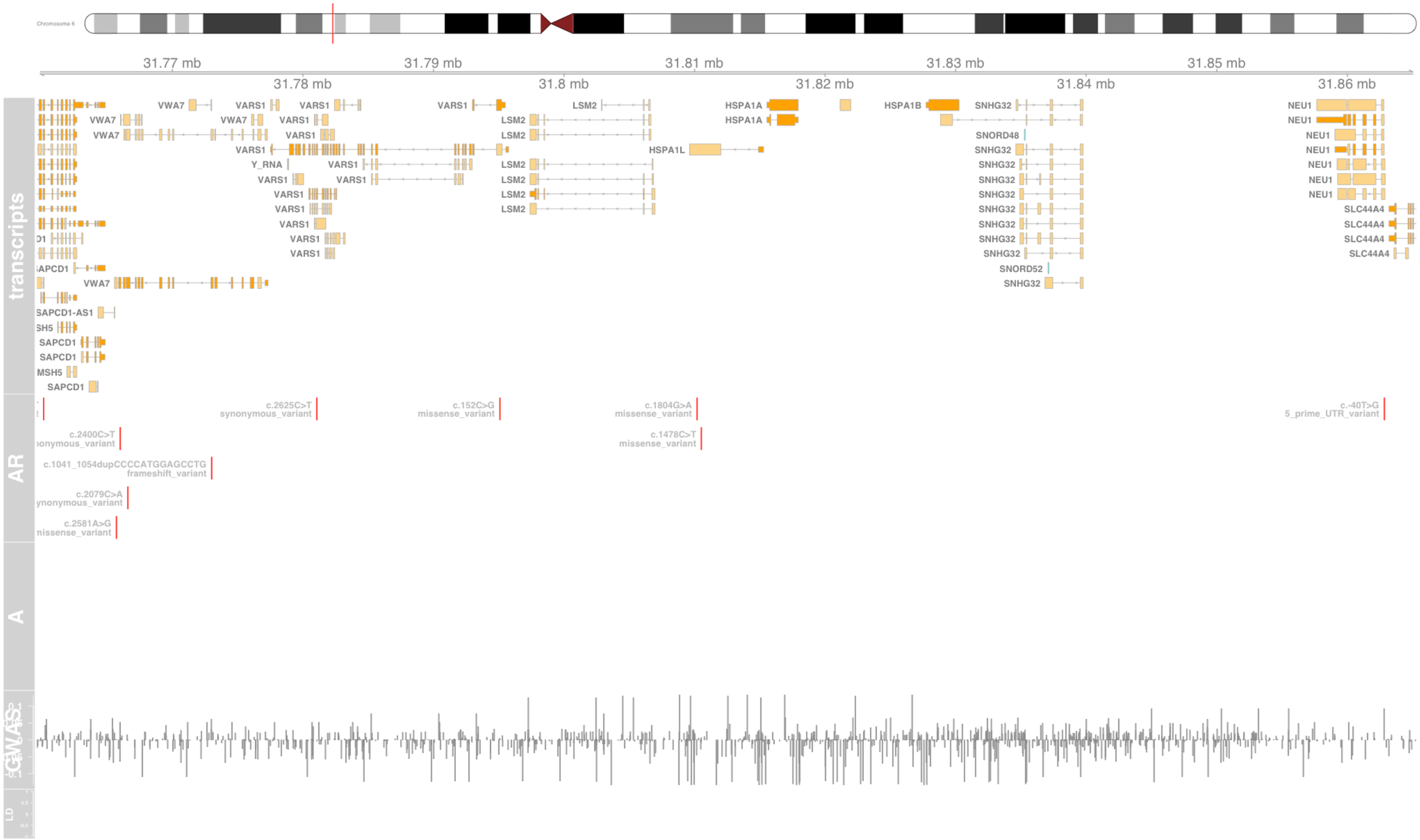

Fig S60 IER3

immediate early response 3 [Source:HGNC Symbol;Acc:HGNC:5392]

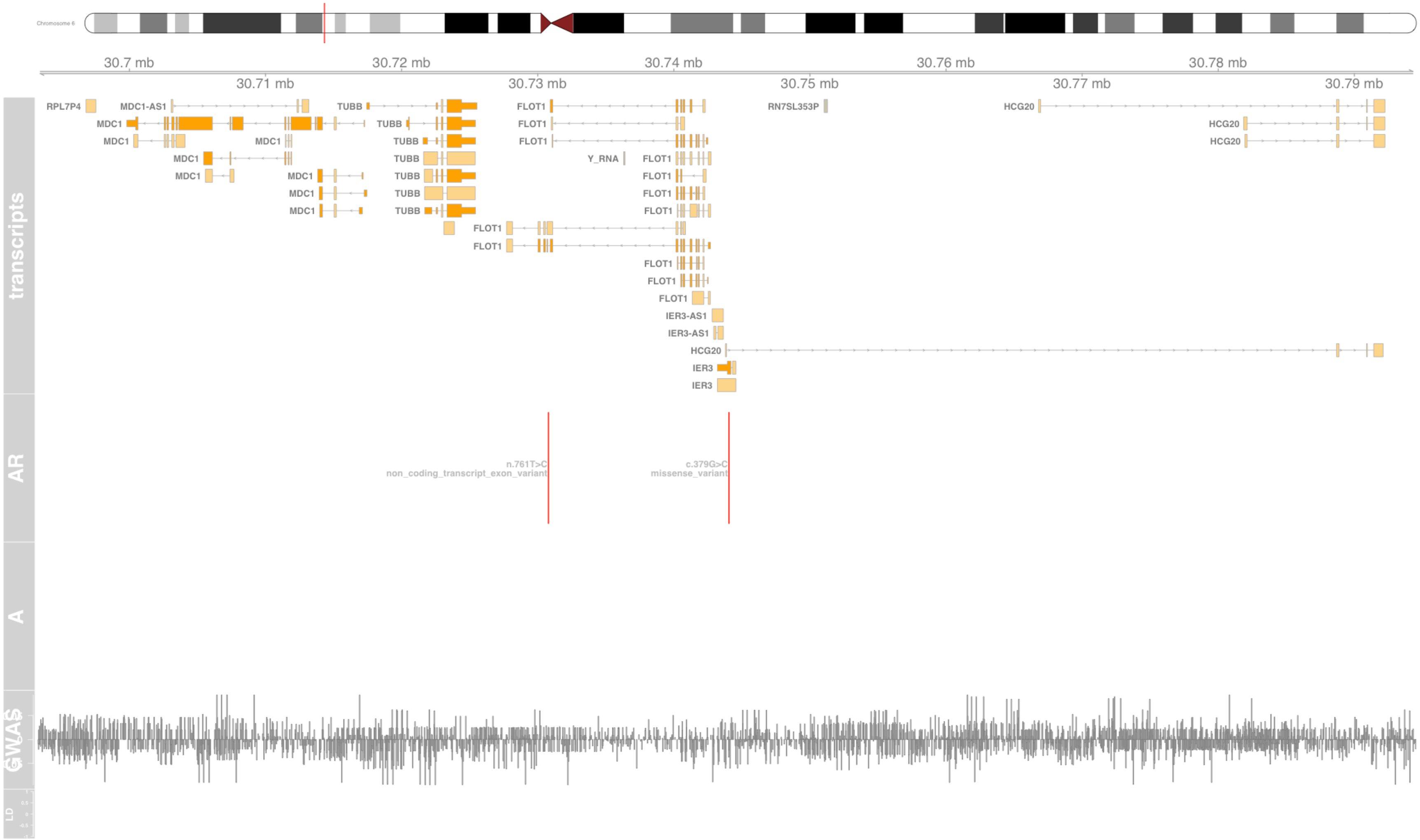

Fig S61 IKZF3

IKAROS family zinc finger 3 [Source:HGNC Symbol;Acc:HGNC:13178]

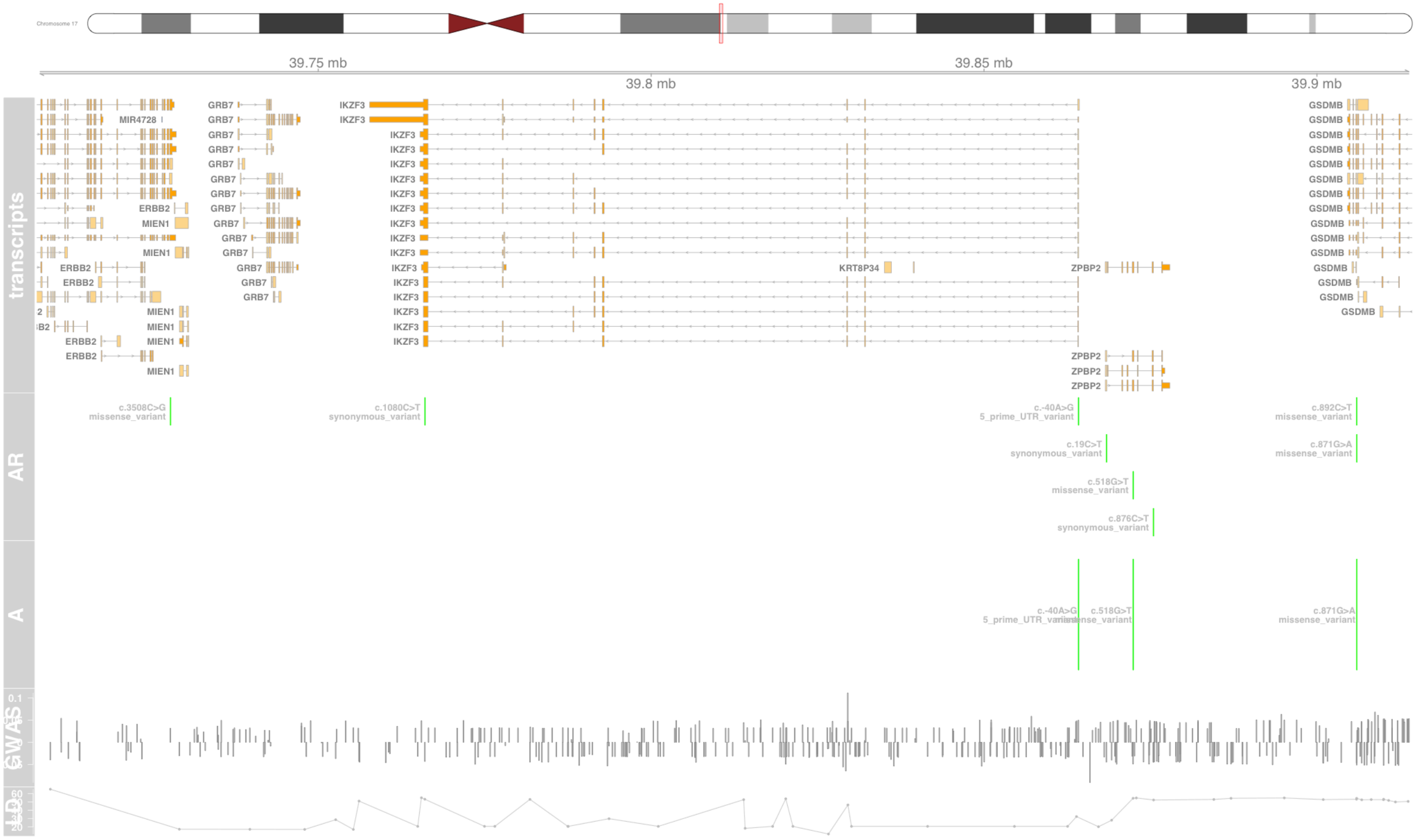

Fig S62 IL13

interleukin 13 [Source:HGNC Symbol;Acc:HGNC:5973]

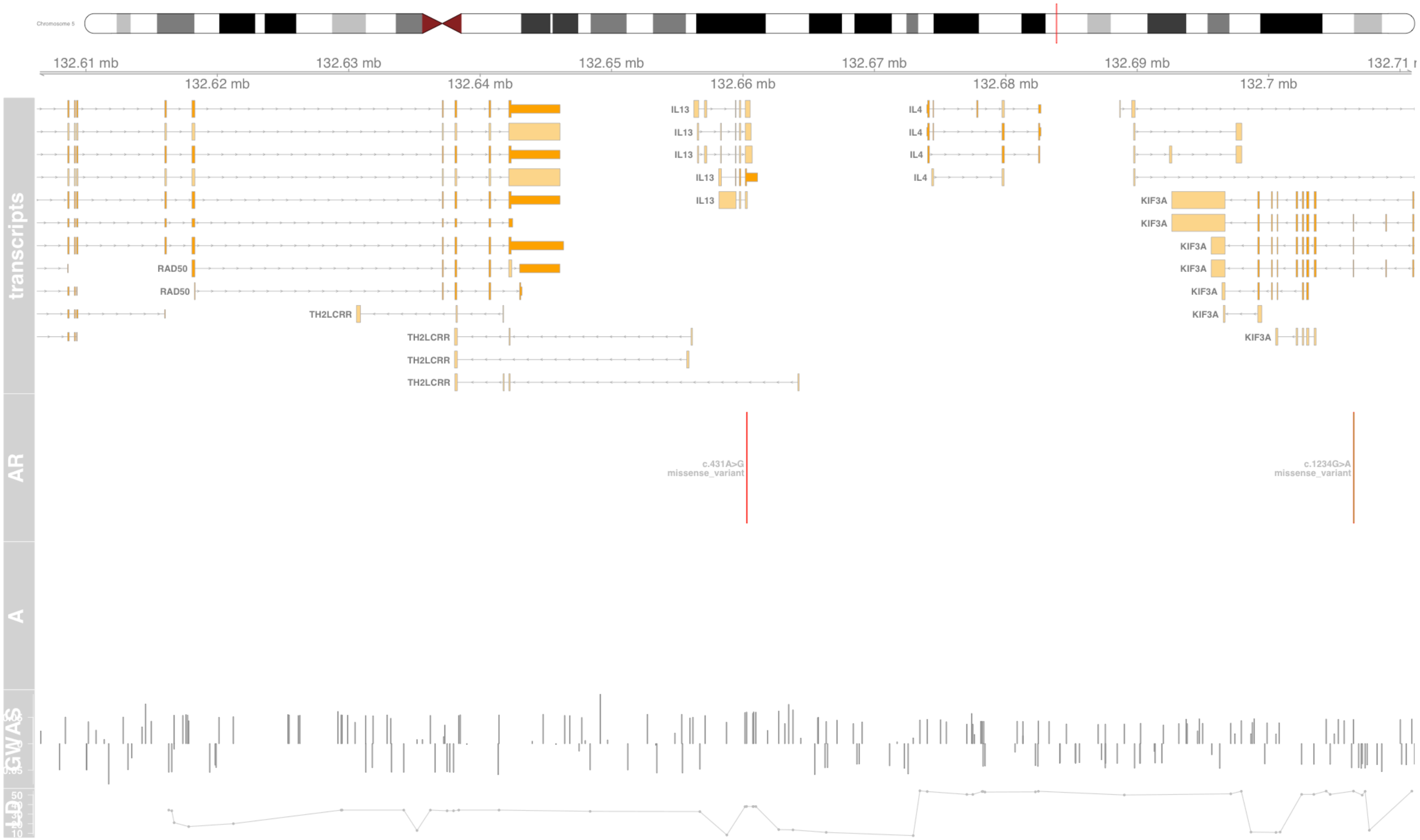

Fig S63 IL18R1

interleukin 18 receptor 1 [Source:HGNC Symbol;Acc:HGNC:5988]

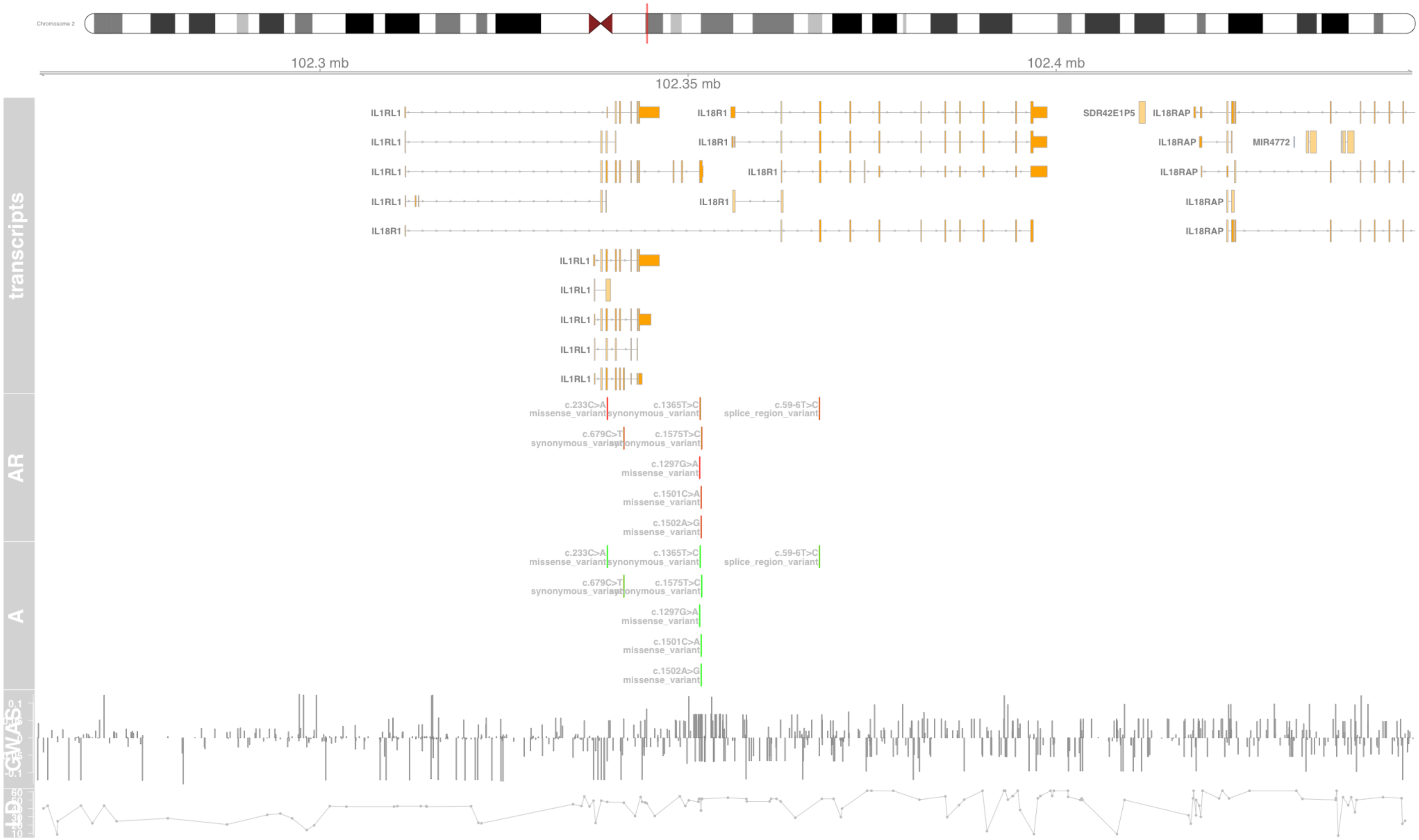

Fig S64 IL1RL1

interleukin 1 receptor like 1 [Source:HGNC Symbol;Acc:HGNC:5998]

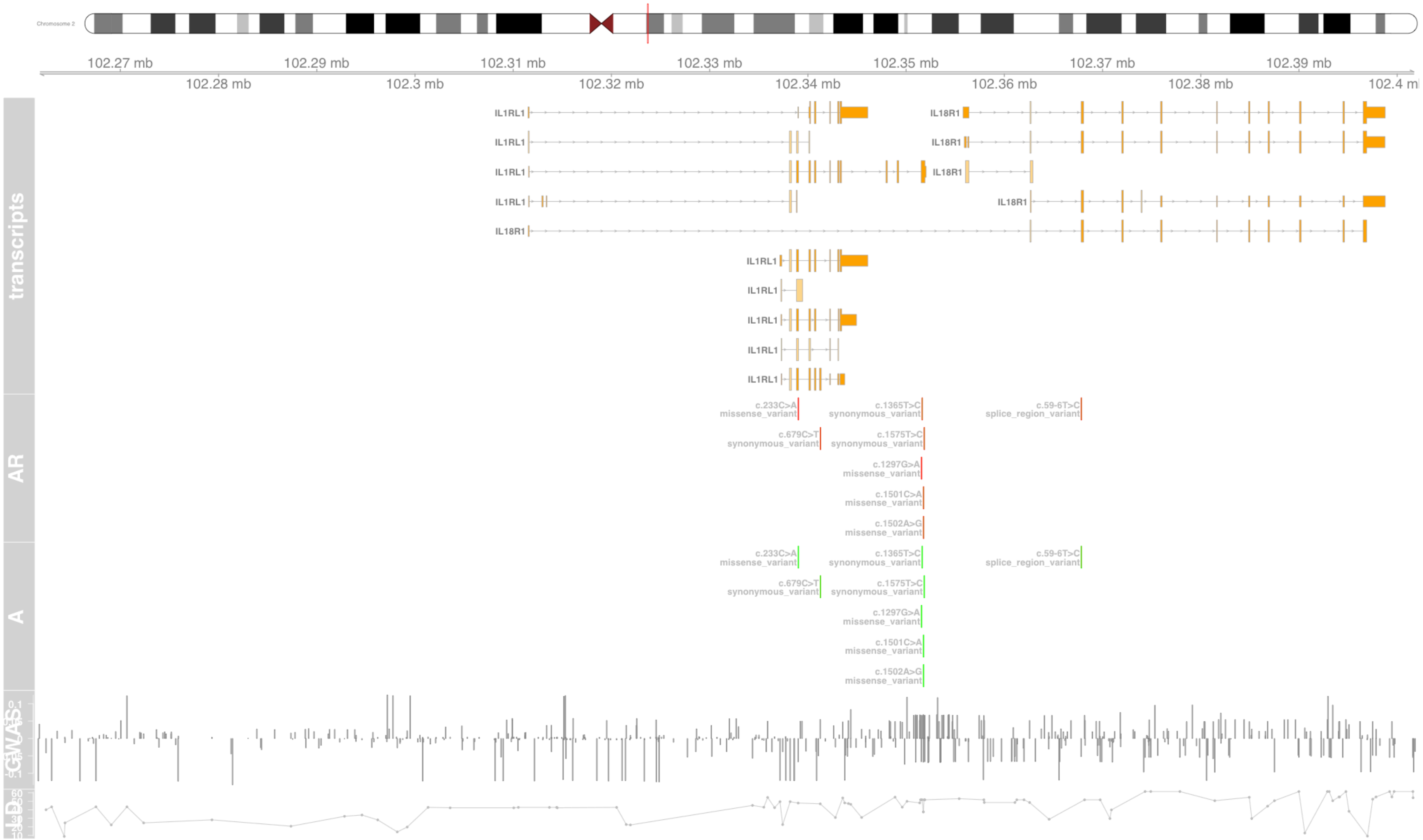

Fig S65 IL1RL2

interleukin 1 receptor like 2 [Source:HGNC Symbol;Acc:HGNC:5999]

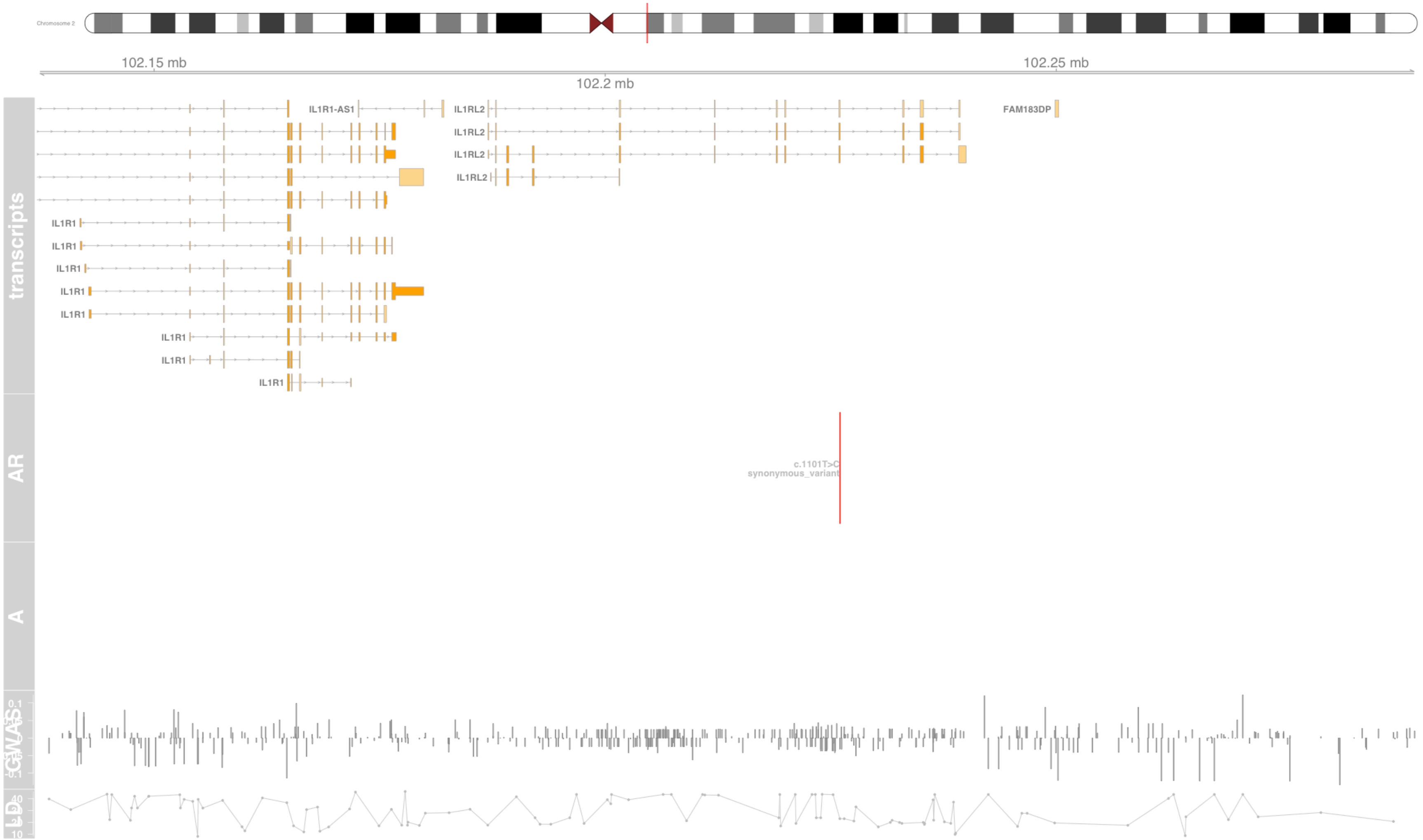

Fig S66 IL2

interleukin 2 [Source:HGNC Symbol;Acc:HGNC:6001]

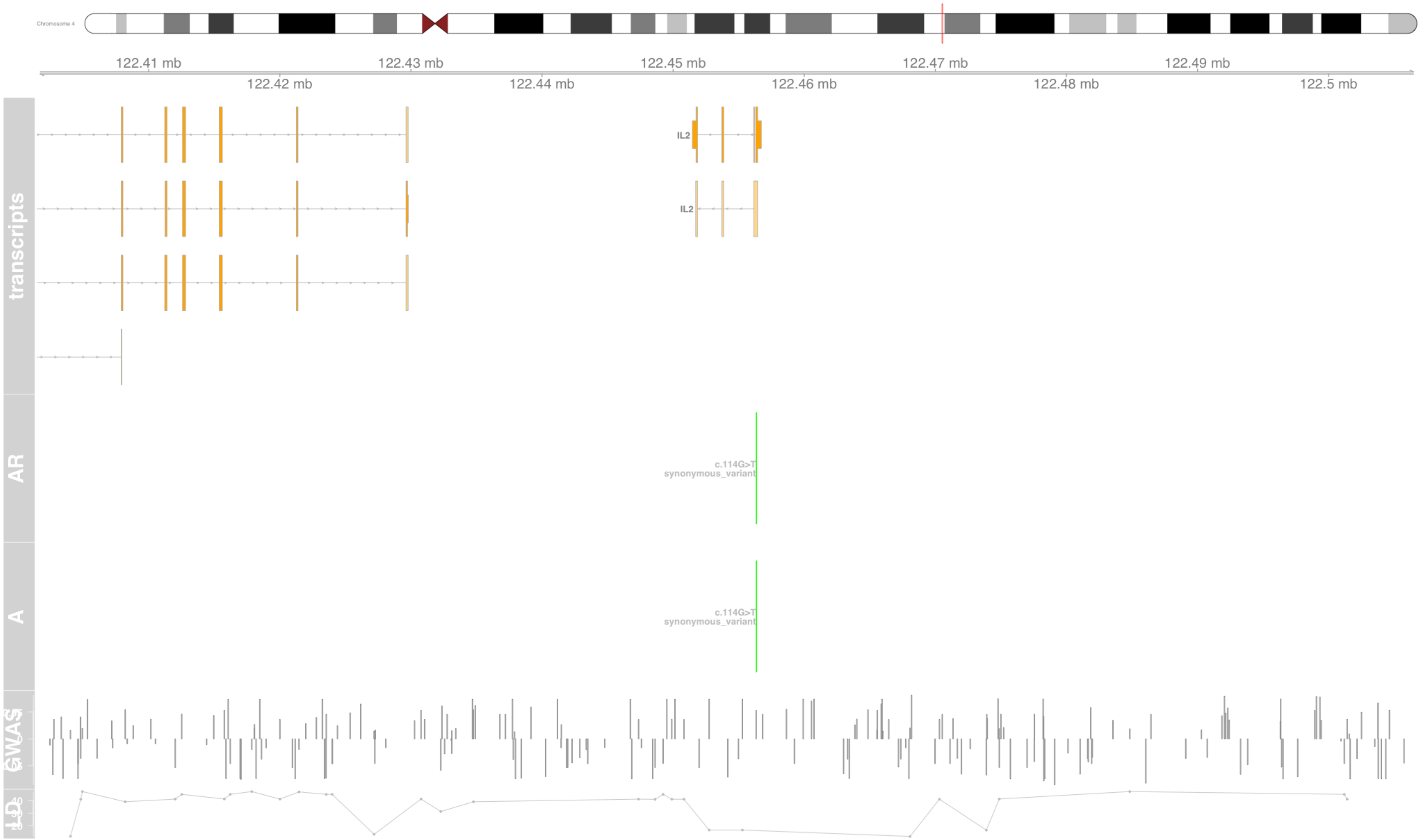

Fig S67 IL33

interleukin 33 [Source:HGNC Symbol;Acc:HGNC:16028]

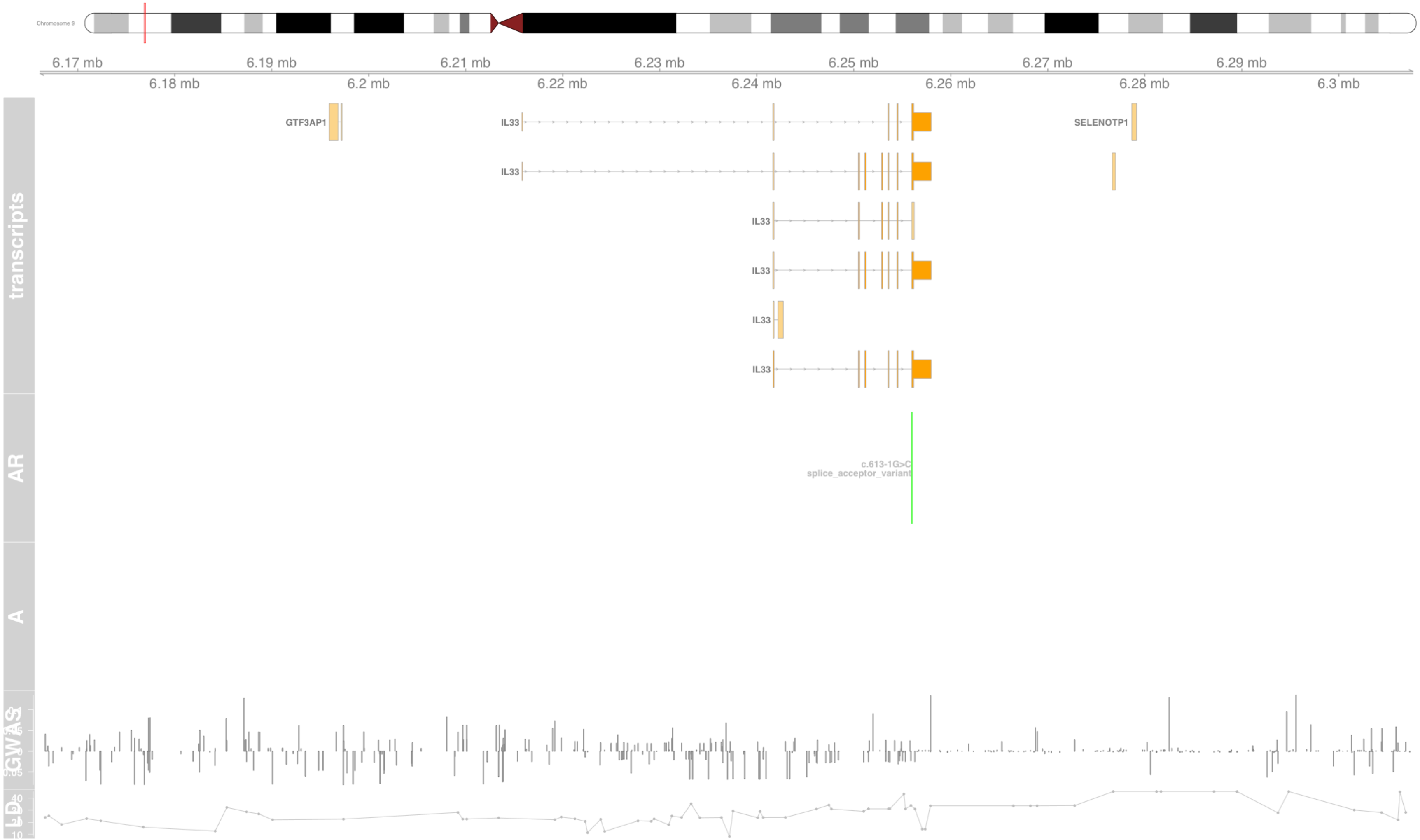

Fig S68 IL4R

interleukin 4 receptor [Source:HGNC Symbol;Acc:HGNC:6015]

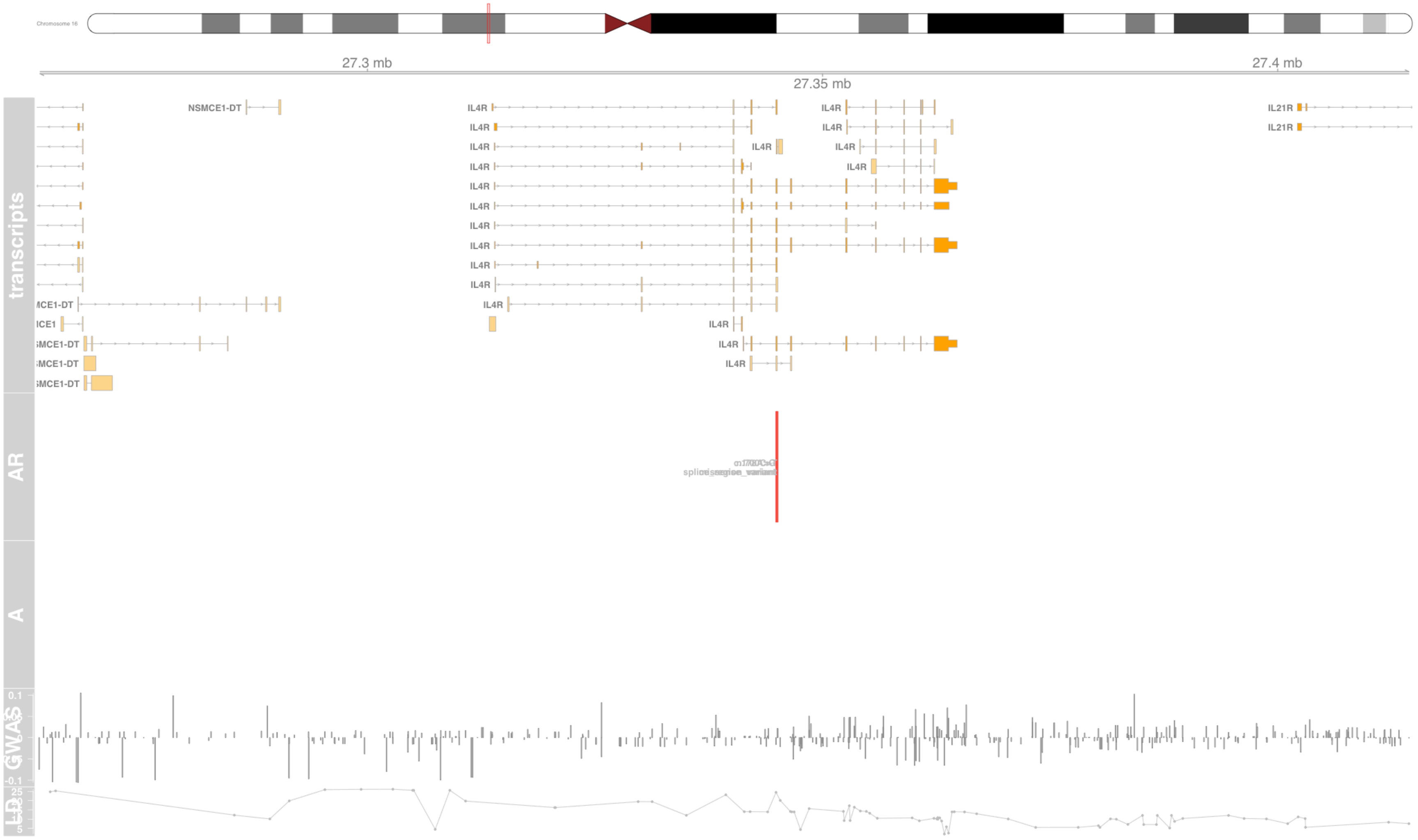

Fig S69 IRF1

interferon regulatory factor 1 [Source:HGNC Symbol;Acc:HGNC:6116]

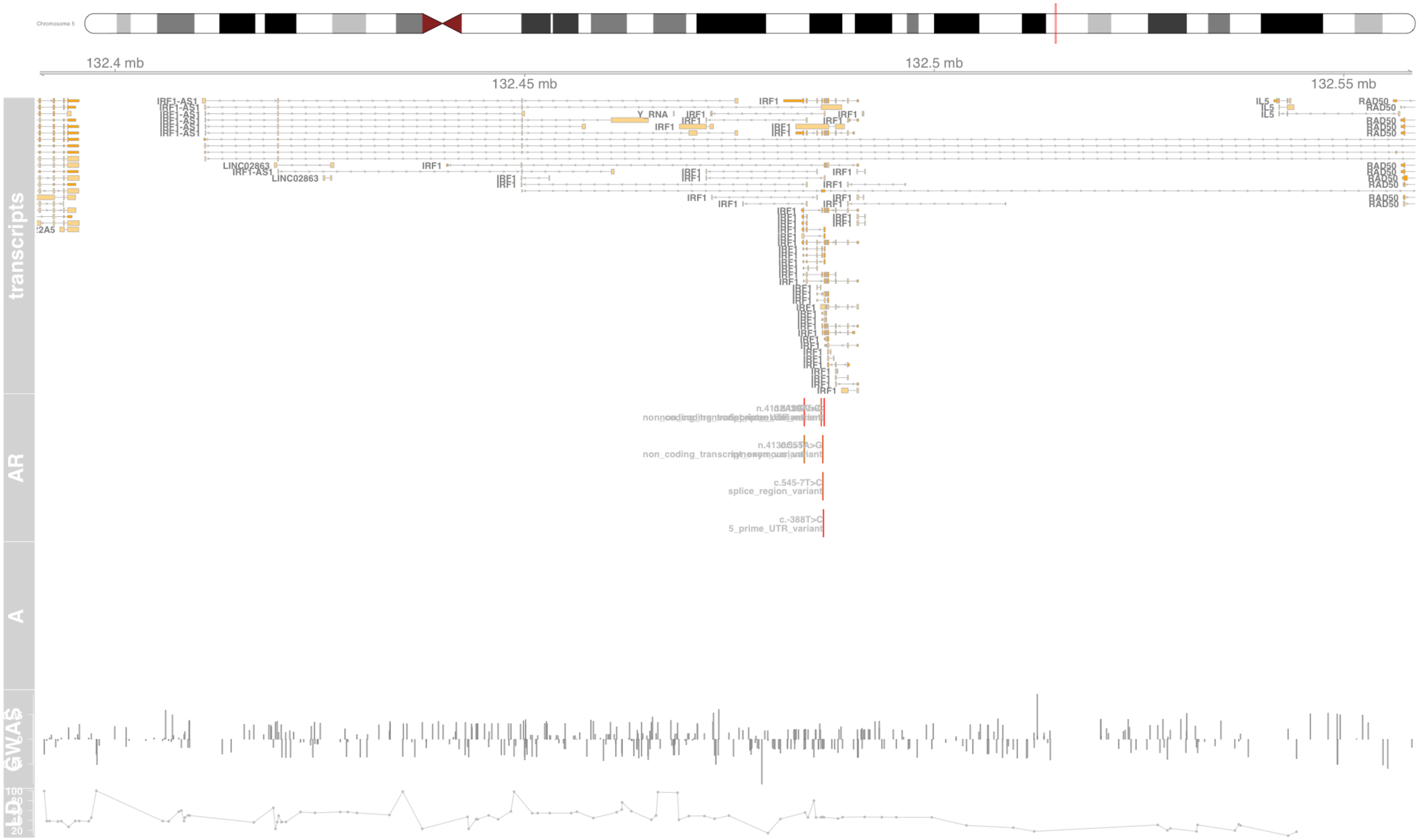

Fig S70 KIAA1109

KIAA1109 [Source:HGNC Symbol;Acc:HGNC:26953]

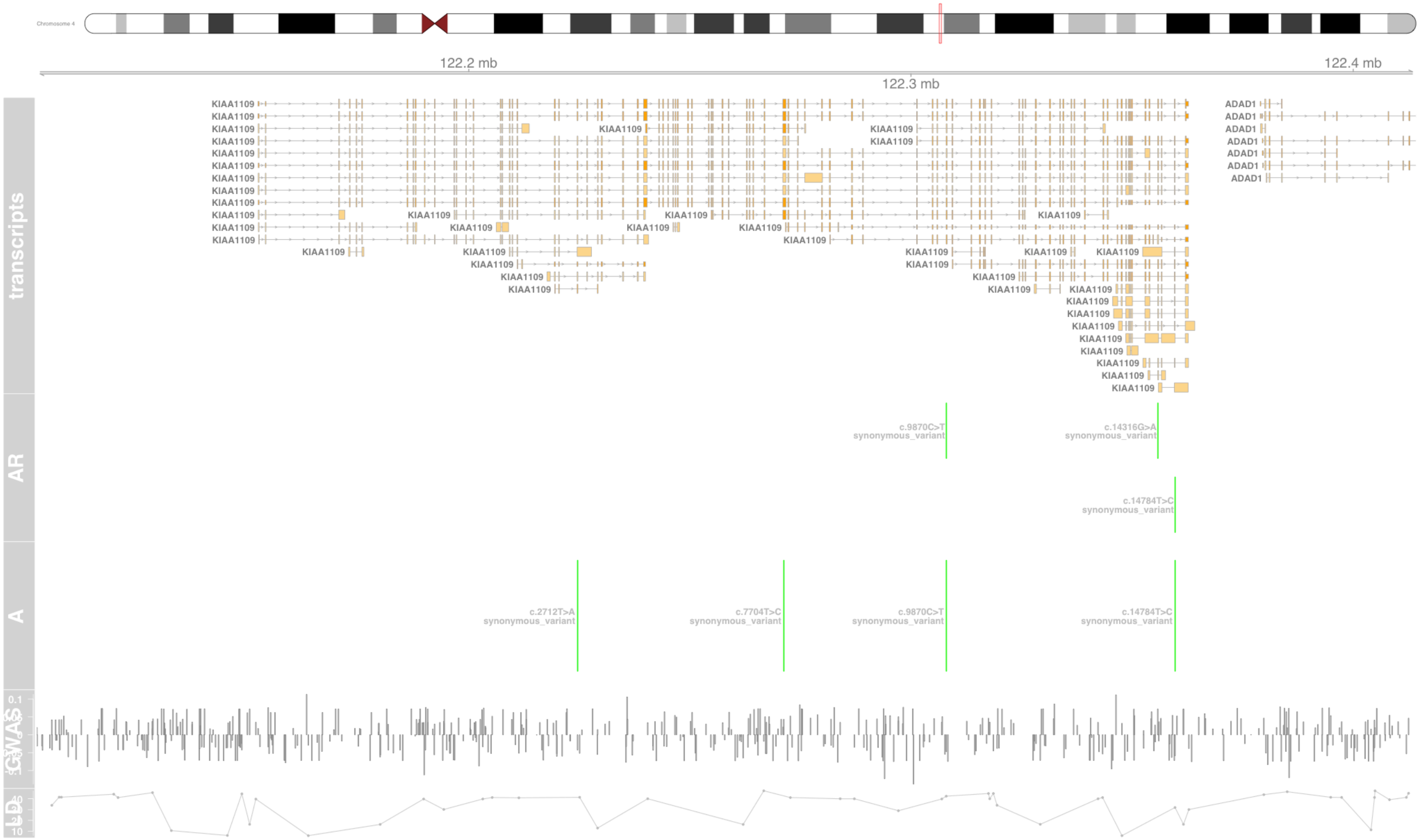

Fig S71 KIF3A

kinesin family member 3A [Source:HGNC Symbol;Acc:HGNC:6319]

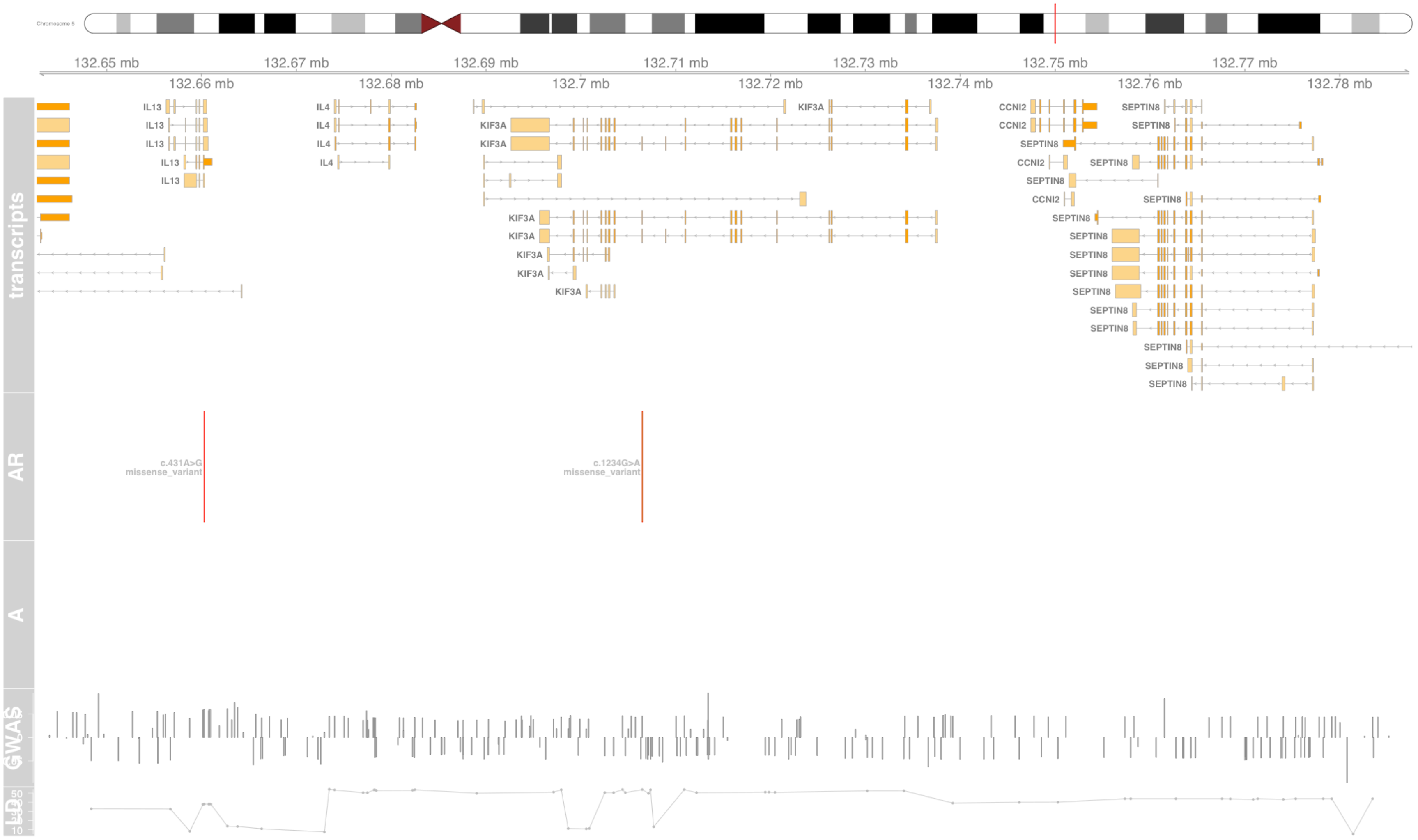

Fig S72 LRP1

LDL receptor related protein 1 [Source:HGNC Symbol;Acc:HGNC:6692]

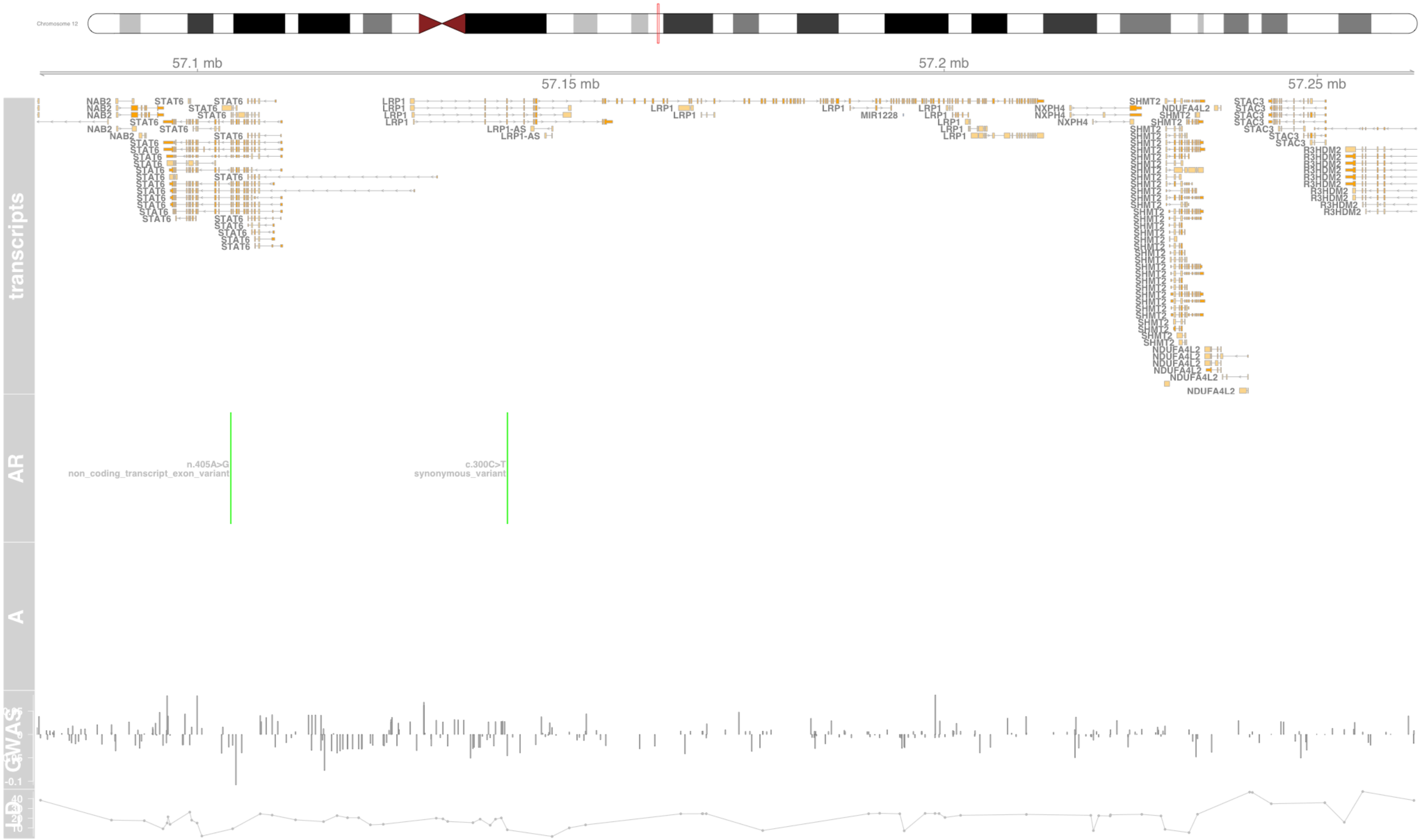

Fig S73 LTA

lymphotoxin alpha [Source:HGNC Symbol;Acc:HGNC:6709]

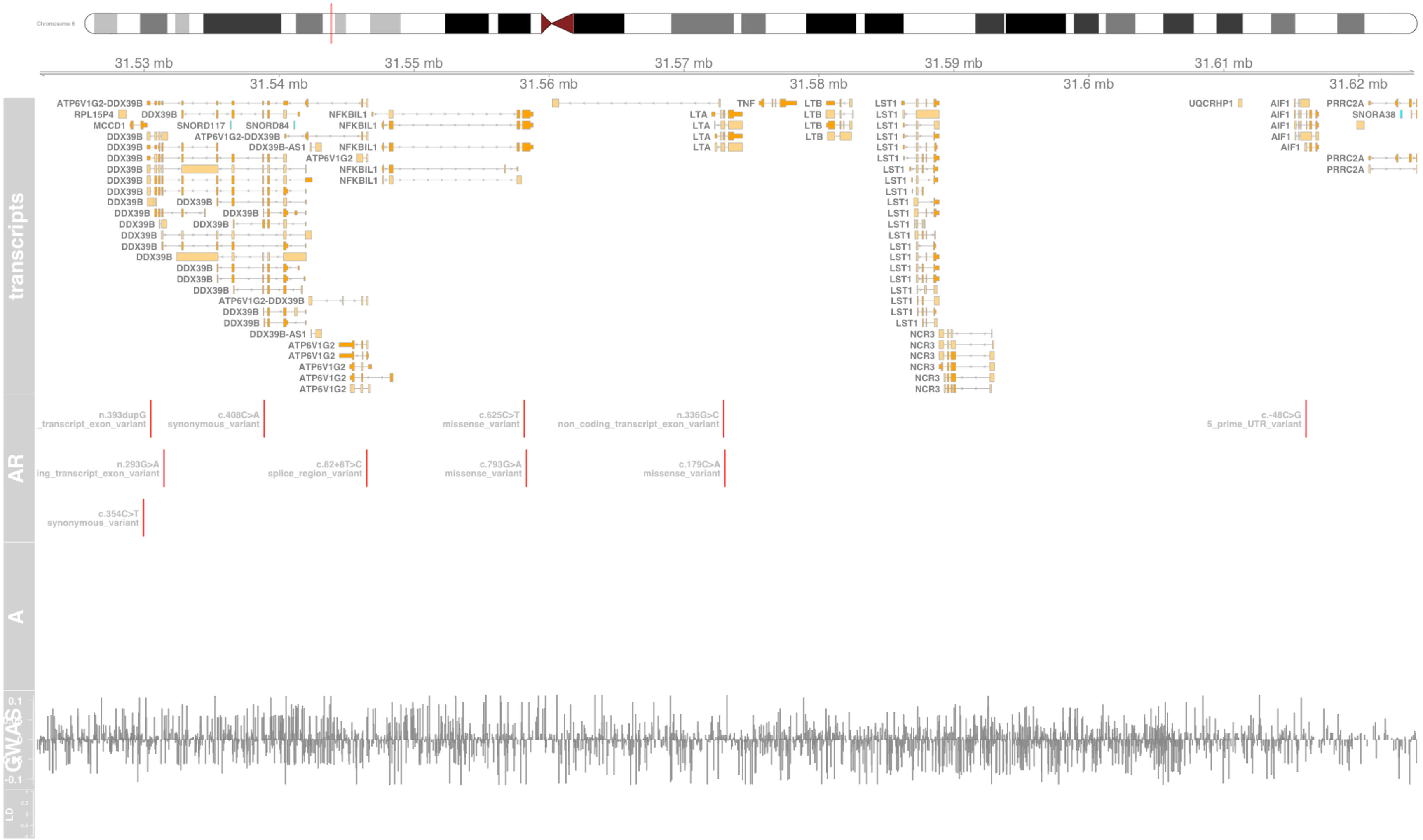

Fig S74 LY6G6C

lymphocyte antigen 6 family member G6C [Source:HGNC Symbol;Acc:HGNC:13936]

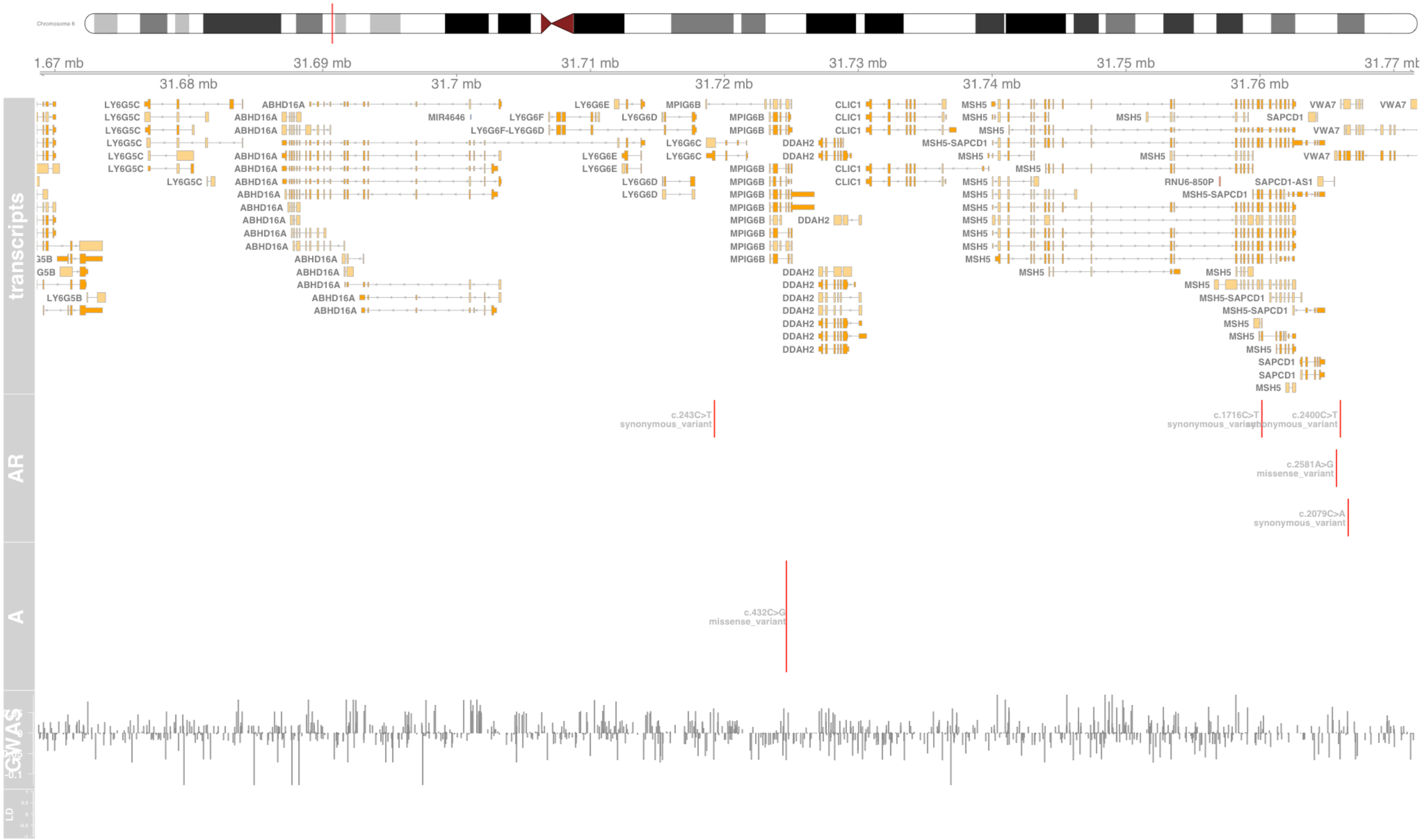

Fig S75 MCCD1

mitochondrial coiled-coil domain 1 [Source:HGNC Symbol;Acc:HGNC:20668]

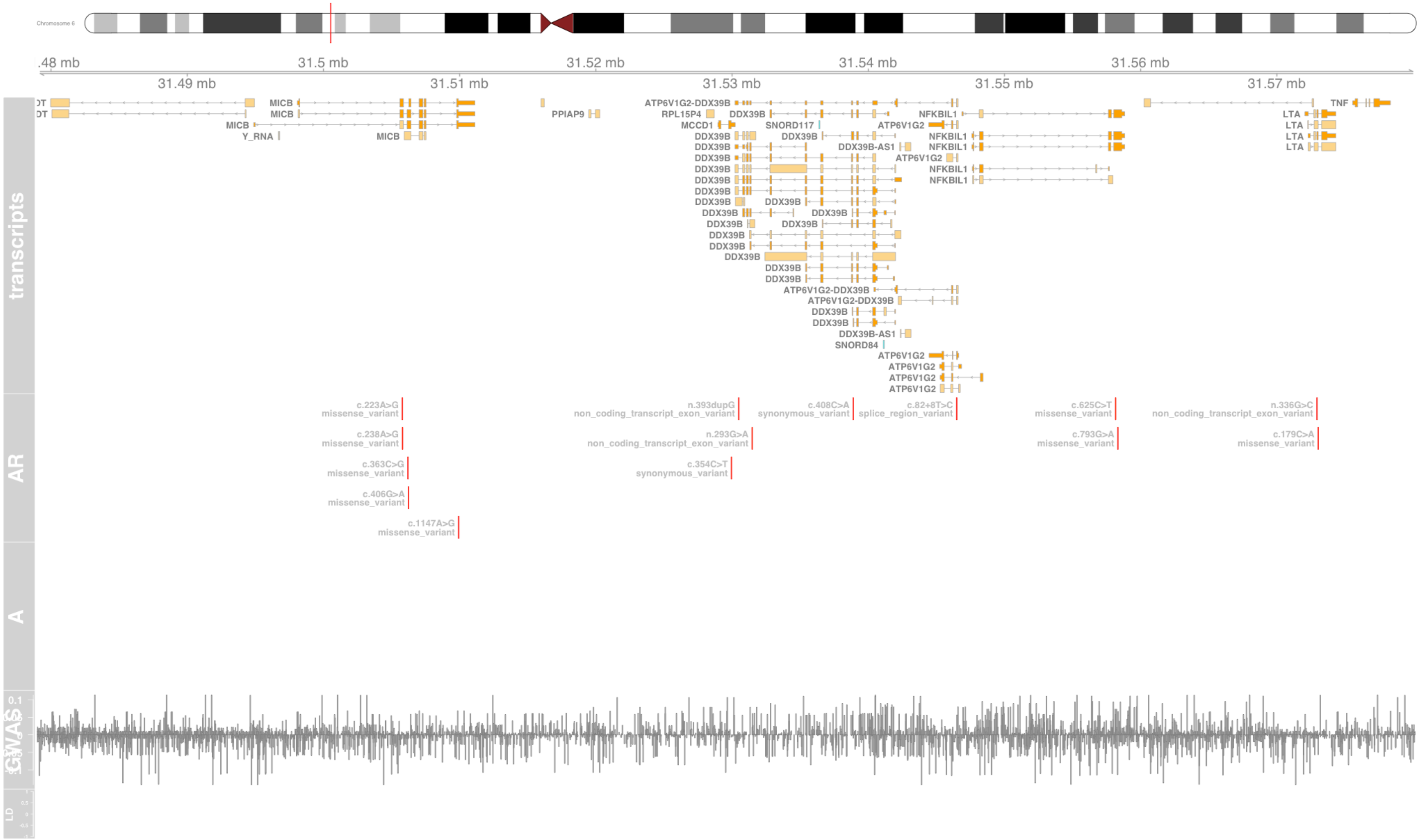

Fig S76 MED24

mediator complex subunit 24 [Source:HGNC Symbol;Acc:HGNC:22963]

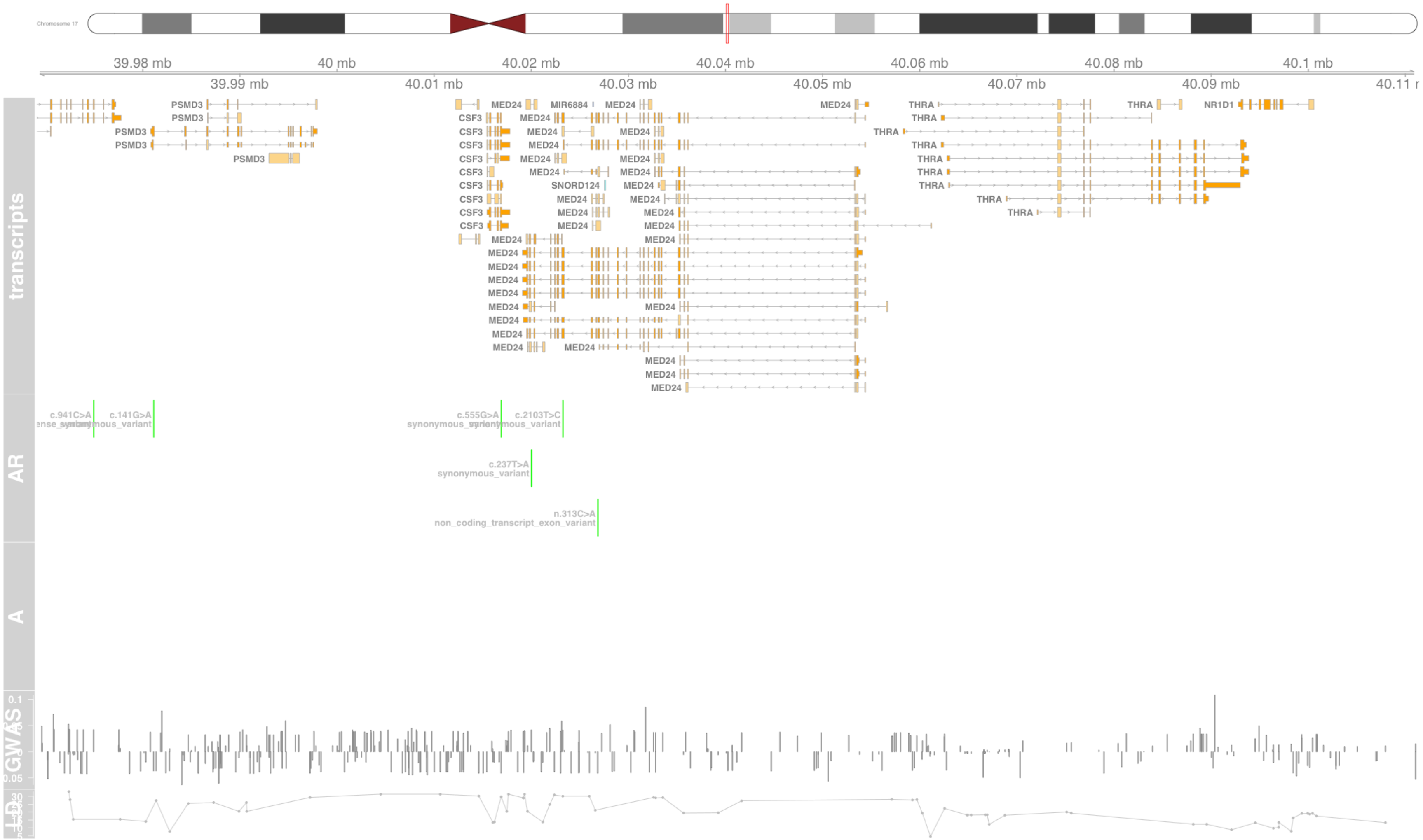

Fig S77 MEI1

meiotic double-stranded break formation protein 1 [Source:HGNC Symbol;Acc:HGNC:28613]

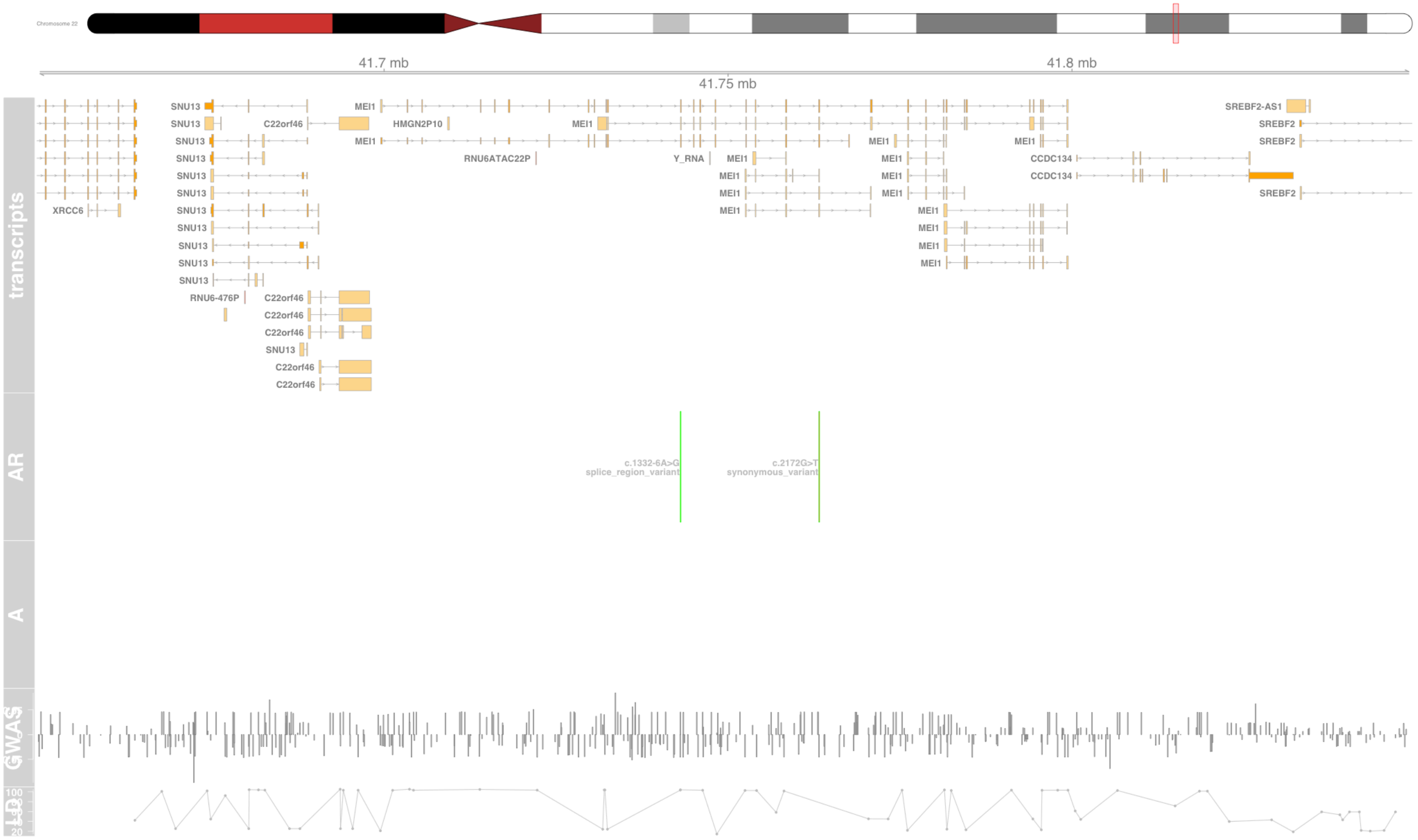

Fig S78 MICA

MHC class I polypeptide-related sequence A [Source:HGNC Symbol;Acc:HGNC:7090]

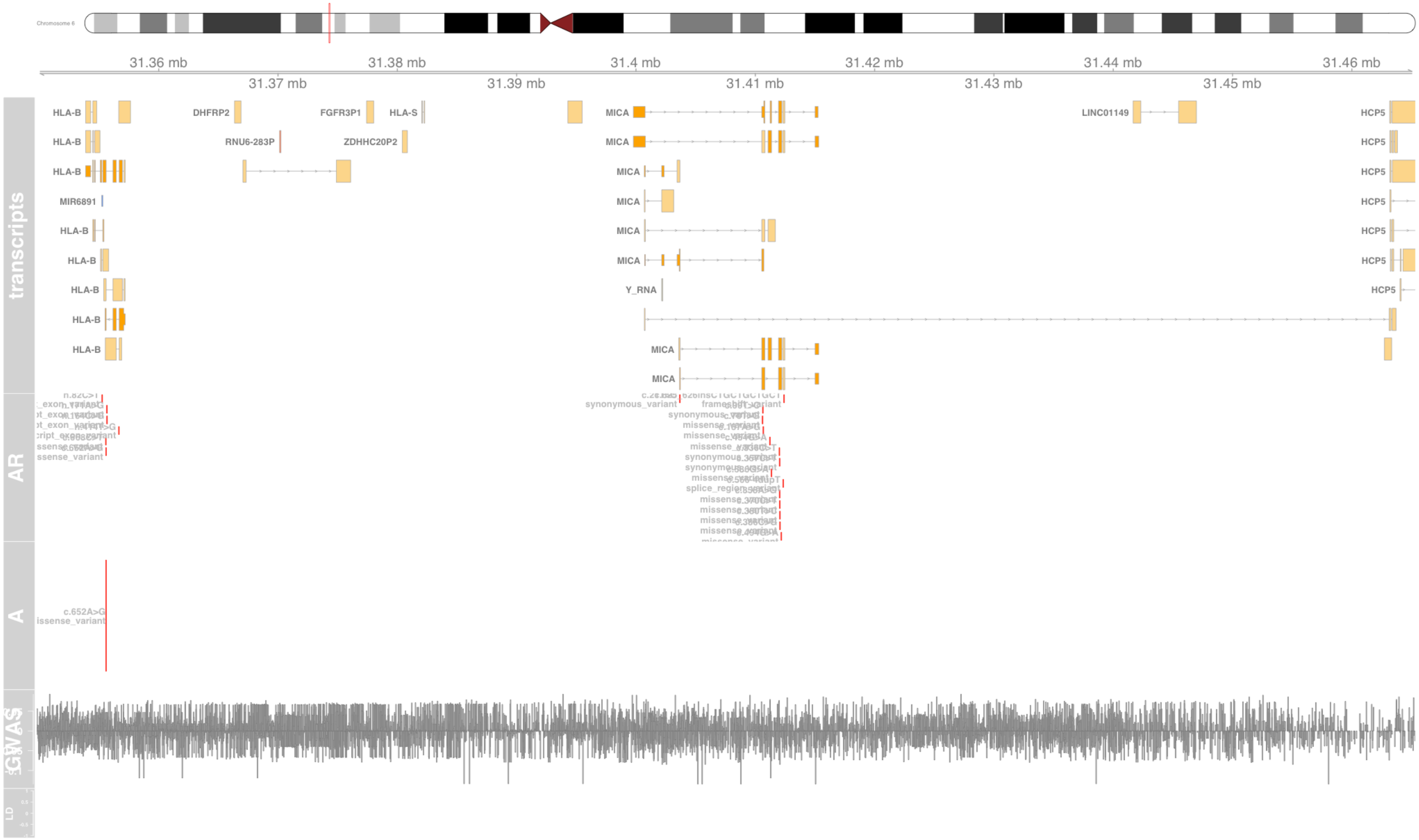

Fig S79 MICB

MHC class I polypeptide-related sequence B [Source:HGNC Symbol;Acc:HGNC:7091]

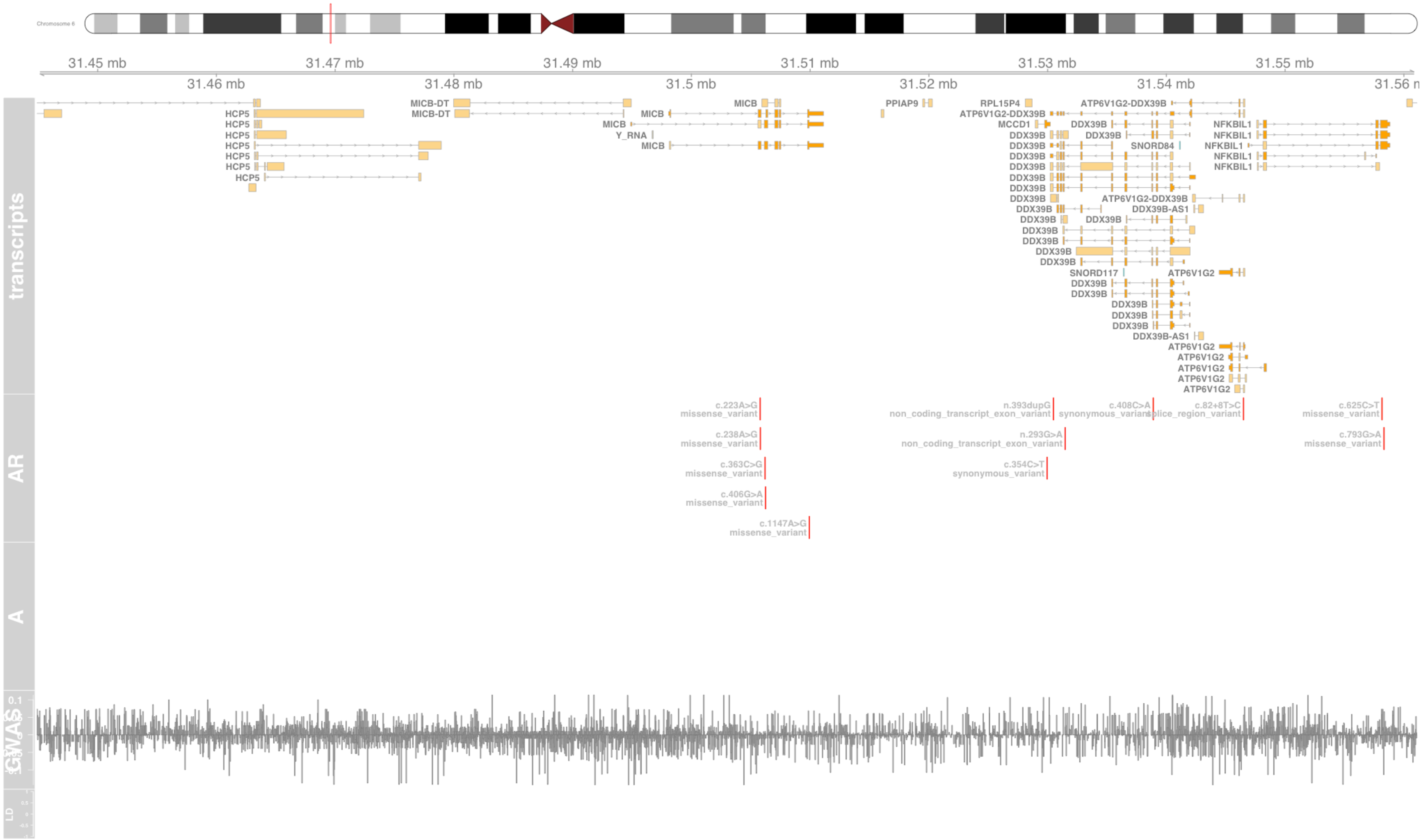

Fig S80 MIR6891

microRNA 6891 [Source:HGNC Symbol;Acc:HGNC:50243]

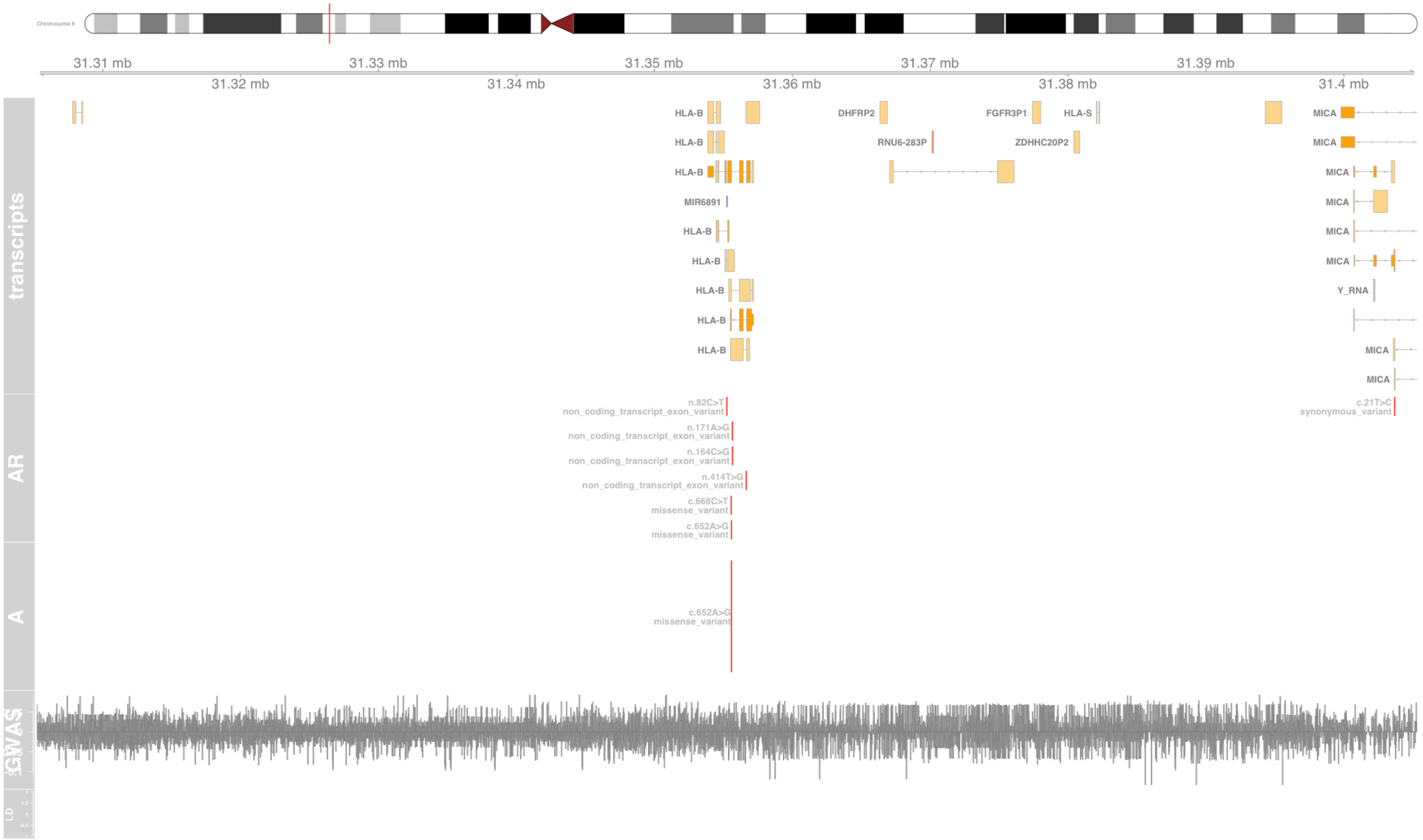

Fig S81 MPIG6B

megakaryocyte and platelet inhibitory receptor G6b [Source:HGNC Symbol;Acc:HGNC:13937]

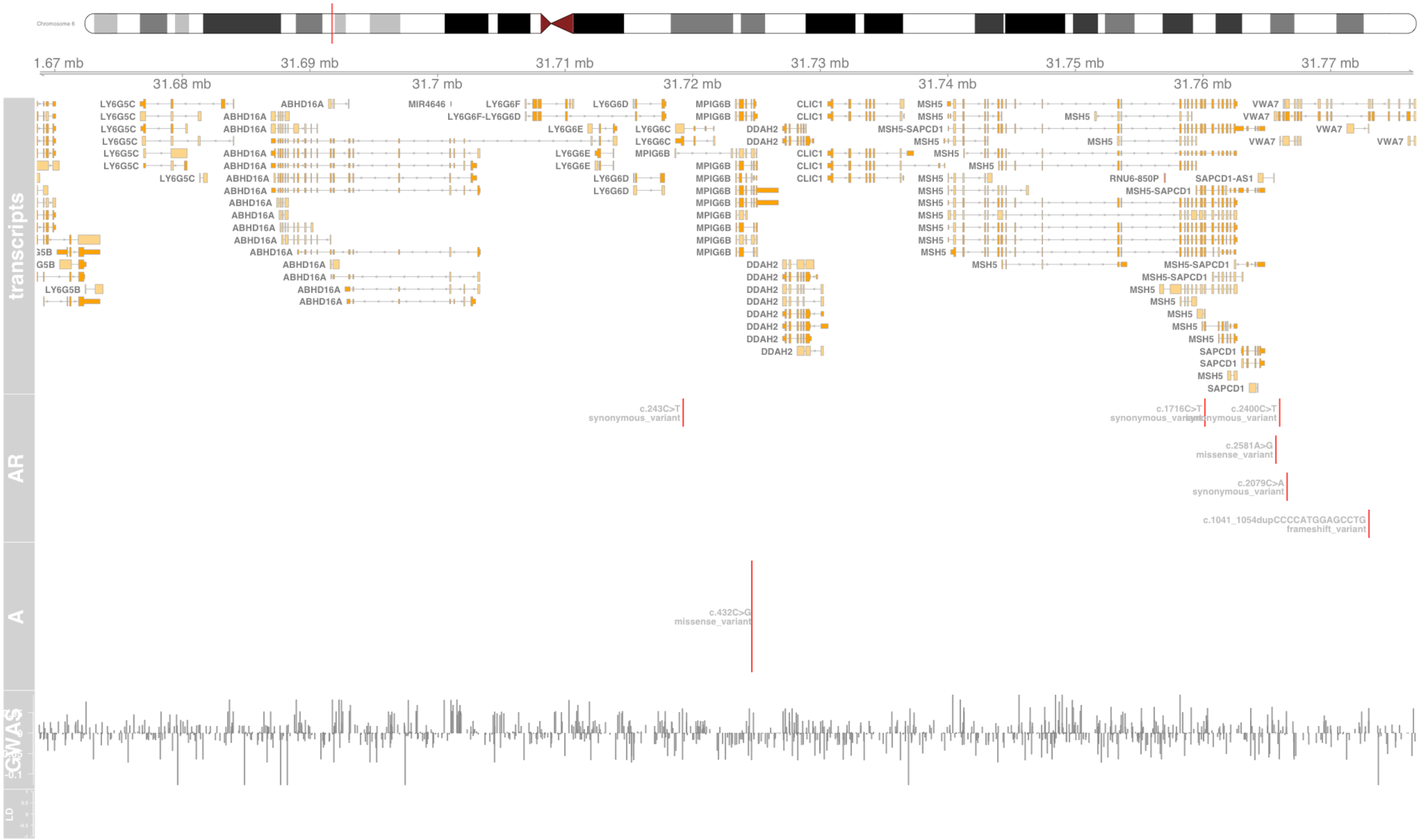

Fig S82 MSH5

mutS homolog 5 [Source:HGNC Symbol;Acc:HGNC:7328]

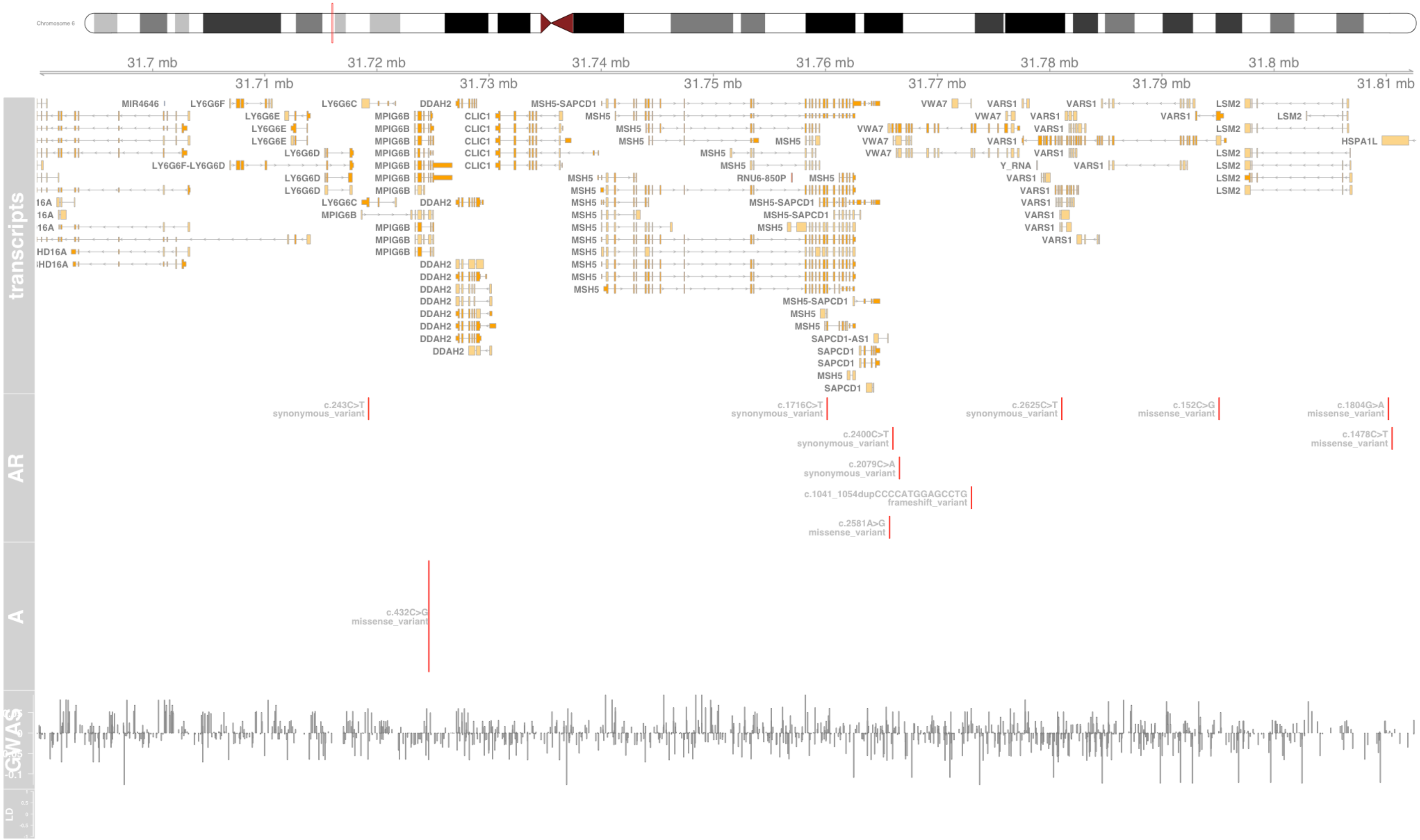

Fig S83 MUC22

mucin 22 [Source:HGNC Symbol;Acc:HGNC:39755]

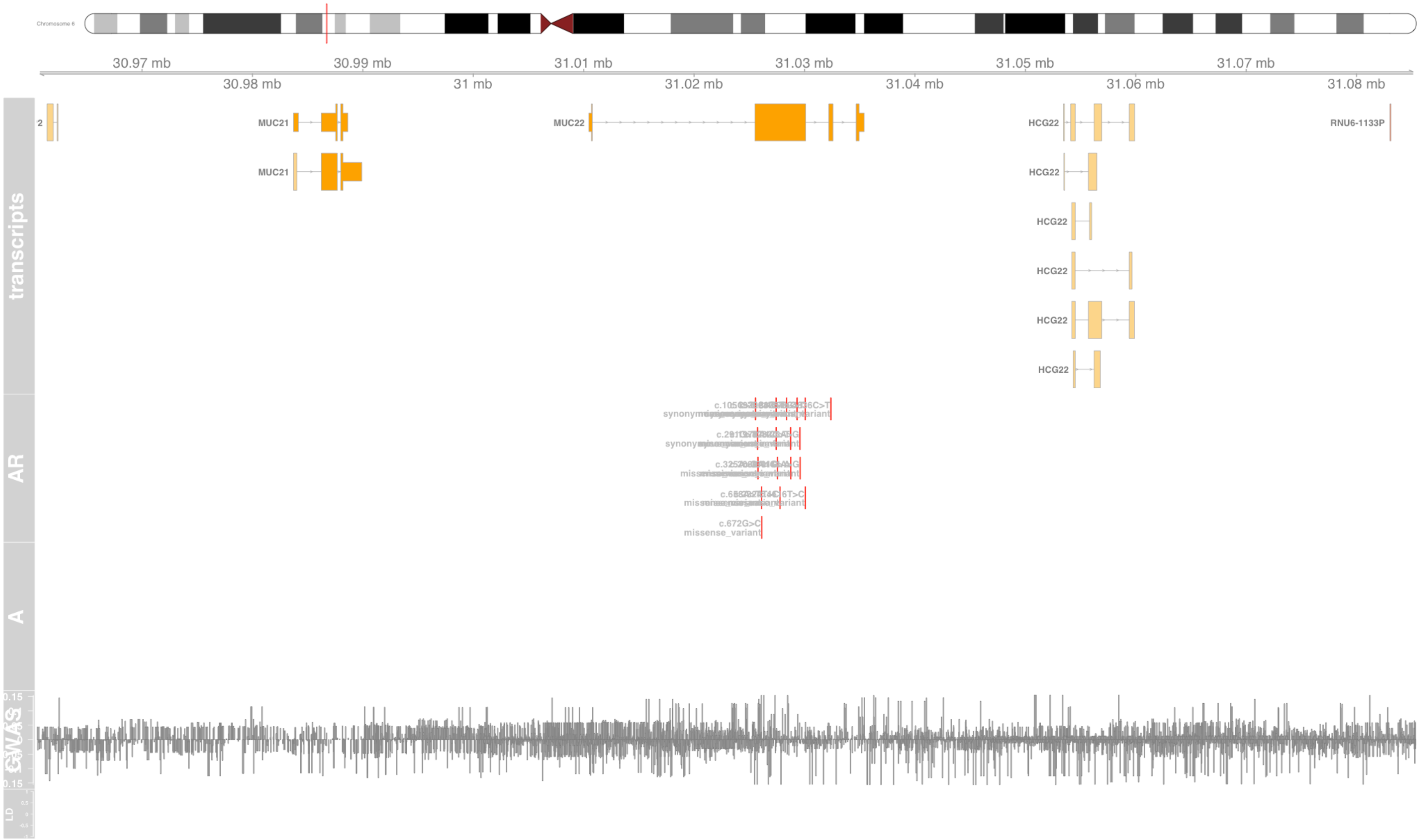

Fig S84 MYRF

myelin regulatory factor [Source:HGNC Symbol;Acc:HGNC:1181]

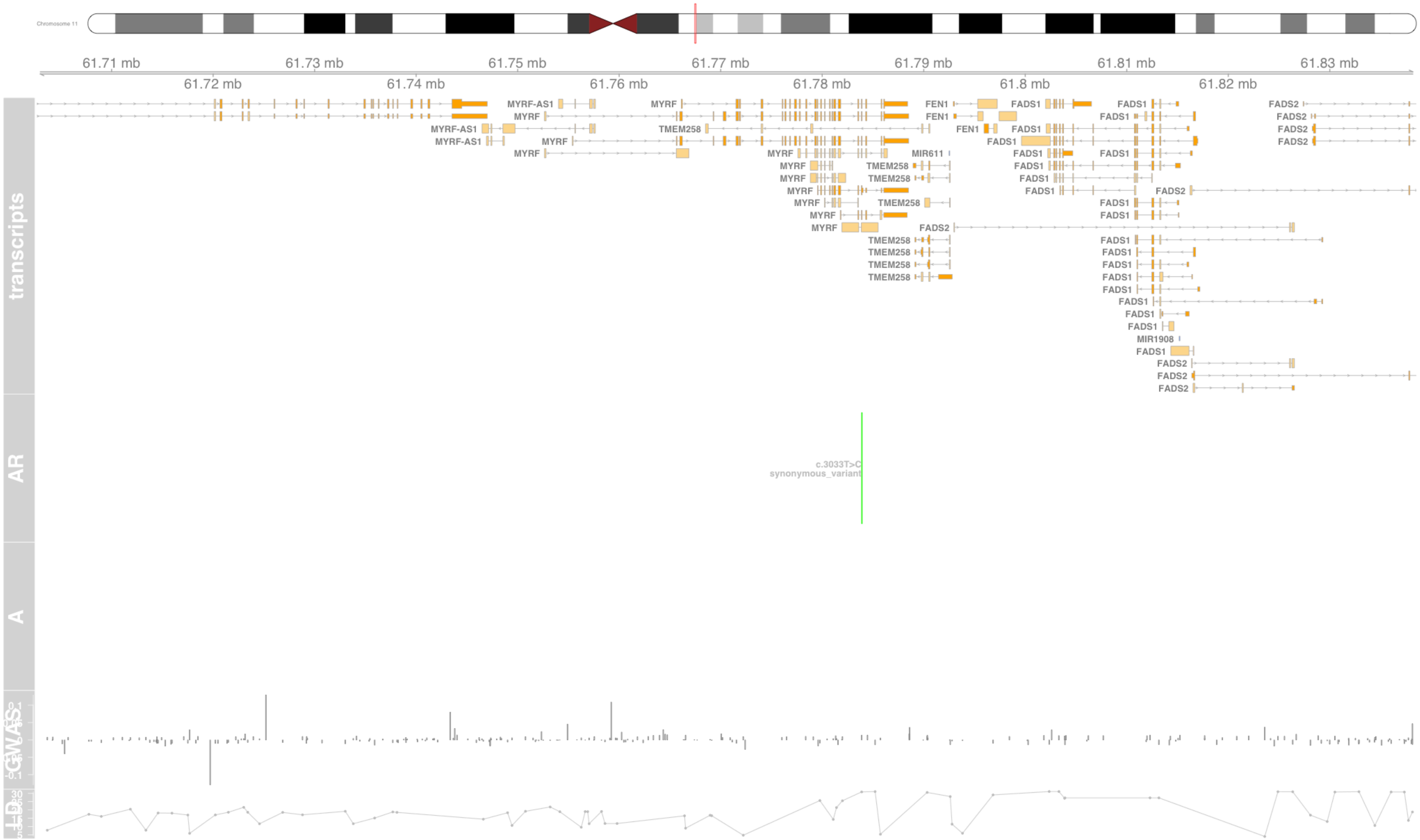

Fig S85 NEU1

neuraminidase 1 [Source:HGNC Symbol;Acc:HGNC:7758]

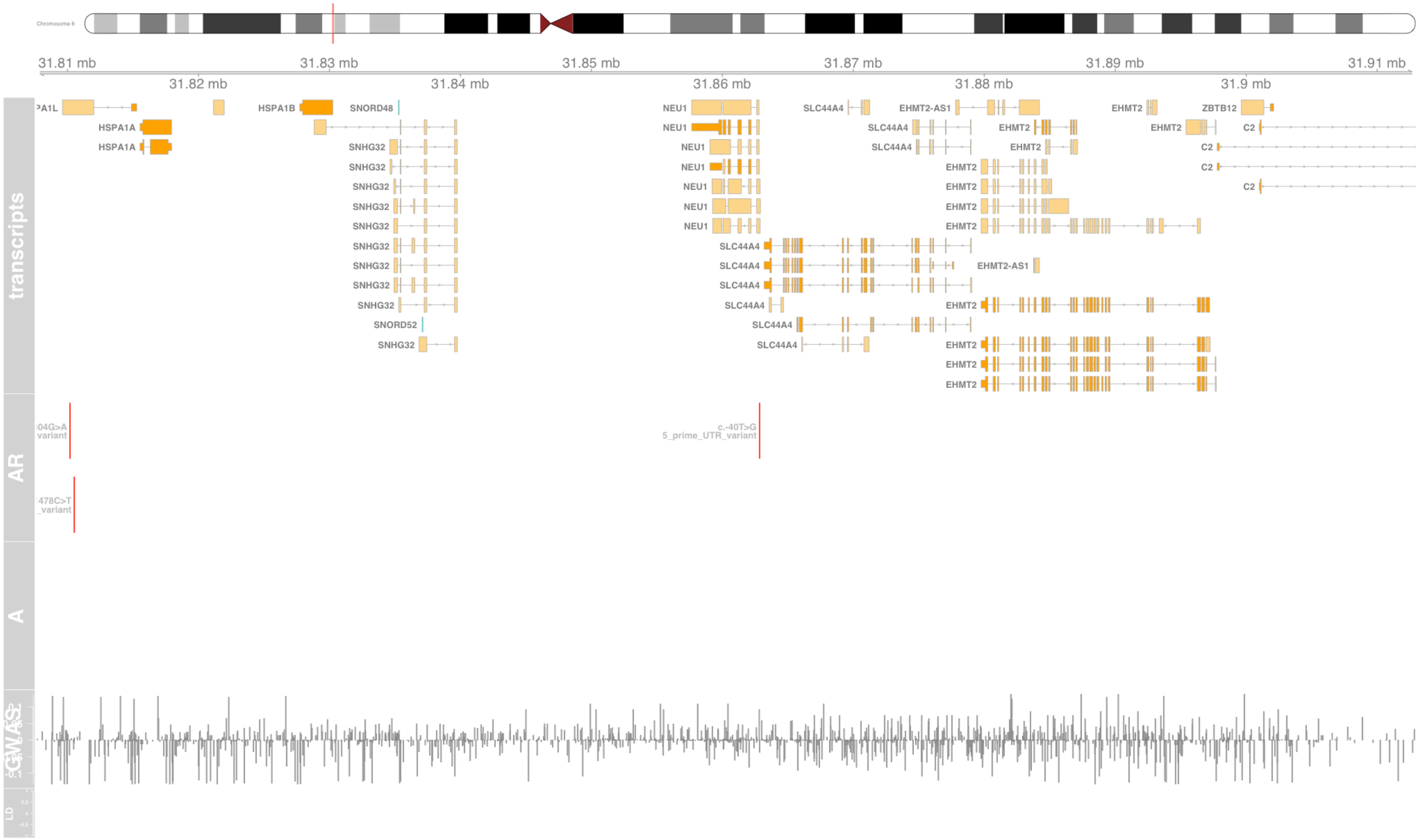

Fig S86 NFKBIL1

NFKB inhibitor like 1 [Source:HGNC Symbol;Acc:HGNC:7800]

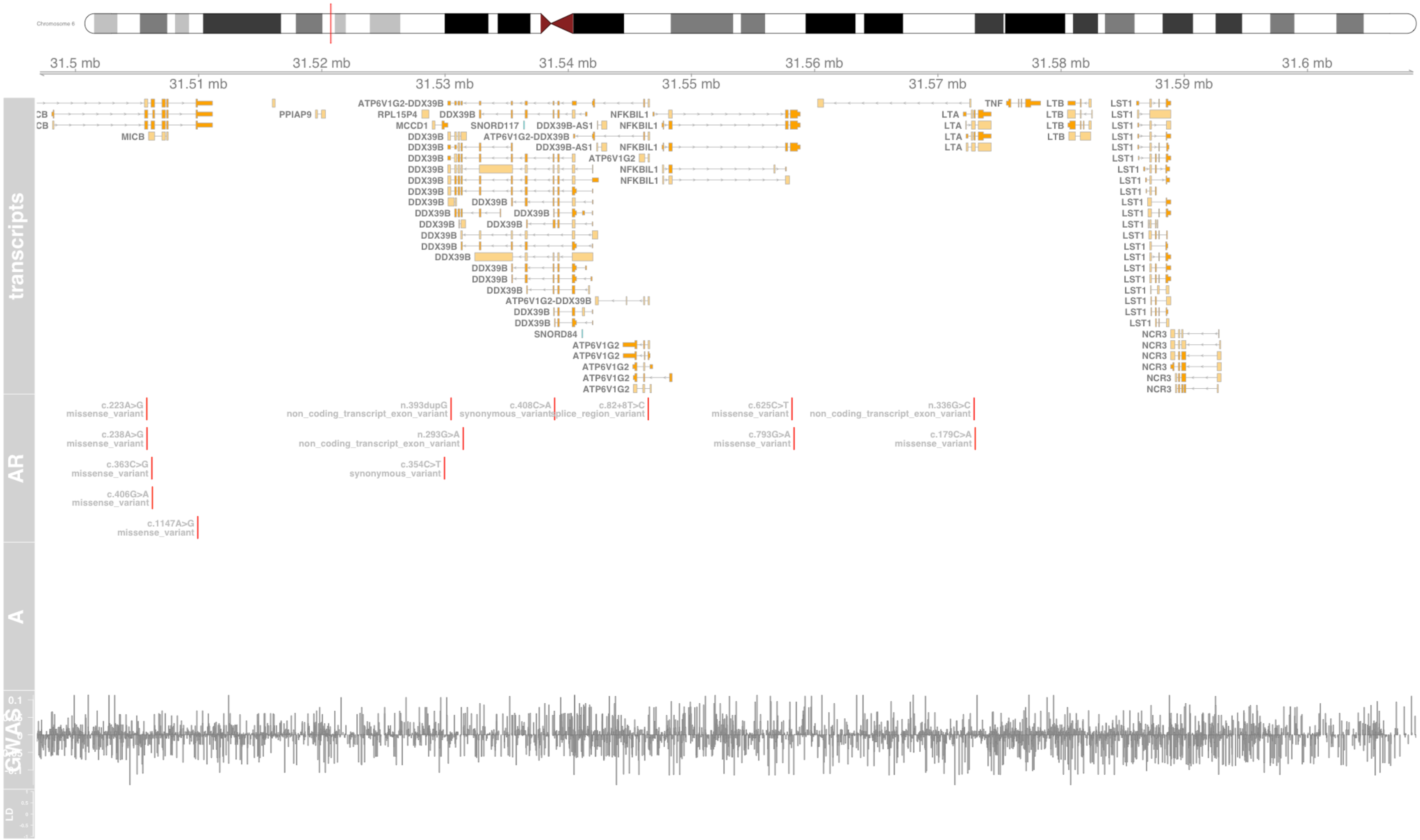

Fig S87 NOTCH4

notch receptor 4 [Source:HGNC Symbol;Acc:HGNC:7884]

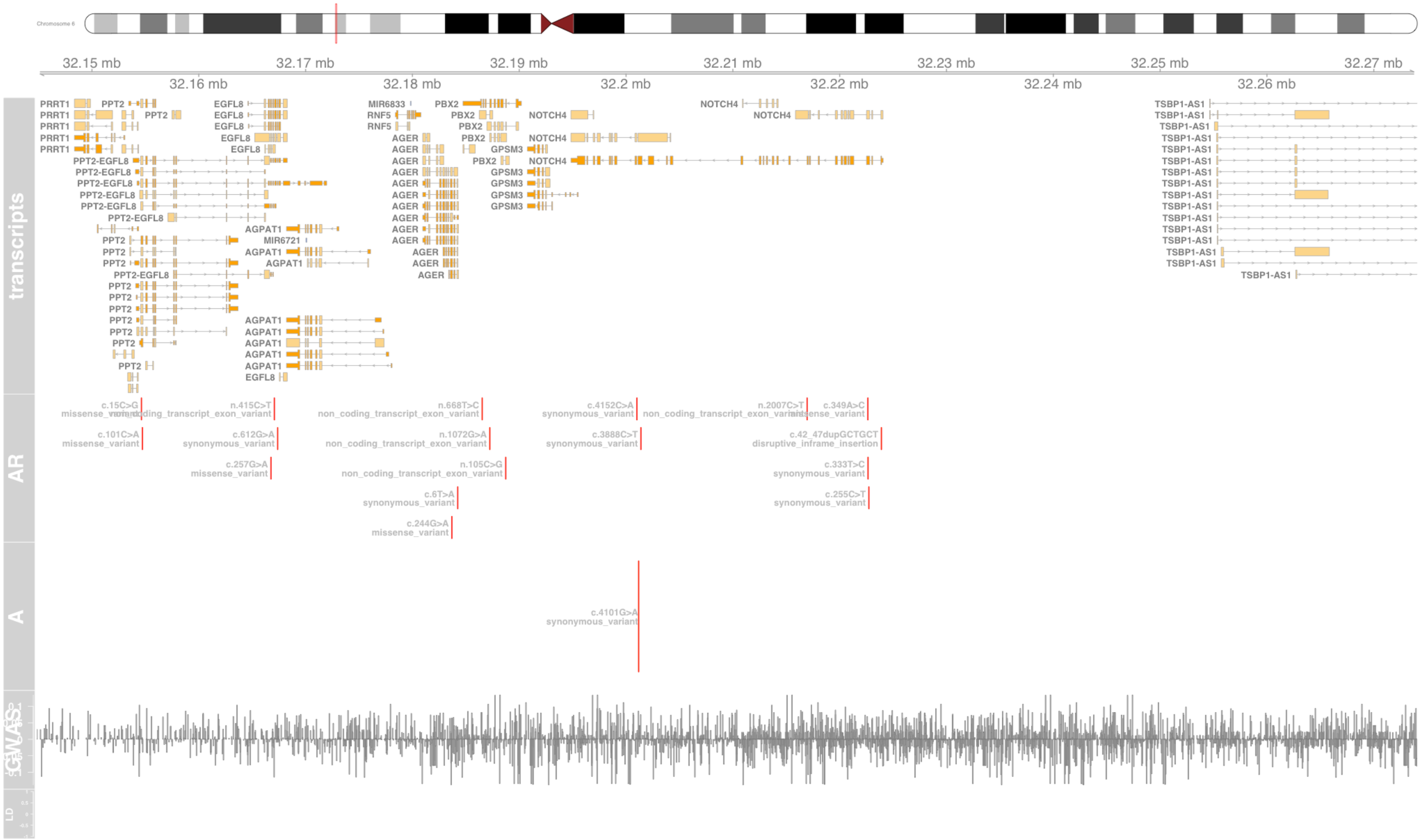

Fig S88 NSMCE1

NSE1 homolog, SMC5-SMC6 complex component [Source:HGNC Symbol;Acc:HGNC:29897]

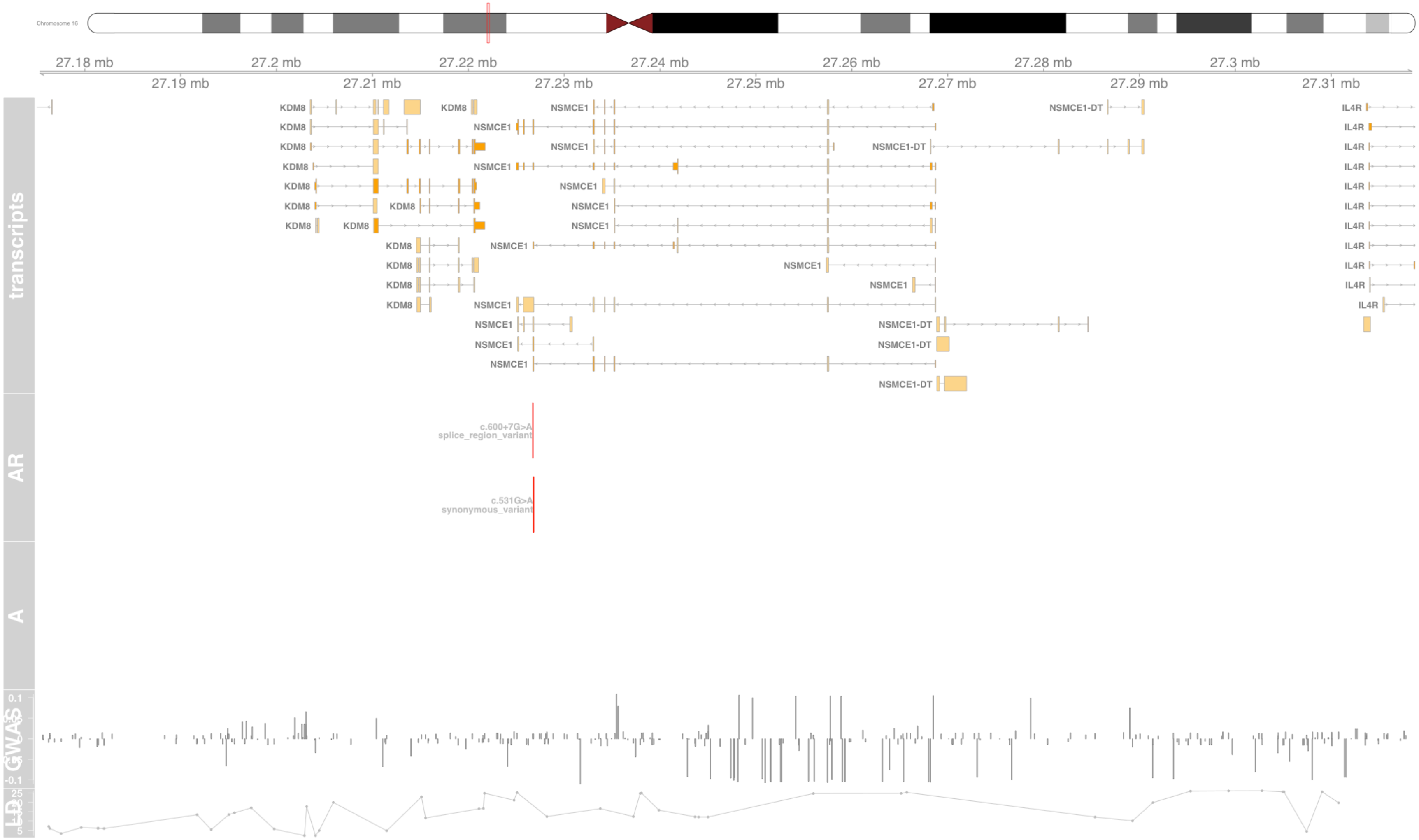

Fig S89 OR12D3

olfactory receptor family 12 subfamily D member 3 [Source:HGNC Symbol;Acc:HGNC:13963]

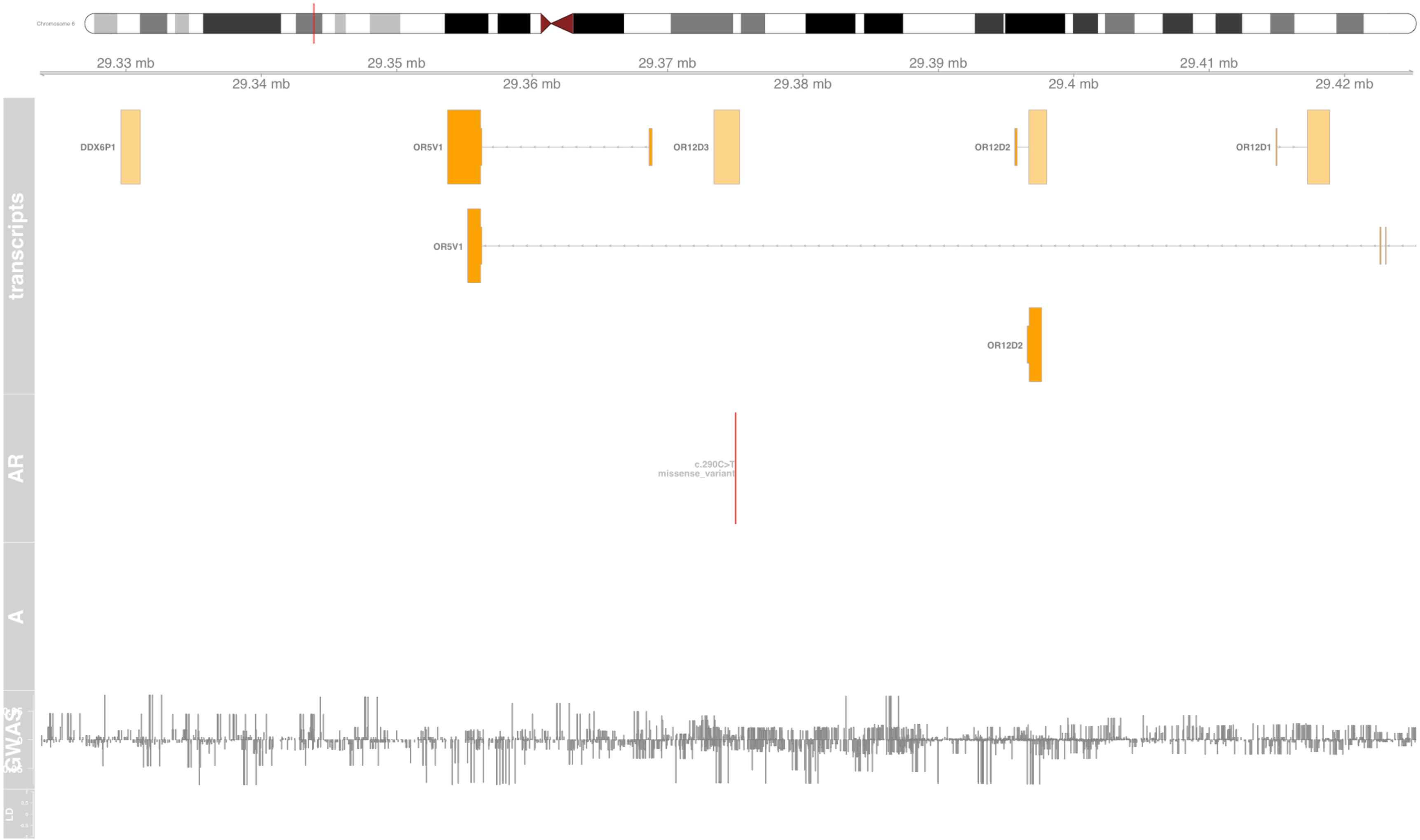

Fig S90 OR2B2

olfactory receptor family 2 subfamily B member 2 [Source:HGNC Symbol;Acc:HGNC:13966]

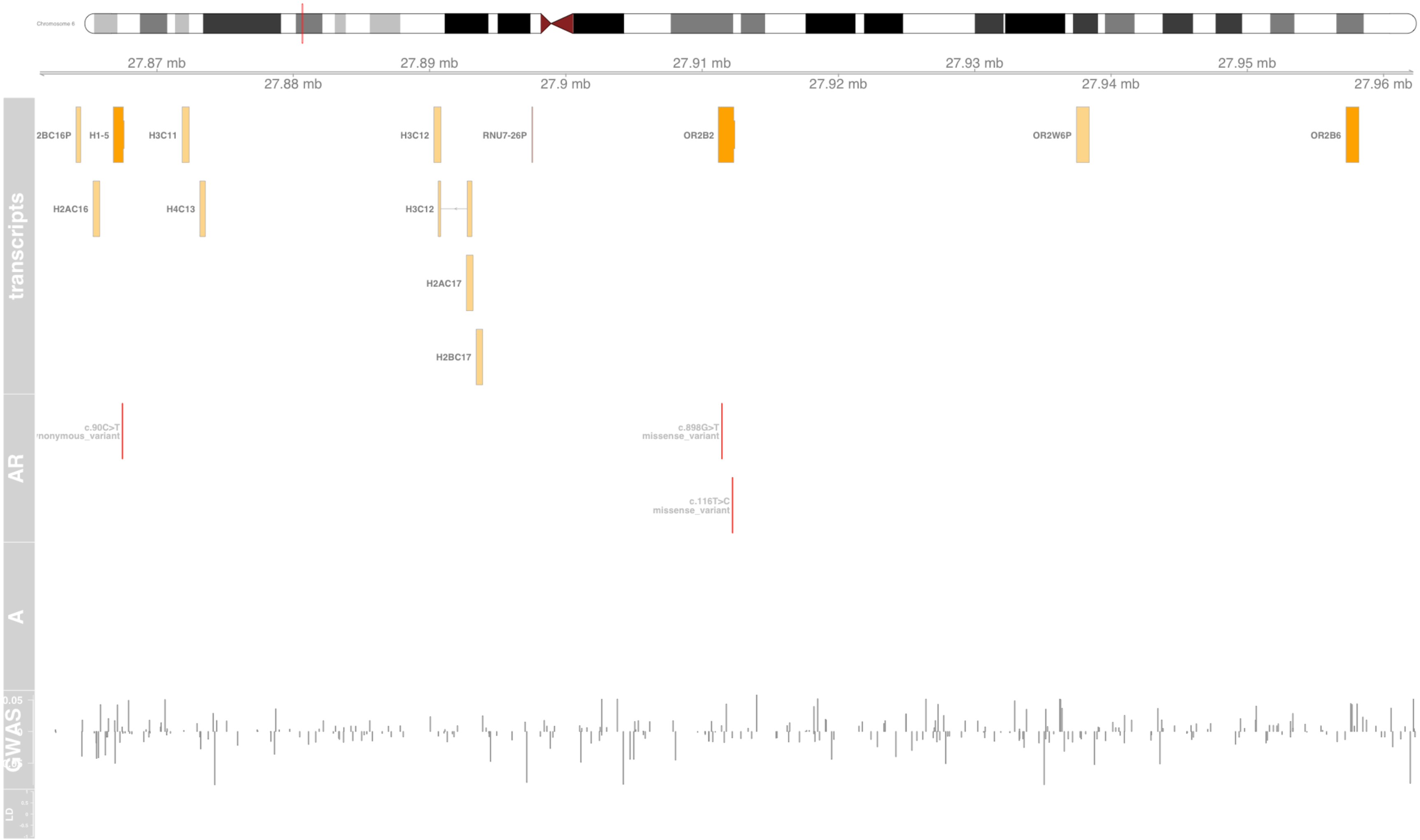

Fig S91 PBX2

PBX homeobox 2 [Source:HGNC Symbol;Acc:HGNC:8633]

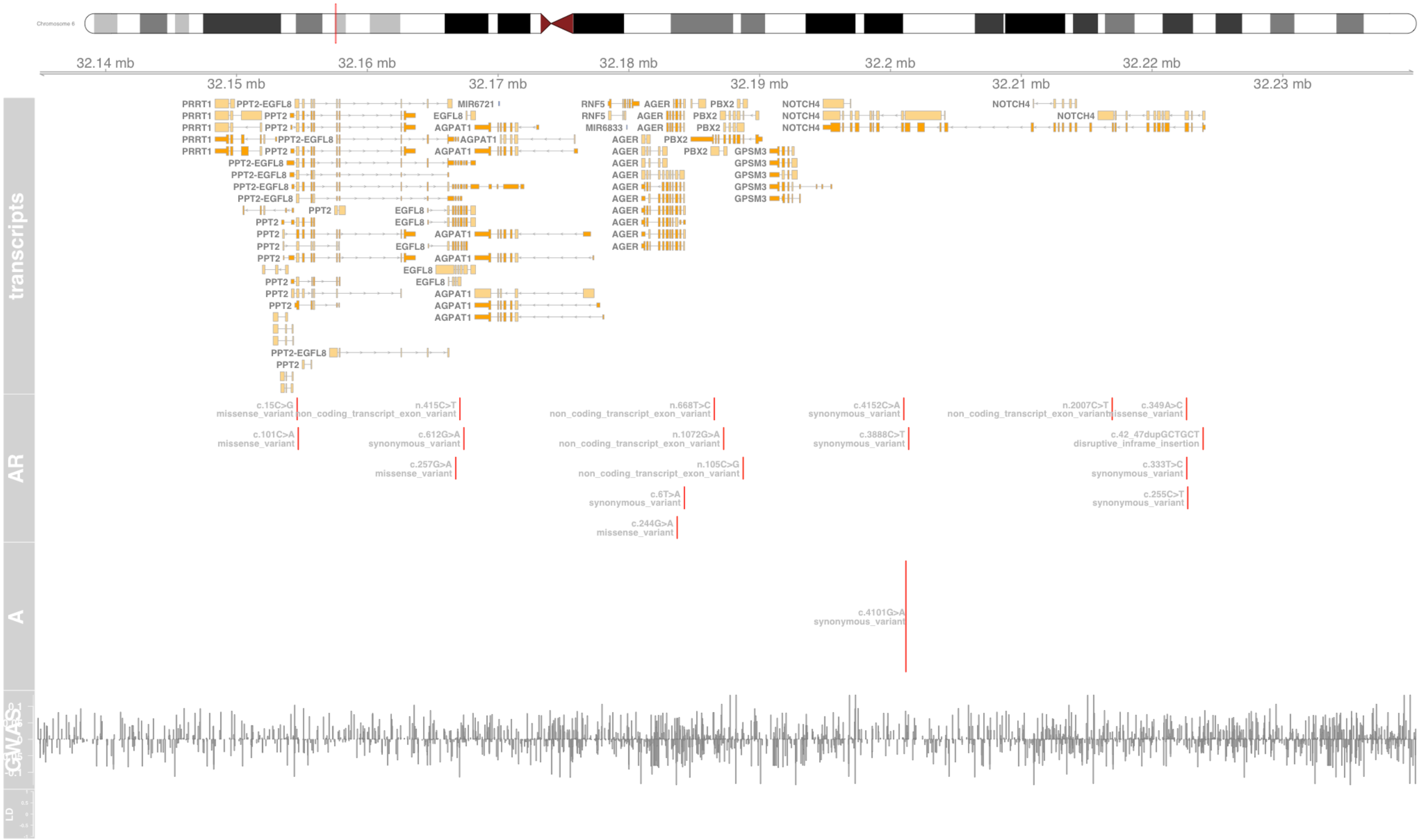

Fig S92 PDLIM4

PDZ and LIM domain 4 [Source:HGNC Symbol;Acc:HGNC:16501]

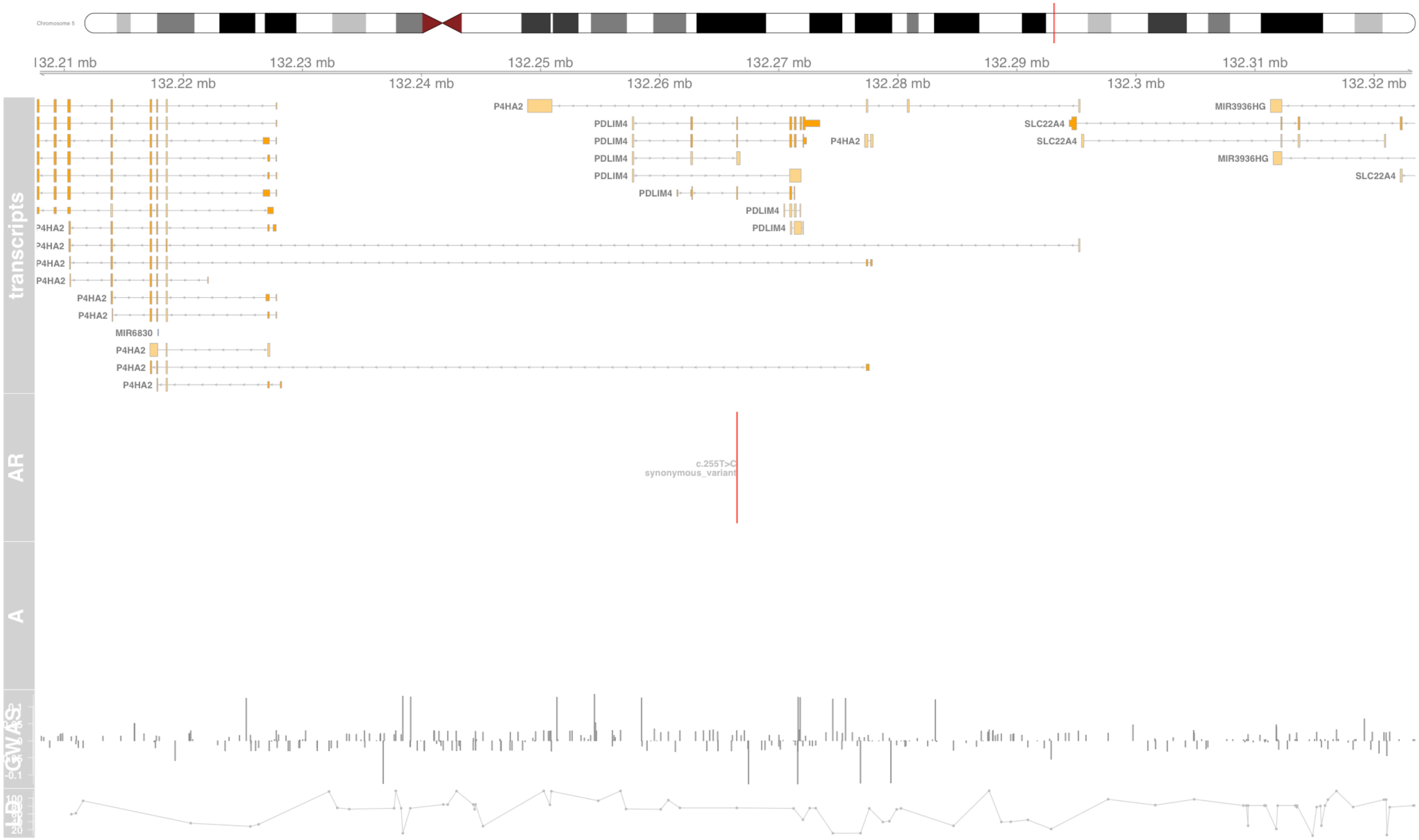

Fig S93 PGAP3

post-GPI attachment to proteins phospholipase 3 [Source:HGNC Symbol;Acc:HGNC:23719]

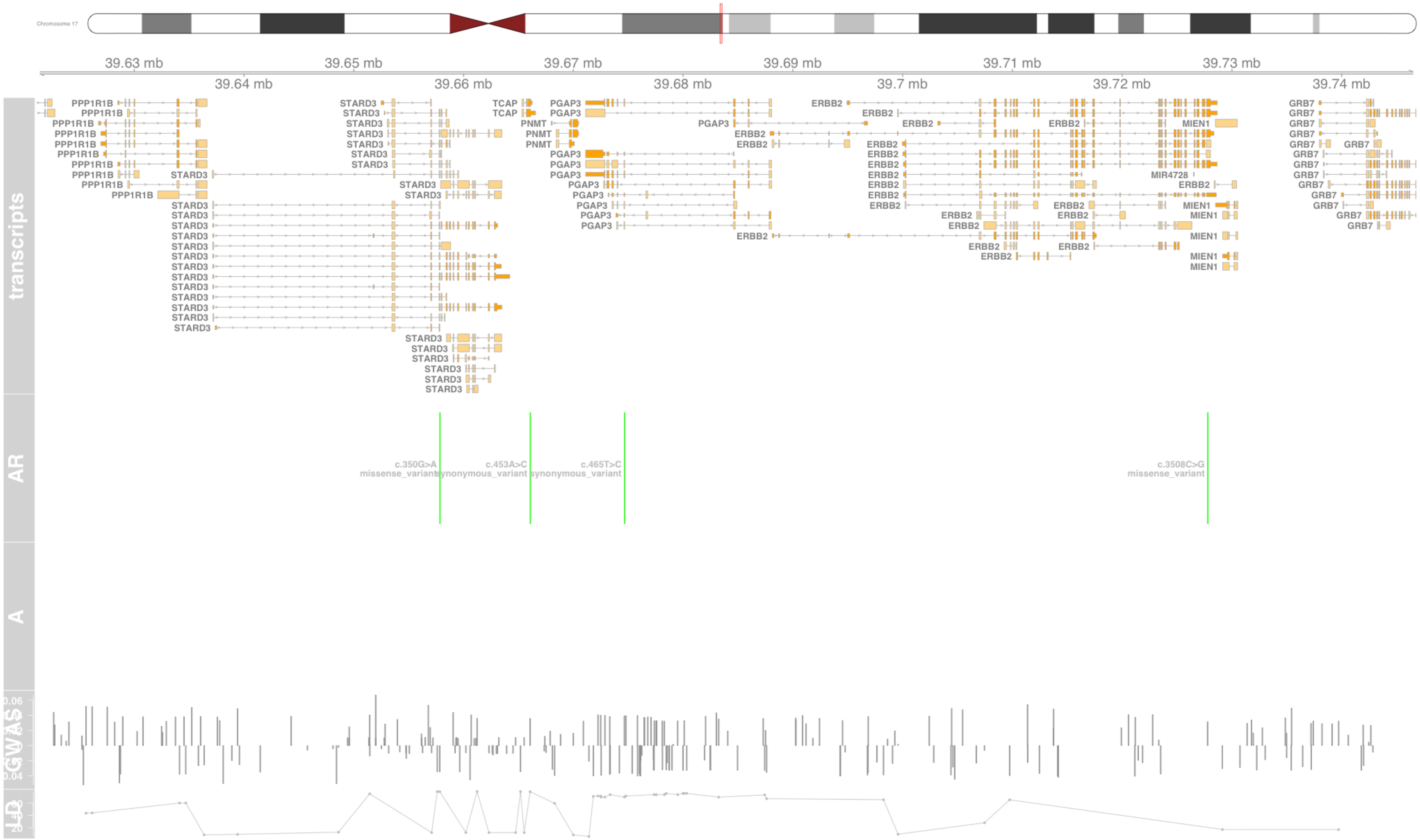

Fig S94 PGBD1

piggyBac transposable element derived 1 [Source:HGNC Symbol;Acc:HGNC:19398]

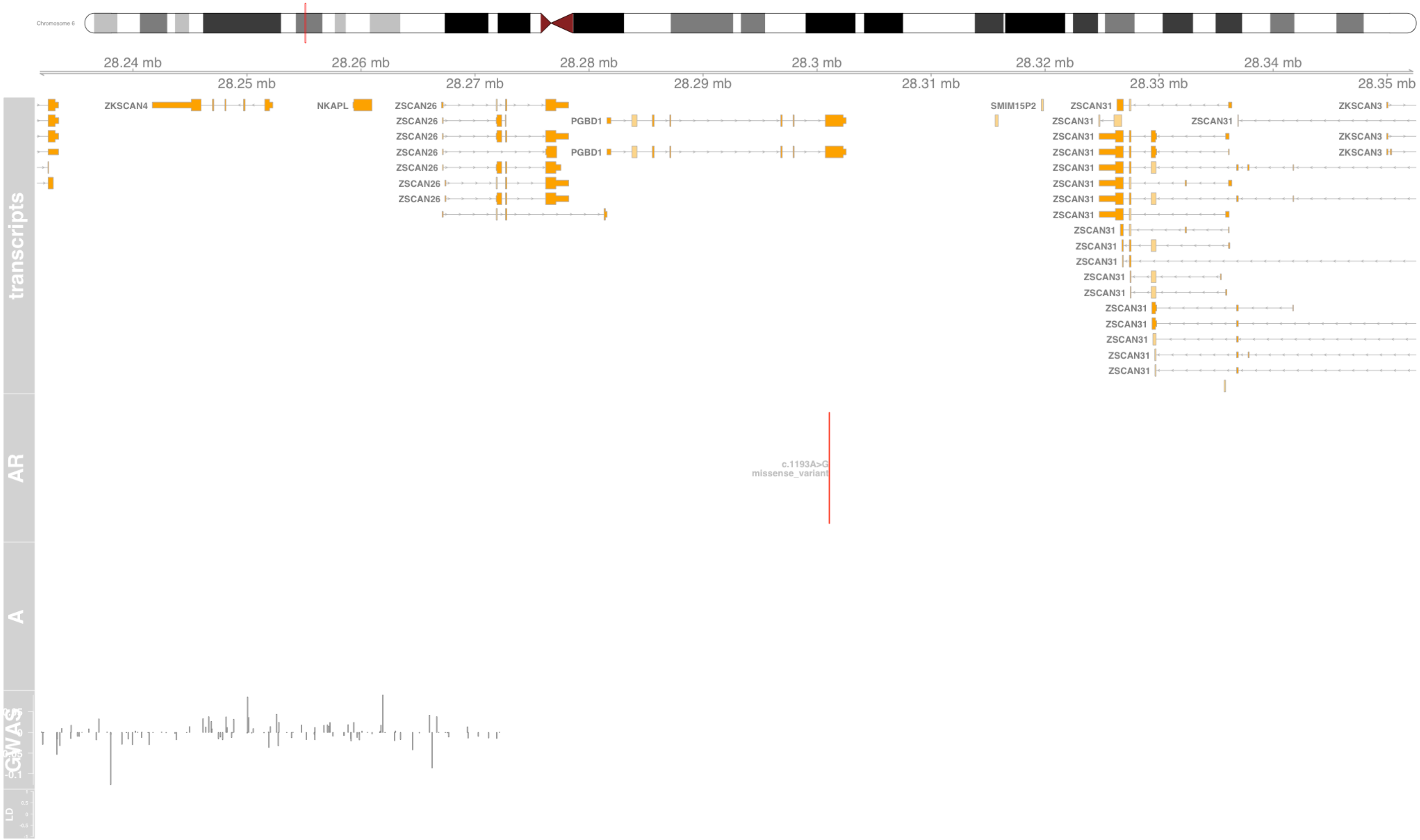

Fig S95 PHB

prohibitin [Source:HGNC Symbol;Acc:HGNC:8912]

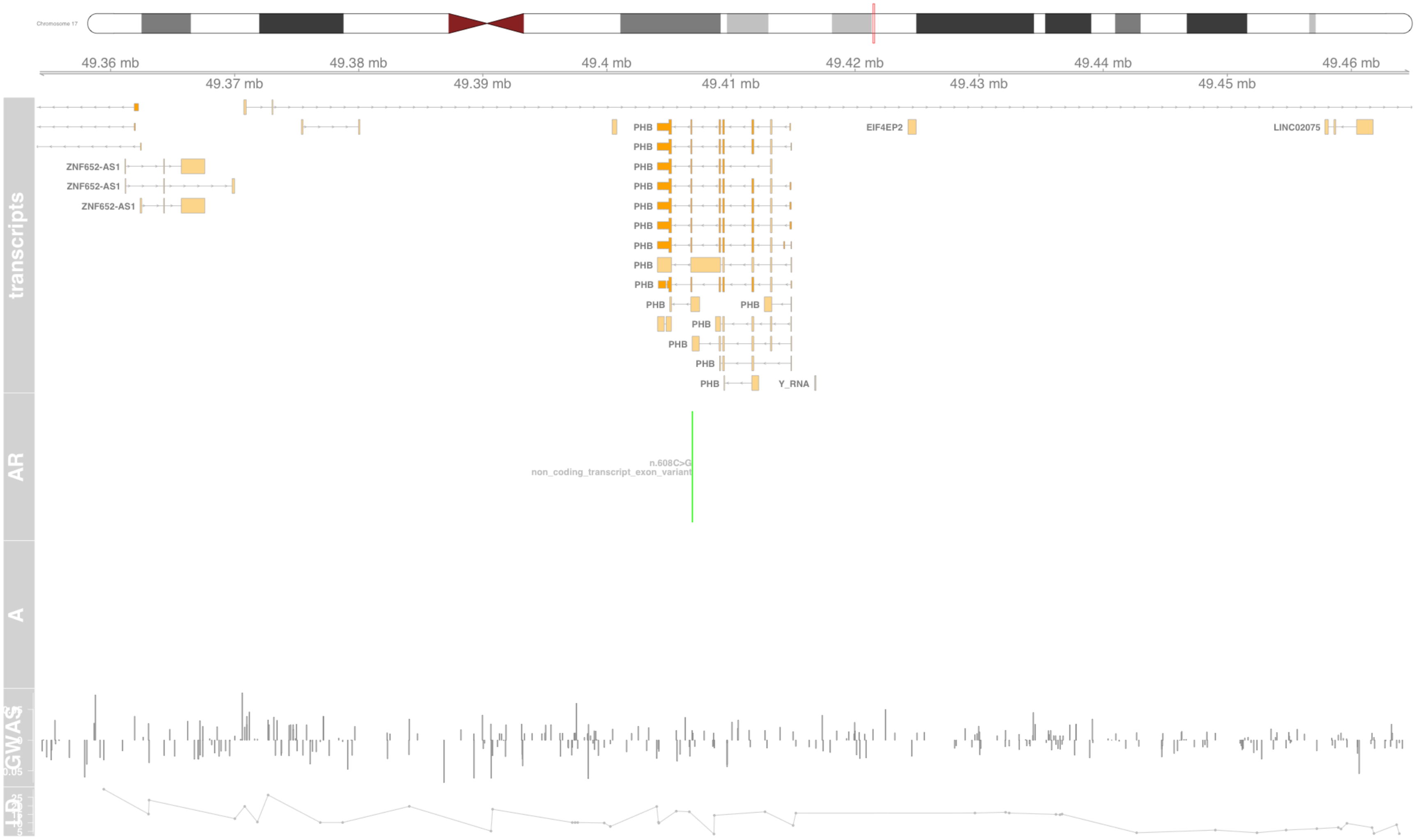

Fig S96 POU5F1

POU class 5 homeobox 1 [Source:HGNC Symbol;Acc:HGNC:9221]

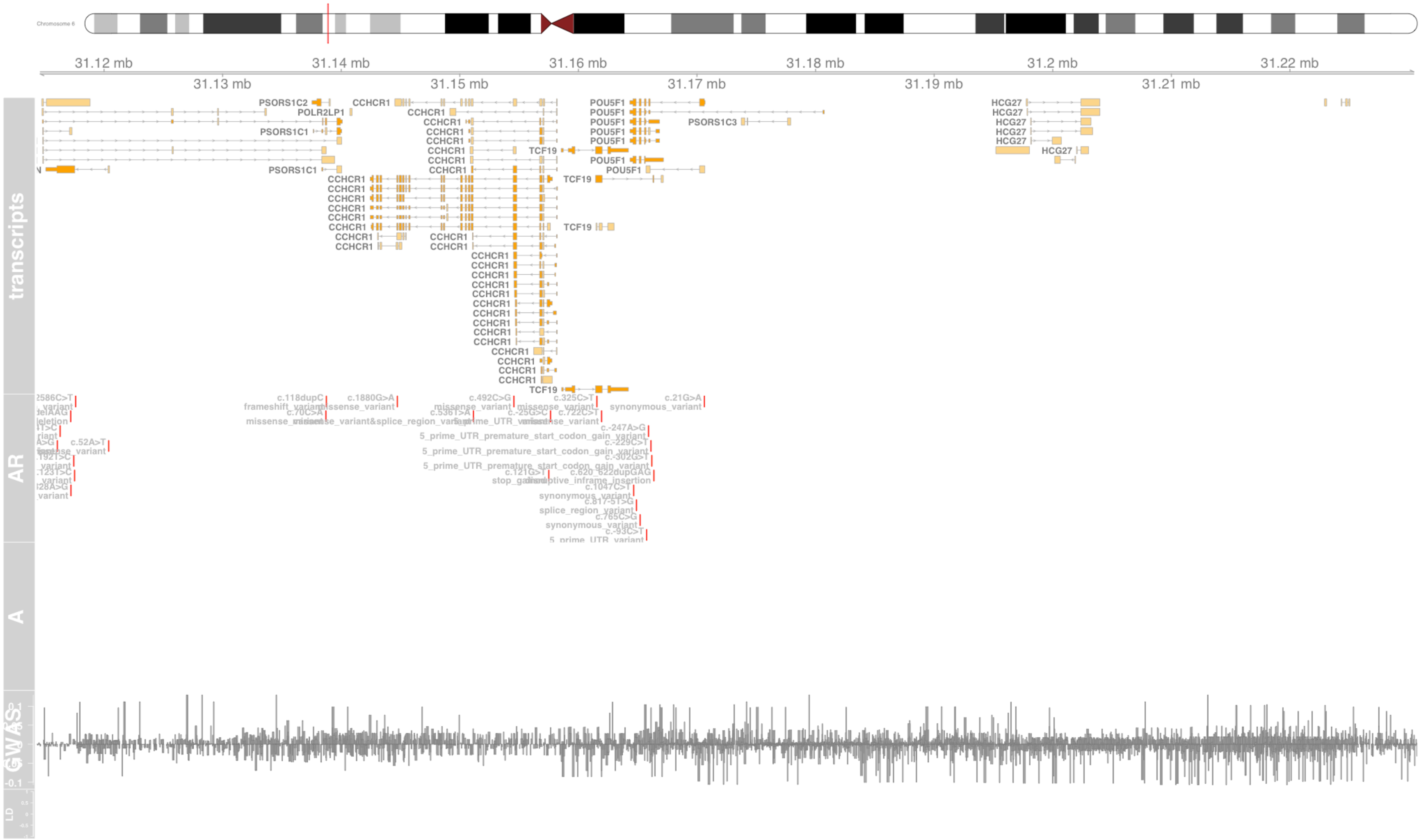

Fig S97 PPP1R18

protein phosphatase 1 regulatory subunit 18 [Source:HGNC Symbol;Acc:HGNC:29413]

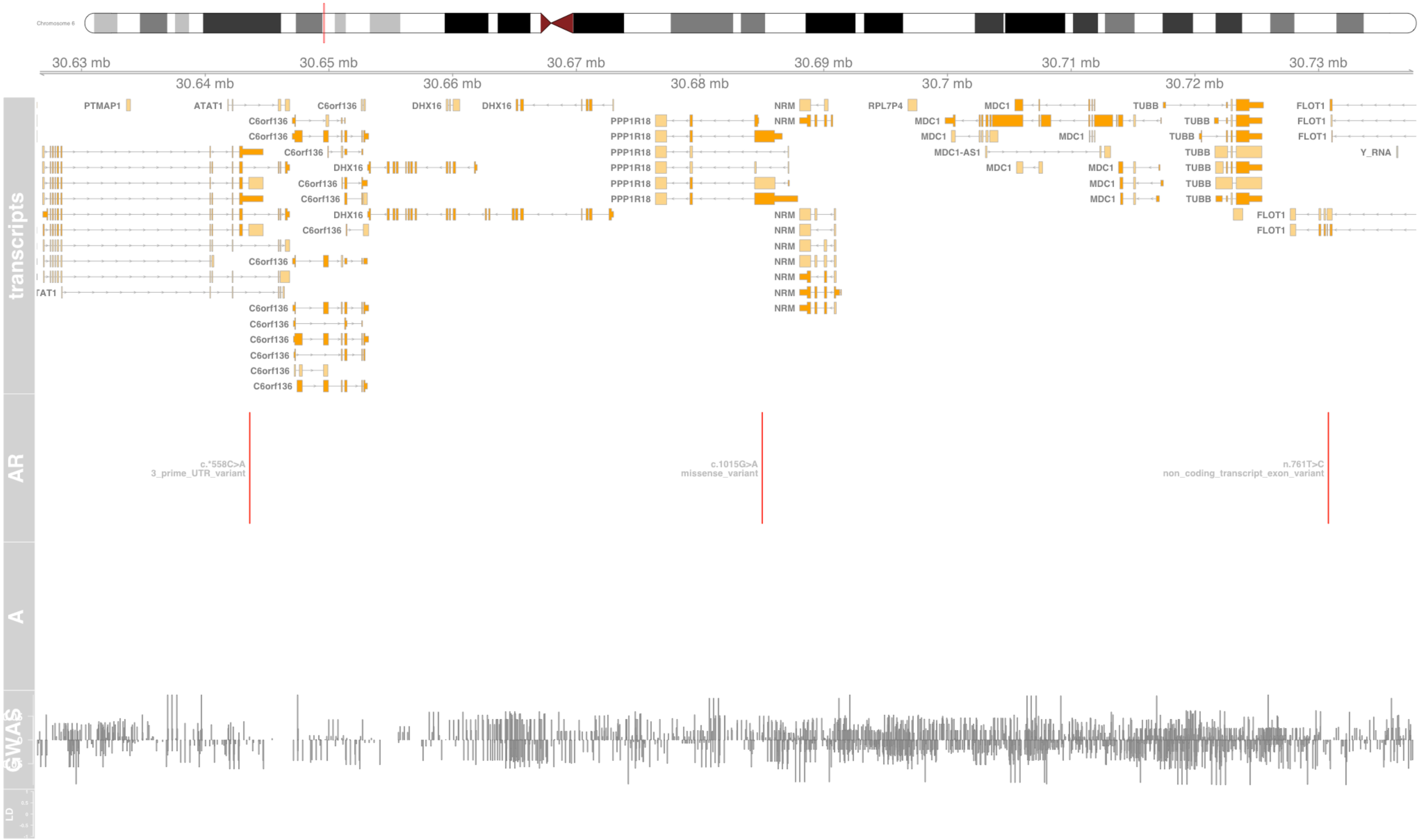

Fig S98 PPT2

palmitoyl-protein thioesterase 2 [Source:HGNC Symbol;Acc:HGNC:9326]

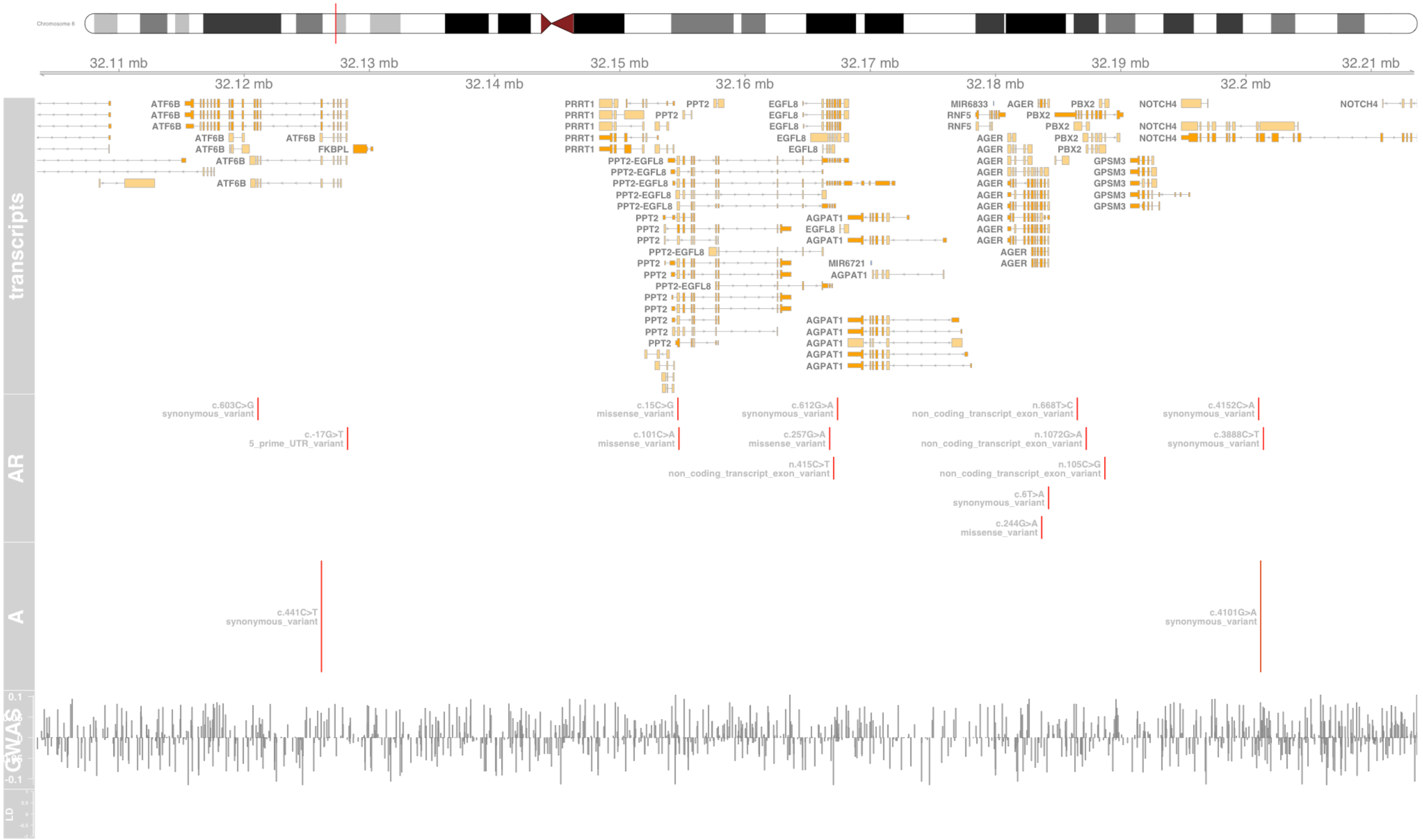

Fig S99 PRRC2A

proline rich coiled-coil 2A [Source:HGNC Symbol;Acc:HGNC:13918]

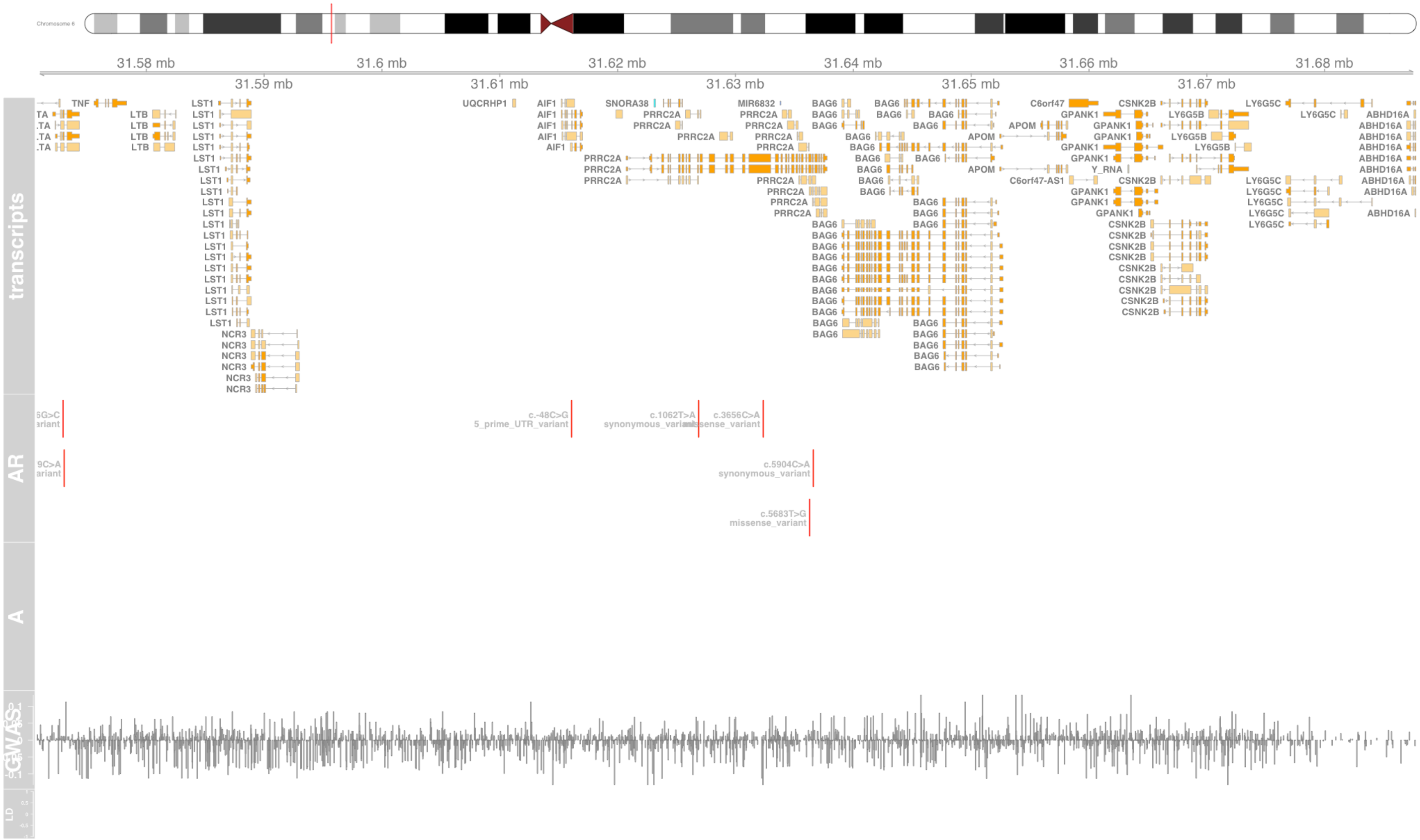

Fig S100 PSMB8

proteasome 20S subunit beta 8 [Source:HGNC Symbol;Acc:HGNC:9545]

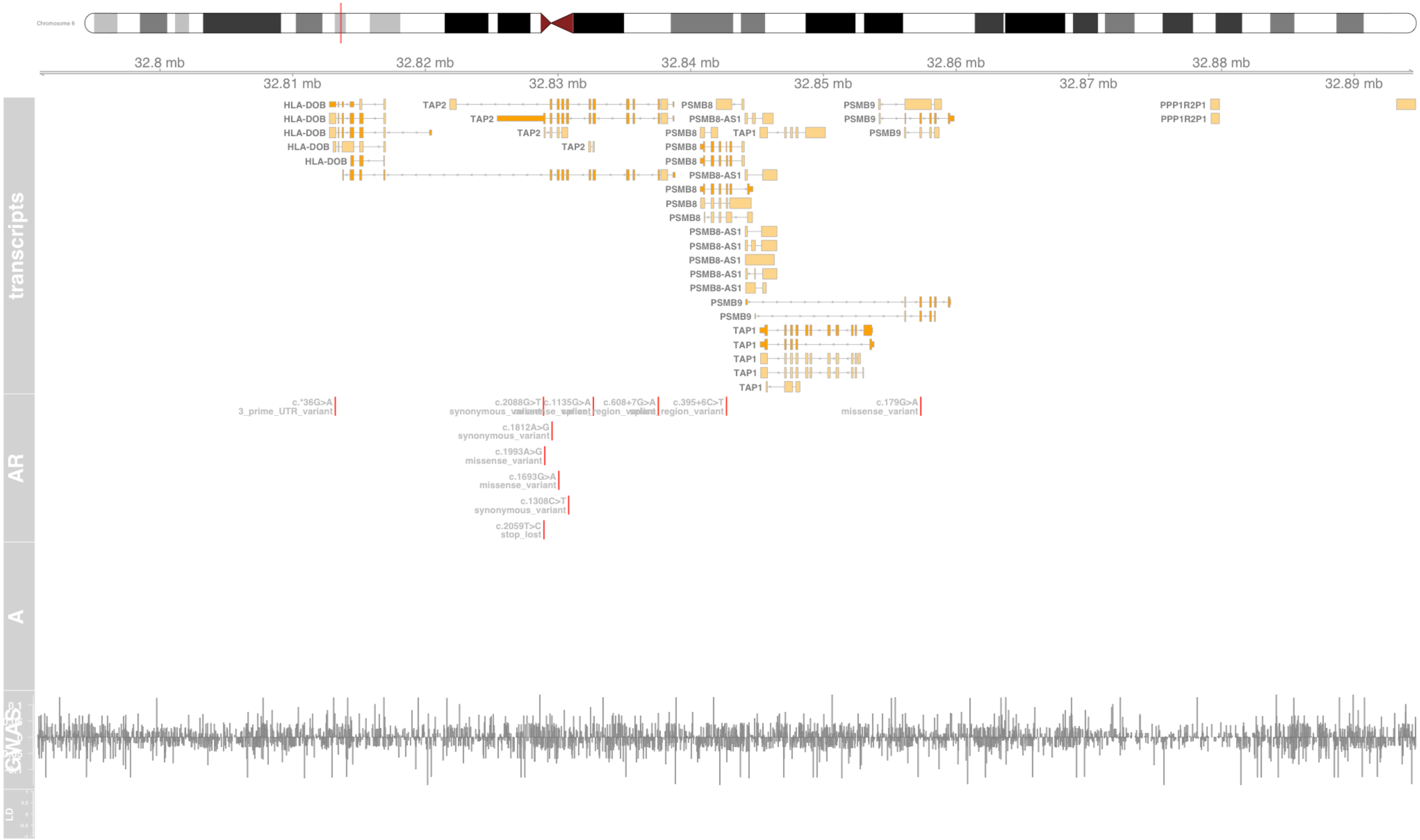

Fig S101 PSMB9

proteasome 20S subunit beta 9 [Source:HGNC Symbol;Acc:HGNC:9546]

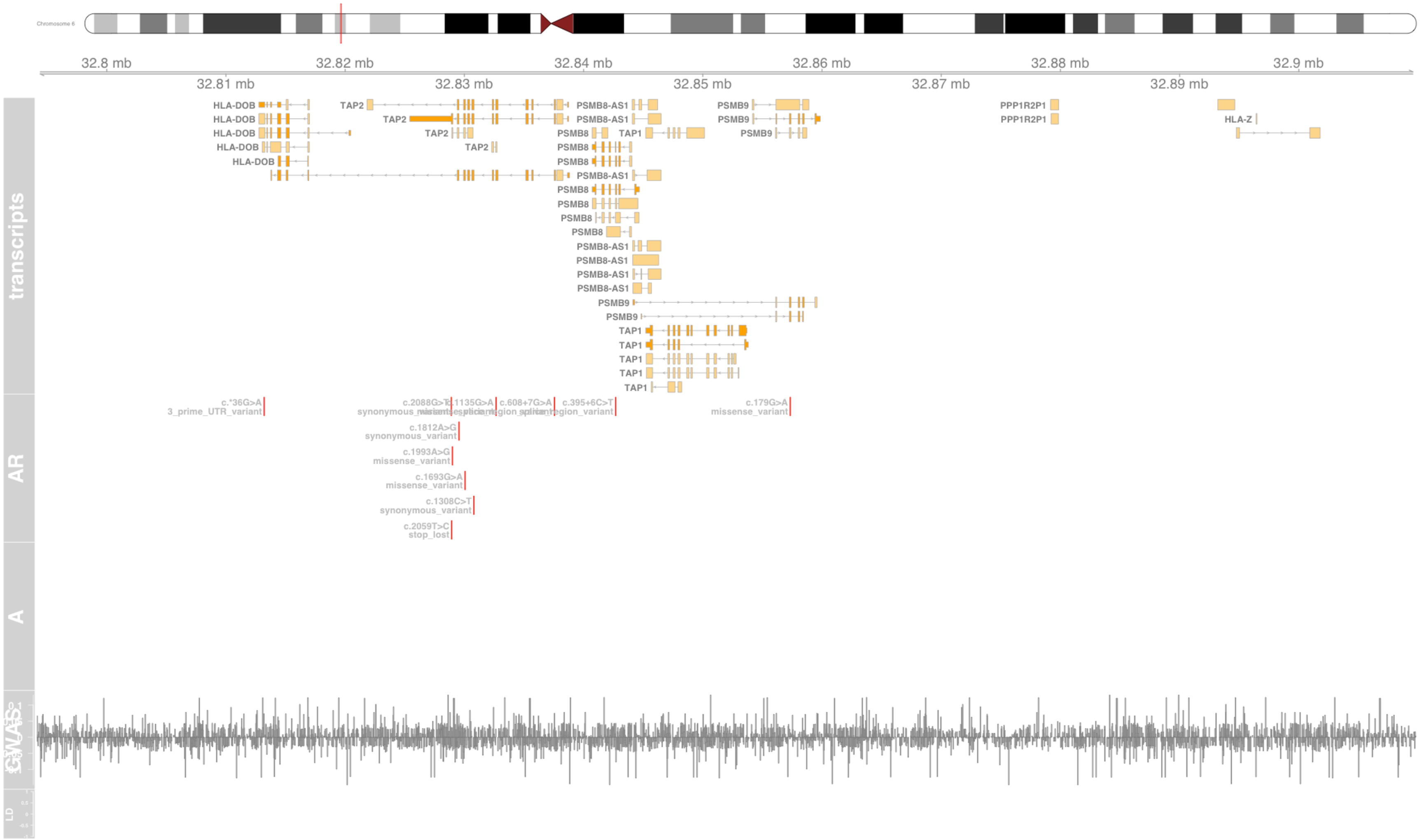

Fig S102 PSMD3

proteasome 26S subunit, non-ATPase 3 [Source:HGNC Symbol;Acc:HGNC:9560]

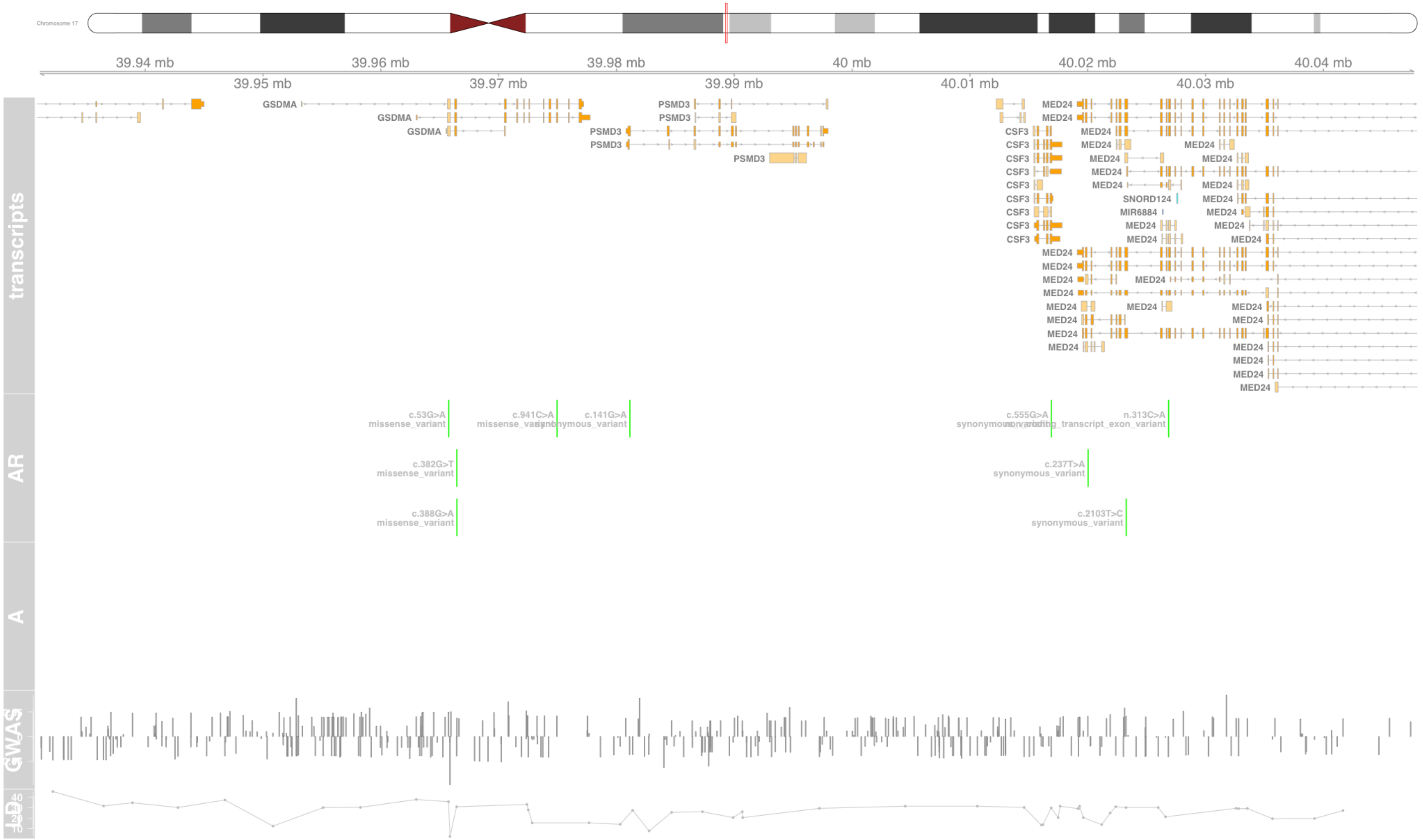

Fig S103 PSORS1C1

psoriasis susceptibility 1 candidate 1 [Source:HGNC Symbol;Acc:HGNC:17202]

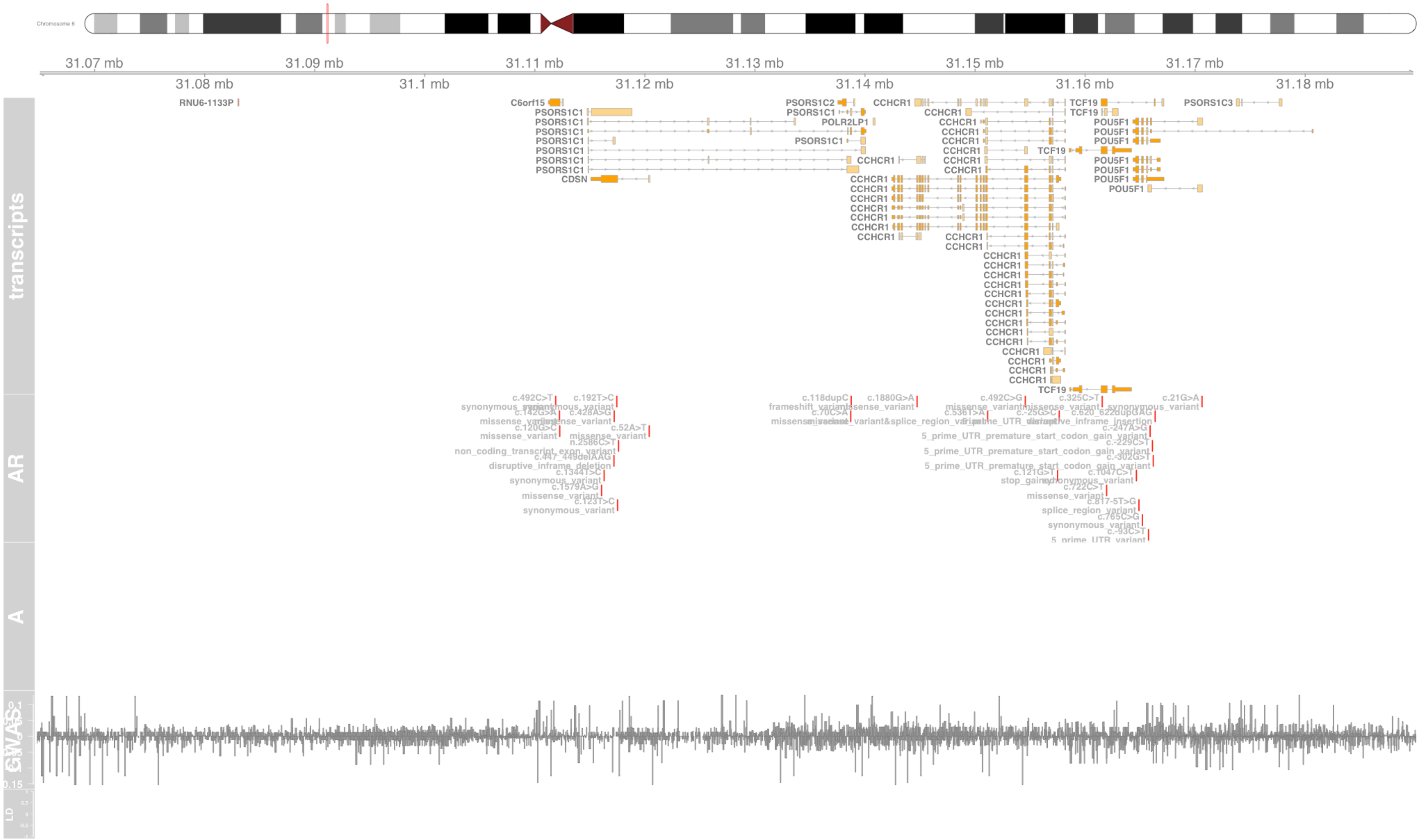

Fig S104 RPS26

ribosomal protein S26 [Source:HGNC Symbol;Acc:HGNC:10414]

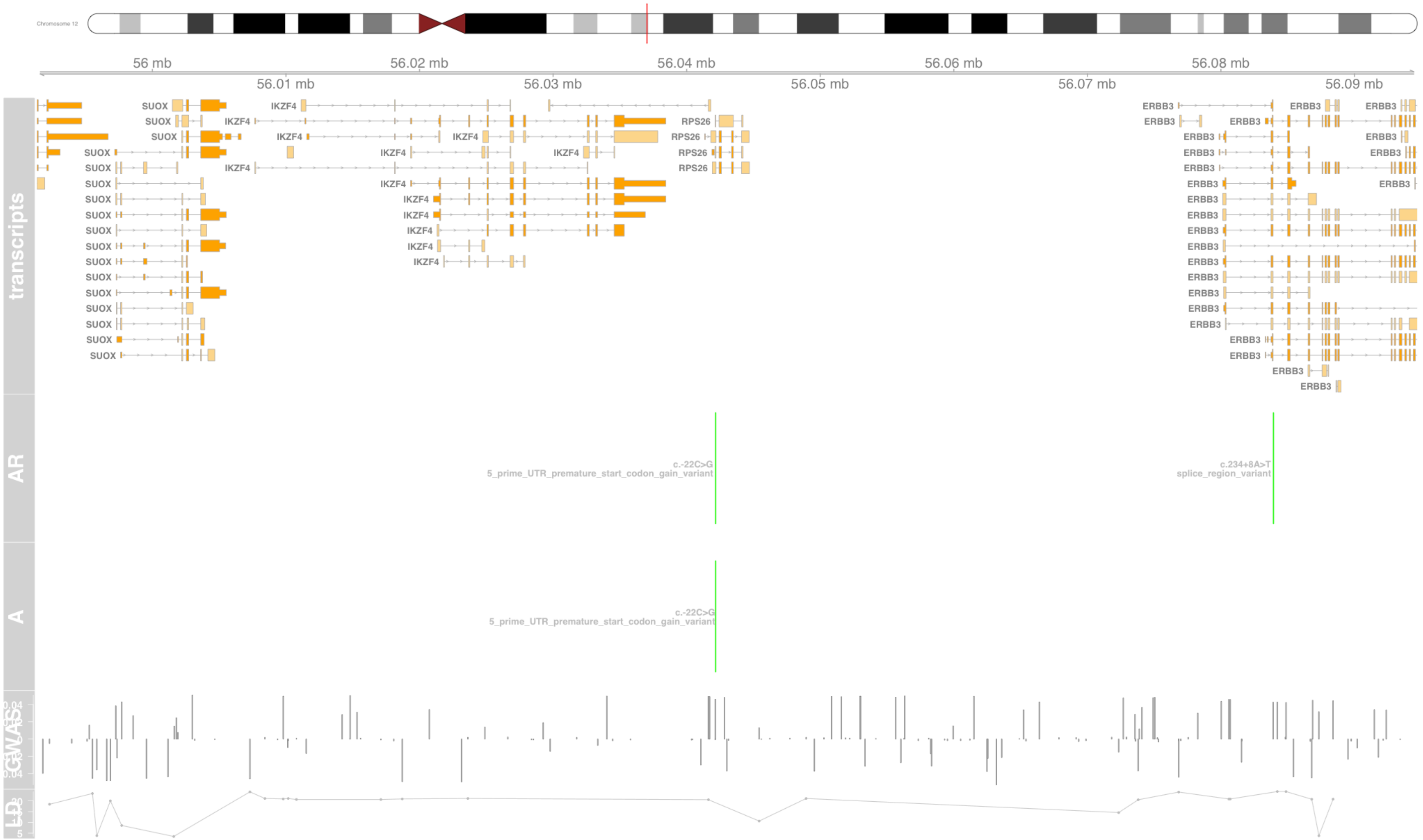

Fig S105 SKIV2L

Ski2 like RNA helicase [Source:HGNC Symbol;Acc:HGNC:10898]

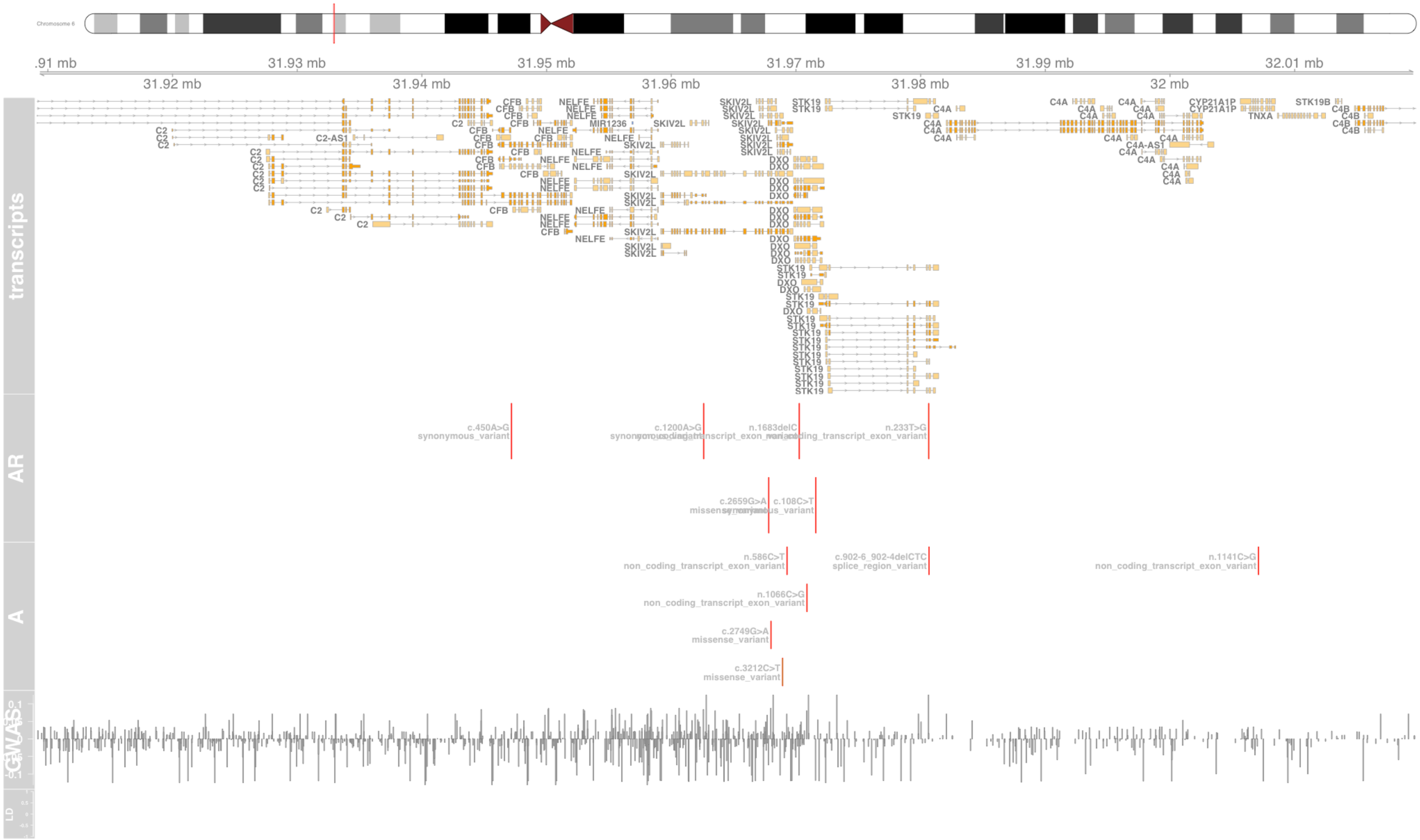

Fig S106 SLC22A4

solute carrier family 22 member 4 [Source:HGNC Symbol;Acc:HGNC:10968]

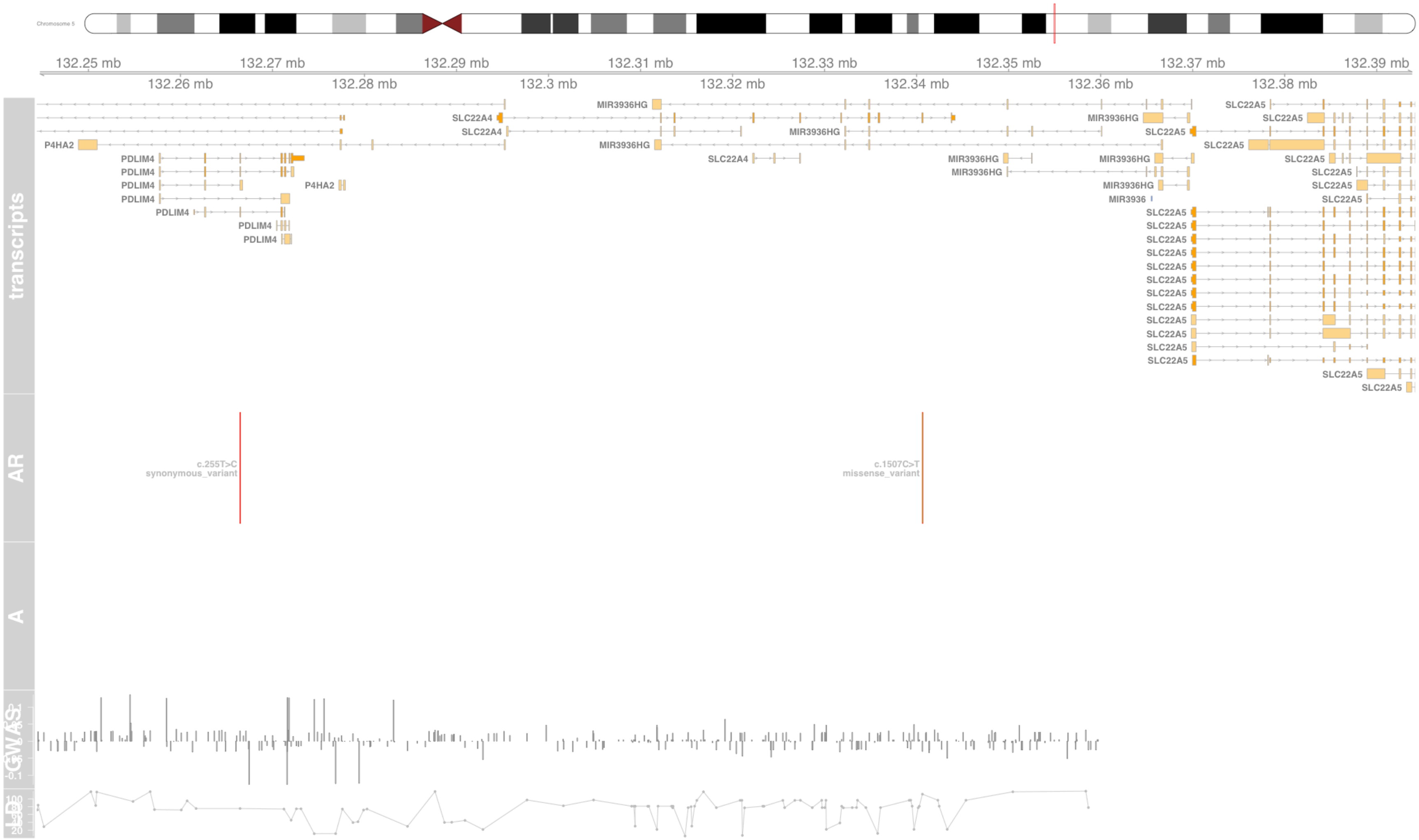

Fig S107 STARD3

StAR related lipid transfer domain containing 3 [Source:HGNC Symbol;Acc:HGNC:17579]

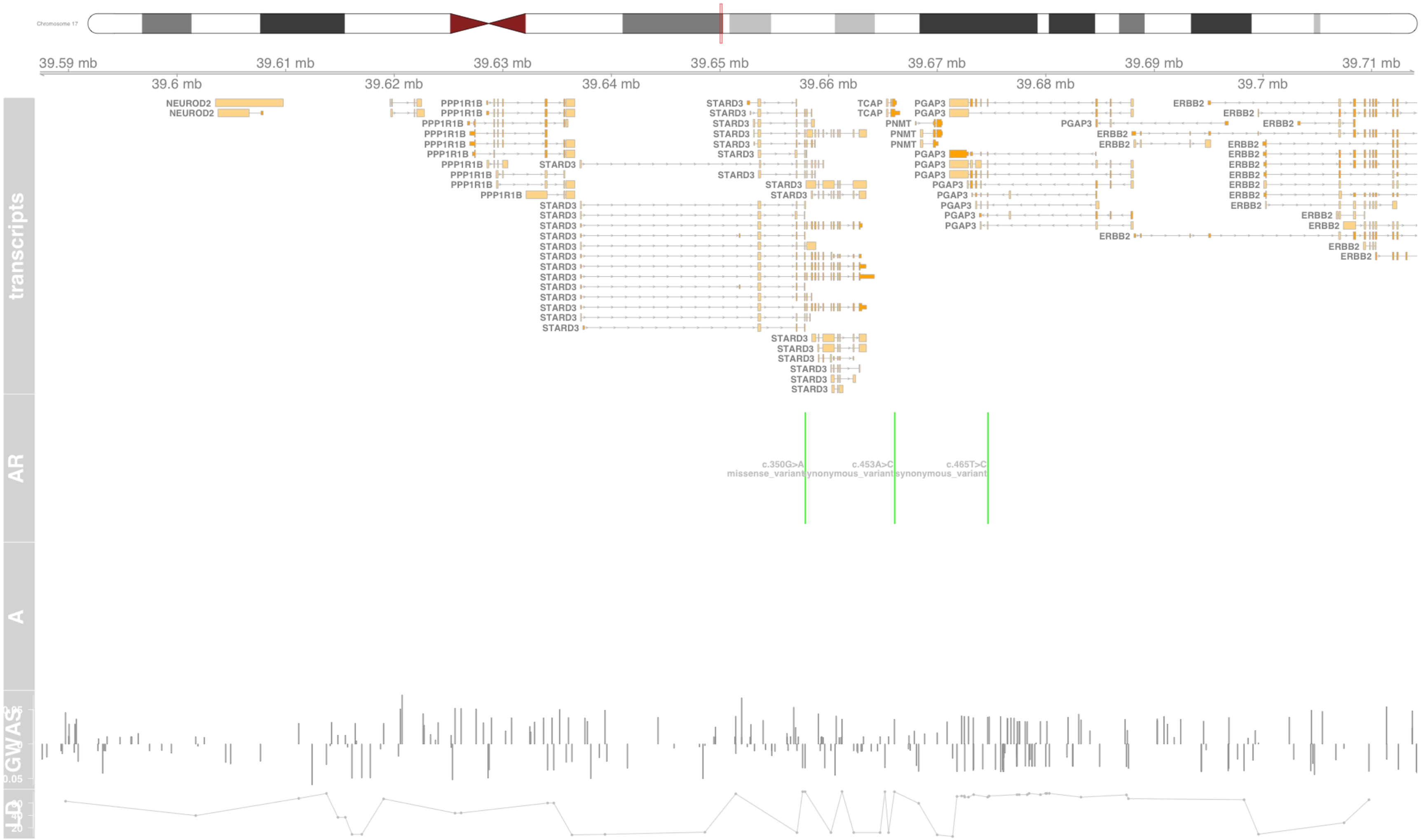

Fig S108 STAT6

signal transducer and activator of transcription 6 [Source:HGNC Symbol;Acc:HGNC:11368]

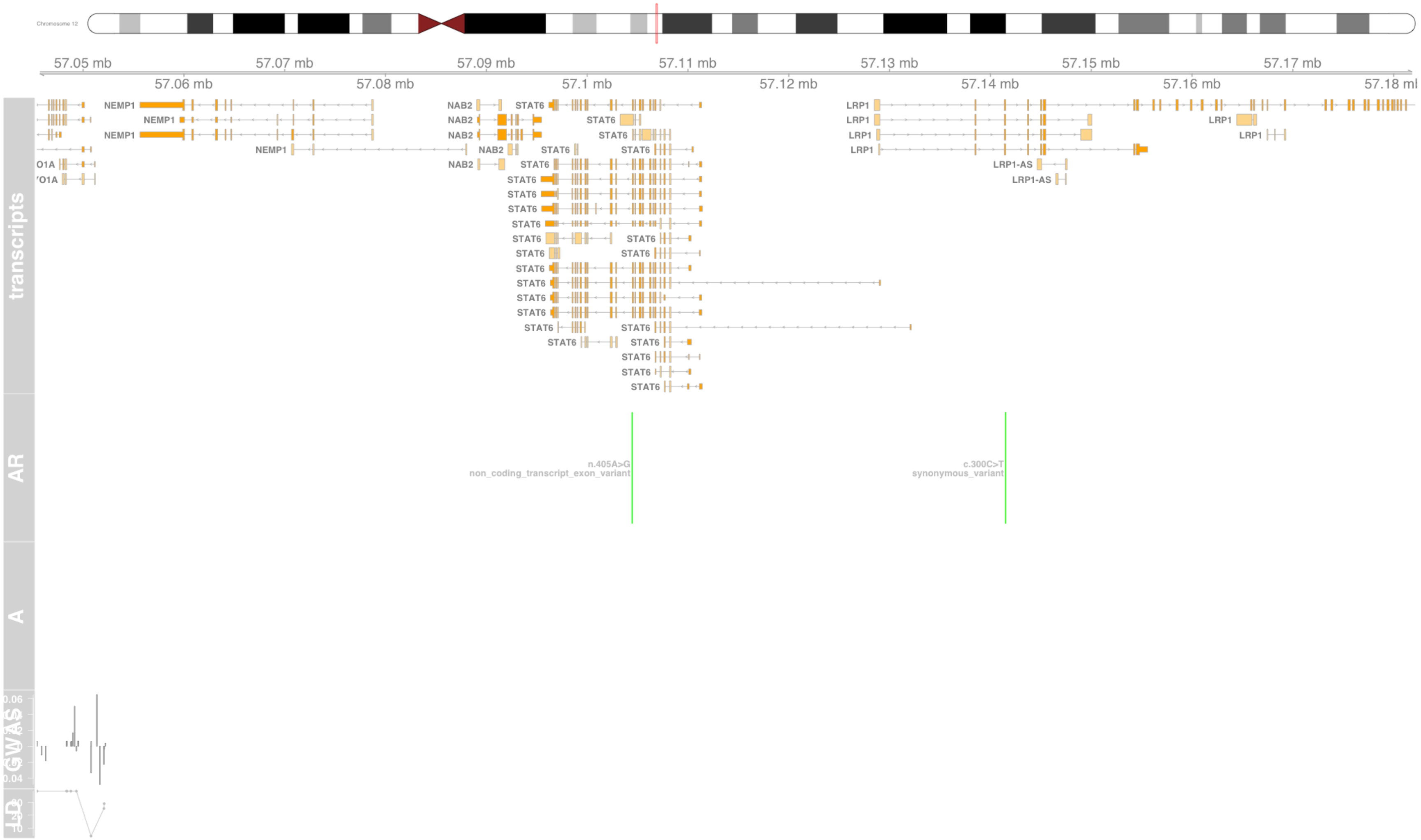

Fig S109 STK19

serine/threonine kinase 19 [Source:HGNC Symbol;Acc:HGNC:11398]

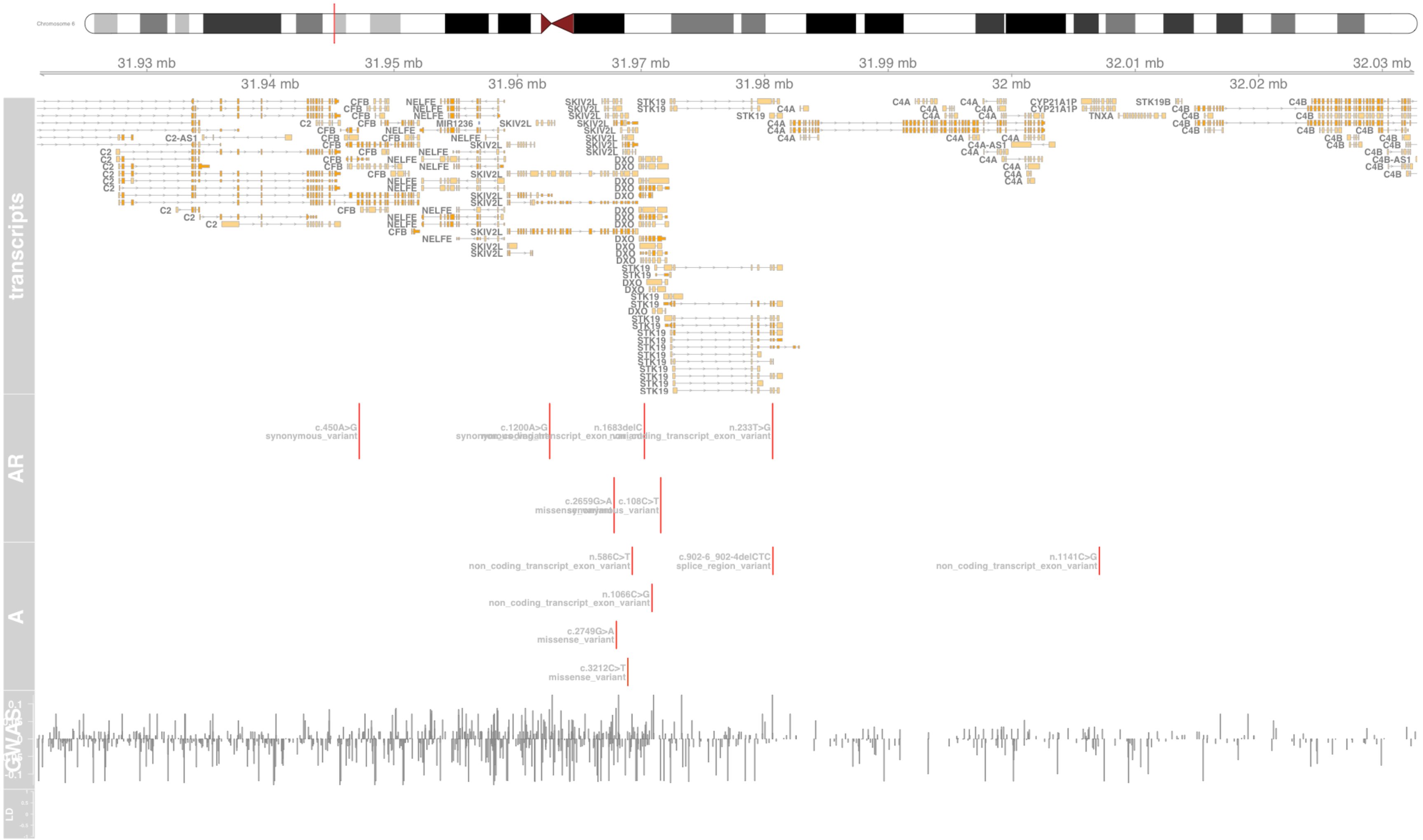

Fig S110 TAP2

transporter 2, ATP binding cassette subfamily B member [Source:HGNC Symbol;Acc:HGNC:44]

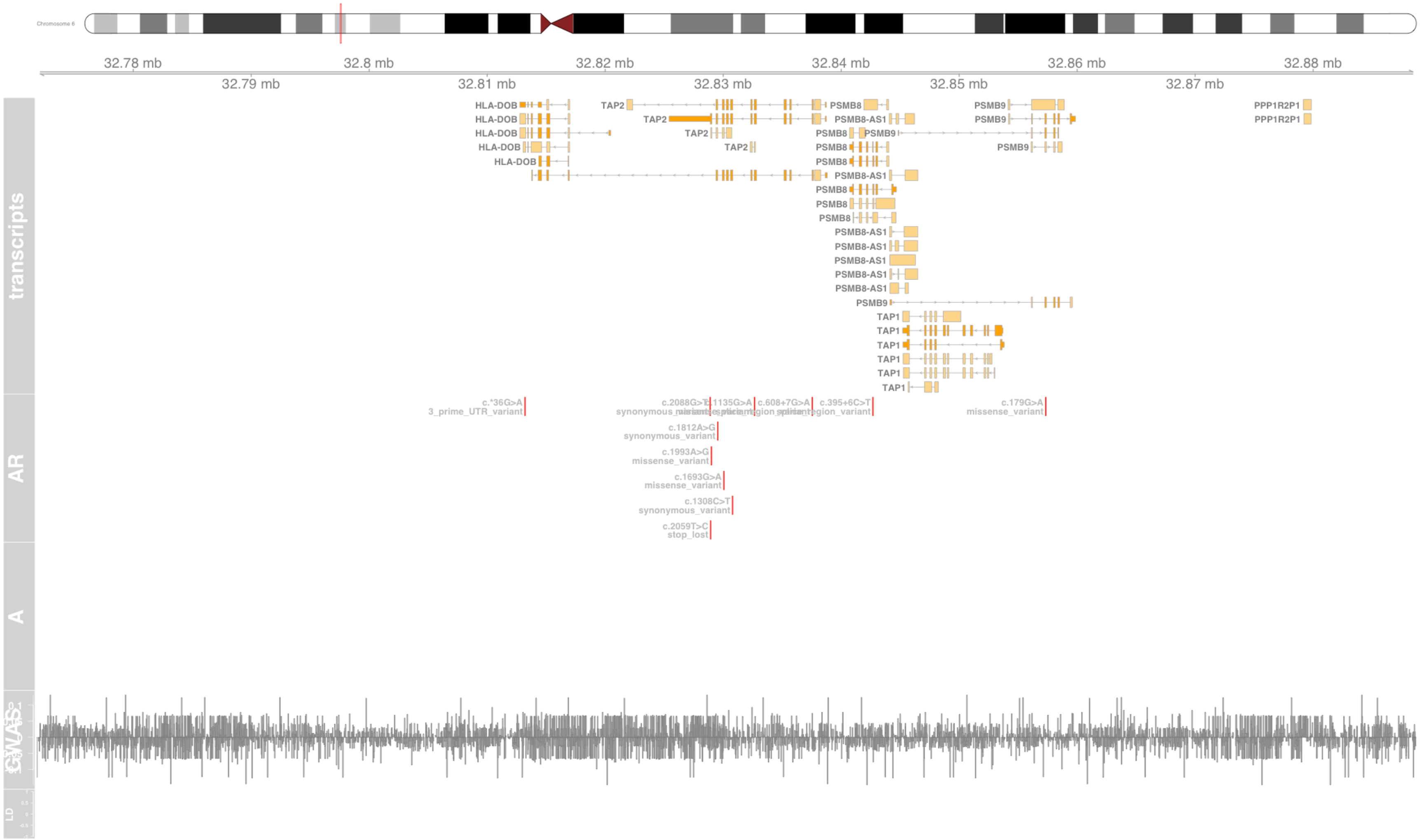

Fig S111 TCAP

titin-cap [Source:HGNC Symbol;Acc:HGNC:11610]

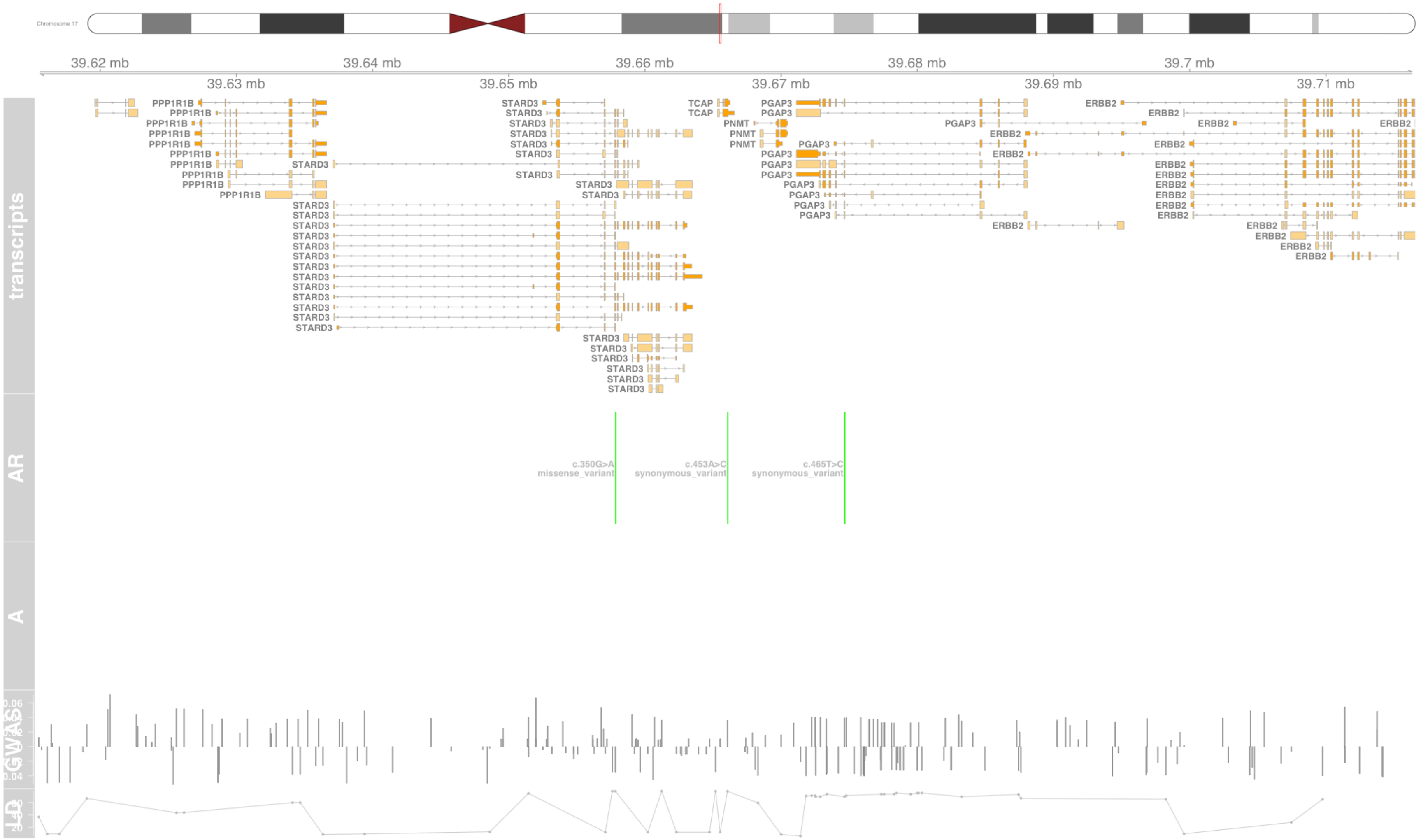

Fig S112 TCF19

transcription factor 19 [Source:HGNC Symbol;Acc:HGNC:11629]

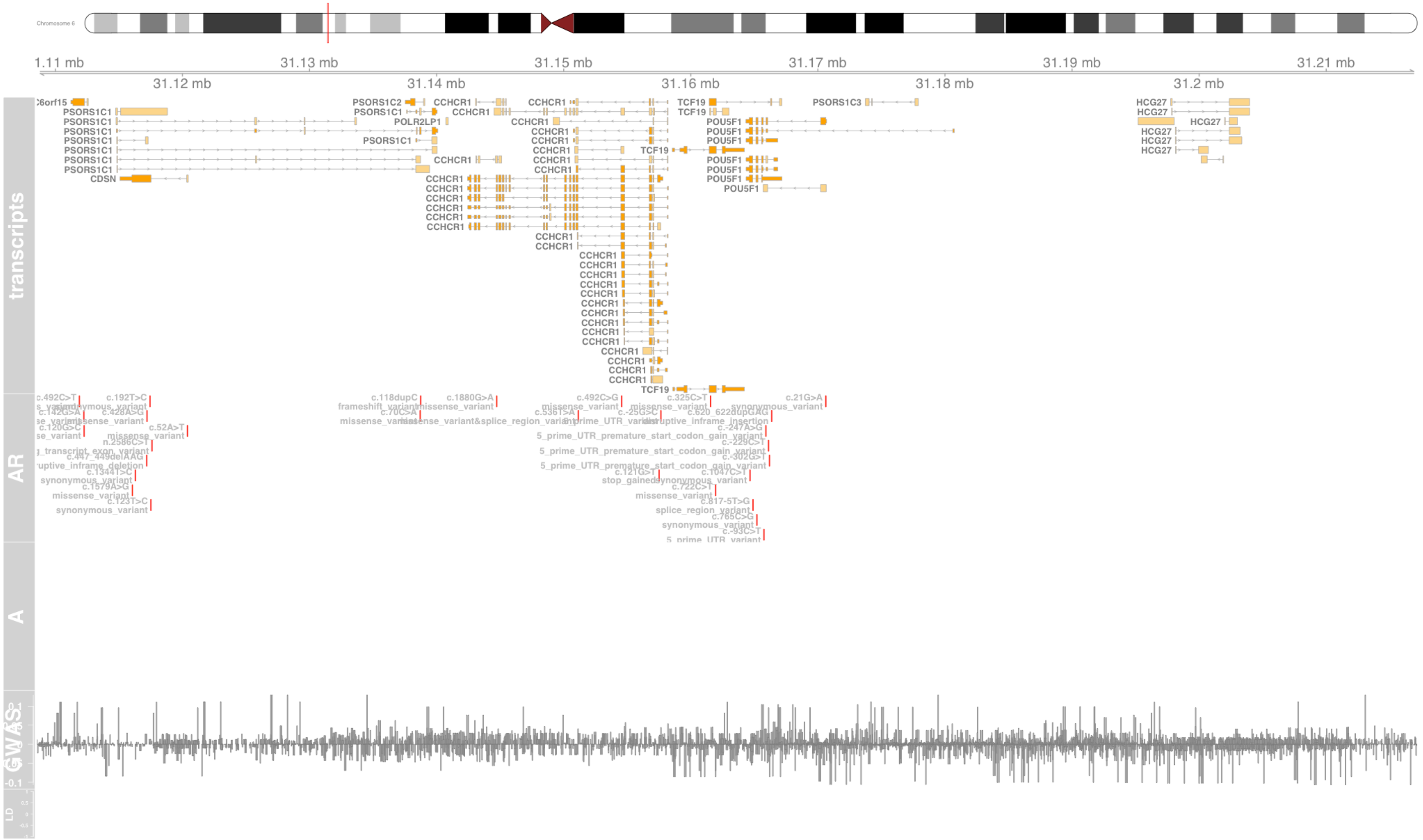

Fig S113 TLR1

toll like receptor 1 [Source:HGNC Symbol;Acc:HGNC:11847]

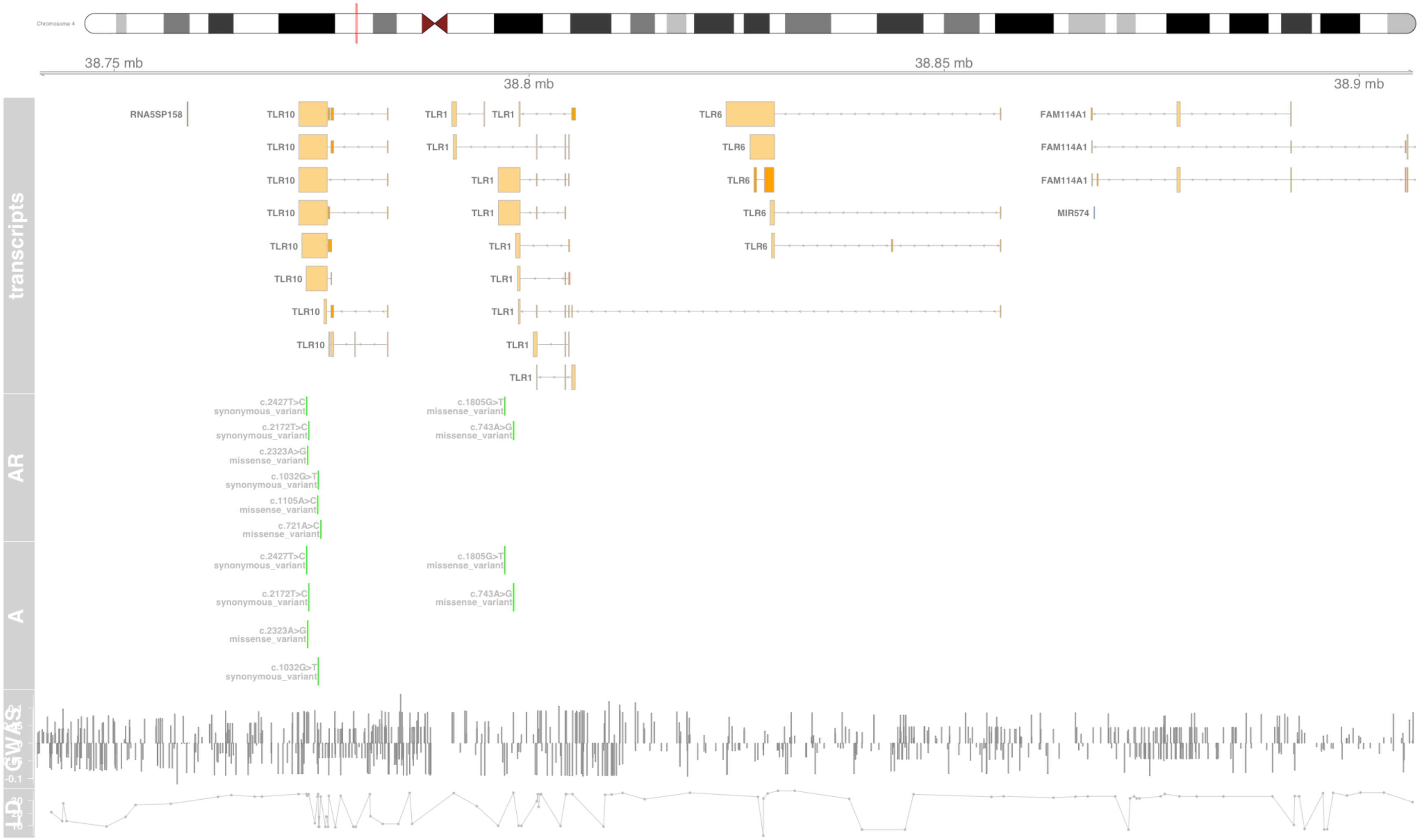

Fig S114 TLR10

toll like receptor 10 [Source:HGNC Symbol;Acc:HGNC:15634]

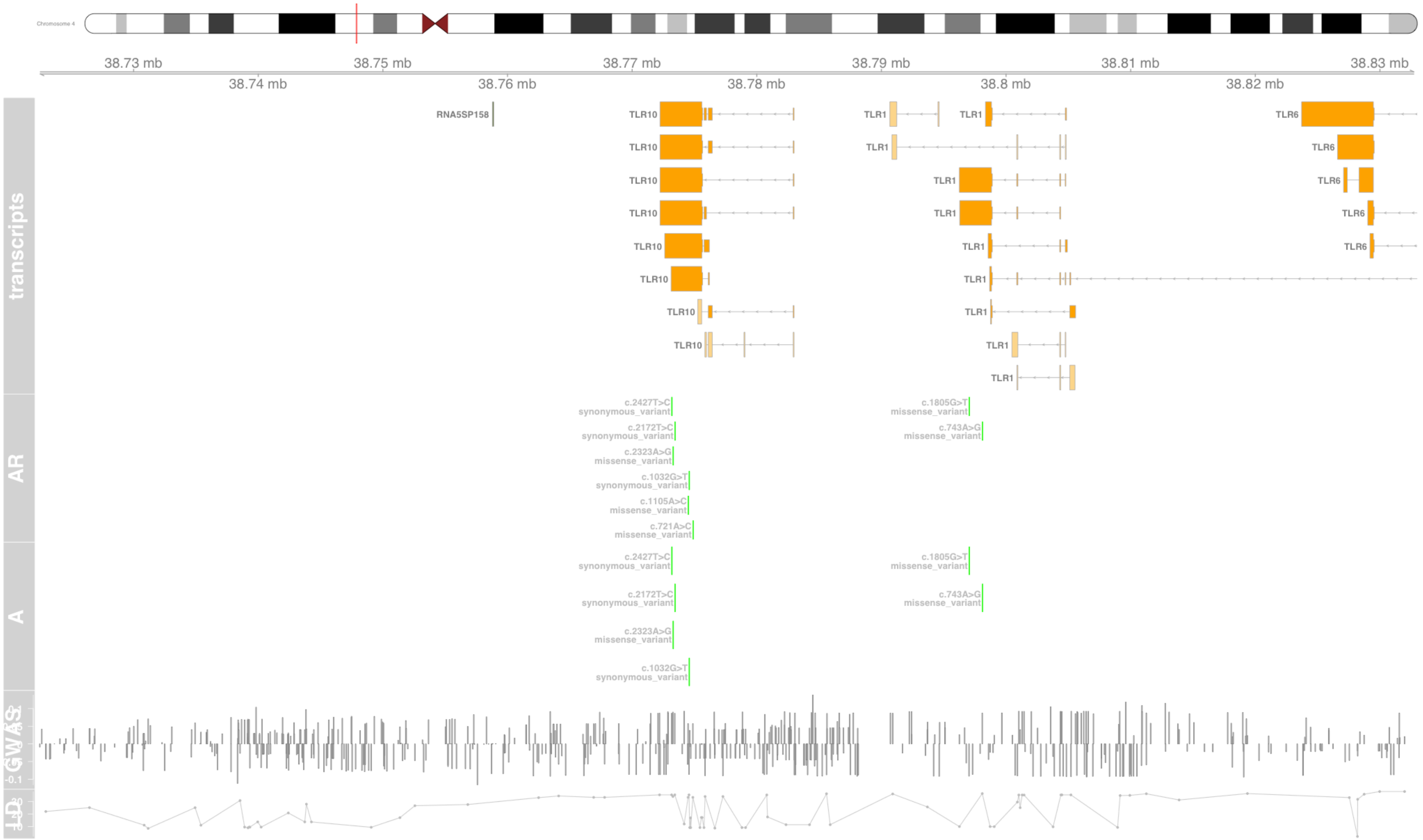

Fig S115 TNXB

tenascin XB [Source:HGNC Symbol;Acc:HGNC:11976]

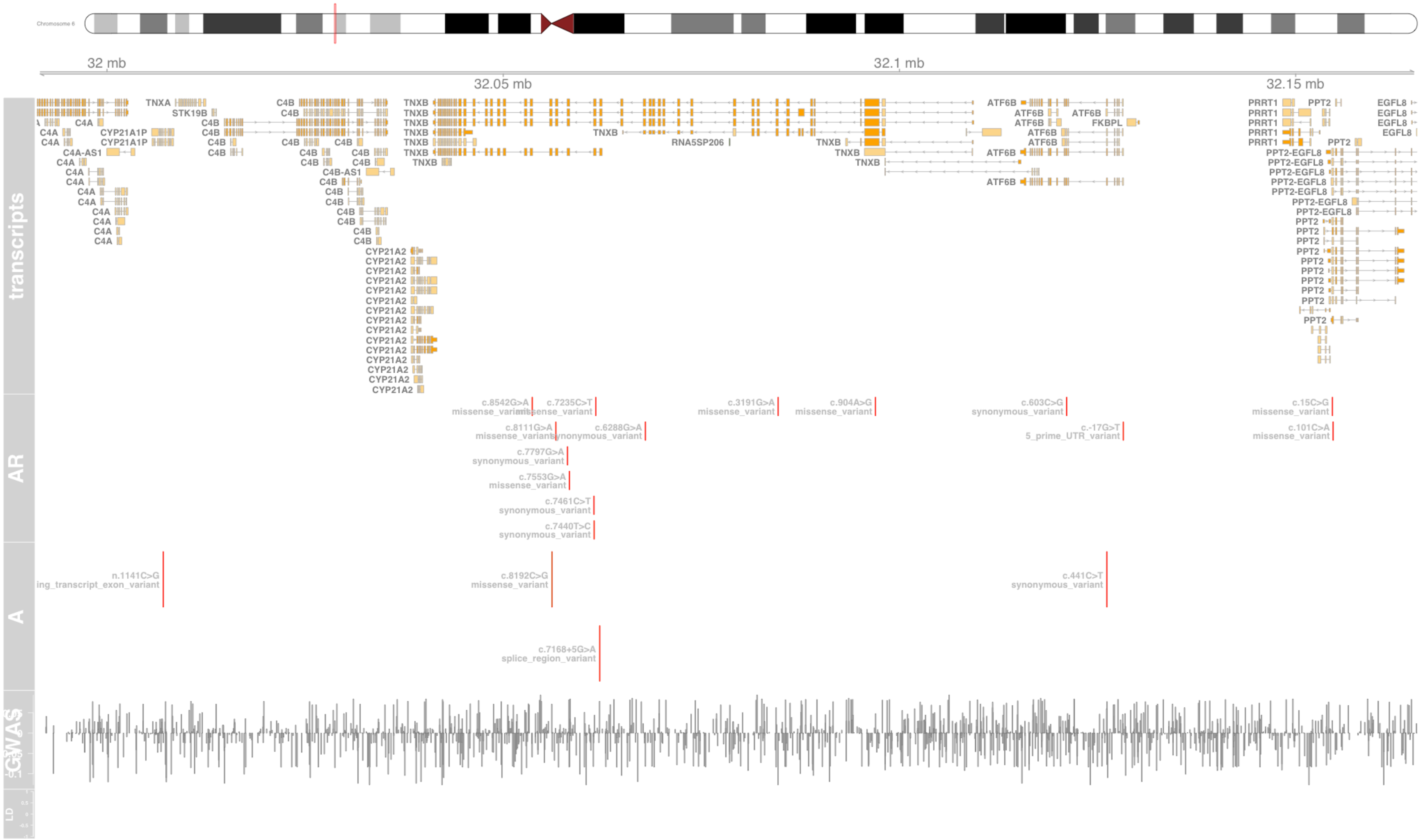

Fig S116 TOB2

transducer of ERBB2, 2 [Source:HGNC Symbol;Acc:HGNC:11980]

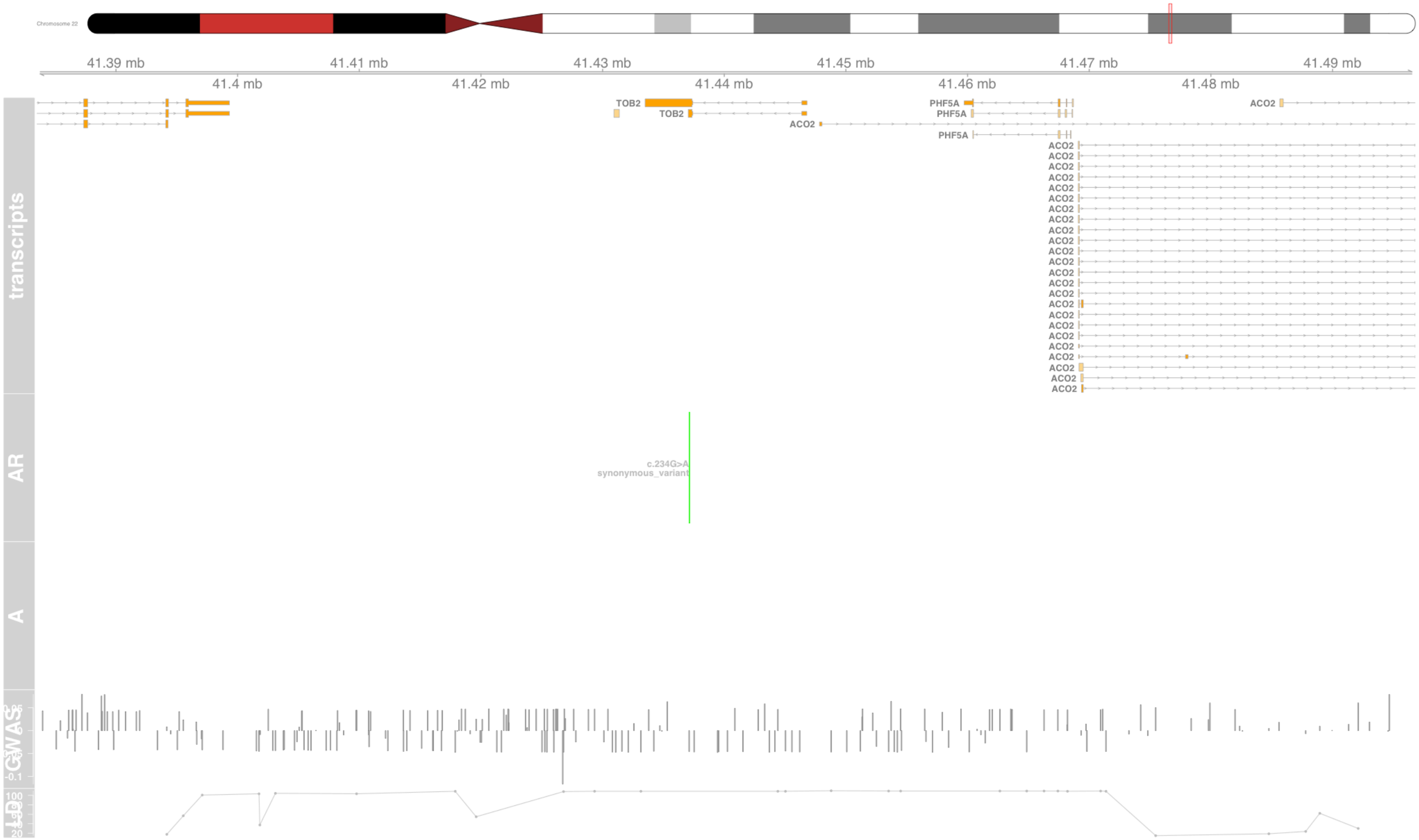

Fig S117 TRIM26

tripartite motif containing 26 [Source:HGNC Symbol;Acc:HGNC:12962]

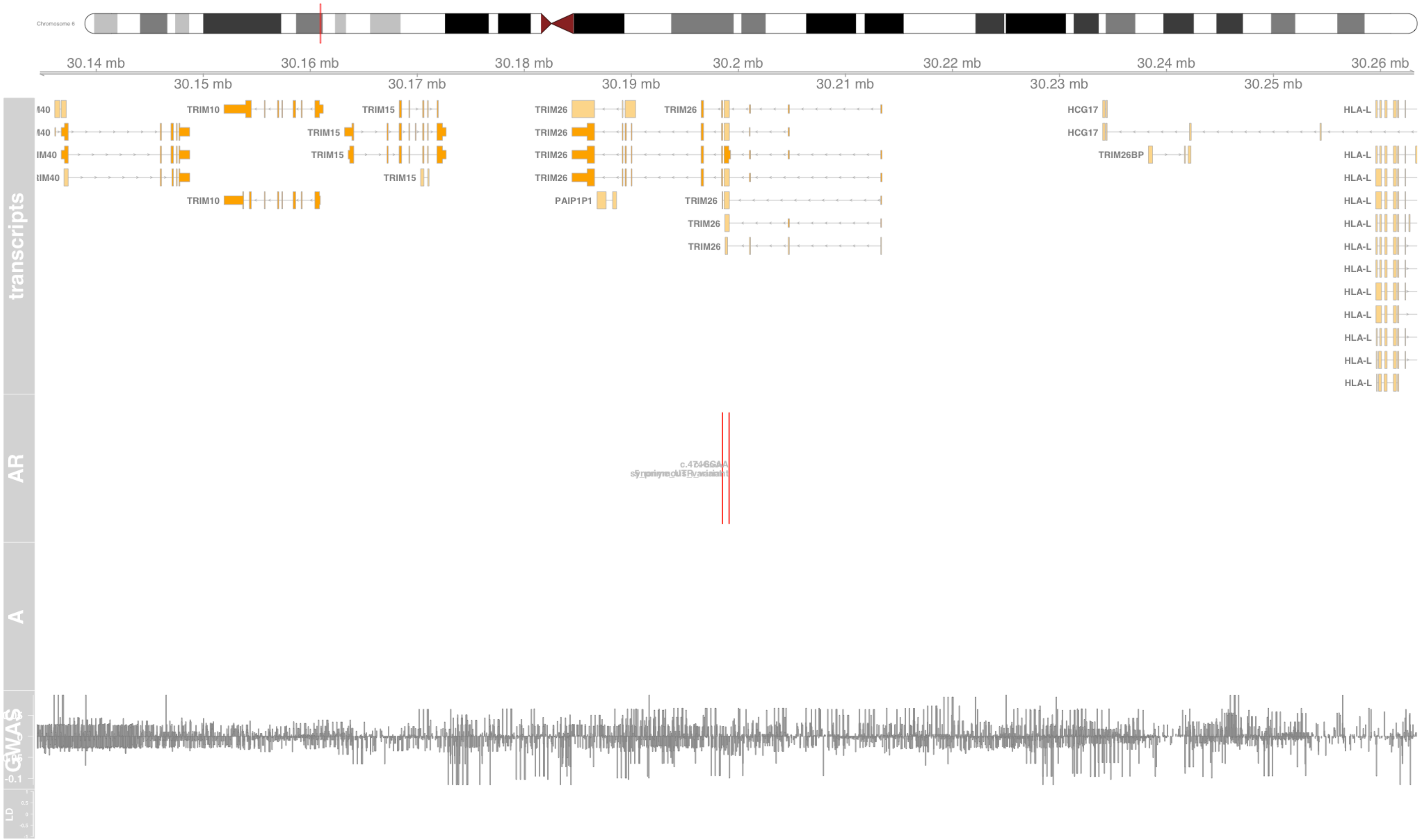

Fig S118 TRIM27

tripartite motif containing 27 [Source:HGNC Symbol;Acc:HGNC:9975]

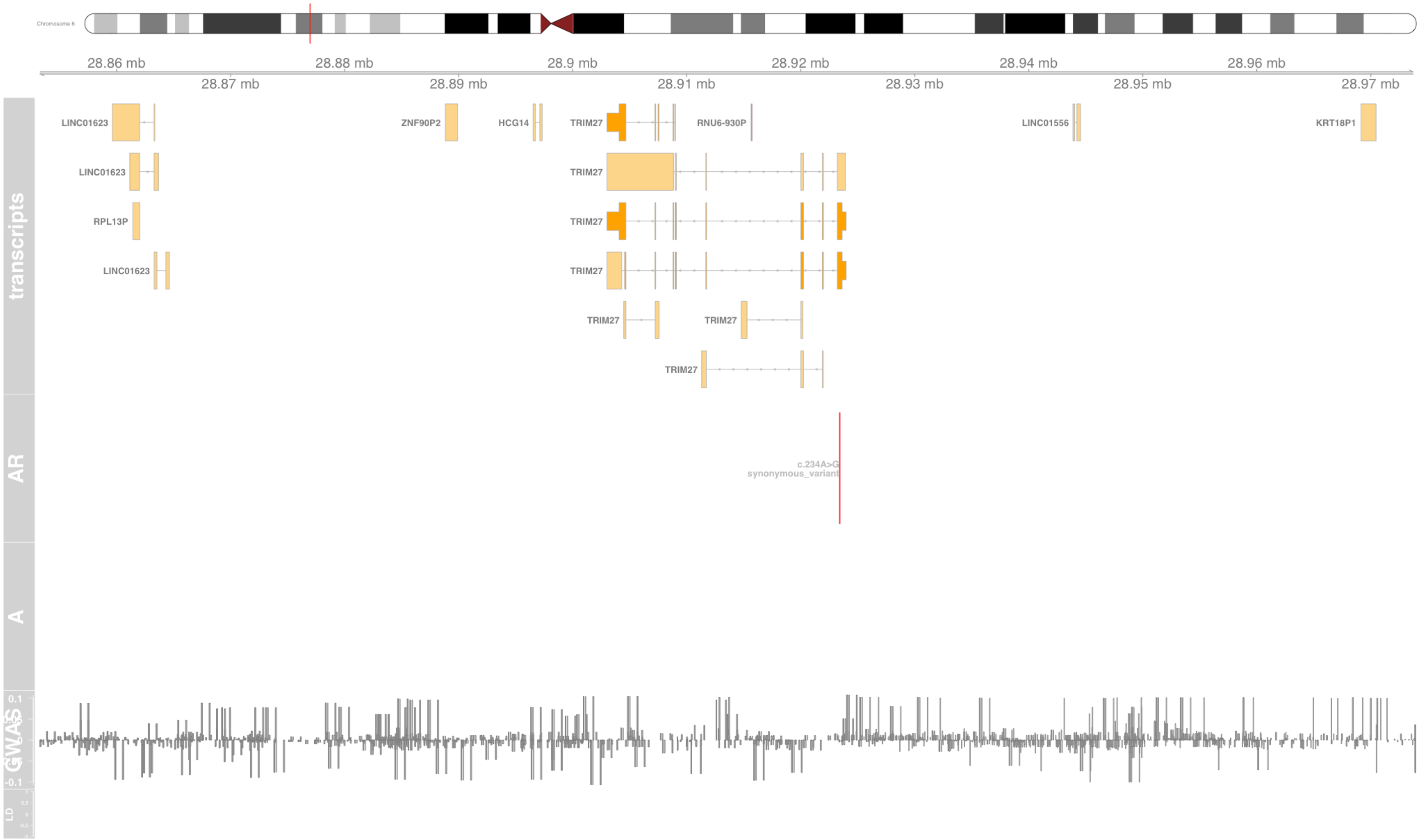

Fig S119 TRIM31

tripartite motif containing 31 [Source:HGNC Symbol;Acc:HGNC:16289]

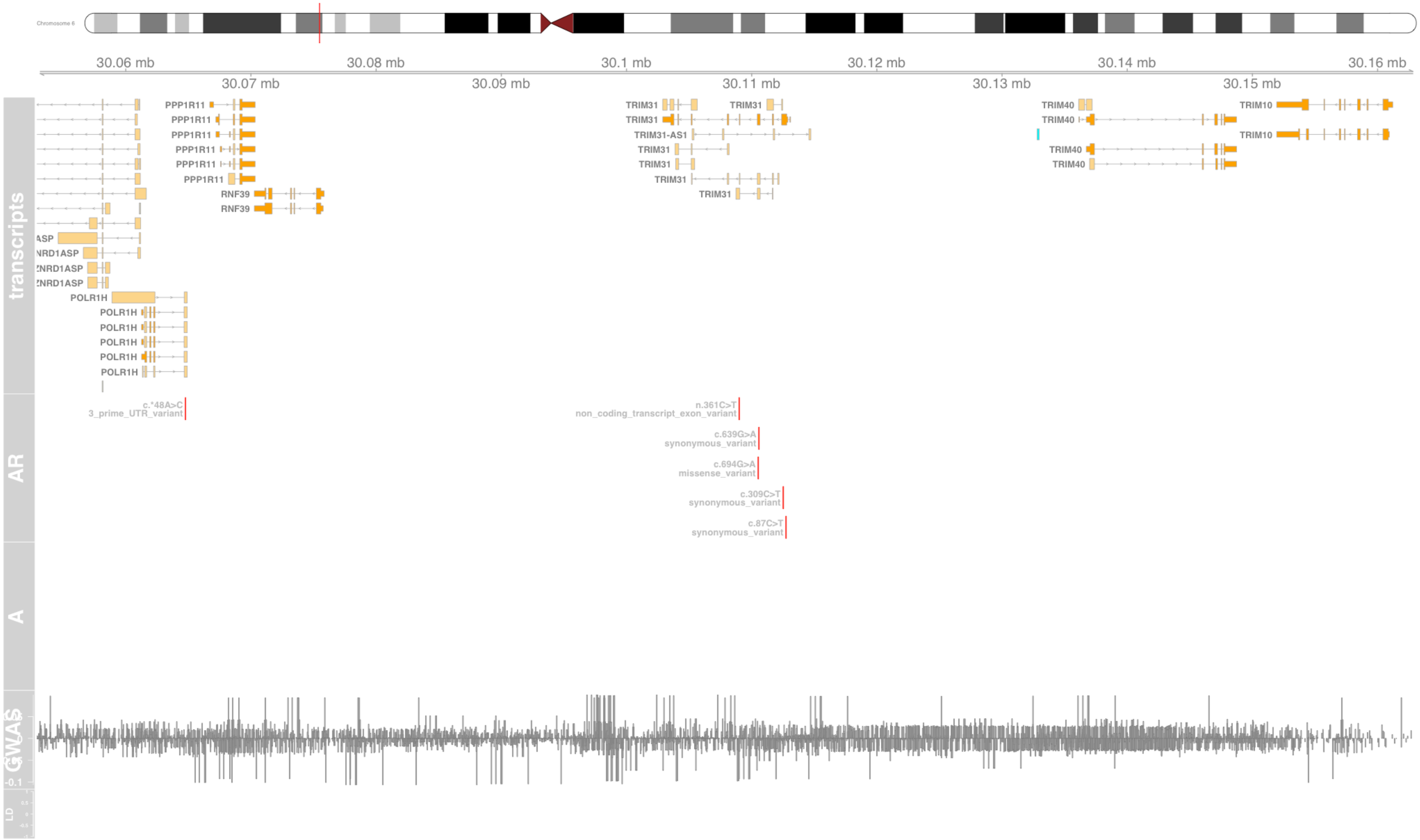

Fig S120 TSPAN8

tetraspanin 8 [Source:HGNC Symbol;Acc:HGNC:11855]

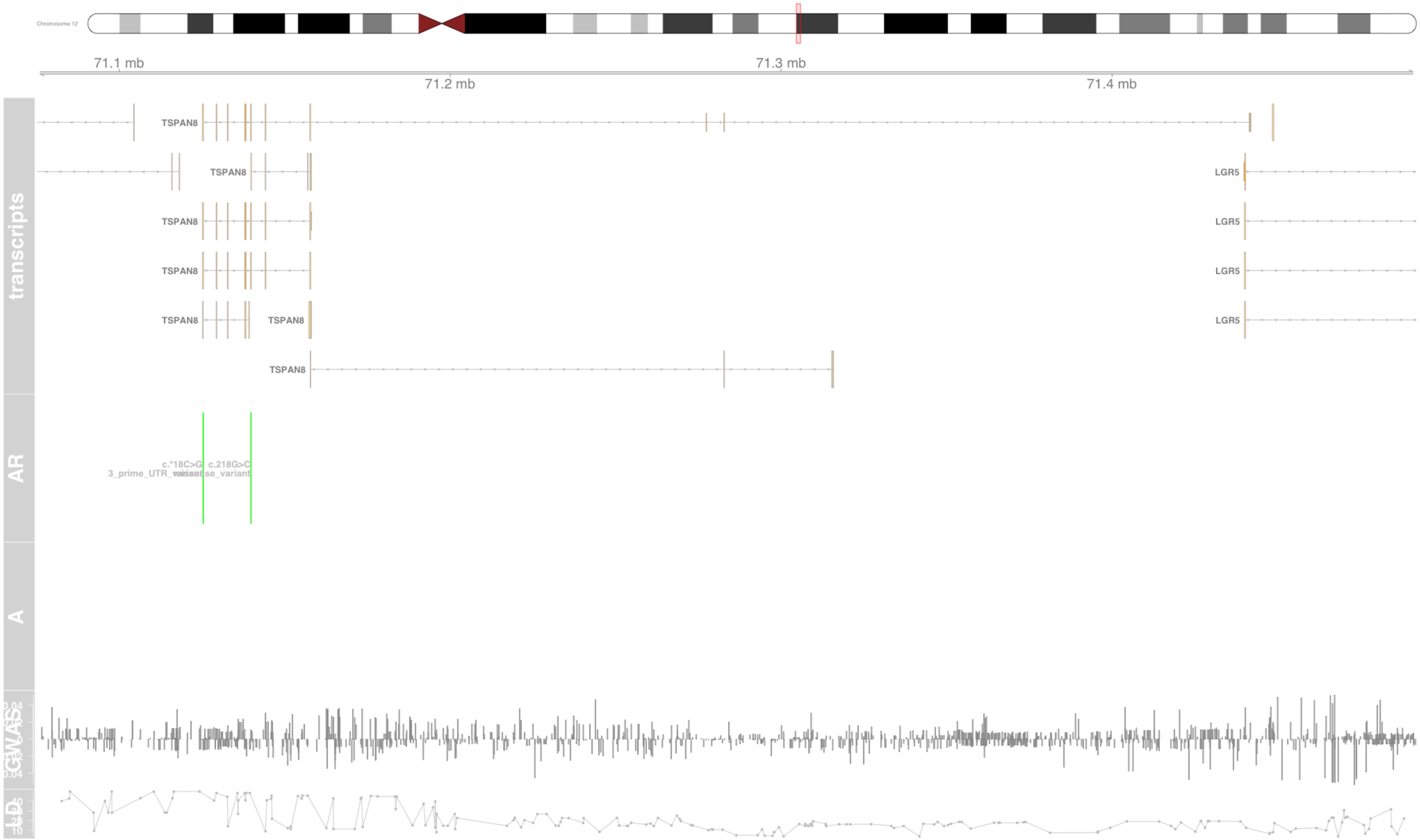

Fig S121 UBD

ubiquitin D [Source:HGNC Symbol;Acc:HGNC:18795]

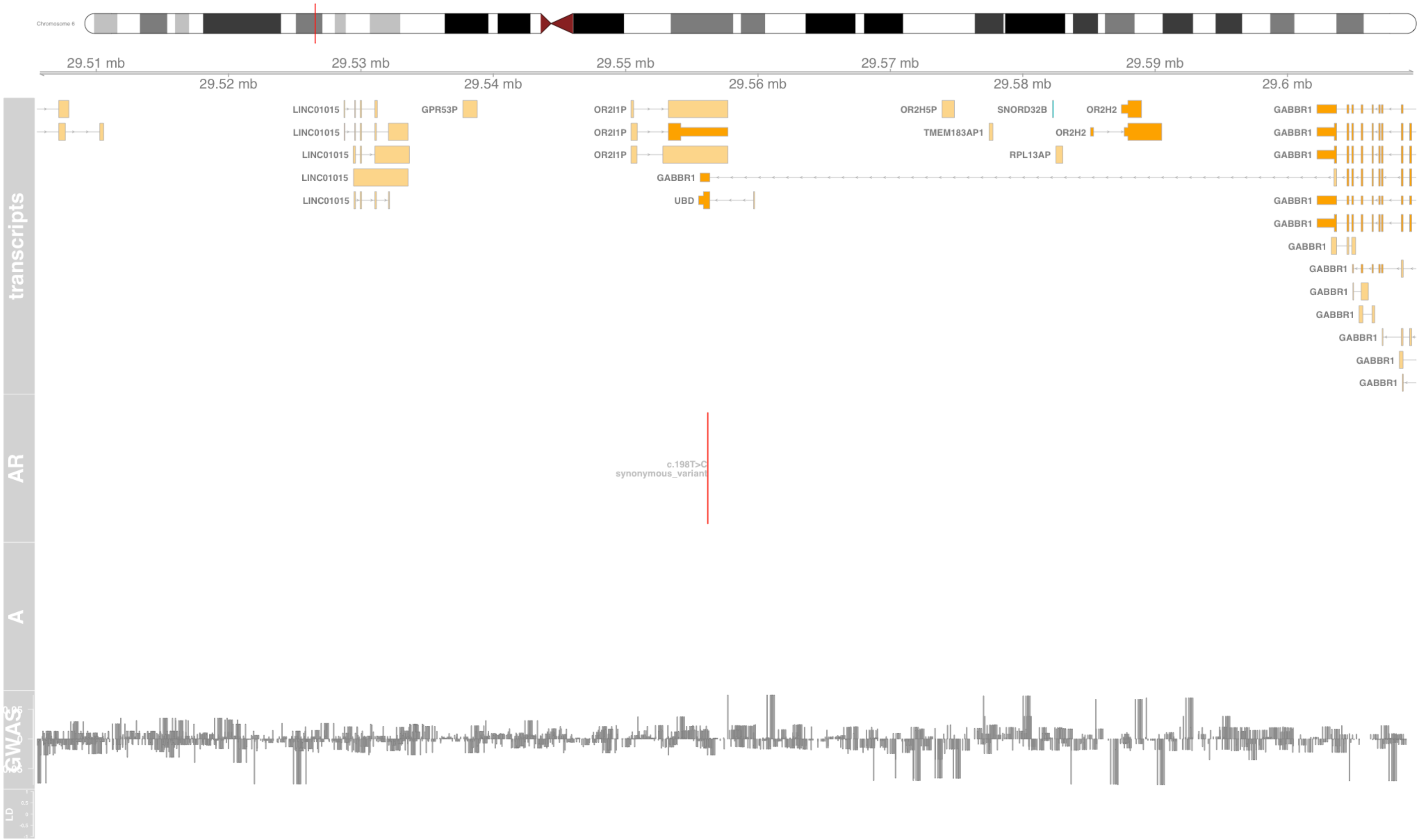

Fig S122 UGT3A1

UDP glycosyltransferase family 3 member A1 [Source:HGNC Symbol;Acc:HGNC:26625]

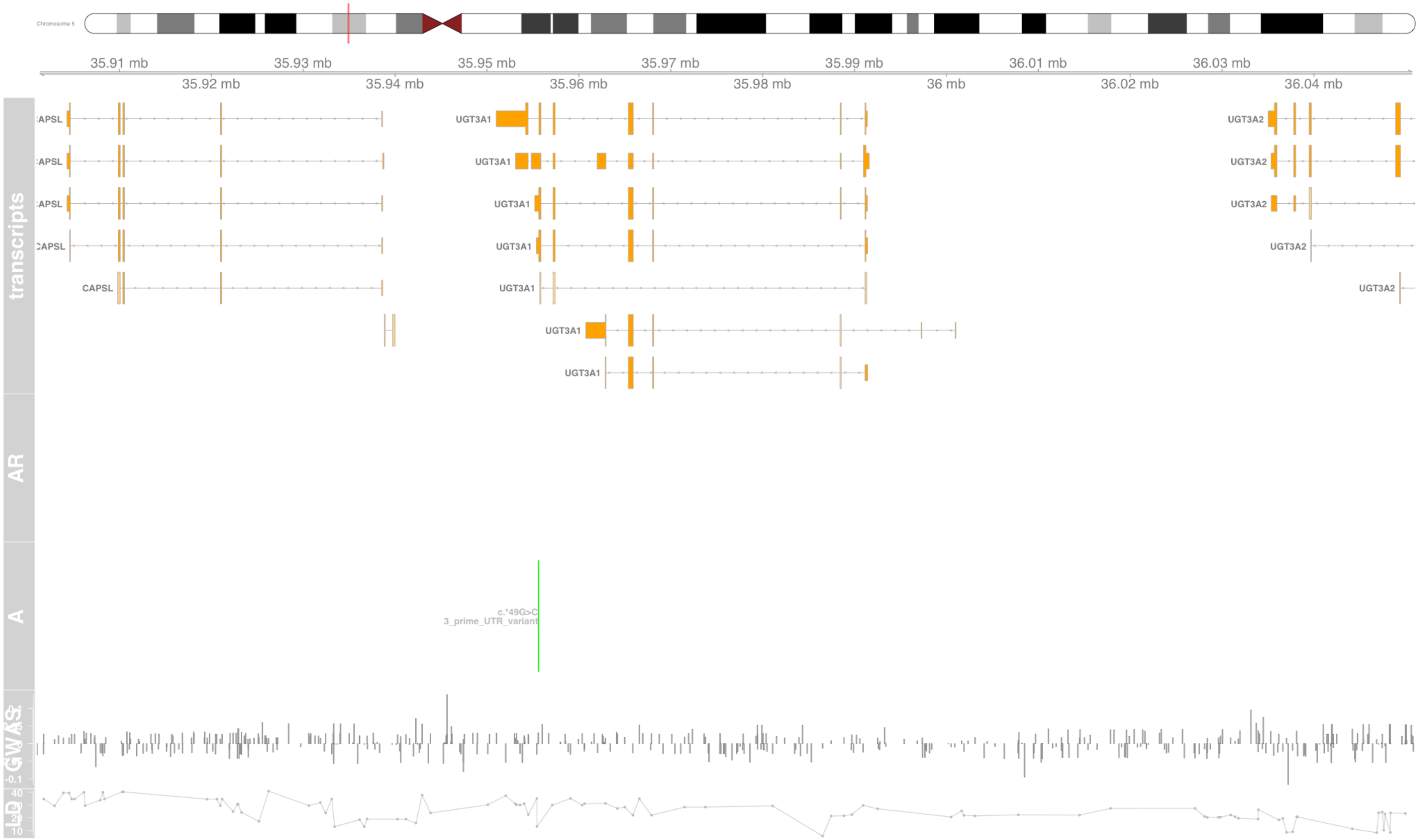

Fig S123 VWA7

von Willebrand factor A domain containing 7 [Source:HGNC Symbol;Acc:HGNC:13939]

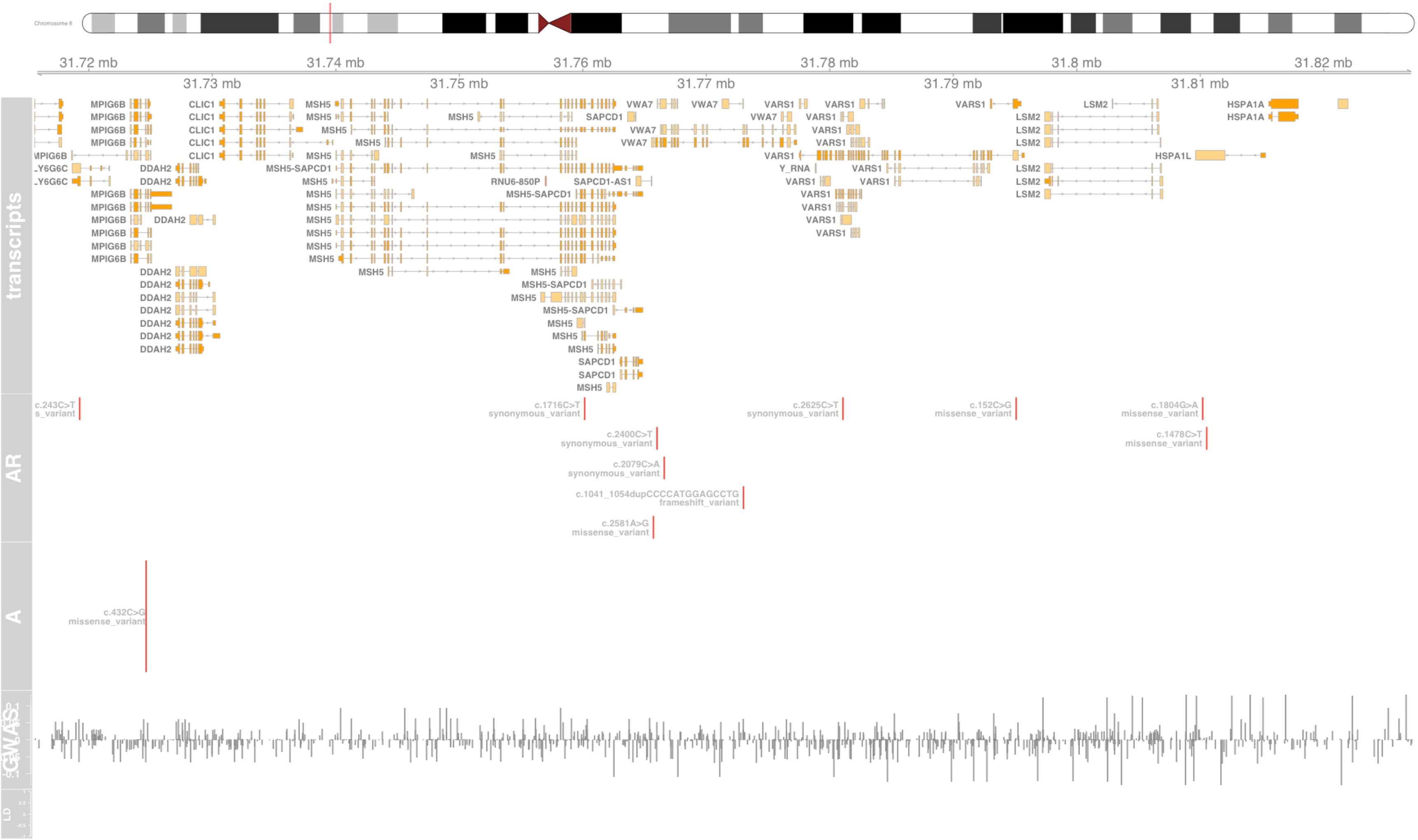

Fig S124 WDR36

WD repeat domain 36 [Source:HGNC Symbol;Acc:HGNC:30696]

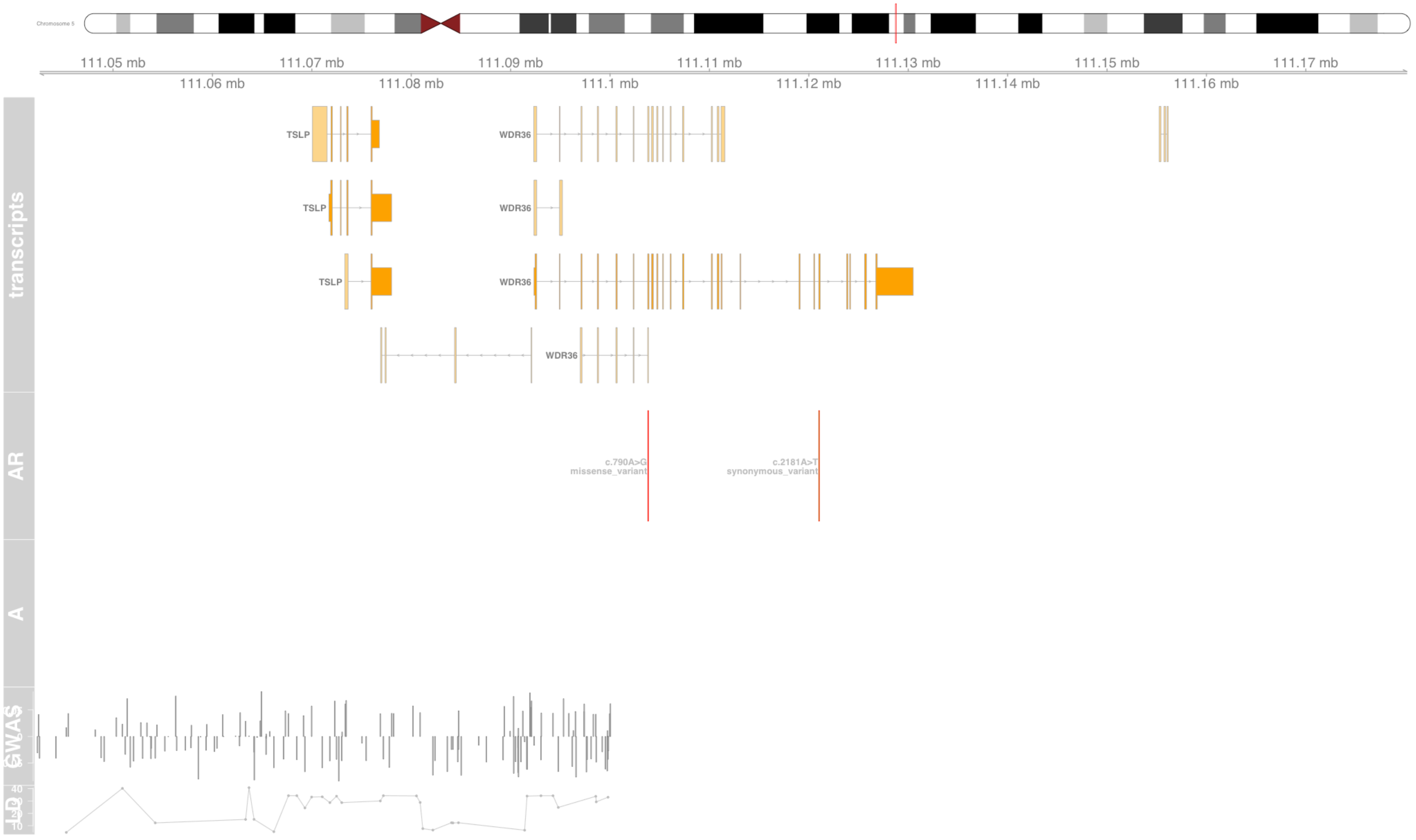

Fig S125 ZBED9

zinc finger BED-type containing 9 [Source:HGNC Symbol;Acc:HGNC:13851]

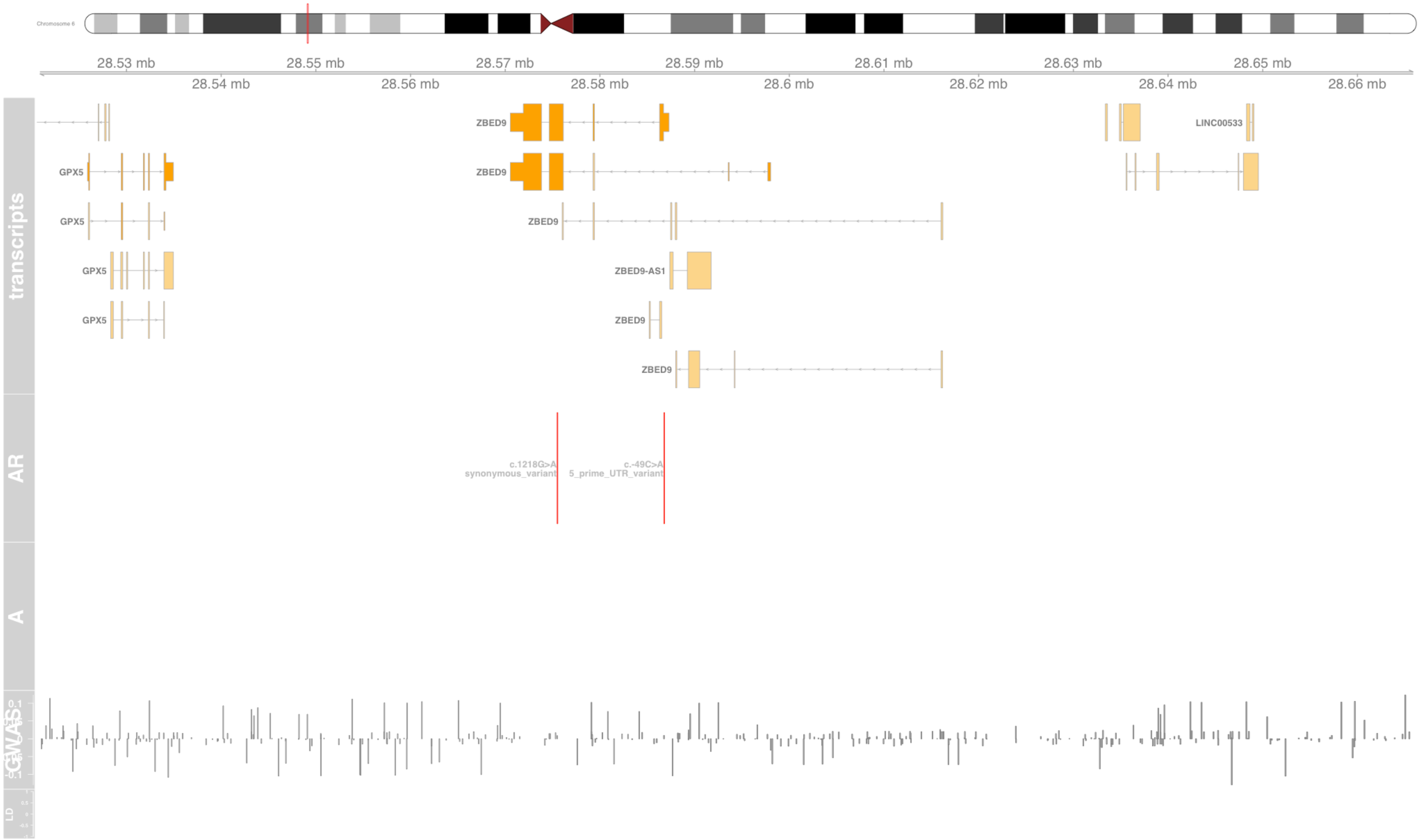

Fig S126 ZPBP2

zona pellucida binding protein 2 [Source:HGNC Symbol;Acc:HGNC:20678]

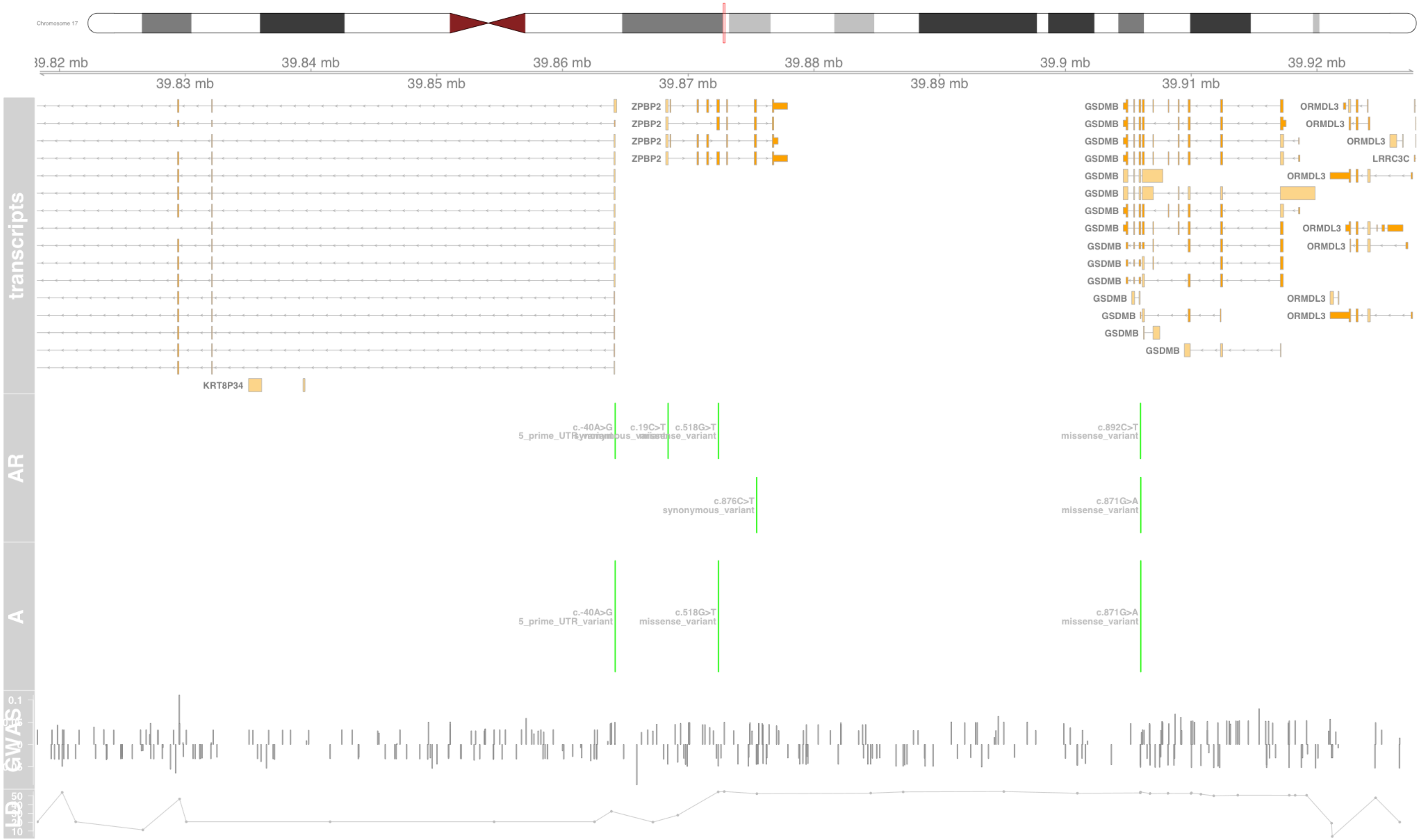

Fig S127 ZSCAN12

zinc finger and SCAN domain containing 12 [Source:HGNC Symbol;Acc:HGNC:13172]

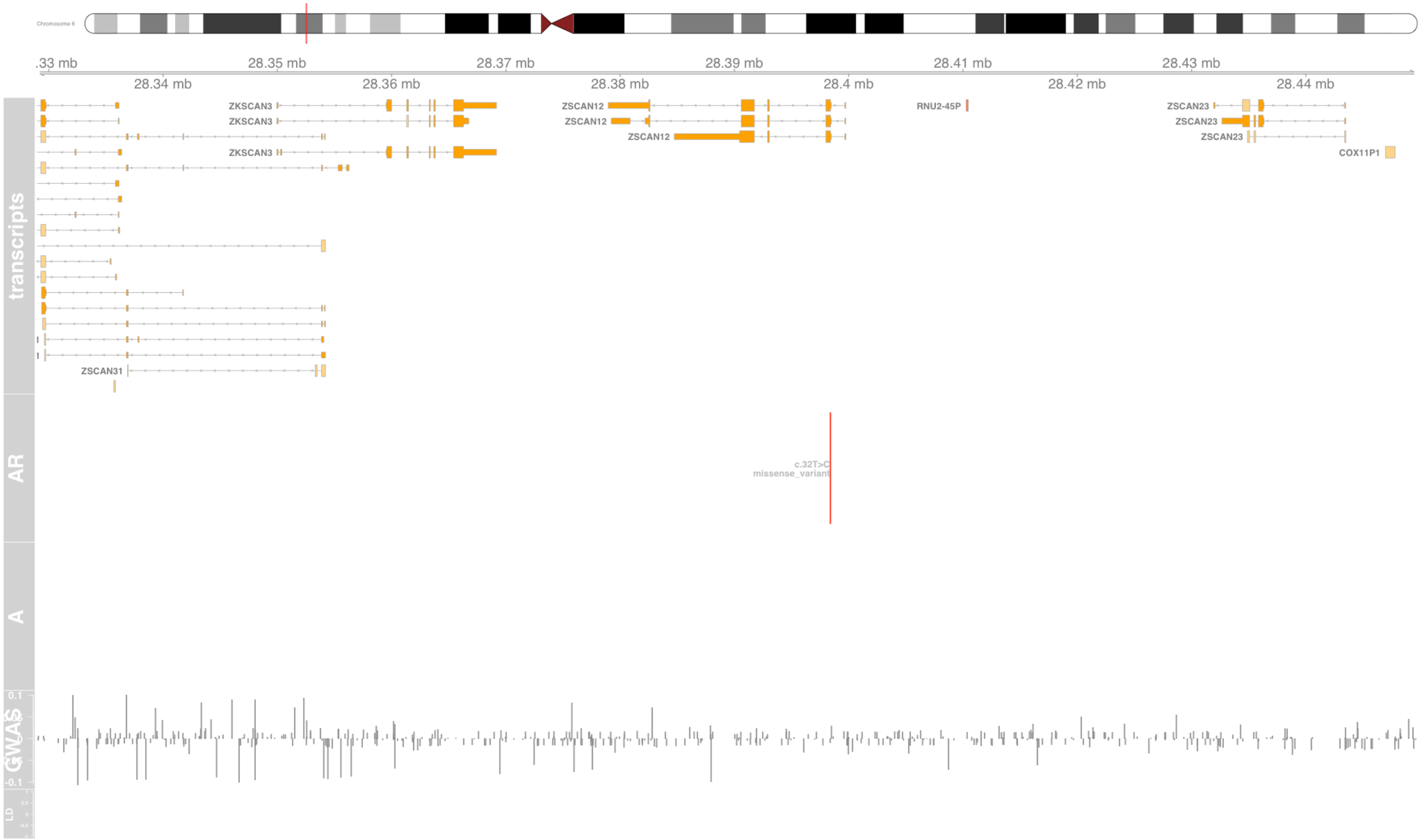

Supplement: Supplementary file 1 — Supplementary Information. [file 41598_2022_24960_MOESM1_ESM.pdf]
